# Molecular Markers: An Overview of Data Published for Fungi over the Last Ten Years

**DOI:** 10.3390/jof8080803

**Published:** 2022-07-29

**Authors:** Manuela Oliveira, Luísa Azevedo

**Affiliations:** 1 i3S—Instituto de Investigação e Inovação em Saúde, Universidade do Porto, 4200-135 Porto, Portugal; lazevedo@ipatimup.pt; 2 Ipatimup—Instituto de Patologia e Imunologia Molecular, Universidade do Porto, 4200-135 Porto, Portugal; 3 FCUP—Departamento de Biologia, Faculdade de Ciências, Universidade do Porto, 4200-135 Porto, Portugal

**Keywords:** detection, identification, fungi, genotyping, molecular marker

## Abstract

Fungi are amongst the most abundant and diverse organisms. Despite being widely known for their adverse role in food spoilage or as pathogens for humans, animals, or plants, they also present several beneficial effects. Fungi contribute to human well-being due to their role as decomposers, degrading decay matter into smaller molecules which can be easily used by other ecosystem members. These organisms can produce medicinal compounds or modulate protective immune responses in human intestine. Fungi intervene in diverse food processes or act as a food supply. Due to fungal diversity, the unequivocal identification of these organisms is crucial to increasing their practical applications and decreasing their adverse effects. The process of identification could be achieved through the integral sequencing of fungi genomes. However, this procedure would be time-consuming and rather cost-inefficient. Therefore, several molecular markers have been developed to overcome these limitations. The chronology of DNA-based molecular markers development can be divided into three main steps: (1) prior to the development of the PCR technique (RFLP); (2) after the development of the PCR technique (RAPD, AFLP, ISSR, VNTR, SNP, InDels, and DNA barcoding); (3) after the development of the massive parallel sequencing technique (Metabarcoding and WGS). Therefore, the present review covers an overview of the most recently developed molecular markers used for fungal detection and identification.

## 1. Introduction

When considering the biodiversity on Earth, Fungi are the third most diverse group of eukaryotic organisms, with approximately 140,000 known species, although the estimates can be as low as 700,000 and as high as 12 million [1,2,3,4]. Fungi can be found in terrestrial, e.g., [5], freshwater, e.g., [6] and marine ecosystems, e.g., [7]. In these ecosystems these organisms are important pathogens for humans, animal and plants, and also important carbon and nutrient recycling agents [8].

Molecular markers are the portions of DNA sequences dispersed along the genome used to identify a given organism [9]. As the number of molecular markers increases, the identification, characterization, and detection of essential fungi become timelier and more straightforward [10,11].

In this work, a systematic revision of the literature was performed to identify the most commonly used molecular markers and their use in Kingdom Fungi. Between March and June 2022, the online Google Scholar database was searched using the keywords “Restriction Fragment Length Polymorphism; fungi”, “Random Amplification of Polymorphic DNA; fungi”, “Amplified Fragment Length Polymorphism; fungi”, “Inter-Simple Sequence Repeats; fungi”, “Minisatellites; fungi”, “Microsatellites; fungi”, “Single-nucleotide polymorphisms; fungi”, “InDels; fungi”, “DNA barcoding; fungi”, “Metabarcoding; fungi”, and “; “Whole Sequencing Genome; fungi” limited to the period between 2012 and 2022 and the resulting articles were ordered by relevance. More than 4400 articles were initially selected. After an exhaustive manual curation was performed on this list, all the selected articles were read and analyzed. A total of 1332 papers were considered for the preparation of this study.

## 2. DNA-Based Molecular Markers

### 2.1. Restriction Fragment Length Polymorphism (RFLP)

Restriction Fragment Length Polymorphism (RFLP) was developed by Alec Jeffreys in 1984. In this technique, DNA samples are digested with specific endonucleases resulting in a profile of fragments of different lengths which is characteristic of each species. RFLPs have the advantage of resulting in medium polymorphic variability and do not require prior knowledge of the genome sequence analyzed. However, as disadvantages, these markers have high development and running costs and require high quality and quantity of DNA [12].

Polymerase chain reaction-restriction fragment length polymorphism (PCR-RFLP) is based on Polymerase Chain Reaction (PCR) amplification of a target region containing polymorphic sites followed by digestion with restriction enzymes and fragment separation by electrophoresis. Primers are designed to flank the polymorphic sites and positioned to create unequally sized fragments upon restriction endonuclease cleavage of the PCR products [13].

Terminal-RFLP (T-RFLP), the automated version of RFLP, involves an intermediate restriction digest after PCR and before the separation of the fragments. The procedure can be applied to rRNA genes and nuclear ribosomal internal transcribed spacer (ITS) region, with amplified fragments subjected to digestion with one or more restriction enzymes and the fragments separated by gel electrophoresis. T-RFLP develops this by separating fragments using an automated sequencer that detects fluorescently labeled (using fluorescently labeled primers during PCR) amplicons. In T-RFLP analysis, the fragments are determined by the peak height and intensity (relative fluorescence). Each set of restriction fragments creates specific pattern fingerprints [14].

The Multiplex PCR-terminal restriction fragment length polymorphism (MT-RFLP) was developed to simultaneously analyze a significant number of samples in a single reaction, reducing the cost and time of the study. Multiplexing allows performing one common reaction for various groups of microorganisms in a condition when the parameters of the reaction will be appropriate for each tested element. The multiplexing might be applied to PCR, and each subsequent step is performed with a mixture of multiplex PCR products. Alternatively, single reactions (PCR and restriction) can be carried out and followed by multiplex fragment analysis of pooled samples [15].

**Figure 1:**
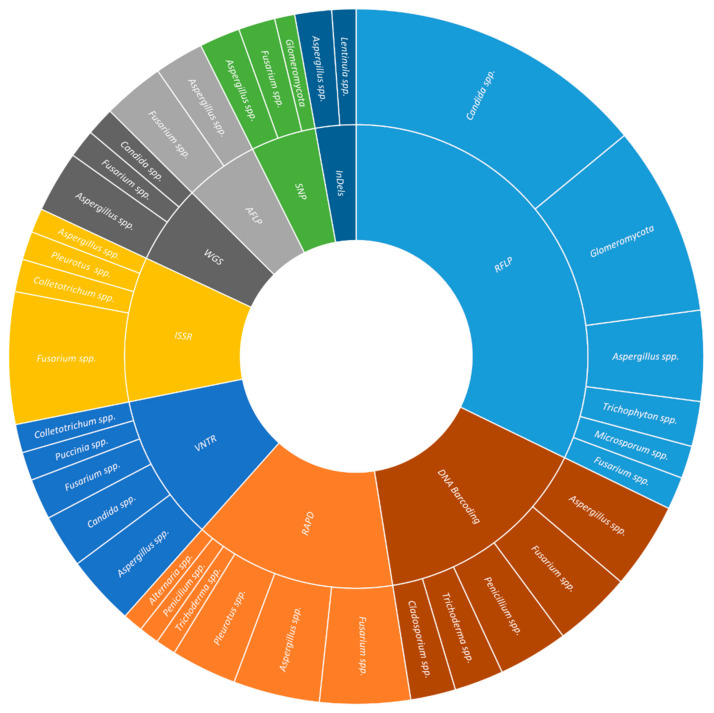
Distribution of the main fungal species according to the molecular markers used. The number of published articles for each molecular marker was normalized to allow a direct comparison between the different molecular markers (only normalized values higher than 1 are represented).

Over the last ten years, RFLP and their derived techniques have been used to identify and detect fungal species associated with human health (41.6% of the papers analyzed), the study of soil microorganisms (28.7%), and plant health (11.9%). In human health, the most frequently studied fungi were *Candida* spp. (48.4%), the etiological agent of diseases such as candidiasis and candidemia, *Trichophyton* spp. (15.6%), responsible for dermatomycosis and onychomycosis, and *Aspergillus* spp. (10.2%), associated with superficial and invasive infections. In the soil, aspects such as the effects of geographical distance (12.5%), plant composition (12.5%), and application of fertilizers (9.4%) on microbial composition were studied. The most studied plant pathogens were *Colletotrichum* spp. (20.8%), *Fusarium* spp. (12.5%), and *Penicillium* spp. (8.3%) (Figure 1 and Figure S1A; Table S1).

### 2.2. Random Amplification of Polymorphic DNA (RAPD)

Random Amplification of Polymorphic DNA (RAPD) was simultaneously developed by the teams of Welsh and McClelland [16] and Williams and co-workers, in 1990 [17]. This technique uses short random PCR primers (8–15 nucleotides) complementary to several genomic regions, generating complex PCR profiles characteristic to each species [18].

This technique uses high polymorphic molecular markers and requires a medium quantity of DNA, and presents intermediate technical development and running costs. Among the disadvantages is the low reproducibility of the results [18].

Since 2012, RAPD markers have been commonly used for the study of fungal genetic diversity (34.06% of the papers analyzed), the study of diverse plant pathogens (29.0%), and the authentication or safety verification of products such as food and drinks (15.9%). Genetic diversity in fungi was the focus of studies related to diseases in plants and humans (i.e., *Fusarium*—19.0%; *Aspergillus*—9.5%; *Rhizoctonia*—7.1%). These studies include vegetative compatibility, fingerprinting of toxigenic and non-toxigenic strains, and antifungal susceptibility. Among the plant pathogens, *Fusarium* spp. (26.9%) was associated with wilts in different crops and pokkah boeng disease in sugarcane; *Alternaria* spp. (7.7%), associated with diseases such as brown spot, leaf spot, and black rot or being endophytic fungi; and *Phaeoacremonium* spp. (7.7%), responsible for grapevine diseases such as esca. In the case of the identification of food products, the main species identified were the edible mushrooms *Pleurotus* (30.8%) and *Agaricus* (3.8%), while the main species concerning food contamination and spoilage were *Aspergillus* spp. (17.3%) and *Penicillium* (7.7%) (Figure 1 and Figure S1B; Table S2).

### 2.3. Amplified Fragment Length Polymorphism (AFLP)

Amplified Fragment Length Polymorphism (AFLP) was developed by KeyGene in 1990 [19]. In this technique, DNA samples are digested using two restriction enzymes after the annealing of adapters, which create cut boundaries that act as primer binding sites for PCR amplification. Polymorphisms are recognized by the presence or absence of DNA fragments following analysis on polyacrylamide gels [20].

These markers are highly polymorphic and abundant in the genome. The main limitations of this technique are the high development and running costs, high quality and quantity of DNA requirements, prior knowledge of the DNA sequence, and show intermediate reproducibility and low automation capacity [21,22].

AFLP markers have been mainly used for the study of plant health (50.5%), fungal genetic variability (12.6%), and food and beverage products (10.7%). Concerning phytopathogens, the most frequently studied crop diseases were rots (14.3%) caused by fungal species such as *Coniophora* species-complex, *Fusarium* spp., *Macrophomina phaseolina*, *Nigrospora oryzae*, and *Sclerotinia sclerotiorum*; rusts (11.9%) induced by *Veronaea botryose*, *Puccinia striiformis*, *Hemileia vastatrix*, and *Phakopsora pachyrhizi*; and anthracnose (9.5%) caused by species belonging to the *Colletotrichum* genus; rice blast (8.1%) caused by *Magnoporthe* spp.. In the case of fungal genomic diversity were studied aspect as the difference among strains among the same species (41.2%), differential gene expression (17.6%), phylogeny (11.8%), or population structure (11.8%) (Figure 1 and Figure S1C; Table S3).

### 2.4. Inter-Simple Sequence Repeats (ISSR)

Inter-Simple Sequence Repeats (ISSR) was developed by Ztetikiewicz and colleagues in 1994 [23]. This technique consists of the amplification of a DNA segment located at an amplifiable distance between two identical microsatellites (16–25 bp long) oriented in opposite directions [23].

The microsatellite markers present several advantages over the above-mentioned markers: high polymorphism level and low development and running costs, and requirements for low quality and quantity of DNA [24].

Since 2012, ISSR have been used mainly to study plant pathogens (42.2%) and fungal genetic diversity (25.0%), and to identify food and beverage products (17.2%). Amongst the 51 fungal species studied, the most representative ones were *Fusarium* spp. (15.5%) as the etiological agent of the yellowing disease, wilt, and storage rot in different cultures, *Colletotrichum* (13.8%), causing bitter rot and anthracnose, and *Alternaria* (5.2%), responsible for symptoms of brown leaf spot and blight. In terms genomic diversity, the most common fungi studied were *Fusarium* (14.7%), *Aspergillus* (11.8%), and *Sclerotium* (8.8%) comprehending aspects such as genetic diversity (65%), virulence (10%), and vegetative compatibility (5%). Finally, in the field of the identification of food and beverage products, the most frequently studied fungal species were *Pleurotus* (21.9%), *Agaricus* (9.4%), and *Aspergillus* (9.4%) (Figure 1 and Figure S1D; Table S4).

### 2.5. Variable Number of Tandem Repeats (VNTR)

Variable Number of Tandem Repeats (VNTR) includes minisatellites and microsatellites. Minisatellites, first described by Alec Jeffreys and his team in 1990, are repeat motifs mostly about 9 to 30 bp long [25,26]. Microsatellites (Simple Sequence Repeats (SSRs) or Short Tandem Repeats (STRs)), first described by Litt and Luty in 1989, are repeat motifs mostly about 2 to 4 bp, consisting of tandem repeats of mono-, di-, tri-, tetra-, or pentanucleotide units arranged throughout the genome [27].

Microsatellites are generally abundant and polymorphic in non-transcribed genomic regions, the reason why this marker is considered selectively neutral. Nevertheless, SSR loci can also occur in genomic regions involved in transcription, translation, chromatin organization, or recombination [28]. Due to replication slippage, SSRs loci mutate from 10–100 thousand times more frequently per generation than single-nucleotide substitutions [29]. Their high mutation rates and neutral evolution allow the accumulation of numerous population-specific (that is, private) alleles, which are significant for unveiling hidden population structures. Due to their multi-allelic nature, there is a higher probability to detect heterozygosity than, for instance, an equal number of bi-allelic markers. However, the unusually high variability of SSRs concerning other genomic regions might not necessarily reflect patterns of genome-wide genetic diversity [30,31,32].

Furthermore, the rapid mutation rates of SSRs may also be a confounding signal of population structuring and divergence. For instance, frequent forward and backward mutations can create identical alleles in unrelated or genetically isolated (that is, homoplasy) populations. This undesirable effect can be compensated by increasing the number of polymorphic SSR loci used, but populations’ level of genetic differentiation that diverged a long time ago could still be underestimated [33].

Minisatellites and microsatellites show a high level of polymorphism and genomic abundance, low requirements in terms of both DNA quality and quantity, and high reproducibility.

Over the last ten years, ISSR markers have been used in studies of plant (40.2%) and human health (15.0%), fungal diversity (15.0%), and food and beverage (8.4%). The most commonly plant pathogens studied were *Puccinia* (7.4%), the etiological agent of rust; *Colletotrichum* (6.4%) causing anthracnose; *Fusarium* (6.4%), responsible for diseases such as blight, rot, and wilt; *Alternaria* (3.2%), the etiological agent of blight and rot, *Diaporthe* (3.2%) as endophytic fungi; *Ustilago* (3.2%), inducing smut; and *Rhizoctonia* (3.2%), causing aggregate sheath spot and blight. The human pathogens most frequently studied were *Candida* spp. (40.0%), the etiological agent of candidiasis and candidemia; *Aspergillus* spp. (17.1%), inducing invasive infections; *Cryptococcus* spp. (14.3%), causing cryptococcosis. As for fungal genetic diversity, the most studied species were *Aspergillus* (13.5%), *Fusarium* (13.5%), and *Epichloë* (8.1%), comprehending topics such as genetic diversity (43.6%), mating-type (20.5%), and population structure (12.8%) (Figure 1 and Figure S1E; Table S5).

### 2.6. Single-Nucleotide Polymorphisms (SNP)

Single-nucleotide polymorphisms (SNP), developed by Lander in 1996, result from changes in a single nucleotide position in the DNA sequence [34]. These markers occur twice as frequently in intergenic and non-coding regions of the genome than in coding regions [35]. However, genome-wide association studies revealed that occasionally SNPs located in non-coding regions are often physically linked to functional or regulatory genomic sites, thus reflecting, for example, selection signatures [36]. Given that SNPs are mostly bi-allelic, traditional population genetic statistics can easily be applied to them, but a greater number of loci sufficiently polymorphic might be necessary to reach the same power as multi-allelic SSR loci [29]. The advent of next-generation sequencing techniques has considerably accelerated, simplified, and automated genome-wide SNP detection and genotyping. However, considering that also a relatively small number of highly polymorphic SNPs can potentially give a similar genetic resolution as randomly chosen and multi-allelic SSRs [37], an alternative strategy to genome-wide SNP screening might be targeting polymorphic sites in unlinked single-copy genes, generally known to be conserved in the targeted phylum [38]. As a result of single nucleotide replacements, these markers are biallelic, but rare cases exist of triallelism for the target position.

Single-nucleotide polymorphisms are co-dominant markers with high level of polymorphism and very high genomic abundance. The analyses of these markers require a low quantity of DNA, allowing a high automation capacity resulting in very reliable and reproducible data.

In the selected period, SNP markers have been mainly used for the study of plant (42.9%) and human health (16.0%), and genomic variability (10.1%). Concerning plant pathogens, the most frequently studied fungal pathogens were *Fusarium* spp. (15.3%) and *Monilinia* spp. (5.1%), being the most prevalent diseases rusts (11.5%) and blight (7.7%). The most common studied crop was wheat (20.0%). In the case of plant pathogens, *Aspergillus* spp. (19.4%) and *Exophiala* spp. (16.1%) were frequently studied. The most studied diseases were aspergillosis (33.3%) and candidiasis (26.7%). Genomic diversity studies were conducted in *Glomeromycota* (50.0%) and *Fusarium* spp., covering aspects such as species genetic diversity (36.4%), population structure (27.3%), and toxin production (18.2%) (Figure 1 and Figure S1F; Table S6).

### 2.7. Small Insertions or Deletions (InDels)

The molecular markers Insertions or Deletions are fragments of different sizes (ranging from 1 to 1000 bp) inserted or lost at a given location in the genome. Given its nature and the unlikeliness of recurrence or back mutation, these markers are very stable within the genome and can, therefore, be relevant for population studies [39].

These markers are co-dominant, with high polymorphism and very abundant, presenting both high reliability and reproducibility.

In the last ten years, InDels markers have been used in studies of plant (29.0%) and human health (17.0%), and food and beverage (17.0%). The most commonly studied plant pathogens were *Fusarium* spp. (16.7%), *Botrytis* spp. (10.0%), and *Puccinia* spp. (10%). The majority of the published papers cover aspects such as genetic diversity (23.7%), the distinction between the species of the complex (7.9%), resistance to fungicides (7.9%), in crops wheat (21.4%), oilseed rape (14.3%), and soybean (14.3%). The most frequently studied human pathogens were *Cryptococcus* spp. (19.2%), *Aspergillus* spp. (11.5%), and *Trichophyton* spp. (11.5%). This marker was used to elucidate aspects such as genetic diversity (40.0%), recombination (15.0%), and virulence factors (15.0%). The diseases included were aspergillosis (25.0%), diarrhea (16.7%), and other enteric diseases (16.7%) (Figure 1 and Figure S1G; Table S7).

### 2.8. DNA Barcoding

Theoretically, DNA barcoding relies on using a single universal marker—the DNA barcode sequence—for rapid and accurate species identification and classification, mainly by non-taxonomist [40]. “DNA barcode” implies using a standardized DNA sequence similar to the 11-digit Universal Product Code that identifies retail products in the supermarket [41]. This technique comprehends two methodological steps. Initially, the DNA barcode library of known species is constructed, and then the barcode sequence of the unknown sample is matched against this library for identification purposes [42].

For animals, the universal standard barcode is cytochrome c oxidase 1 (COI). Many strategies have been suggested for plants, some based on a single chloroplast genomic region or a combination of different regions [43]. For phytoplasmas, the universal DNA barcode is based on the elongation factor Tu (TUF) and SSU (16S rRNA); for archaea and bacteria is used the SSU gene, with two different sets of primers, one for bacteria (targeting the V3–V4 hypervariable regions) and another for archaea (targeting the V1–V2 hypervariable regions) [43,44]. Fungal ITS is frequently proposed as the first universal barcoding marker. This genomic region is known to be easily amplified and sequenced, providing acceptable resolution in a wide range of taxa. Nonetheless, ITS does not provide sufficient resolution in closely related species of indoor and food-borne molds (e.g., *Aspergillus* spp., *Penicillium* spp.), plant, human or animal pathogens (e.g., *Alternaria* spp., *Cladosporium* spp., *Colletotrichum* spp., *Fusarium* spp., and *Phytophthora* spp.) or other fungi (e.g., freshwater Sordariomycetes, *Trichoderma* spp. and slime molds). As such, it is common to resort to secondary barcoding markers, such as the intergenic spacer (IGS), β-tubulin II (TUB2), DNA-directed RNA polymerase II largest (RPB1) and second largest (RPB2) subunits, translational elongation factor 1α (TEF-1α), DNA topoisomerase I (TOP1), phosphoglycerate kinase (PGK), and cytochrome c oxidase subunit I (COX1) and subunit II (COX2), 28S nrDNA (LSU), and 18S nrDNA (SSU) [45,46].

These markers are co-dominant and present high genomic abundance, being their analysis highly reliable and reproducible. A disadvantage is that the same marker cannot be universally used for all fungal species, and prior knowledge of the DNA sequence is required [47].

Over the considered ten years, DNA barcoding has been used in studies on plant (21.4%) and human health (13.4%), and food and beverage (12.5%). Regarding plant health, the most used barcode genes were ITS (36.2%), TEF-1α (13.8%), and LSU (12.1%), applied to *Fusarium* spp. (11.6%), *Alternaria* spp. (2.9%), *Curvularia* spp. (2.9%), *Diaporthe* spp. (2.9%), *Exserohilum* spp. (2.9%), *Mycosphaerella* spp. (2.9%), and *Neofusicoccum* spp. (2.9%) fungal species collected from several crops (e.g., blueberry, chili, sorghum, *Pinus*). The aims of such studies were diagnosis (15.8%), identification of leaf spot pathogens (15.8%), and quarantine species (10.5%). In human health, the most common barcode genes were ITS (33.3%), β-TUB (18.5%), and TEF-1α (14.8%). The human pathogens included were *Scedosporium* spp. (9.7%), *Aspergillus* spp. (6.5%), *Cunninghamella* spp. (6.5%), and *Fusarium* spp. (6.5%); whereas, the major diseases were invasive fungal infections (28.6%), keratitis (21.4%), and opportunistic fungi (14.3%). In the category of food and beverage production and spoilage, the barcodes were mainly ITS (43.3%), LSU (20.0%), and β-TUB (10.0%), applied to the study of *Lactarius* spp. (2.7%), *Penicillium* spp. (2.7%), and *Pleurotus* spp. (2.7%) associated either with edible mushrooms (53.3%) or dairy products (20.0%) (Figure 1 and Figure S1H; Table S8).

### 2.9. Massive Parallel Sequencing (MPS)

The developments of massive parallel sequencing (MPS) techniques allow a more profound knowledge of the microorganisms, either unraveling the entire microbial composition retrieved from a given environment both in terms of identification and quantification (relative abundance) [48] or determining the complete genome sequence of a single microorganism, aiming for a complete portrait of the gene present mainly considering aspects such as metabolic profile, virulence, antifungal resistance, or recombination [49].

The advantages of MPS include the high-throughput capacity; a single protocol can be applied for all microorganisms for identification and genotyping purposes; no need for DNA cloning (only require libraries preparation); no need for an a priori knowledge about the sequence of a particular gene/genome (MPS can read the DNA templates randomly distributed throughout the entire genome); no need for isolation and culture of the microorganism to be studied (many strains are unable to grow in culture media); and reduced costs (less than US $1000 per genome) and the turnaround time (only a few hours) [50,51]. On the contrary, these techniques are associated with data storage and analysis (the considerable amount of data obtained, the process profoundly depends on storage ability and the bioinformatics capacity to produce valuable data) and the biases introduced by each step of the protocol (for instance, changing the DNA extraction kit or the sequencing platforms produces significantly different results) [52].

Metabarcoding corresponds to the automated identification of various organisms present in a single bulk sample or from an environmental sample with degraded DNA (i.e., soil, water, feces) using a species-specific genetic marker (DNA meta-barcode) [53].

As previously referred to, ITS can be indicated as the universal marker for fungi due to its high interspecific variability and conserved primer sites. However, due to length limits intrinsic to the existing sequencing platforms, only one of the two subunits (ITS1 or ITS2) can be analyzed in DNA metabarcoding [54]. The region that gives the best results in taxonomic resolution is still a matter of debate. ITS1 is the most frequently used for fungal community studies, followed by ITS2 and more rarely by the complete ITS region. However, the selection between ITS1 or ITS2 alters the read number (higher using ITS2) and the Shannon index (higher using ITS1), not altering the estimation of species richness [54,55,56].

Nowadays, several sequencing platforms and bioinformatics pipelines are available for the MPS of the fungal community. The 454 pyrosequencing from Roche Life Sciences (Basel, Switzerland) was the most used platform until 2013 when Illumina took the lead [57,58], a situation that persists today. MiSeq, from Illumina (San Diego, CA, USA), allows read lengths of nearly 2 × 250 bp at a relatively low cost, while 454 pyrosequencing provides longer reads (nearly 800 bp) at a higher cost and is not available in the market since 2016. Alternatives are Ion Torrent, from Thermo Scientific (Waltham, MA, USA), which allows read lengths of nearly 400 bp coupled with short run-time and decreased sequencing costs, and Pacific Biosciences (Menlo Park, CA, USA) or Oxford Nanopore Technologies (Oxford, UK), which allow longer reads (nearly thousands of kb) with low quality [54].

High-throughput DNA sequencing data rely on a bioinformatics pipeline’s efficient and accurate use [59]. General pipelines for DNA metabarcoding analyses of fungal communities frequently used are implemented in QIIME [60] and Mothur [61]. Alternatives especially developed for ITS sequences are CLOTU [62], PIPITS [63], and CONTAX [64], among others. Additionally, pipelines have been developed to serve specific purposes, such as studies of environmental microbial communities (BioMaS) [53] or human fungal microbiome (HumanMycobiomeScan) [65].

From 2012 to 2022, Metabarcoding studies included plant health (14.9%), food and beverage (14.4%), and soil (10.9%). In plant health studies, the most frequently used metabarcodes were ITS2 (31.0%) and ITS1 (27.6%) either isolated or in a combination of both (13.8%). The selected works included studies on fungal community (30.8%), endophytes (26.9%), trunk disease (11.5%) applied to forest trees (27.3%), cereals (22.7%), and fruit trees (13.6%). As for food and beverage, although in different proportions, the same metabarcodes were used (ITS2: 36.0%; ITS1: 16.0%; ITS1+ITS2: 12.0%). Sequencing was used for the identification of species involved in beverage fermentation (26.9%), food contamination (19.2%), and the production of dairy products (15.4%). In the soil’s fungal diversity studies, the three usual metabarcodes were used (ITS2: 20.0%; ITS1: 15.0%; ITS1+ITS2: 15.0%). The selected soils were forests (21.1%), agro-environment (15.8%), and public gardens (15.8%) (Figure 1 and Figure S1I; Table S9).

Whole genome sequencing includes two different techniques: de novo genome assembly, when the species to be studied has not been previously sequenced and assembled; or re-sequencing, which identifies genome-wide variants (copy number variants, structural variants, and SNPs and indels) comparing an existing reference assembly with a sequenced isolate through the alignment of sequence reads against the reference [49]. These variants are significant in studies such as fungal microevolution, fungicide resistance, virulence factors monitoring, and outbreak analysis [66]. Short-read platforms (e.g., Illumina) allow the detection of single base pair variants (SNVs). In contrast, long-read platforms (e.g., Pacific Biosciences or Oxford Nanopore) allow the detection of large structural variants, copy number variants, or pathogenicity islands [66].

In the same period, whole genome sequencing has been used to elucidate aspects associated with plant (32.9%) and human health (13.4%), and food and beverage (12.8%). Regarding plant health, the main fungal phytopathogens studied were *Fusarium* spp. (12.1%), *Trichoderma* spp. (6.1%), and *Venturia* spp. (6.1%); while the major diseases analyzed were rot (27.8%), blight (19.4%), and cancer (8.3%). The most common studied cultures were soybean (13.6%), grapevine (9.1%), sugarcane (9.1%), and wheat (9.1%). Concerning human health, the most common fungal species were *Candida* spp. (42.1%), the etiological agent of candidiasis and candidemia (42.1%), and *Aspergillus* spp. (15.8%) causing aspergillosis (10.5%) and other infections (5.3%). In the identification of food and beverages, species such as *Agaricus bisporus* (4.8%), *Auricularia* spp. (4.8%), *Flammulina* spp. (4.8%), among others, were studied for their interest as edible mushroom (55.0%); while *Aspergillus* spp. (23.8%) and *Penicillium* spp. (19.0%) were studied for their role in food spoilage (45.0%) (Figure 1 and Figure S1J; Table S10).

## 3. Conclusions

Identification of fungal species is a crucial aspect of many fields in science. Although whole genome sequencing techniques are now available at an amenable cost, this was not true thirty or twenty years ago. The scientific community has developed several molecular techniques to obtain information on these species. Nowadays, these markers range from non-PCR, PCR-based techniques to more advanced MPS-based techniques.

Over the past ten years, these molecular markers have been mainly used to study aspects related to plant (31.8%) and human health (13.7%), and genomic diversity (12.0%). Regarding plant health, the most commonly studied fungal phytopathogens were *Fusarium* spp. (14.9%), *Colletotrichum* spp. (6.2%), *Alternaria* spp. (3.7%), and *Puccinia* spp. (3.7%). Diseases such as rots (27.8%), rust (11.1%), wilts (11.1%), and cankers (10.2%) were included. The most frequent human pathogens studied over the last 10 years were *Candida* spp. (32.2%), *Aspergillus* spp. (12.2%), *Trichophyton* spp. (12.2%), and *Cryptococcus* spp. (4.2%). The most commonly studied diseases were aspergillosis (14.7%), candidiasis (11.6%), dermatophytosis (20.0%), and mucormycosis (8.4%). Genomic studies included *Aspergillus* spp. (13.0%), *Fusarium* spp. (12.4%), *Colletotrichum* spp. (2.5%), *Ganoderma lucidum* (2.5%), and *Lentinus* spp. (2.5%), covering aspects such as molecular diversity (59.7%), recombination (8.3%), antifungal resistance (5.0%), population structure (4.4%), and virulence (4.4%).

Soon, the most dated techniques (non-PCR and PCR-based techniques) will be surpassed by MPS-based techniques. However, some breakthroughs associated with increasing storage data availability, bioinformatic analysis, and standardization of protocols are required.

## Data Availability

Data sharing not applicable.

## Supplementary Materials

The following supporting information can be downloaded at: https://www.mdpi.com/article/10.3390/jof8080803/s1, Figure S1: Distribution of the published papers per molecular marker; Table S1: Molecular markers in fungi: an overview of manuscripts published in the last ten years using RFLP and similar techniques.; Table S2: Molecular markers in fungi: an overview of manuscripts published in the last 10 years using RAPD and similar techniques.; Table S3: Molecular markers in fungi: an overview of manuscripts published in the last 10 years using AFLP and similar techniques; Table S4: Molecular markers in fungi: an overview of manuscripts published in the last 10 years using ISSR and similar techniques; Table S5: Molecular markers in fungi: an overview of manuscripts published in the last 10 years using VNTR and similar techniques; Table S6: Molecular markers in fungi: an overview of manuscripts published in the last 10 years using SNP; Table S7: Molecular markers in fungi: an overview of manuscripts published in the last 10 years using InDels; Table S8: Molecular markers in fungi: an overview of manuscripts published in the last 10 years using DNA Barcoding; Table S9: Molecular markers in fungi: an overview of manuscripts published in the last 10 years using Metagenomics; Table S10: Molecular markers in fungi: an overview of manuscripts published in the last 10 years using whole genome sequencing.
