# Supplementary material for: Molecular Markers: An Overview of Data Published for Fungi over the Last Ten Years
Source: J Fungi (Basel). 2022 Jul 29;8(8):803. doi: 10.3390/jof8080803 (PMC9410331; doi:10.3390/jof8080803)
Supplement: Supplementary file 1 [file jof-08-00803-s001.zip › jof-1820928-supplementary.pdf]

References: Supplementary Table S1

1. Korres, G.; Meletiadis, J.; Delides, A.; Antoniadou, A.; Petrikkos, G.; Zerva, L.; Yiotakis, I.; Siopi, M.; Kalogeropoulou, E.; Georgantis, I.; Nikolopoulos, T. P. Fungal malignant otitis externa caused by *Alternaria chlamydospora*: first case report. *JMM Case Rep.*, **2015**, *2*, e000021. https://doi.org/10.1099/jmmcr.0.000021
2. Nasri, T.; Hedayati, M. T.; Abastabar, M.; Pasqualotto, A. C.; Armaki, M. T.; Hoseinnejad, A.; Nabili, M.. PCR-RFLP on β-tubulin gene for rapid identification of the most clinically important species of *Aspergillus*. *J. Microbiol. Methods*, **2015**, *117*, 144-147. https://doi.org/10.1016/j.mimet.2015.08.007
3. Diba, K.; Mirhendi, H.; Kordbacheh, P.; Rezaie, S. Development of RFLP-PCR method for the identification of medically important *Aspergillus* species using single restriction enzyme. *MoI. Braz. J. Microbiol.*, **2014**, *45*, 503-507. https://doi.org/10.1590/S1517-83822014000200018
4. Diba, K.; Jangi, F.; Makhdoomi, K.; Moshiri, N.; Mansouri, F. *Aspergillus* diversity in the environments of nosocomial infection cases at a university hospital. *J. Med. Life*, **2019**, *12*, 128. https://doi.org/10.25122/jml-2018-0057
5. Mohammadi, A.; Hashemi, S. M.; Abtahi, S. H.; Lajevardi, S. M.; Kianipour, S.; Mohammadi, R. An investigation on non-invasive fungal sinusitis; Molecular identification of etiologic agents. *J. Res. Med. Sci.*, **2017**, *22*, 67. https://doi.org/10.4103/jrms.JRMS_166_17
6. Ayatollah, M. S. A.; Salari, S.; Rezaei, S.; Shahabi, N. N.; Hadizadeh, S.; Kamyabi, H.; Aghasi, H. Identification of *Candida* species isolated from oral colonization in Iranian HIV-positive patients, by PCR-RFLP method. *Jundishapur J. Microbiol.*, **2012**, *5*, 336-340. https://doi.org/10.5812/kowsar.20083645.2429
7. Vijayakumar, R.; Giri, S.; Kindo, A. J. Molecular species identification of *Candida* from blood samples of intensive care unit patients by polymerase chain reaction–restricted fragment length polymorphism. *J. Lab. Physicians*, **2012**, *4(1)* . https://doi.org/001-004 10.4103/0974-2727.98661
8. Fallahi, A. A.; Korbacheh, P.; Zaini, F.; Mirhendi, H.; Zeraati, H.; Noorbakhsh, F.; Moazeni, M.; Andonian, L.; Nazeri, M; Rezaie, S. *Candida* species in cutaneous candidiasis patients in the Guilan province in Iran; identified by PCR-RFLP method. *Acta Med. Iran*, **2013**, *51(11)*, 799-804. PMID: 24390951
9. Noha, E. M.; Raafat, D.; Elewa, A.; Othman, W. Polymerase chain reaction-restriction fragment length polymorphism for characterization of Candida species causing onychomycosis. *Afr. J. Microbiol. Res.*, **2013**, *7(21)*, 2519-2523. https://doi.org/10.1093/jaoac/78.6.1542
10. Mohammadi, R.; Badiee, P.; Badali, H.; Abastabar, M.; Safa, A. H.; Hadipour, M.; Yazdani, H.; Heshmat, F. Use of restriction fragment length polymorphism to identify Candida species, related to onychomycosis. *Adv. Biomed. Res.*, **2015**, *4*, 95 https://doi.org/10.4103/2277-9175.156659
11. Gharaghani, M.; Ahmadi, B.; Taheripour Sisakht, M.; Ilami, O.; Aramesh, S.; Mouhamadi, F.; Barati, Z.; Roozmeh, S.; Mohammadi, H.; Nouripour-Sisakht, S. Identification of *Candida* species isolated from vulvovaginal candidiasis patients by polymerase chain reaction-restriction fragment length polymorphism (PCR-RFLP) in Yasuj southwestern Iran. *Jundishapur J. Microbiol.*, **2018**, *11*, e65359. https://doi.org/10.5812/jjm.65359
12. Bayraktar, S.; Duran, N.; Duran, G. G.; Eryilmaz, N.; Aslan, H.; Önlen, C.; Özer, B. Identification of medically important Candida species by polymerase chain reaction-restriction fragment length polymorphism analysis of the rDNA ITS1 and ITS2 regions. *Indian J. Med. Microbiol.*, 2017, 35, 535-542. https://doi.org/10.4103/ijmm.IJMM_17_102
13. Fatima, A.; Bashir, G.; Wani, T.; Jan, A.; Kohli, A.; Khan, M. S. Molecular identification of *Candida* species isolated from cases of neonatal candidemia using polymerase chain reaction-restriction fragment length polymorphism in a tertiary care hospital. Indian J. Pathol. Microbiol., **2017**, *60*, 61.
14. Abharian, P. H.; Dehghan, P.; Hassani-Abharian, P.; Jabalameli, Z. Frequency of *Candida* species in the oral cavity of narcotics and stimulants smokers in Isfahan, using polymerase chain reaction-restriction fragment length polymorphism method. *Adv. Biomed. Res.*, 2020, **9**, 30 https://doi.org/10.4103/abr.abr_38_20
15. Ge, Y. P.; Wang, L.; Lu, G. X.; Shen, Y. N.; Liu, W. D. A simple and reliable PCR-restriction fragment length polymorphism assay to identify Candida albicans and its closely related *Candida dubliniensis*. *Braz. J. Microbiol.*, **2012**, *43*, 873-879. https://doi.org/10.1590/S1517-83822012000300004
16. Barbedo, L. S.; Figueiredo-Carvalho, M. H. G.; Muniz, M. D. M.; Zancopé-Oliveira, R. M. The identification and differentiation of the *Candida parapsilosis* complex species by polymerase chain reaction-restriction fragment length polymorphism of the internal transcribed spacer region of the rDNA. *Mem. Inst. Oswaldo Cruz.*, **2016**, *111*, 267-270. https://doi.org/10.1590/0074-02760150466
17. Sousa, D. R. T. D.; Santos, C. S. D. S.; Wanke, B.; Silva Júnior, R. M. D.; Santos, M. C. D.; Cruz, K. S.; Monte, R. L.; Nocker, A.; Souza, J. V. B. D.. PCR-RFLP as a useful tool for diagnosis of invasive mycoses in a healthcare facility in the North of Brazil. Electron. *J. Biotechnol.*, **2015**, *18*, 231-235. https://doi.org/10.1016/j.ejbt.2015.03.012
18. Florek, M.; Król, J.; Woźniak-Biel, A. Atypical URA5 gene restriction fragment length polymorphism banding profile in Cryptococcus neoformans strains. *Folia Microbiol.*, **2019**, *64*, 857-860. https://doi.org/10.1007/s12223-019-00699-y
19. Jha, B. K.; Murthy, S. M.; Devi, N. L. Molecular identification of dermatophytosis by polymerase chain reaction (PCR) and detection of source of infection by restricted fragment length polymorphism (RFLP). *JCMS-Nepal*, **2012**, *8*, 20127-15
20. Takeda, K.; Nishibu, A.; Anzawa, K.; Mochizuki, T. Molecular epidemiology of a major subgroup of *Arthroderma benhamiae* isolated in Japan by restriction fragment length polymorphism analysis of the non-transcribed spacer region of ribosomal RNA gene. *Jpn. J. Infect. Dis.*, **2012**, *65*, 233-239. https://doi.org/10.7883/yoken.65.233

21. Elavarashi, E.; Kindo, A. J.; Kalyani, J. Optimization of PCR—RFLP directly from the skin and nails in cases of dermatophytosis, targeting the ITS and the 18S ribosomal DNA regions. *J. Clin. Diagnostic Res.*, **2013**, *7*, 646. https://doi.org/10.7860/JCDR/2013/5363.2873
22. Jung, H. J.; Kim, S. Y.; Jung, J. W.; Park, H. J.; Lee, Y. W.; Choe, Y. B.; Ahn, K. J. Identification of dermatophytes by polymerase chain reaction-restriction fragment length polymorphism analysis of metalloproteinase-1. *Ann. Dermatol.*, **2014**, *26*, 338-342. https://doi.org/10.5021/ad.2014.26.3.338
23. Mohammadi, R.; Abastabar, M.; Mirhendi, H.: Badali, H.; Shadzi, S.; Chadeganipour, M.; Pourfathi, P.; Jalalizand, N.; Haghani, I. Use of restriction fragment length polymorphism to rapidly identify dermatophyte species related to dermatophytosis. Jundishapur J. Microbiol., 8, 2015, e17296
24. Kaul, S.; Kour, H.; Pandita, D.; Kour, S.; Dhar, M. K. Polymerase chain reaction: restriction fragment length polymorphism differentiates the environmental and clinically important fungal isolates. *Natl. Acad. Sci. Lett.*, **2012**, *36*, 139-146. https://doi.org/10.1007/s40009-013-0121-7
25. Zarrin, M.; Ganj, F.; Faramarzi, S. Analysis of the rDNA internal transcribed spacer region of the *Fusarium* species by polymerase chain reaction-restriction fragment length polymorphism. *Biomed. Rep.*, **2016**, *4*, 471-474. https://doi.org/10.3892/br.2016.615
26. Taha, M.; Elfangary, M.; Essa, S.; Younes, A. Species identification of dermatophytes isolated from human superficial fungal infections by conventional and molecular methods. *J. Egypt. Women's Dermatologic Soc.*, **2017**, *14*, 76-84. https://doi.org/10.1097/01.EWX.0000499598.84966.cb
27. Al-Khafajii, K.; Hasan, K. M.; Mohsin, R. D. A. RFLP-PCR for identification of dermatophytes and *Candida* species from clinical isolates of hair and skin. *Ann. Trop. Med. Public Health*, **2019**, *22*, 73-80. https://doi.org/10.36295/ASRO.2019.220411
28. Lubis, N. Z.; Muis, K.; Nasution, L. H. Polymerase chain reaction-restriction fragment length polymorphism as a confirmatory test for onychomycosis. *Open Access Maced. J. Med. Sci.*, **2018**, *6*, 280. https://doi.org/10.3889/oamjms.2018.098
29. Haghani, I.; Shams-Ghahfarokhi, M.; Dalimi Asl, A.; Shokohi, T.; Hedayati, M. T. Molecular identification and antifungal susceptibility of clinical fungal isolates from onychomycosis (uncommon and emerging species). *Mycoses*, **2019**, *62*, 128-143. https://doi.org/10.1111/myc.12854
30. Wumba, R.; Jean, M.; Benjamin, L. M.; Madone, M.; Fabien, K.; Josué, Z; ; Marc, T. *Enterocytozoon bieneusi* identification using real-time polymerase chain reaction and restriction fragment length polymorphism in HIV-infected humans from Kinshasa province of the Democratic Republic of Congo. *J. Parasitol. Res.*, **2012**, *2012*, 278028. https://doi.org/10.1155/2012/278028
31. Zaman, K.; Rudramurthy, S. M.; Das, A.; Panda, N.; Honnavar, P.; Kaur, H.; Chakrabarti, A. Molecular diagnosis of rhino-orbito-cerebral mucormycosis from fresh tissue samples. *J. Med. Microbiol.*, **2017**, *66*, 1124-1129. https://doi.org/10.1099/jmm.0.000560
32. Mogoye, B. K.; Du Plessis, D.; Poonsamy, B.; Frean, J. Characterisation of *Pneumocystis jirovecii* DHPS genotypes using a simple, inexpensive restriction fragment length polymorphism analysis. *S. Afr. J. Infect. Dis.*, **2015**, *30*, 46-50. https://doi.org/10.1080/23120053.2015.1054180
33. Roberto, T. N.; Rodrigues, A. M.; Hahn, R. C.; de Camargo, Z. P. Identifying *Paracoccidioides* phylogenetic species by PCR-RFLP of the alpha-tubulin gene. *Sabouraudia*, **2015**, *54*, 240-247. https://doi.org/10.1093/mmy/myv083
34. Cui, G.; Zhu, R.; Zuo, X.; Zhong, S.; Yang, S.; Liang, M.; Sun, J.; Liu, J. Identification and phyletic evolution analysis of Proteus mirabilis strains by PCR and restriction fragment length polymorphism of 16S-23S rRNA gene intergenic spacer region. *Chin. J. Prev. Vet. Med.*, **2013**, *33*, 367-399. https://www.cabdirect.org/cabdirect/abstract/20133225705
35. Worasilchai, N.; Chaumpluk, P.; Chakrabarti, A.; Chindamporn, A. Differential diagnosis for pythiosis using thermophilic helicase DNA amplification and restriction fragment length polymorphism (tHDA-RFLP). Med. Mycol., 56, 2018, 216-224. https://doi.org/10.1093/mmy/myx033
36. Kordalewska, M.; Kalita, J.; Bakuła, Z.; Brillowska-Dąbrowska, A.; Jagielski, T. PCR-RFLP assays for species-specific identification of fungi belonging to *Scopulariopsis* and related genera. *Med Mycol.*, **2019**, *57*, 643-648. https://doi.org/10.1093/mmy/myy106
37. Szekely, J.; Chelae, S.; Ingviya, N.; Rukchang, W.; Auepemkiate, S.; Aiempanakit, K. Universal multiplex polymerase chain reaction-restriction fragment length polymorphism (UMPCR-RFLP) for rapid detection and species identification of fungal and mycobacterial pathogens. *Walailak J. Sci. & Tech.*, **2020**, *17*, 1113-1125. https://doi.org/10.48048/wjst.2020.10713
38. Verrier, J.; Pronina, M.; Peter, C.; Bontems, O.; Fratti, M.; Salamin, K.; Schürch, S.; Gindro, K.; Wolfender, J. L.; Harshman, K.; Monod, M. Identification of infectious agents in onychomycoses by PCR-terminal restriction fragment length polymorphism. *J. Clin. Microbiol.*, **2012**, *50*, 553-561. https://doi.org/10.1128/JCM.05164-11
39. Ziółkowska, G.; Nowakiewicz, A.; Gnat, S.; Trościańczyk, A.; Zięba, P.; Majer Dziedzic, B. Molecular identification and classification of *Trichophyton mentagrophytes* complex strains isolated from humans and selected animal species. *Mycoses*, **2015**, *58*, 119-126. https://doi.org/10.1111/myc.12284
40. Sun, P. L.; Lin, H. C.; Hsiao, P. F.; Lu, J. J. Application of restriction fragment length polymorphism (RFLP) and high resolution melting (HRM) analysis of beta-tubulin gene for species and strains differentiation in *Trichophyton mentagrophytes* species complex. *J. Dermatol. Sci.*, **2016**, *83*, 247-250. https://doi.org/10.1016/j.jdermsci.2016.05.013
41. Kawasaki, M.; Anzawa, K.; Mochizuki, T.; Ishizaki, H. New strain typing method with *Sporothrix schenckii* using mitochondrial DNA and polymerase chain reaction restriction fragment length polymorphism (PCR–RFLP) technique. *J. Dermatol.*, **2012**, *39*, 362-365. https://doi.org/10.1111/j.1346-8138.2011.01379.x
42. Rodrigues, A. M.; de Hoog, G. S.; de Camargo, Z. P.. Genotyping species of the *Sporothrix schenckii* complex by PCR-RFLP of calmodulin. *Diagn. Microbiol. Infect. Dis.*, **2014**, *78*, 383-387. https://doi.org/10.1016/j.diagmicrobio.2014.01.004

43. Verrier, J.; Jeanneau-Imparato, L.; Guillet, C.; Bourdeau, P.; Bouchara, J. P.. PCR-terminal restriction fragment length polymorphism for direct detection and identification of dermatophytes in veterinary mycology. *Med. Mycol.*, **2019**, *57*, 447-456. https://doi.org/10.1093/mmy/myy058
44. Ai, S; Zhong, Z; Peng, G; Wang, C; Luo, Y; He, T; Gu, W; Li, C; Li, G; Wu, H; Liu, X; Xia, Y; Liu, Y; Zhou, X. Intestinal fungal diversity of sub-adult giant panda. *Acta Microbiol. Sin.*, **2014**, *54*, 1344-1352. PMID: 25752141
45. Taha, M.; Tartor, Y. H.; El-Hamid, A.; El-Mesalamy, M. M.; EL Sayed, A. PCR-restriction fragment length polymorphism and DNA sequencing for identification of *Malassezia* species isolated from animals in Egypt. *Zagazig Vet. J.*, **2020**, *48*, 128-139. https://doi.org/10.21608/zvjz.2019.18116.1086
46. Fadda, M. E.; Pisano, M. B.; Scaccabarozzi, L.; Mossa, V.; Deplano, M.; Moroni, P.; Liciardi, M..; Cosentino, S. Use of PCR-restriction fragment length polymorphism analysis for identification of yeast species isolated from bovine intramammary infection. *Int. J. Dairy Sci.*, **2013**, *96*, 7692-7697. https://doi.org/10.3168/jds.2013-6996
47. Abass, M. H. A PCR ITS-RFLP method for identifying fungal contamination of date palm (*Phoenix dactylifera* L.) tissue cultures. *Afr. J. Biotechnol.*, **2013**, *12*, 5054-5059. https://doi.org/10.5897/AJB2013.12407
48. Behie, S. W.; Jones, S. J.; Bidochka, M. J. Plant tissue localization of the endophytic insect pathogenic fungi *Metarhizium* and *Beauveria*. *Fungal Ecol.*, **2015**, *13*, 112-119. https://doi.org/10.1016/j.funeco.2014.08.001
49. Maharaj, A.; Rampersad, S. N. Genetic differentiation of *Colletotrichum gloeosporioides* and *C. truncatum* associated with anthracnose disease of papaya (*Carica papaya* L.) and bell pepper (*Capsium annuum* L.) based on ITS PCR-RFLP fingerprinting. *Mol. Biotechnol.*, **2012**, *50*, 237-249. https://doi.org/10.1007/s12033-011-9434-2
50. Ramdeen, S.; Rampersad, S. N. Intraspecific differentiation of *Colletotrichum gloeosporioides sensu lato* based on in silico multilocus PCR-RFLP fingerprinting. *Mol. Biotechnol.*, **2013**, *53*, 170-181. https://doi.org/10.1007/s12033-012-9509-8
51. Gumiere, T.; Durrer, A.; Bohannan, B. J.; Andreote, F. D. Biogeographical patterns in fungal communities from soils cultivated with sugarcane. *J. Biogeogr.*, **2016**, *43*, 2016-2026. https://doi.org/10.1007/s12033-012-9509-8
52. Srivastava, S.; Gupta, P. S.; Lal, S.; Sinha, O. K. Rapid identification of endophytic fungi of sugarcan (saccharum spp. hybrid) using PCR-RFLP of rDNA. *J. Environ. Biol.*, **2017**, *38*, 21.
53. Shubin, L.; Juan, H.; RenChao, Z.; ShiRu, X.; YuanXiao, J. Fungal endophytes of *Alpinia officinarum* rhizomes: insights on diversity and variation across growth years, growth sites, and the inner active chemical concentration. *PloS One*, **2014**, *9(12)*, e115289. https://doi.org/10.1371/journal.pone.0115289
54. Judova, J.; Dubikova, K.; Gaperova, S.; Gaper, J., Pristas, P. The occurrence and rapid discrimination of *Fomes fomentarius* genotypes by ITS-RFLP analysis. *Fungal Biol.*, **2012**, *116*, 155-160. https://doi.org/10.1016/j.funbio.2011.10.010
55. Kambouris, M. E.; Manoussopoulos, Y.; Kritikou, S.; Milioni, A.; Mantzoukas, S.; Velegraki, A. Toward decentralized agrigenomic surveillance? A polymerase chain reaction–restriction fragment length polymorphism approach for adaptable and rapid detection of user-defined fungal pathogens in potato crops. *OMICS J. Integr. Biol.*, **2018**, *22(4)*, 264-273. https://doi.org/10.1089/omi.2018.0012
56. Penuelas, J.; Rico, L.; Ogaya, R.; Jump, A. S.; Terradas, J. J. P. B. Summer season and long-term drought increase the richness of bacteria and fungi in the foliar phyllosphere of *Quercus ilex* in a mixed Mediterranean forest. *Plant Biol.*, **2012**, *14*, 565-575. https://doi.org/10.1111/j.1438-8677.2011.00532.x
57. Kim, J. S.; Kang, N. J.; Kwak, Y. S.; Lee, C. Investigation of genetic diversity of *Fusarium oxysporum f. sp. fragariae* using PCR-RFLP. *Plant Pathol. J.*, **2017**, *33*, 140. https://doi.org/10.5423/PPJ.FT.01.2017.0011
58. Quecine, M. C.; Bini, A. P.; Romagnoli, E. R.; Andreote, F. D.; Moon, D. H.; Labate, C. A. Genetic variability in *Puccinia psidii* populations as revealed by PCR-DGGE and T-RFLP markers. *Plant Dis.*, **2014**, *98*, 16-23. https://doi.org/10.1094/PDIS-03-13-0332-RE
59. Koffi, Y. F.; Diguta, C. ; Alloue-Boraud, M. ; Koffi, L. B.; Dje, M.; Gherghina, E. ; Matei, F. PCR-ITS-RFLP identification of pineapple spoilage fungi. *Rom. Biotechnol. Lett.*, **2019**, *24*, 418-424. https://doi.org/10.25083/rbl/24.3/418.424
60. Sun, Y.; Liu, Y. Investigating of yeast species in wine fermentation using terminal restriction fragment length polymorphism method. *Food Microbiol.*, **2014**, *38*, 201-207. https://doi.org/10.1016/j.fm.2013.09.001
61. Zhang, W.; Zhang, X.; Li, M.; Shi, Y.; Zhang, P.; Cheng, X. L.; Wei, F.; Ma, S. Identification of Chinese caterpillar medicinal mushroom, *Ophiocordyceps sinensis* (Ascomycetes) from counterfeit species. *Int. J. Med. Mushrooms*, **2017**, *19*, 1061-1070. https://doi.org/10.1615/IntJMedMushrooms.2017024823
62. Rosenzweig, N.; Hanson, L. E.; Clark, G.; Franc, G. D.; Stump, W. L.; Jiang, Q. W.; Stewart, J.; Kirk, W. W. Use of PCR-RFLP analysis to monitor fungicide resistance in *Cercospora beticola* populations from sugarbeet (*Beta vulgaris*) in Michigan, United States. *Plant Dis.*, **2015**, *99*, 355-362. https://doi.org/10.1094/PDIS-03-14-0241-RE
63. Mallick, P.; Sikdar, S. R.. Restriction fragment length polymorphism and sequence analysis of rRNA-ITS region of somatic hybrids produced between *Pleurotus florida* and *Lentinula edodes*. *Ann. Microbiol.*, **2016**, *66*, 389-395. https://doi.org/10.1007/s13213-015-1121-2
64. Zarrin, M.; Erfaninejad, M. Molecular variation analysis of *Aspergillus flavus* using polymerase chain reaction-restriction fragment length polymorphism of the internal transcribed spacer rDNA region. *Exp. Ther. Med.*, **2016**, *12*, 1628-1632. https://doi.org/10.3892/etm.2016.3479
65. Lu, Y.; Tong, Z.; Zhang, X.; Chou, T.; Xu, W.; Chen, B.; Xie, B. Rapid and effective identification of A mating-type loci in *Volvariella volvacea* by polymerase chain reaction-restriction fragment length polymorphism. *Sci. Hortic.*, **2019**, *250*, 266-270. https://doi.org/10.1016/j.scienta.2019.02.063

66. Andronov, E. E.; Petrova, S. N.; Pinaev, A. G.; Pershina, E. V.; Rakhimgalieva, S. Z.; Akhmedenov, K. M., Gorobets, A. V. ;Sergaliev, N. K.. Analysis of the structure of microbial community in soils with different degrees of salinization using T-RFLP and real-time PCR techniques. *Eurasian Soil Sci.*, **2012**, *45*, 147-156. https://doi.org/10.1134/S1064229312020044
67. Bainard, L. D.; Koch, A. M.; Gordon, A. M.; Klironomos, J. N. Temporal and compositional differences of arbuscular mycorrhizal fungal communities in conventional monocropping and tree-based intercropping systems. *Soil Biol. Biochem.*, **2012**, *45*, 172-180. https://doi.org/10.1016/j.soilbio.2011.10.008
68. Feng, Y.; Tang, M.; Chen, H.; Cong, W. Community diversity of bacteria and arbuscular mycorrhizal fungi in the rhizosphere of eight plants in Liudaogou watershed on the Loess Plateau China. *Huanjing Kexue*, **2012**, *33*, 314-322. PMID: 22452228
69. Verbruggen, E.; Kuramae, E. E.; Hillekens, R.; de Hollander, M., Kiers; E. T., Röling; W. F.; ; van der Heijden, M. G. Testing potential effects of maize expressing the *Bacillus thuringiensis* Cry1Ab endotoxin (Bt maize) on mycorrhizal fungal communities via DNA-and RNA-based pyrosequencing and molecular fingerprinting. *Appl. Environ. Microbiol.*, **2012**, *78*, 7384-7392. https://doi.org/10.1128/AEM.01372-12
70. Hazard, C.; Gosling, P.; Van Der Gast, C. J.; Mitchell, D. T.; Doohan, F. M.; Bending, G. D. The role of local environment and geographical distance in determining community composition of arbuscular mycorrhizal fungi at the landscape scale. *ISME J.*, **2013**, *7*, 498-508. https://doi.org/10.1038/ismej.2012.127
71. Hazard, C.; Gosling, P.; Mitchell, D. T.; Doohan, F. M.; Bending, G. D. Diversity of fungi associated with hair roots of ericaceous plants is affected by land use. *FEMS Microbiol. Ecol.*, **2014**, *87*, 586-600. https://doi.org/10.1111/1574-6941.12247
72. Yuan, H.; Ge, T.; Zhou, P.; Liu, S.; Roberts, P.; Zhu, H.; Zou, Z.; Tong, C.; Wu, J. Soil microbial biomass and bacterial and fungal community structures responses to long-term fertilization in paddy soils. *J. Soils Sediments*, **2013**, *13*, 877-886. https://doi.org/10.1007/s11368-013-0664-8
73. Liu, W.; Zhang, Y.; Jiang, S.; Deng, Y.; Christie, P.; Murray, P. J.; Li, X.; Zhang, J. Arbuscular mycorrhizal fungi in soil and roots respond differently to phosphorus inputs in an intensively managed calcareous agricultural soil. *Sci. Rep.*, **2016**, *6*, 1-11. https://doi.org/10.1038/srep24902
74. Liu, W.; Zhang, Y.; Jiang, S.; Murray, P. J.; Liao, L.; Li, X.; Zhang, J. Spatiotemporal differences in the arbuscular mycorrhizal fungi communities in soil and roots in response to long-term organic compost inputs in an intensive agricultural cropping system on the North China Plain. *J. Soils Sediments*, **2019**, *19*, 2520-2533. https://doi.org/10.1007/s11368-019-02244-3
75. Anderson, I. C.; Genney, D. R.; Alexander, I. J. Fine-scale diversity and distribution of ectomycorrhizal fungal mycelium in a Scots pine forest. *New Phytol.*, **2014**, *201*, 1423-1430. https://doi.org/10.1111/nph.12637
76. Montes-Borrego, M.; Metsis, M.; Landa, B. B. Arbuscular mycorhizal fungi associated with the olive crop across the Andalusian landscape: factors driving community differentiation. *PLoS One*, **2014**, *9*, e96397. https://doi.org/10.1371/journal.pone.0096397
77. Karamanlioglu, M.; Houlden, A.; Robson, G. D. Isolation and characterisation of fungal communities associated with degradation and growth on the surface of poly (lactic) acid (PLA) in soil and compost. *Int. Biodeterior. Biodegradation*, **2014**, *95*, 301-310. https://doi.org/10.1016/j.ibiod.2014.09.006
78. Kil, Y. J.; Eo, J. K.; Lee, E. H.; Eom, A. H. Root age-dependent changes in arbuscular mycorrhizal fungal communities colonizing roots of *Panax ginseng*. *Mycobiol.*, **2014**, *42*, 416-421. https://doi.org/10.5941/MYCO.2014.42.4.416
79. Wang, N. N.; Chen, Y.; Ying, J. Y.; Gao, Y. S.; Bai, Y. F. Effects of typical plant on soil microbial communities in an Inner Mongolia grassland. *Chin. J. Plant Ecol.*, **2014**, *38*, 201
80. Liang, Y.; Pan, F.; He, X.; Chen, X.; Su, Y. Effect of vegetation types on soil arbuscular mycorrhizal fungi and nitrogen-fixing bacterial communities in a karst region. *Environ. Sci. Pollut. Res.*, **2016**, *23*, 18482-18491. https://doi.org/10.1007/s11356-016-7022-5
81. Wetzel, K.; Silva, G.; Matczinski, U.; Oehl, F.; Fester, T. Superior differentiation of arbuscular mycorrhizal fungal communities from till and no-till plots by morphological spore identification when compared to T-RFLP. *Soil Biol. Biochem.*, **2014**, *72*, 88-96. https://doi.org/10.1016/j.soilbio.2014.01.033
82. Martínez-García, L. B.; Richardson, S. J.; Tylianakis, J. M.; Peltzer, D. A.; Dickie, I. A. Host identity is a dominant driver of mycorrhizal fungal community composition during ecosystem development. *New Phytol.*, **2015**, *205(4)*, 1565-1576. https://doi.org/10.1111/nph.13226
83. Haňáčková, Z.; Koukol, O.; Štursová, M.; Kolařík, M.; Baldrian, P. Fungal succession in the needle litter of a montane *Picea abies* forest investigated through strain isolation and molecular fingerprinting. *Fungal Ecol.*, **2016**, *13*, 157-166. https://doi.org/10.1016/j.funeco.2014.09.007
84. Turrini, A.; Sbrana, C.; Giovannetti, M. Belowground environmental effects of transgenic crops: a soil microbial perspective. *Res. Microbiol.*, **2015**, *166*, 121-131. https://doi.org/10.1016/j.resmic.2015.02.006
85. Ge, Y.; Priester, J. H.; Mortimer, M.; Chang, C. H.; Ji, Z.; Schimel, J. P.; Holden, P. A. Long-term effects of multiwalled carbon nanotubes and graphene on microbial communities in dry soil. *Environm. Sci. Technol.*, **2016**, *50*, 3965-3974. https://doi.org/10.1021/acs.est.5b05620
86. Grudzinska-Sterno, M.; Yuen, J.; Stenlid, J.; Djurle, A. Fungal communities in organically grown winter wheat affected by plant organ and development stage. *Eur. J. Plant Pathol.*, **2016**, *146*, 401-417. https://doi.org/10.1007/s10658-016-0927-5
87. Ruankaew Disyatat, N.; Yomyart, S.; Sihanonth, P.; Piapukiew, J. Community structure and dynamics of ectomycorrhizal fungi in a dipterocarp forest fragment and plantation in Thailand. *Plant Ecol. Divers.*, **2016**, *9*, 577-588. https://doi.org/10.1080/17550874.2016.1264018
88. Varenius, K.; Kårén, O.; Lindahl, B.; Dahlberg, A. Long-term effects of tree harvesting on ectomycorrhizal fungal communities in boreal Scots pine forests. *For. Ecol. Manag.*, **2016**, *380*, 41-49. https://doi.org/10.1016/j.foreco.2016.08.006

89. Gaggini, L.; Rusterholz, H. P.; Baur, B. The invasive plant *Impatiens glandulifera* affects soil fungal diversity and the bacterial community in forests. *App. Soil Ecol.*, **2018**, *124*, 335-343. https://doi.org/10.1016/j.apsoil.2017.11.021
90. Chen, J.; Liu, X.; Zheng, J.; Zhang, B.; Lu, H.; Chi, Z.; Pan, G.; Li, L.; Zheng, J.; Zhang, X.; Wang, J.; Yu, X. Biochar soil amendment increased bacterial but decreased fungal gene abundance with shifts in community structure in a slightly acid rice paddy from Southwest China. *Appl. Soil Ecol.*, **2013**, *71*, 33-44. https://doi.org/10.1016/j.apsoil.2013.05.003
91. Wang, Y.: Ma, Z.; Wang, X.; Sun, Q.; Dong, H.; Wang, G.; Chen, X.; Yin, C; Han, Z.; Mao, Z. Effects of biochar on the growth of apple seedlings, soil enzyme activities and fungal communities in replant disease soil. *Sci. Hortic.*, **2019**, *256*, 108641. https://doi.org/10.1016/j.scienta.2019.108641
92. Siczek, A.; Frąc, M.; Gryta, A.; Kalembasa, S.; Kalembasa, D. Variation in soil microbial population and enzyme activities under faba bean as affected by pentachlorophenol. *App. Soil Ecol.*, **2020**, *150*, 103466. https://doi.org/10.1016/j.apsoil.2019.103466
93. Yao, Z.; Li, Y.; Li, Y.; Chen, Z. Effects of different tea plantation ages on soil microbial community structure and diversity. *J. App. Ecol.*, **2020**, *31(8)*, 2749-2758. https://doi.org/10.13287/j.1001-9332.202008.037
94. Allsup, C. M.; Lankau, R. A.; Paige, K. N. Herbivory and soil water availability induce changes in arbuscular mycorrhizal fungal abundance and composition. *Microb. Ecol.*, 2021. https://doi.org/10.1007/s00248-021-01835-3
95. Salamone, A. L.; Robicheau, B. M.; Walker, A. K. Fungal diversity of marine biofilms on artificial reefs in the north-central Gulf of Mexico. *Botanica Marina*, **2016**, *59*, 291-305. https://doi.org/10.1515/bot-2016-0032
96. Zumsteg, A.; Luster, J.; Göransson, H.; Smittenberg, R. H.; Brunner, I.; Bernasconi, S. M.; Zeyer, J.; Frey, B. Bacterial, archaeal and fungal succession in the forefield of a receding glacier. *Microb. Ecol.*, **2012**, *63*, 552-564. https://doi.org/10.1007/s00248-011-9991-8
97. Edwards, A.; Douglas, B.; Anesio, A. M.; Rassner, S. M.; Irvine-Fynn, T. D.; Sattler, B.; Griffith, G. W. A distinctive fungal community inhabiting cryoconite holes on glaciers in Svalbard. *Fungal Ecol.*, **2013**, *6*, 168-176. https://doi.org/10.1016/j.funeco.2012.11.001
98. Zumsteg, A.; Bååth, E.; Stierli, B.; Zeyer, J.; Frey, B. Bacterial and fungal community responses to reciprocal soil transfer along a temperature and soil moisture gradient in a glacier forefield. *Soil Biol. Biochem.*, **2013**, *61*, 121-132. https://doi.org/10.1016/j.soilbio.2013.02.017
99. Hu, W.; Zhang, Q.; Li, D.; Cheng, G.; Mu, J.; Wu, Q.; Niu, F; An, L.; Feng, H. Diversity and community structure of fungi through a permafrost core profile from the Qinghai-Tibet Plateau of China. *J. Basic Microbiol.*, **2014**, *54*, 1331-1341. https://doi.org/10.1002/jobm.201400232
100. Ortiz-Vera, M. P.; Olchanheski, L. R.; da Silva, E. G.; de Lima, F. R.; Martinez, L. R. D. P. R.; Sato, M. I. Z.; Jaffé, R.; Alves, R.; Ichiwaki, S.; Padilla, G.; Araújo, W. L. Influence of water quality on diversity and composition of fungal communities in a tropical river. *Sci. Rep.*, **2018**, *8*, 1-9. https://doi.org/10.1038/s41598-018-33162-y
101. Pietryczuk, A.; Cudowski, A.; Hauschild, T.; Świsłocka, M.; Więcko, A.; Karpowicz, M. Abundance and species diversity of fungi in rivers with various contaminations. *Curr. Microbiol.*, **2018**, *75*, 630-638. https://doi.org/10.1007/s00284-017-1427-3
103. Iordache, O.; Cornea, C. P.; Diguta, C.; Rodino, S.; Ionescu, I.; Matei, A.; Dumitrescu, I. Molecular identification of fungal isolates from textile wastewater by ITS-RFLP method. Bulletin UASVM Animal Science and Biotechnologies, 72, 2, 2015. https://doi.org/10.15835/buasvmcn-asb:11608

References: Supplementary Table S2

1. Xu, H.; Liu, M.; Chen, Y.; Huang, J.; Xu, C.; Lu, L. Randomly amplified polymorphic deoxyribonucleic acid (DNA) analysis of Candida albicans isolates from clinical sources of hospital in south China. *Afr. J. Microbiol. Res.*, **2012**, *6(10)*, 2552-2558. https://doi.org/10.5897/AJMR12.111
2. Diba, K.; Makhdoomi, K.; Mirhendi, H. Molecular characterization of *Aspergillus* infections in an Iranian educational hospital using RAPD-PCR method. *Iran. J. Basic Med. Sci.*, **2014**, *17(9)*, 646
3. Diba, K., Makhdoomi, K., Rahimirad, M., Jabari, D. Survey of opportunistic fungi in the clinical and environmental specimens obtained from Urmia educational hospitals. Journal of North Khorasan University of Medical Sciences, 6(1), 2014, 51-58. PMID: 25691939
4. Paluchowska, P., Tokarczyk, M., Bogusz, B., Skiba, I., Budak, A. Molecular epidemiology of *Candida albicans* and *Candida glabrata* strains isolated from intensive care unit patients in Poland. *Mem. Inst. Oswaldo Cruz*, **2014**, *109*, 436-441. https://doi.org/10.1590/0074-0276140099
5. Kordalewska, M.; Jagielski, T.; Brillowska-Dąbrowska, A. Typing of *Scopulariopsis* and *Microascus* fungi by Random Amplified Polymorphic DNA (RAPD). *Mycoses*, **2015**, *58*, 185-185
6. Gamarra, S., Chaves, M. S., Cabeza, M. S., Macedo, D., Leonardelli, F., Franco, D., Boleas, M.; Garcia-Effron, G. Mucormycosis outbreak due to *Rhizopus microsporus* after arthroscopic anterior cruciate ligament reconstruction surgery evaluated by RAPD and MALDI-TOF Mass spectrometry. *J. Mycol. Med.*, **2018**, *28(4)*, 617-622. https://doi.org/10.1016/j.mycmed.2018.09.002
7. Abdullah, H. I., Hammadi, S. Y., Hussein, A. S., Dheeb, B. I. Investigation of genetic diversity and relationships among the clinical Candida species using random amplified polymorphic DNA (RAPD) analysis. *Res. J. Biotechnol.*, **2019**, *14(1)*, 6-13.
8. Amini, K. Evaluation of genomic polymorphism of *Candida albicans*, isolated from the patients with vaginitis by RAPD-PCR method. *Journal of Guilan University of Medical Sciences*, **2019**, *27*, 1-10.
9. Alshehri, B.; Manikandan, P.; Abdel-hadi, A.; Dukhyil, A. A. B.; Alaidarous, M.; Banawas, S.; Alharbi, R. A.; Aloyuni, S. A.; Vágvölgyi, C.; Kredics, L.; Kocsubé, S.; Al-Malki, E. S.; Vijayakumar, R.; Selvamh, K. P.; Shobana, C. S. Corneal ulcer/keratitis

derived *Aspergillus flavus* and *Aspergillus tamarii* and their RAPD-PCR typing. *J. King Saud Univ. Sci.*, **2020**, *32(3)*, 2103-2111. https://doi.org/10.1016/j.jksus.2020.02.019
10. Amini, K., Chehreii, S., & Malekabadi, P. Genomic polymorphism of *Trichophyton rubrum* isolated from keratinized clinical sources using the RAPD-PCR method. *JIUMS*, **2021**, *29*, 39-49
11. Freed, S., Feng-Liang, J., Shun-Xiang, R. Intraspecific variability among the Isolates of *Metarhizium anisopliae* var. *anisopliae* by RAPD Markers. *Int. J. Agric. Biol.*, **2014**, *16(5)*, 899-904.
12. Ghiasi, M.; Khosravi, A. L. I. R. E. Z. A.; Soltani, M.; Sharifpour, I.; Binaii, M.; Ebrahimzadeh Mosavi, H.; Bahonar, A. Evaluation of physiological aspects and molecular identification of *Saprolegnia* isolates from rainbow trout (*Oncorhynchus mykiss*) and Caspian trout (*Salmo trutta caspius*) eggs based on RAPD–PCR. *Iran. Fish. Sci. J.*, **2014**, *22*, 82-92.
13. Kasim, A. A.; Dragh, M. Identification of nematode trapping fungus *Monacrosporium eudermatum* based on genetic diversity using RAPD technique. *Am. J. Microbiol*. Res., **2016**, *4*, 178-180. https://doi.org/10.12691/ajmr-4-6-4
14. Kamarudin, N., Moslim, R., Ali, S. R. A., Pong, K. K., Azmi, W. A. Genetic variation of entomopathogenic fungi, *Metarhizium anisopliae* and *Isaria amoenerosea* and their pathogenicity against subterranean termite, *Coptotermes curvignathus*. *J. Oil Palm Res.*, **2017**, *29(1)*, 35-46. https://doi.org/10.21894/jopr.2017.2901.04
15. Arabi, M. I. E.; Nabulsi, I.; MirAli, N. Molecular phylogeny of *Pyrenophora graminea* as determined by RAPD and ISSR fingerprints. *J. Plant Biol. Res.*, **2012**, *1(2)*, 25-35
16. Bahrabadi, M.; Karimi, S. M.; Hashemi, M. Study on genetic variability in fungi associated with esca disease in North Khorasan province vineyards with RAPD-PCR. J. Plant Prot. Res., 26, 2012, 92-100
17. Baturo-Ciesniewska, A., Grabowski, A., Panka, D. Diversity in the Polish isolates of *Drechslera teres* in spring barley as determined through morphological features, mating types, reaction to control agents and RAPD markers. *J. Plant Pathol.*, **2012**, *92*, 339-351
18. Gupta, V. K. PCR-RAPD profiling of *Fusarium* spp. causing guava wilt disease in India. *J. Environ. Sci. Health, Part B*, **2012**, *47(4)*, 315-325. https://doi.org/10.1080/03601234.2012.640915
19. Kakvan, N.; Zamanizadeh, H.; Morid, B.; Taheri, H.; Hajmansor, S. Study on pathogenic and genetic diversity of *Alternaria alternata* isolated from citrus hybrids of Iran, based on RAPD-PCR technique. *Eur. J. Exp. Biol.*, **2012**, *2(3)*, 570-576
20. Noruzi, Z.; Moosavi, S. A.; Darvishnia, M.; Azadbakht, N.; Fayazi, F. Investigation of genetic diversity of isolate common smut of corn by using RAPD marker in Lorestan Province. *Agric. Sci. Technol.*, **2012**, *4(4)*, 354-357
21. Que, Y.; Xu, L.; Lin, J.; Chen, R.; Grisham, M. P. Molecular variation of *Sporisorium scitamineum* in Mainland China revealed by RAPD and SRAP markers. *Plant Dis.*, **2012**, *96(10)*, 1519-1525. https://doi.org/10.1094/PDIS-08-11-0663-RE
22. Sangeetha, G.; Anandan, A.; Rani, S. U. Morphological and molecular characterisation of *Lasiodiplodia theobromae* from various banana cultivars causing crown rot disease in fruits. *Arch. Phytopathol. Pflanzenschutz*, **2012**, *45(4)*, 475-486. https://doi.org/10.1080/03235408.2011.587986
23. Sangeeta, M.; Gaur, V. K.; Sharma, R. A.; Jagdish, P. Assessment of genetic variability among *Fusarium oxysporum f. sp. cumini* isolates based on pathogenicity and RAPD markers. *Ind. Phytopathol.*, **2012**, *65(1)*, 76-79
24. Liu, J.; Cao, T.; Feng, J.; Chang, K. F.; Hwang, S. F.; Strelkov, S. E. Characterization of the fungi associated with ascochyta blight of field pea in Alberta, Canada. *Crop Prot.*, **2013**, *54*, 55-64
25. Yadav, B.; Singh, R.; Kumar, A. Genetic variability and relationship analysis of *Bipolaris sorokiniana* isolates causing spot blotch disease in wheat using random amplified polymorphic DNA (RAPD) markers. *Afr. J. Biotechnol.*, **2013**, *12(19)*, 2526-2531.
26. Haghighi, M. T., Shahdoust, E. Molecular analysis of genetic diversity of endophytic *Myrothecium* spp. by RAPD and ISSR markers. *Int. J. Sci. Inven. Today*, **2014**, *3(6)*, 692-704
27. Martín, L.; Saenz de Miera, L. E.; Martín, M. T. AFLP and RAPD characterization of *Phaeoacremonium aleophilum* associated with *Vitis vinifera* decline in Spain. *J. Phytopathol.*, **2014**, *162*, 245-257. https://doi.org/10.1111/jph.12180
28. Nath, V. S.; Sankar, M. S. A.; Hegde, V. M.; Jeeva, M. L.; Misra, R. S.; Veena, S. S.; Raj, M. Analysis of genetic diversity in *Phytophthora colocasiae* causing leaf blight of taro (*Colocasia esculenta*) using AFLP and RAPD markers. *Ann. Microbiol.*, **2014**, *64*, 185-197. https://doi.org/10.1007/s13213-013-0651-8
29. Pereira, M. C.; da Silva Coelho, I.; da Silva Valadares, R. B.; Oliveira, S. F.; Bocayuva, M.; Pereira, O. L.; Araújo, E. F.; Kasuya, M. C. M. Morphological and molecular characterization of *Tulasnella* spp. fungi isolated from the roots of *Epidendrum secundum*, a widespread Brazilian orchid. *Symbiosis*, **2014**, *62*, 111-121. https://doi.org/10.1007/s13199-014-0276-0
30. Singh, P.; Srivastava, M.; Singh, A.; Shahid, M. Genetic variability of *Trichoderma atroviride* isolated from lentil field through RAPD analysis. *Progress. Agric.*, **2014**, *14(1)*, 117-121
31. Akram, M., Singh, P. K., Singh, J., Srivastava, R. Genetic variation in *Alternaria* isolates pathogenic to linseed (*Linum usitatissimum*) based on RAPD. *Curr. Adv. Agric. Sci.*, **2015**, *7(2)*, 147-150. https://doi.org/10.5958/2394-4471.2015.00036.2
32. Hassan Zadeh Davarani, F. Genetic diversity and pathogenesity of *Fusarium solani* isolates of chickpea using RAPD and AFLP markers in Razavi and Northern Khorasan provinces. *Agric. Biotechnol. J.*, **2015**, *7(1)*, 57-74
33. Shinde, A. S., Kalaskar, S. R., Rathod, A. H., Sheikh, W. A., Acharya, S. RAPD based molecular diversity analysis of different *Fusarium udum* butler isolates of pigeon pea wilt. *Int. J. Plant Protect.*, **2015**, *8(1)*, 81-85
34. Sy-Ndir, M.; Assigbetse, K. B.; Nicole, M.; Diop, T. A.; Ba, A. T. Differentiation of *Stemphylium solani* isolates using random amplified polymorphic DNA markers. *Afr. J. Microbiol. Res.*, **2015**, *9(13)*, 915-921. https://doi.org/10.5897/ajmr2015.7365
35. Abass, M. H. Identification of different fungal fruit rot pathogens of date palm (*Phoenix dactylifera L.*) using ITS and RAPD markers. *Basra* J. *Date Palm Res.*, **2016**, *15*, 1-19.
36. Abramczyk, B., & Król, E. Use of RAPD-PCR and ITS markers for identification of *Diaporthe/Phomopsis* from fruit trees in south-eastern Poland. *Acta Sci. Pol.-Hortorum Cultus*, *2016*, *15(6)*, 161-175.

37. Arvindganth, R., Kathiravan, G., Thirugnanasambadam, V. B. T. Genetic diversity and random amplifies polymorphic DNA analysis of *Pestalotia* sp. isolates of endophytes from different host. *Asian J. Pharm. Clin. Res.*, **2016**, *9(1)*, 152-156
38. Sharma, M.; Kapatia, A.; Kulshrestha, S. Analysis of genetic diversity amongst *Fusarium* spp. associated with root rot of apple. *Biosci. Biotechnol. Res. Asia*, **2016**, *13(1)*, 463-468. http://dx.doi.org/10.13005/bbra/2055
39. Singha, I. M.; Kakoty, Y.; Unni, B. G.; Das, J.; Kalita, M. C. Identification and characterization of *Fusarium* sp. using ITS and RAPD causing fusarium wilt of tomato isolated from Assam, North East India. *J. Genet. Eng. Biotechnol.*, **2016**, *14(1)*, 99-105. https://doi.org/10.1016/j.jgeb.2016.07.001
40. Vishwakarma, S. K.; Nigam, A. M. R. I. T. A.; Singh, A. T. U. L. Molecular phylogenetic analysis of *Fusarium* isolates causing pokkah boeng disease in sugarcane based on RAPD marker. *Int. J. Agric. Sci. Res.*, **2016**, *6(3)*, 177-186.
41. Akram, A., Amber, P., Iqbal, S. M., Qureshi, R., Javaid, A., Mukhtar, S. RAPD based characterization of chickpea isolates of *Sclerotium rolfsii*. *Pak. J. Bot.*, **2017**, *49(5)*, 2015-2022.
42. Aggarwal, R., Sharma, S., Gupta, S., Banerjee, S., Bashyal, B. M., Bhardwaj, S. C. Molecular characterization of predominant Indian wheat rust pathotypes using URP and RAPD markers. *Ind. J. Biotechnol.*, **2018**, *17*, 327-336. https://doi.org/10.1590/1678-4685-GMB-2017-0171
43. Chowdhury, D. R.; Chattopadhyay, S. K.; Roy, S. K. Molecular phylogeny and genetic diversity studies of some potent endophytic fungi isolated from medicinal plants (*Calotropis procera* and *Catharanthus roseus*) using 18S rRNA and RAPD analysis. *Afr. J. Microbiol. Res.*, **2018**, *12(33)*, 796-805. https://doi.org/10.5897/AJMR2018.8866
44. Lorenzini, M.; Zapparoli, G. Yeast-like fungi and yeasts in withered grape carposphere: characterization of *Aureobasidium pullulans* population and species diversity. *Int. J. Food Microbiol.*, **2019**, *289*, 223-230. https://doi.org/10.1016/j.ijfoodmicro.2018.10.023
45. Rajput, M. A.; Rajput, N. A.; Syed, R. N.,; Khanzada, M. A.; Khan, I. A.; Lodhi, A. M. Genetic characterization of *Sporisorium scitamineum* isolates in sugarcane through RAPD analysis. *Fresenius Environ. Bull.*, **2020**, *29*, 4355-4363.
46. Cakmakci, S.; Cetin, B.; Gurses, M.; Dagdemir, E.; Hayaloglu, A. A. Morphological, molecular, and mycotoxigenic identification of dominant filamentous fungi from moldy civil cheese. *J. Food Prot.*, **2012**, *75(11)*, 2045-2049. https://doi.org/10.4315/0362-028X.JFP-12-107
47. Gautam, A. K.; Bhadauria, R. Characterization of *Aspergillus* species associated with commercially stored triphala powder. *Afr. J. Biotechnol.*, **2012**, *11(104)*, 16814-16823
48. Shivani, C.; Shammi, K.; Thakur, S. K. RAPD marker assisted development of improved strains of *Agaricus bisporus* (Lange) Sing. *J. Pure Appl. Microbiol.*, **2012**, *6(4)*, 1841-1848.
49. Akhtar, N.; Anjum, T.; Jabeen, R. Isolation and identification of storage fungi from citrus sampled from major growing areas of Punjab, Pakistan. *Int. J. Agric. Biol.*, **2013**, *15(6)*, 1283–1288
50. Abdulmalk, H. W. Rapid differentiation of *Pleurotus ostreatus* from *Pleurotus sapidus* using PCR technique. *Sci. j. Univ. Zakho*, **2013**, *1(1)*, 153-157
51. Vieira, F. R.; Pereira, D. M.; Andrade, M. C. N. D.; Minhoni, M. T. D. A. Molecular characterization of *Pleurotus ostreatus* commercial strains by random amplified polymorphic DNA (RAPD). *Afr. J. Agric. Res.*, **2013**, *8*, 3146-3150
52. Agarwal, K.; Prasad, M. P.; Rindhe, G. Genomic discrimination of eleven commercial mushrooms by DNA fingerprinting using RAPD marker. *Res. J. Biol. Sci.*, **2013**, *2(10)*, 1-5
53. Mahmoud, M. A.; El-Zaidy, M.; Al-Othman, M. R.; Abd El-Aziz, A. R.; Al-Wadai, A. S. Molecular characterization of *Aspergillus flavus* contamination of barley grains using RAPD and ISSR. *J. Pure Appl. Microbiol.*, **2014**, *8*, 77-85.
54. Kumar, V.; Kumar, A.; Kannojia, S. K.; Singh, R. P. Development of molecular marker and variability characterization of *Aspergillus flavus* isolates of chilies (*Capsicum frutescens* L.) through RAPD-PCR and estimation of aflatoxin B1 by indirect competitive ELISA in India. *Open Access Library Journal*, **2014**, *1*, e1161. http://dx.doi.org/10.4236/oalib.1101161
55. Leiminger, J. H.; Auinger, H. J.; Wenig, M.; Bahnweg, G.; Hausladen, H. Genetic variability among *Alternaria solani* isolates from potatoes in Southern Germany based on RAPD-profiles. *J. Plant Dis. Prot.*, **2013**, *120(4)*, 164-172. https://doi.org/10.1007/BF03356470
56. Yin, Y.; Liu, Y.; Li, H.; Zhao, S.; Wang, S.; Liu, Y.; Wu, D.; Xu, F. Genetic diversity of *Pleurotus pulmonarius* revealed by RAPD, ISSR, and SRAP fingerprinting. *Curr. Microbiol.*, **2014**, *68*, 397-403. https://doi.org/10.1007/s00284-013-0489-0
57. Chhotaray, A.; Dhua, U.; Behera, L.; Mukherjee, A. K. Detection of seedborne *Aspergillus flavus* from rice cultivars using molecular markers. *Arch. Phytopathol. Pflanzenschutz*, **2015**, *48(4)*, 297-305. https://doi.org/10.1080/03235408.2014.886420
58. Apollos, W. P.; Joshua, V. I.; Musa, H. D.; Gyang, M. S.; Nyam, D. D. Molecular characterization and phylogenetic analysis of some Agaricomycetes (mushroom) fungi from Kogi State, Central Nigeria. *Int. J. Sci. Basic Appl. Res.*, **2017**, *35(2)*, 276-292.
59. Suržženko, M.; Kontram, K.; Sarand, I. PCR–based fingerprinting and identification of contaminative fungi isolated from rye breads. *Agron. Res.*, **2017**, *15(1)*, 288-297.
60. Yang, C. L.; Wu, X. P.; Chen, B.; Deng, S. S.; Chen, Z. E.; Huang, Y. Y.; Jin, S. S. Comparative analysis of genetic polymorphisms among *Monascus* strains by ISSR and RAPD markers. *J. Sci. Food Agric.*, **2017**, *97(2)*, 636-640. https://doi.org/10.1002/jsfa.7780
61. Razali, S. M.; Lee, H. Y.; Jinap, S.; Mahyudin, N. A. Characterization of fungi from palm kernel cake (PKC) and the effect of storage temperature on fungi growth. *Pertanika J. Trop. Agric. Sci.*, **2018**, *41(1)*, 115-128
62. Sharma, R.; Sharma, B. M.; Sharma, P. N. Genetic diversity analysis of *Pleurotus* spp. in Himachal Pradesh using RAPD fingerprints. *Int. J. Curr. Microbiol. App. Sci.*, **2018**, *7(7)*, 2148-2154. https://doi.org/10.20546/ijcmas.2018.707.253
63. Tiwari, K.; Alhoot, M. A.; Eltariki, F. E. M.; Sharma, A. K. Prevalence and genetic diversity assessment of mycotoxigenic *Colletotricum gleosporioides* in wheat grains collected from different regions of Libya. *Int. J. Med. Toxicol. Leg. Med.*, **2019**, *22(1-2)*, 184-185. https://doi.org/10.5958/0974-4614.2019.00039.1

64. Al-Askar, A. A.; Ghoneem, K. M.; Hafez, E. E.; Saber, W. I. A case study in Saudi Arabia: biodiversity of maize seed-borne pathogenic fungi in relation to biochemical, physiological, and molecular characteristics. *Plants*, **2022**, *11(6)*, 829. https://doi.org/10.3390/plants11060829
65. Bahmani, Z.; Nejad, R. F.; Nourollahi, K.; Fayazi, F.; Mahinpo, V. Investigation of *Fusarium verticillioides* on the basis of RAPD analysis, and vegetative compatibility in Iran. *J. Plant Pathol. Microb.*, **2012**, *3*, 147. https://doi.org/10.4172/2157-7471.1000147
66. Banerjee, S.; Dutta, S.; Mondal, A.; Mandal, N.; Bhattacharya, S. Characterization of molecular variability in *Rhizoctonia solani* isolates from different agro-ecological zones by random amplified polymorphic DNA (RAPD) markers. *Afr. J. Biotechnol.*, **2012**, *11(40)*, 9543-9548.
67. Hegde, V. M.; Jeeva, M. L.; Misra, R. S.; Veena, S. S.; Raj, M. Analysis of genetic diversity in *Phytophthora colocasiae* using RAPD markers. *Asian Australas. J. Plant Sci. Biotechnol.*, **2012**, *6(1)*, 38-43.
68. Hu, Z.; Zhao, L.; Kong, B.; Chen, H.; Fan, J.; Liu, F.; Li, X.; Cai, H.; Yang, G.; Qin, X.; Fang, D. Comparison and analysis of molecular methods ISSR and RAPD used for assessment of genetic diversity in *Alternaria alternata*. Southwest China Journal of Agricultural Sciences, **2012**, *25(3)*, 917-921.
69. Sadeghi, L.; Alizadeh, A.; Safaie, N.; Jamali, S. H. Genetic diversity of *Gaeumannomyces graminis var. tritici* populations using RAPD and ERIC markers. *J. Plant Pathol. Microb.*, **2012**, *3*, 143.
70. Villarino, M.; Larena, I.; Martinez, F.; Melgarejo, P.; De Cal, A. Analysis of genetic diversity in *Monilinia fructicola* from the Ebro Valley in Spain using ISSR and RAPD markers. *Eur. J. Plant Pathol.*, **2012**, *132(4)*, 511-524. https://doi.org/10.1007/s10658-011-9895-y
71. Bonde, S. R.; Gade, A. K.; Rai, M. K. Genetic diversity among fourteen different *Fusarium* species using RAPD marker. *Biodiversitas*, **2013**, *14(2)*, 55-60. https://doi.org/10.13057/biodiv/d140201
72. Ebadi, M.; Riahi, H. Morphological and genetic variation of the *Fusarium semitectum* isolates associated with cereal grains using RAPD markers. *Int. J. Agric. Biosci.*, **2013**, *2(6)*, 327-332
73. Hernandez, A.; Jimenez, M.; Arcia, A.; Ulacio, D.; Mendez, N. Molecular characterization of 12 isolates of *Trichoderma* spp. using RAPD and rDNA-ITS. *Bioagro*, **2013**, *25(3)*, 167-174. ISSN 1316-3361
74. Gad, M. A., Ibrahim, N. A., Bora, T. C. Molecular biodiversity in phytopathogenic fungi, *Pyricularia* spp. *J. Biol. Chem. Res*., **2013**, *30(1)*, 216-226. ISSN 0970-4973
75. Gad, M. A., Ibrahim, N. A., Bora, T. C. RAPD based genetic variation in *Rhizoctonia* sp. in India. *J. Biol. Chem. Res.*, **2013**, *30(1)*, 67-75. ISSN 0970-4973
76. Ortu, G.; Spadaro, D.; Gullino, M. L.; Garibaldi, A. RAPD of isolates of *Fusarium oxysporum f. sp. raphani*, pathogen of cultivated and wild rocket. *Protezione Colture*, **2013**, *2*, 104.
77. Pérez-Martínez, C.; Rojas-Martínez, R. I.; Alatorre-Rosas, R.; Hernández-Rosas, F.; Otero-Colina, G. Análisis de la variación intraespecífica de Conidiobolus coronatus usando RAPD y secuencias ITS. *Enferm. Infecc. Microbiol. Clin.*, **2013**, *31(6)*, 357-362. https://doi.org/10.1016/j.eimc.2012.10.007
78. Uddin, M. N.; Saifullah, M. A.; Ali, I.; Iqbal, A.; Khan, N. U. Genetic characterization of *Verticillium chlamydosporium* isolated from Pakistan using Random Amplified Polymorphic DNA (RAPD) primers. *Pak. J. Bot.*, **2013**, *45(2)*, 467-472
79. Ye, C.; Tan, H.; Wu, Y.; Wu, M.; Zhu, X. Study on the genetic diversity of *Lentinula edodes* 135 and 9015 by random amplified polymorphic DNA (RAPD) markers. *J. Zhejiang Univ. Sci. B*, **2013**, *40(2)*, 230-234.
80. Younes, H. M.; Elamri, N. A.; Farag, I. S. Genetic variation of *Fusarium solani* isolates based on RAPD-PCR analysis. *Persian Gulf Crop Protect.*, **2013**, *2(1)*, 44-51.
81. Choudhary, A. K.; Kumari, P.; Aggarwall, R. RAPD based DNA fingerprinting of toxigenic and non-toxigenic strains of *Aspergillus flavus* isolated from different habitats. *Ind. Phytopathol.*, **2014**, *67(3)*, 291-297.
82. Deshmukh, R.; Mathew, A.; Purohit, H. J. Characterization of antibacterial activity of bikaverin from *Fusarium* sp. HKF15. *J. Biosci. Bioeng.*, **2014**, *117(4)*, 443-448. https://doi.org/10.1016/j.jbiosc.2013.09.017
83. Jorjandi, M.; Baghizadeh, A. Molecular diversity of antagonistic *Botrytis alli*, the agent of onion gray mold using Random Amplified Polymorphic DNA (RAPD) markers. *J. Stress Physiol. Biochem*, **2014**, *10(3)*, 273. ISSN 1997-0838
84. Mustafa, G.; Tahir, A.; Asgher, M.; Rahman, M. U.; Jamil, A. Comparative sequence analysis of citrate synthase and 18S ribosomal DNA from a wild and mutant strains of *Aspergillus niger* with various fungi. *Bioinformation*, **2014**, *10(1)*, 1. . https://doi.org/10.6026/97320630010001
85. Alipour, M.; Mozafari, N. A. Terbinafine susceptibility and genotypic heterogeneity in clinical isolates of *Trichophyton mentagrophytes* by random amplified polymorphic DNA (RAPD). *J. Mycol. Med.*, **2015**, *25(1)*, e1-e9. https://doi.org/10.1016/j.mycmed.2014.09.001
86. Hashoosh, Q. H.; Al-Araji, A. M.; Al-Assie, A. H. Genetic diversity using random amplified polymorphic DNA (RAPD) analysis for *Aspergillus niger* isolates. *Iraqi J. Sci.*, **2015**, *56*, 3376-3389. ISSN: 0067-2904
87. Kamel, E. A. R.; Rashed, M. E. Molecular characterization of *Penicillium* isolates using RAPD technique. *Annu. Res. Rev. Biol.*, **2015**, *7(3)*, 185-199. https://doi.org/10.9734/ARRB/2015/13657
88. Kumari, K. Studies on characterization of molecular variability using RAPD markers in *Rhizoctonia solani* isolated from different geographical regions of South India. *Int. J. App. Sci. Biotechnol.*, **2015**, *3(4)*, 599-603. https://doi.org/10.3126/ijasbt.v3i4.13684
89. Mishra, P. K. Genotyping of *Rhizoctonia solani* isolates obtained from various hosts. *Trends Biosci.*, **2015**, *8(23)*, 6630-6633. ISSN 0974-8431
90. Naik, R. B.; Rao, A. R.; Krishna, T. M.; Devaki, K. Molecular characterization of *Beauveria bassiana* isolates through RAPD-PCR. *Curr. Biot.*, **2015**, *9(2)*, 105-118.

91. Prabhukarthikeyan, S. R.; Keerthana, U.; Archana, S.; Raguchander, T. Analysis of genetic diversity among different isolates of *Beauveria bassiana* by RAPD-PCR. *JBC*, **2017**, *31(1)*, 18-24. https://doi.org/10.18311/jbc/2017/15581
92. Renu, T.; Pooja, K.; Sharma, P. N.; Sharma, B. M. Assessment of genetic diversity in *Ganoderma lucidum* using RAPD and ISSR markers. *Ind. Phytopathol.*, **2015**, *68(3)*, 316-320
93. Ren, W.; Zhao, H.; Shao, W.; Ma, W.; Wang, J.; Zhou, M.; Chen, C. Identification of a novel phenamacril-resistance-related gene by the cDNA-RAPD method in *Fusarium asiaticum*. *Pest Manag. Sci.*, **2016**, *72(8)*, 1558-1565. https://doi.org/10.1002/ps.4186
94. Tok, F. M.; Derviş, S.; Arslan, M. Analysis of genetic diversity of *Sclerotinia sclerotiorum* from eggplant by mycelial compatibility, random amplification of polymorphic DNA (RAPD) and simple sequence repeat (SSR) analyses. *Biotechnol. Biotechnol. Equip.*, **2016**, *30(5)*, 921-928. https://doi.org/10.1080/13102818.2016.1208059
95. Irfan, M.; Yang, S.; Yuxin, L.; Sun, J. X. Genetic diversity analysis of *Morchella* sp. by RAPD. *Mol. Biol. Res. Commun.*, **2017**, *6(1)*, 27. PMID: 28447046
96. Thomas, B. T.; Ogunkanmi, L. A.; Iwalokun, B. A.; Popoola, D. Genetic diversity of ochratoxigenic *Aspergillus* section Nigri, using RAPD and VCG techniques. *Afr. J. Clinic. Exp. Microbiol.*, **2017**, *18(4)*, 205-209. https://doi.org/10.4314/ajcem.v18i4.4
97. Baria, T. T. Molecular characterization of *Fusarium pallidoroseum* isolates by using RAPD technique. *Green Farming*, **2018**, *9(2)*, 357-360.
98. Dwivedi, S.; Singh, S.; Chauhan, U. K.; Tiwari, M. K. Inter and intraspecific genetic diversity (RAPD) among three most frequent species of macrofungi (*Ganoderma lucidum*, *Leucoagricus* sp. and *Lentinus* sp.) of tropical forest of Central India. *J. Genet. Eng. Biotechnol.*, **2018**, *16(1)*, 133-141. https://doi.org/10.1016/j.jgeb.2017.11.008
99. Kaushal, S.; Chandel, S.; Sharma, M.; Kashyap, P. Morphological and molecular diversity study among the isolates of *Trichoderma* using RAPD markers. *Int. J. Chem. Stud.*, **2018**, *6(4)*, 1731-1735. ISSN: 2349–8528
100. Patel, P.; Rajkumar, B. K.; Parmar, P.; Shah, R.; Krishnamurthy, R. Assessment of genetic diversity in *Colletotrichum falcatum* Went accessions based on RAPD and ISSR markers. *J. Genet. Eng. Biotechnol.*, **2018**, *16(1)*, 153-159. https://doi.org/10.1016/j.jgeb.2017.11.006
101. Yang, H.; Tan, Y.; Su, W.; Qin, Y.; Ma, T.; Pu, H.; Zhou, Z. Genetic diversity analysis of white *Flammulina velutipes* strains based on RAPD. *Acta Agriculturae Jiangxi*, **2019**, *31(2)*, 62-65. https://doi.org/10.1016/j.gene.2016.07.009
102. Razaq, A.; Shaheen, S.; Nafees, M. A.; Rajput, A. Q.; Shahzad, S. Morphological and molecular characterization of macro fungi from district Astore, Gilgit-Baltistan, Pakistan. *Pak. J. Bot.*, **2020**, *52(6)*, 2229-2235. http://dx.doi.org/10.30848/PJB2020-6(32)
103. Sharma, S.; Khanna, P. K.; Kapoor, S. Molecular characterization of shiitake medicinal mushroom, *Lentinus edodes* strains (higher Basidiomycetes) using RAPD and ITS sequencing. *Int. J. Med. Mushrooms*, **2014**, *16*, 169-177. https://doi.org/10.1615/IntJMedMushr.v16.i2.70
104. Fu, J. J.; Mei, Z. Q.; Tania, M.; Yang, L. Q.; Cheng, J. L.; Khan, M. A. Development of RAPD-SCAR markers for different *Ganoderma* species authentication by improved RAPD amplification and molecular cloning. *Genet. Mol. Res.*, 14, 2015, 5667-5676. http://dx.doi.org/10.4238/2015.May.25.19
105. Okafor, U. A.; Ebuehi, A. O.; Okochi, V. I.; Adekunle, A.; Magbagbeola, O. A. Pectinase hyperproduction in fungi isolates. Experimental Biology 2013 Meeting Abstracts, 27, 2013, 570.1-570.1. https://doi.org/10.1096/fasebj.27.1_supplement.570.1
106. Helen, P. M.; Shiny, M.; Ruskin, S.; Sree, S. J.; Nizzy, A. M. Screening of antibiotic producing fungi from soil. *J. Environ. Sci. Comp. Sci. Engg. Technol.*, **2012**, *1*, 141-151. E-ISSN: 2278–179X
107. Othira, J. O.; Omolo, J. O.; Kiruki, S.; Onek, L. A.; Wachira, F. N. Molecular diversity of arbuscular mycorrhizal fungi (AMF) in Lake Victoria Basin of Kenya. *J. Ecol. Nat. Environ.*, **2014**, *6(4)*, 145-152. https://doi.org/10.5897/JENE2014.0437
108. Al, S. A. D. A. S; Alwan, A. H. Molecular identification of rhizosphere *Trichoderma* spp. and their antagonistic impact against some plant pathogenic fungi. *Baghdad Sci. J.*, **2016**, *13(1)*, 53-65. : http://dx.doi.org/10.21123/bsj.2016.13.1.0053
109. Shakya, S.; Tripathi, N.; Bhadauria, S. Molecular analysis of *Rhizopus* fungal species on deteriorating historical sandstone monuments: an advanced approach. New Visions in Biological Science, **2021**, *4*, 31-38. https://doi.org/10.9734/bpi/nvbs/v4/4847F
110. Singh, V.; Wahi, N.; Garg, G.; Singh, A.; Bhadauria, S. Ecological and genetical diversity of black fungus-*Aspergillus* sp. inhabiting stone monuments of Mughal dynasty in Agra (UP) analysed by RAPD method. *Arch. Phytopathol. Pflanzenschutz*, **2022**, 55(7), 815-832. https://doi.org/10.1080/03235408.2022.2047377

References: Supplementary Table S3

1. Deng, S.; Tsui, C. K.; Gerrits Van Den Ende, A. H. G.; Yang, L.; Najafzadeh, M. J.; Badali, H.; Li, R.; Hagen, F.; Meis, J. F.; Sun, J.; Dolatabadi, S.; Papierok, B.; Pan, W.; de Hoog, G. S.; Liao, W. Global spread of human chromoblastomycosis is driven by recombinant *Cladophialophora carrionii* and predominantly clonal *Fonsecaea* species. *PLoS Negl. Trop. Dis.*, **2015**, *9*, e0004004. https://doi.org/10.1371/journal.pntd.0004004
2. Kanani, A.; Zaini, F.; Kordbacheh, P.; Falahati, M.; Rezaie, S.; Daie, R.; Farahyar, S; Safara, M.; Fateh, R.; Faghihloo, E.; Fattahi, A.; Heidari, M. Identification of azole resistance markers in clinical isolates of *Candida tropicalis* using cDNA-AFLP method. *J. Clin. Lab. Anal.*, **2016**, *30*, 266-272. https://doi.org/10.1002/jcla.21847
3. Prakash, H.; Ghosh, A. K.; Rudramurthy, S. M.; Paul, R. A.; Gupta, S.; Negi, V.; Chakrabarti, A. The environmental source of emerging *Apophysomyces variabilis* infection in India. *Sabouraudia*, **2016**, *54(6)*, 567-575. https://doi.org/10.1093/mmy/myw014
4. Zhao, Z; Fu, Y-X; Hewett,-E. D., Boerwinkle E. Investigating single nucleotide polymorphism (SNP) density in the human genome and its implications for molecular evolution. Gene, **2003**, *312*, 207–213. https://doi.org/10.1016/S0378-1119(03)00670-X

5. Marchetta, A.; Gerrits van den Ende, B.; Al-Hatmi, A.; Hagen, F.; Zalar, P.; Sudhadham, M.; Gunde-Cimerman, N; Urzí, C.; De Hoog , S.; De Leo, F. Global molecular diversity of the halotolerant fungus *Hortaea werneckii*. *Life*, **2018**, *8(3)*, 31. https://doi.org/10.3390/life8030031
6. Engel, T. G.; Slabbers, L.; de Jong, C.; Melchers, W. J.; Hagen, F.; Verweij, P. E.; Merkus, P.; Meis, J, F. Dutch cystic fibrosis fungal collection consortium. prevalence and diversity of filamentous fungi in the airways of cystic fibrosis patients–a dutch, multicentre study. *J. Cyst. Fibros.*, **2019**, *18*, 221-226. https://doi.org/10.1016/j.jcf.2018.11.012
7. Gao, Y.; Zhan, P.; Hagen, F.; Menken, S. B.; Sun, J.; Rezaei-Matehkolaei, A.; de Hoog, S. Molecular epidemiology and in vitro antifungal susceptibility of *Trichophyton schoenleinii*, agent of *tinea capitis favosa*. *Mycoses*, **2019**, *62(5)*, 66-474. https://doi.org/10.1111/myc.12889
8. de Carvalho, J. A.; Hagen, F.; Fisher, M. C.; de Camargo, Z. P.; Rodrigues, A. M. Genome-wide mapping using new AFLP markers to explore intraspecific variation among pathogenic *Sporothrix* species. *PLoS Negl. Trop. Dis.*, **2020**, *14*, e0008330. https://doi.org/10.1371/journal.pntd.0008330
9. Roberto, T. N.; de Carvalho, J. A.; Beale, M. A.; Hagen, F.; Fisher, M. C.; Hahn, R. C.; de Camargo, Z. P.; Rodrigues, A. M. Trends in the molecular epidemiology and population genetics of emerging *Sporothrix* species. *Stud. Mycol.*, **2021**, *100(1)*, 100131-100131. https://doi.org/10.1016/j.simyco.2021.100131
10. Vatanshenassan, M.; Boekhout, T.; Mauder, N.; Robert, V.; Maier, T.: Meis, J. F.; Berman, J.; Then, E.; Kostrzewa, M.; Hagen, F. Evaluation of microsatellite typing, ITS sequencing, AFLP fingerprinting, MALDI-TOF MS, and Fourier-transform infrared spectroscopy analysis of *Candida auris*. *J. Fungi*, **2020**, *6(3)*, 146. https://doi.org/10.3390/jof6030146
11. Roberto, T. N.; De Carvalho, J. A.; Beale, M. A.; Hagen, F.; Fisher, M. C.; Hahn, R. C.; Camargo, Z. P.; Rodrigues, A. M. Exploring genetic diversity, population structure, and phylogeography in *Paracoccidioides* species using AFLP markers. *Stud. Mycol.*, **2021**, 100, 100131. https://doi.org/10.1016/j.simyco.2021.100131
12. Badali, H.; Yazdanparast, S. A.; Bonifaz, A.; Mousavi, B.; de Hoog, G. S.; Klaassen, C. H.; Meis, J. F. *Veronaea botryosa*: molecular identification with amplified fragment length polymorphism (AFLP) and in vitro antifungal susceptibility. *Mycopathol.*, **2013**, *175*, 505-513. https://doi.org/10.1007/s11046-013-9631-6
13. Duarte-Escalante, E.; Zúñiga, G.; Frías-De-León, M. G.; Canteros, C.; Castañón-Olivares, L. R.; del Reyes-Montes, M. R. AFLP analysis reveals high genetic diversity but low population structure in *Coccidioides posadasii* isolates from Mexico and Argentina. *BMC Infect. Dis.*, 13, 2013, 1-9. https://doi.org/10.1186/1471-2334-13-411
14. Hagen, F.; Ceresini, P. C.; Polacheck, I.; Ma, H.; Nieuwerburgh, F. V.; Gabaldón, T.; Kagan S.; Pursall, E. R.; Hoogveld, H. L.; van Iersel, L. J. J.; Klau, G. W.; Kelk, S. M.; Stougie, L.; Bartlett, K. H.; Voelz, K.; Pryszcz, L. P.; Castañeda, E.; Lazera, M.; Meyer, W.; Deforce, D.; Meis, J. F.; May, R. C.; Klaassen, C. H. W.; Boekhout, T. Ancient dispersal of the human fungal pathogen *Cryptococcus gattii* from the Amazon rainforest. *PloS One*, **2013**, *8*, e71148. https://doi.org/10.1371/journal.pone.0071148
15. Prakash, A.; Randhawa, H. S.; Khan, Z. U.; Ahmad, S.; Hagen, F.; Meis, J. F.; Chowdhary, A. Environmental distribution of *Cryptococcus* species and some other yeast-like fungi in India. *Mycoses*, 61, 2018, 305-313. https://doi.org/10.1111/myc.12741
16. Vicente, V. A.; Orélis-Ribeiro, R.; Najafzadeh, M. J.; Sun, J.; Guerra, R. S.; Miesch, S.; Ostrensky, A.; Meis, J. F.; Klaassen, C. H.; de Hoogahi, G.S.; Boeger, W. A. Black yeast-like fungi associated with Lethargic Crab Disease (LCD) in the mangrove-land crab, *Ucides cordatus* (Ocypodidae). *Vet. Microbiol.*, **2012**, *158*, 109-122. https://doi.org/10.1016/j.vetmic.2012.01.031
17. Arahana, V. S.; Bastidas, N.; de Lourdes Torres, M.; González, P. Diversidad genética de una colección de hongos entomopatógenos de Ecuador utilizando un enfoque AFLP modificado. *Av. en Cienc. e Ing.*, **2013**, *5(1)*, https://doi.org/10.18272/aci.v5i1.121
18. Trissi, A. N.; El Bouhsini, M.; Alsalti, M. N.; von Korff, M.; Hamwieh, A.; Skinner, M.; Parker, B. L.; Baum, M. Genetic diversity among summer and winter *Beauveria bassiana* populations as revealed by AFLP analysis. *J. Asia-Pac. Entomol.*, **2013**, *16*, 269-273. https://doi.org/10.1016/j.aspen.2013.03.006
19. Rezinciuc, S.; Galindo, J.; Montserrat, J.; Diéguez-Uribeondo, J. AFLP-PCR and RAPD-PCR evidences of the transmission of the pathogen *Aphanomyces astaci* (Oomycetes) to wild populations of European crayfish from the invasive crayfish species, *Procambarus clarkii*. *Fungal Biol.*, **2014**, *118*, 612-620. https://doi.org/10.1016/j.funbio.2013.10.007
20. Keyser, C. A.; De Fine Licht, H. H.; Steinwender, B. M.; Meyling, N. V. Diversity within the entomopathogenic fungal species Metarhizium flavoviride associated with agricultural crops in Denmark. *BMC Microbiol.*, **2015**, *15*, 1-11. https://doi.org/10.1186/s12866-015-0589-z
21. Kooij, P. W.; Poulsen, M.; Schiøtt, M.; Boomsma, J. J. Somatic incompatibility and genetic structure of fungal crops in sympatric *Atta colombica* and *Acromyrmex echinatior* leaf-cutting ants. *Fungal Ecol.*, **2015**, *18*, 10-17. https://doi.org/10.1016/j.funeco.2015.08.003
22. Galidevara, S.; Reineke, A.; Koduru, U. D. In vivo expression of genes in the entomopathogenic fungus *Beauveria bassiana* during infection of lepidopteran larvae. *J. Invertebr. Pathol.*, **2016**, *136*, 32-34. https://doi.org/10.1016/j.jip.2016.03.002
23. Abadio, A. K. R.; Lima, S. S.; Santana, M. F.; Salomão, T. M. F.; Sartorato, A.; Mizubuti, E. S. G.; Araújo, E. F.; de Queiroz, M. V. Genetic diversity analysis of isolates of the fungal bean pathogen *Pseudocercospora griseola* from central and southern Brazil. *Embrapa Arroz e Feijão-Artigo em periódico indexado (ALICE)*. **2012**.
24. Aduramigba-Modupe, A. O.; Asiedu, R.; Odebode, A. C.; Owolade, O. F. Genetic diversity of *Colletotrichum gloeosporioides* in Nigeria using amplified fragment length polymorphism (AFLP) markers. *Afr. J. Biotechnol.*, **2012**, *11*. https://doi.org/8189-8195. 10.5897/AJB11.230
25. Ghazvini, H.; Tekauz, A. Molecular diversity in the barley pathogen *Bipolaris sorokiniana* (*Cochliobolus sativus*). *Australas. Plant Pathol.*, **2012**, *41*, 283-293. https://doi.org/10.1007/s13313-012-0131-9

26. Leisova-Svobodova, L.; Minarikova, V.; Kucera, L.; Pereyra, S. A. Structure of the *Cochliobolus sativus* population variability. *Plant Pathol.*, 61, 2012, 709-718. https://doi.org/10.1111/j.1365-3059.2011.02547.x
27. Guo, H.; Yao, Q.; Chen, L.; Wang, F.; Lang, X.; Pang, Y.; ; Xu, S. Virulence and molecular diversity in the *Cochliobolus sativus* population causing barley spot blotch in China. *Plant Dis.*, **2019**, *103*, 2252-2262. https://doi.org/10.1094/PDIS-11-18-2103-RE
28. Karimi, S.; Mirlohi, A.; Sabzalian, M. R.; Sayed Tabatabaei, B. E.; Sharifnabi, B. Molecular evidence for Neotyphodium fungal endophyte variation and specificity within host grass species. Mycol., 104, 2012, 1281-1290.
29. Kuang, W.; Zhang, Z.; Ji, H.; Xiang, Y.; Zhang, M.; Peng, Y. Population diversity of *Puccinia striiformis* in Linzhi of Tibet. Southwest China Journal of Agricultural Sciences, **2012**, *25*, 1668-1673. ISSN : 1001-4829
30. Mokrani, L.; Jawhar, M.; Shoaib, A.; Arabi, M. I. E. Characterization of *Pyrenophora graminea* markers associated with a locus conferring virulence on barley. *Plant Pathol. J.*, **2012**, *28(3)*, 290-294. https://doi.org/10.5423/PPJ.NT.11.2011.0217
31. Newman, Z.; Freeman, S.; Biton, I.; Sa'ada, D.; Paz, T.; Maymon, M.; Lavi, U. Molecular diagnosis of mango malformation disease and phylogeny of *Fusarium mangiferae*. *Phytoparasitica*, **2012**, *40*, 287-297. https://doi.org/10.1007/s12600-012-0224-6
32. Prom, L. K.; Perumal, R.; Erattaimuthu, S. R.; Little, C. R.; No, E. G.; Erpelding, J. E.; Rooney, W. L.; Odvody, G. N.; Magill, C. W. Genetic diversity and pathotype determination of *Colletotrichum sublineolum* isolates causing anthracnose in sorghum. *Eur. J. Plant Pathol.*, **2012**, *133*, 671-685. https://doi.org/10.1007/s10658-012-9946-z
33. Setti, B.; Bencheikh, M.; Henni, D. E.; Neema, C. Genetic variability and population structure of *Mycosphaerella pinodes* in western Algeria using AFLP fingerprinting. *J. Plant Pathol.*, **2012**, *94*, 127-133. https://www.jstor.org/stable/45156016
34. Shiraishi, A.; Leslie, J. F.; Zhong, S.; Uchida, J. Y. AFLP, pathogenicity, and VCG analyses of *Fusarium oxysporum* and *Fusarium pseudocircinatum* from Acacia koa. *Plant Dis.*, **2012**, *96*, 1111-1117. https://doi.org/10.1094/PDIS-06-11-0491
35. Sibounnavong, P.; Unartngam, J.; Soytong, K. Genetic variation of *Fusarium oxysporum f. sp. lycopersici* isolated from tomatoes in Thailand using pathogenicity and AFLP markers. *Afr. J. Microbiol. Res.*, **2012**, *6*, 5636-5644. https://doi.org/10.5897/AJMR12.360
36. Sivaramakrishnan, S.; Kannan, S.; Singh, S. D. Detection of genetic variability in *Fusarium udum* using DNA markers. *Indian Phytopathol.*, **2012**, *55*, 258-263. ISSN : 2248-9800
37. Skrede, I.; Carlsen, T.; Stensrud, Ø.; Kauserud, H. Genome wide AFLP markers support cryptic species in *Coniophora* (Boletales). *Fungal Biol.*, **2012**, *116*, 778-784. https://doi.org/10.1016/j.funbio.2012.04.009
38. , M.; Chen, W. Q.; Liu, D.; Liu, T. G.; Gao, L.; Shu, K. Identification of a specific SCAR marker for detection of *Tilletia foetida* (Wall) Liro pathogen of wheat. *Russ. J. Genet.*, **2012**, *48*, 663-666. https://doi.org/10.1134/S1022795412050237
39. Casasnovas, F.; Fantini, E. N.; Palazzini, J. M.; Giaj-Merlera, G.; Chulze, S. N.; Reynoso, M. M.; Torres, A. M. Development of amplified fragment length polymorphism (AFLP)-derived specific primer for the detection of *Fusarium solani* aetiological agent of peanut brown root rot. *J. App. Microbiol.*, **2013**, *114*, 1782-1792. https://doi.org/10.1111/jam.12183
40. Della Rocca, G.; Osmundson, T.; Danti, R.; Doulis, A.; Pecchioli, A.; Donnarumma, F.; Casalone, E.; Garbelotto, M. AFLP analyses of California and Mediterranean populations of *Seiridium cardinale* provide insights on its origin, biology and spread pathways. *For. Pathol.*, **2013**, *43*, 211-221. https://doi.org/10.1111/efp.12019
41. Maia, T. A.; Maciel-Zambolim, E.; Caixeta, E. T.; Mizubuti, E. S. G.;Zambolim, L. The population structure of *Hemileia vastatrix* in Brazil inferred from AFLP. *Australas. Plant Pathol.*, **2013**, *42*, 533-542. https://doi.org/10.1007/s13313-013-0213-3
42. Martin, L.; Saenz de Miera, L. E.; Martín, M. T. Applicability of AFLP fingerprinting markers to molecular discrimination of the three main fungal species associated with grapevine decline in Spain. *J. Phytopathol.*, **2013**, *161*, 689-695. https://doi.org/10.1111/jph.12118
43. Oviedo, M. S.; Sturm, M. E.; Reynoso, M. M.; Chulze, S. N.; Ramirez, M. L. Toxigenic profile and AFLP variability of *Alternaria alternata* and *Alternaria infectoria* occurring on wheat. *Braz. J. Microbiol.*, **2013**, *44*, 447-455.
44. Sarkar, S.; Girisham, S.; Reddy, S. M. Identification of three fruit-rot fungi of banana by 28S ribosomal DNA sequencing. *Int. J. Bioassays*, **2013**, *2*, 422-429. ISSN: 2278-778X
45. Silva, A. D. S.; Oliveira, E. J. D.; Haddad, F.; Laranjeira, F. F.; Jesus, O. N. D.; Oliveira, S. A. S. D.; Costa, M. A. P. C.; Freitas, J. P. X. D. Identification of passion fruit genotypes resistant to *Fusarium oxysporum f. sp. passiflorae*. *Trop. Plant Pathol.*, **2013**, *38*, 236-242. https://doi.org/10.1590/S1982-56762013005000008
46. Xu, X.; Harvey, N.; Roberts, A.; Barbara, D. Population variation of apple scab (*Venturia inaequalis*) within mixed orchards in the UK. Eur. J. Plant Pathol., **2013**, *135(1)*, 97-104. https://doi.org/10.1007/s10658-012-0068-4
47. Martín, L.; Saenz de Miera, L. E.; Martín, M. T. AFLP and RAPD characterization of *Phaeoacremonium aleophilum* associated with Vitis vinifera decline in Spain. *J. Phytopathol.*, **2014**, *162(4)*, 245-257. https://doi.org/10.1111/jph.12180
48. Nath, V. S.; Sankar, M. S. A.; Hegde, V. M.; Jeeva, M. L.; Misra, R. S.; Veena, S. S.; Raj, M. Analysis of genetic diversity in *Phytophthora colocasiae* causing leaf blight of taro (*Colocasia esculenta*) using AFLP and RAPD markers. *Ann. Microbiol.*, **2014**, *64*, 185-197. https://doi.org/10.1007/s13213-013-0651-8
49. Niknam, G. M.; Salehi, J. G.; Javan, N. M. Characterization and phylogenetic analysis of *Magnaporthe* spp. strains on various hosts in Iran. *Iran. J. Biotechnol.*, **2014**, *12*, 71-81
50. Shjerve, R. A.; Faris, J. D.; Brueggeman, R. S.; Yan, C.; Zhu, Y.; Koladia, V.; Friesen, T. L. Evaluation of a *Pyrenophora teres* f. teres mapping population reveals multiple independent interactions with a region of barley chromosome 6H. *Fungal Genet. Biol.*, **2014**, *70*, 104-112. https://doi.org/10.1016/j.fgb.2014.07.012
51. Steenkamp, E. T.; Makhari, O. M.; Coutinho, T. A.; Wingfield, B. D.; Wingfield, M. J. Evidence for a new introduction of the pitch canker fungus *Fusarium circinatum* in South Africa. *Plant Pathol.*, **2014**, *63(3)*, 530-538. https://doi.org/10.1111/ppa.12136
52. Leboldus, J. M.; Kinzer, K.; Richards, J.; Ya, Z.; Yan, C.; Friesen, T. L.; Brueggeman, R. Genotype-by-sequencing of the plant-pathogenic fungi *Pyrenophora teres* and *Sphaerulina musiva* utilizing Ion Torrent sequence technology. *Mol. Plant Pathol.*, **2015**, *16*, 623-632. https://doi.org/10.1111/mpp.12214

53. Lourdes Rocha, C. M.; Vellicce, G. R.; García, M. G.; Pardo, E. M.; Racedo, J.; Perera, M. F.; de Lucía, A.; Gilli, J.; Bogado, N.; Bonnecarrère, V.; German, S.; Marcelino, F.; Ledesma, F.; Reznikova, S.; Plopera, L. D.; Welina, B.; Castagnaro, A. P. Use of AFLP markers to estimate molecular diversity of *Phakopsora pachyrhizi*. *Electron. J. Biotechnol.*, **2015**, *18*, 439-444. http://dx.doi.org/10.1016/j.ejbt.2015.06.007
54. Pirondi, A.; Vela-Corcía, D.; Dondini, L.; Brunelli, A.; Pérez-García, A.; Collina, M. Genetic diversity analysis of the cucurbit powdery mildew fungus *Podosphaera xanthii* suggests a clonal population structure. *Fungal Biol.*, **2015**, *119*, 791-801. https://doi.org/10.1016/j.funbio.2015.05.003
55. Shen, L.; Zhang, G.; Kuang, W.; Ji, H.; Luo, L.; Ni, J.; Peng, Y. Study on changes in virulence of *Puccinia striiformis f. sp. tritici* on wheat varieties and their mixtures. *Southwest China Journal of Agricultural Sciences*, **2015**, *28*, 1634-1638. ISSN : 1001-4829
56. Shu, C.; Chen, J.; Sun, S.; Zhang, M.; Wang, C.; Zhou, E. Two distinct classes of protein related to GTB and RRM are critical in the sclerotial metamorphosis process of *Rhizoctonia solani* AG-1 IA. *Funct. Integr. Genomics*, **2015**, *15(4)*, 449-459. https://doi.org/10.1007/s10142-015-0435-2
57. Singh, D.; Radhakrishnan, T.; Kumar, V.; Bagwan, N. B.; Basu, M. S.; Dobaria, J. R.; Mishra, G. P.; Chanda, S. V. Molecular characterisation of *Aspergillus flavus* isolates from peanut fields in India using AFLP. *Braz. J. Microbiol.*, **2015**, *46*, 673-682. https://doi.org/10.1590/S1517-838246320131115
58. Zimmermann, J.; de Klerk, M.; Musyoki, M. K.; Viljoen, A.; Watson, A. K.; Beed, F.; Gorfer, M.; Cadisch, G.;Rasche, F. An explicit AFLP-based marker for monitoring *Fusarium oxysporum f. sp. strigae* in tropical soils. *Biol. Control.*, **2015**, *89*, 42-52. https://doi.org/10.1016/j.biocontrol.2015.02.008
59. Alananbeh, K. M.; Gudmestad, N. C. Genetic diversity of *Colletotrichum coccodes* in the United States using amplified fragment length polymorphism analysis. *J. Gen. Plant Pathol.*, **2016**, *82*, 199-211. https://doi.org/10.1007/s10327-016-0662-y
60. Amaradasa, B. S.; Everhart, S. E. Effects of sublethal fungicides on mutation rates and genomic variation in fungal plant pathogen, *Sclerotinia sclerotiorum*. *PLoS One*, **2016**, *11*, e0168079. https://doi.org/10.1371/journal.pone.0168079
61. Al-Hatmi, A. M.; Mirabolfathy, M.; Hagen, F.; Normand, A. C.; Stielow, J. B.; Karami-Osbo, R.; van Diepeningen, A. D.; Meis, J. F.; De Hoog, G. S. DNA barcoding, MALDI-TOF, and AFLP data support *Fusarium ficicrescens* as a distinct species within the *Fusarium fujikuroi* species complex. *Fungal Biol.*, **2016**, *120*, 265-278. https://doi.org/10.1016/j.funbio.2015.08.001
62. Benevenuto, J.; Longatto, D. P.; Reis, G. V.; Mielnichuk, N.; Palhares, A. C.; Carvalho, G.; Saito, S.; Quecine, M. C.; Sanguino, A.; Vieira, M. L: C.; Camargo, L. E. A.; Creste, S.; Monteiro-Vitorello, C. B. Molecular variability and genetic relationship among Brazilian strains of the sugarcane smut fungus. *Microbiol. Lett.*, **2016**, *363*, fnw277. https://doi.org/10.1093/femsle/fnw277
63. Ghasemkhani, M.; Garkava-Gustavsson, L.; Liljeroth, E.; Nybom, H. Assessment of diversity and genetic relationships of *Neonectria ditissima*: the causal agent of fruit tree canker. *Hereditas*, **2016**, *153*, 1-11. https://doi.org/10.1186/s41065-016-0011-3
64. Kharkar, H.; Kale, K.; Thakare, P. Molecular characterization of *Pyricularia grisea* isolates using AFLP DNA fingerprinting. *J. Pure Appl. Microbiol.*, **2016**, *10*, 1181-1189
65. Tsehaye, H.; Elameen, A.; Tronsmo, A. M.; Sundheim, L.; Tronsmo, A.; Assefa, D.; Brurberg, M. B. Genetic variation among *Fusarium verticillioides* isolates associated with Ethiopian maize kernels as revealed by AFLP analysis. *Eur. J. Plant Pathol.*, **2016**, *146*, 807-816. https://doi.org/10.1007/s10658-016-0958-y
66. Zhu, J.; Zhou, Y.; Shang, Y.; Hua, W.; Wang, J.; Jia, Q.; Jia, Q.; Liu, M.; Yang, J. Genetic evidence of local adaption and long distance migration in *Blumeria graminis f. sp. hordei* populations from China. *J. Gen. Plant Pathol.*, **2016**, *82*, 69-81. https://doi.org/10.1007/s10327-016-0643-1
67. Alizadeh, A.; Javan-Nikkhah, M.; Salehi Jozani, G. R.; Fotouhifar, K.; Roodbar Shojaei, T.; Rahjoo, V.; Taherkhani, K. AFLP, pathogenicity and mating type analysis of Iranian *Fusarium proliferatum* isolates recovered from maize, rice, sugarcane and onion. *Mycol. Iran.*, **2017**, *4*, 13-28. https://doi.org/10.22043/MI.2017.115180
68. Alvi, A. Characterising populations of *Puccinia striiformis f. sp. tritici* (Pst) using molecular markers (Doctoral dissertation, University of Nottingham). 2017.
69. Chaerani, C.; Kardin, M.; Suhardi, S.; Sofiari, E.; van Ginkel, R. V.; Groenwolt, R.; Voorrips, R. E. Variation in aggressiveness and AFLP among *Alternaria solani* isolates from indonesia. Indones. *J. Agric. Sci.*, **2017**, *18*, 51-62. http//dx.doi.org/10.21082/ijas.v.18.n2.2017
70. Frézal, L.; Jacqua, G.; Neema, C. Adaptation of a fungal pathogen to host quantitative resistance. *Front. Plant Sci.*, **2018**, *9*, 1554. https://doi.org/10.3389/fpls.2018.01554
71. Pertile, G.; Panek, J.; Oszust, K.; Siczek, A.; Frąc, M. Intraspecific functional and genetic diversity of *Petriella setifera*. PeerJ, **2018**, *6*, e4420. https://doi.org/10.7717/peerj.4420
72. Adusei-Fosu, K.; Dickinson, M.; Yankey, E. N. AFLP as a fingerprinting tool for characterising isolates of *Fusarium oxysporum f. sp. elaeidis* causal organism for fusarium wilt disease of oil palm in Ghana. *J. Plant Dis. Prot.*, **2019**, *126*, 575-584. https://doi.org/10.1007/s41348-019-00241-9
73. Chi, M. H.; Park, S. Y. Identification of differentially expressed genes by cDNA-AFLP in *Magnaporthe oryzae*. *Res. Plant Dis.*, **2019**, *25*, 205-212. https://doi.org/10.5423/RPD.2019.25.4.205
74. Jiang, D.; Xu, C.; Han, W.; Harris-Shultz, K.; Ji, P.; Li, Y.; Zhao, T. Identification of fungal pathogens and analysis of genetic diversity of *Fusarium tricinctum* causing root rots of alfalfa in north-east China. *Plant Pathol.*, **2021**, *70*, 804-814. https://doi.org/10.1111/ppa.13333
75. McTaggart, A. R.; Drenth, A.; Wingfield, M. J.; O'Dwyer, C.; Shuey, L. S.; Pegg, G. S. Epidemic spread of smut fungi (Quambalaria) by sexual reproduction in a native pathosystem. *Eur. J. Plant Pathol.*, **2022**, 1-9. https://doi.org/10.1007/s10658-022-02480-3

76. Covarelli, L.; Stifano, S.; Beccari, G.; Raggi, L.; Lattanzio, V. M. T.; Albertini, E. Characterization of *Fusarium verticillioides* strains isolated from maize in Italy: Fumonisin production, pathogenicity and genetic variability. *Food Microbiol.*, **2012**, *31(1)*, 17-24. https://doi.org/10.1016/j.fm.2012.02.002
77. Gallo, A.; Stea, G.; Battilani, P.; Logrieco, A. F.; Perrone, G. Molecular characterization of an *Aspergillus flavus* population isolated from maize during the first outbreak of aflatoxin contamination in Italy. *Phytopathol. Mediterr.*, **2012**, *51*, 198-206
78. Pawlik, A.; Janusz, G.; Koszerny, J.; Małek, W.; Rogalski, J. Genetic diversity of the edible mushroom *Pleurotus* sp. by amplified fragment length polymorphism. *Curr. Microbiol.*, **2012**, *65*, 438-445. https://doi.org/10.1007/s00284-012-0175-7
79. Saleh, A. A.; Esele, J. P.; Logrieco, A.; Ritieni, A.; Leslie, J. F. *Fusarium verticillioides* from finger millet in Uganda. *Food Addit Contam Part A*, **2012**, *29(11)*, 1762-1769. https://doi.org/10.1080/19440049.2012.712062
80. Foulongne-Oriol, M.; Navarro, P.; Spataro, C.; Ferrer, N.; Savoie, J. M. Deciphering the ability of *Agaricus bisporus var. burnettii* to produce mushrooms at high temperature (25 C). *Fungal Genet. Biol.*, **2014**, *73*, 1-11. https://doi.org/10.1016/j.fgb.2014.08.013
81. Hammami, W.; Fiori, S.; Al Thani, R.; Kali, N. A.; Balmas, V.; Migheli, Q.; Jaoua, S. Fungal and aflatoxin contamination of marketed spices. *Food Control*, **2014**, *37*, 177-181. https://doi.org/10.1016/j.foodcont.2013.09.027
82. Fakruddin, M.; Chowdhury, A.; Hossain, M. N.; Ahmed, M. M. Characterization of aflatoxin producing *Aspergillus flavus* from food and feed samples. *SpringerPlus*, **2015**, *4*, 1-6. https://doi.org/10.1186/s40064-015-0947-1
83. Pawlik, A.; Malinowska, A.; Siwulski, M.; Frąc, M.; Rogalski, J.; Janusz, G. Determination of biodiversity of *Coprinus comatus* using genotyping and metabolic profiling tools. *Acta Biochim. Pol.*, **2015**, 62. http://dx.doi.org/10.18388/abp.2015_1102
84. Massi, F. P.; Vieira, M. L. C.; Sartori, D.; Penha, R. E. S.; de Freitas Munhoz, C.; Ferreira, J. M.; ThieIamanaka, B.; Taniwaki, M. H.; Frisvad, J. C.; Fungaro, M. H. P. Brazil nuts are subject to infection with B and G aflatoxin-producing fungus, *Aspergillus pseudonomius*. *Int. J. Food Microbiol.*, **2014**, *186*, 14-21. https://doi.org/10.1016/j.ijfoodmicro.2014.06.006
85. Rahimi, S.; Sohrabi, N.; Ebrahimi, M. A.; Tebyanian, M.; Taghyzadeh, M.; Rahimi, S. A. Studying the effect of aflatoxin genes Aflp and Aflq on *Aspergillus flavus* and *Aspergillus parasiticus* in the cattle feed used in industrial animal husbandries. *Acta Medica Mediterr.*, **2016**, *32*, 2091-2100
86. Viaro, H. P.; da Silva, J. J.; de Souza Ferranti, L.; Bordini, J. G.; Massi, F. P.; Fungaro, M. H. P. The first report of *A. novoparasiticus*, *A. arachidicola* and *A. pseudocaelatus* in Brazilian corn kernels. *Int. J. Food Microbiol.*, **2017**, *243*, 46-51. https://doi.org/10.1016/j.ijfoodmicro.2016.12.002
87. Castell-Miller, C. V; Samac, D. A. Population genetic structure, gene flow and recombination of *Cochliobolus miyabeanus* on cultivated wildrice (*Zizania palustris*). *Plant Pathol.*, **2012**, *61(5)*, 903-914. https://doi.org/10.1111/j.1365-3059.2011.02581.x
88. Illnait-Zaragozí, M. T.; Martínez-Machín, G. F.; Fernández-Andreu, C. M.; Perurena-Lancha, M. R.; Theelen, B.; Boekhout, T.; Meis, J. F.; Klaassen, C. H. Environmental isolation and characterisation of *Cryptococcus* species from living trees in Havana city, Cuba. *Mycoses*, **2012**, *55*, e138-e144. https://doi.org/10.1111/j.1439-0507.2012.02168.x
89. Mukhopadhyay, K.; Haque, I.; Bandopadhyay, R.; Covert, S.; Porter, D. AFLP based assessment of genetic relationships among shiitake (*Lentinula* ssp.) mushrooms. *Mol. Biol. Rep.*, **2012**, *39(5)*, 6059-6065. https://doi.org/10.1007/s11033-011-1420-z
90. Pitt, W. M.; Bailey, K. L.; Fu, Y. B.; Peterson, G. W. Biological and genetic characterisation of *Phoma macrostoma* isolates with bioherbicidal activity. *Biocontrol Sci. Technol.*, **2012**, *22(7)*, 813-835. https://doi.org/10.1080/09583157.2012.691159
91. Ramírez-Camejo, L. A.; Zuluaga-Montero, A.; Lázaro-Escudero, M.; Hernández-Kendall, V.; Bayman, P. Phylogeography of the cosmopolitan fungus *Aspergillus flavus*: is everything everywhere?. *Fungal Biol.*, **2012**, *116*, 452-463. https://doi.org/10.1016/j.funbio.2012.01.006
92. Farahyar, S.; Zaini, F.; Kordbacheh, P.; Rezaie, S.; Safara, M.; Raoofian, R.; Heidari, M. Overexpression of aldo-keto-reductase in azole-resistant clinical isolates of Candida glabrata determined by cDNA-AFLP. *DARU J. Pharm. Sci.*, **2013**, *21(1)*, 1-7. https://doi.org/10.1186/2008-2231-21-1
93. Al-Daoude, A.; Shoaib, A.; Arabi, M. I. E.; Jawhar, M. Molecular variation among isolates of *Fusarium* species. *Bangladesh J. Bot.*, **2014**, *43(2)*, 207-212. https://doi.org/10.3329/bjb.v43i2.21674
94. Gong, T.; Shu, D.; Zhao, M.; Zhong, J.; Deng, H. Y.; Tan, H. Isolation of genes related to abscisic acid production in *Botrytis cinerea* TB-3-H8 by cDNA-AFLP. J. *Basic Microbiol.*, **2014**, *54*, 204-214. https://doi.org/10.1002/jobm.201200311
95. Piliponytė-Dzikienė, A.; Andriūnaitė, E.; Petraitienė, E.; Brazauskienė, I.; Statkevičiūtė, G.; Brazauskas, G. Genetic diversity and occurrence of *Leptosphaeria* spp. on *Brassica oleracea* and *B. napus* in Lithuania. *J. Plant Pathol.*, **2015**, *97(2)*,
96. López, S. C.; Theelen, B.; Manserra, S.; Issak, T. Y.; Rytioja, J.; Mäkelä, M. R.; De Vries, R. P. Functional diversity in *Dichomitus squalens* monokaryons. *IMA fungus*, **2017**, *8*, 17-25. https://doi.org/10.5598/imafungus.2017.08.01.02
97. Pawlik, A.; Janusz, G.; Dębska, I.; Siwulski, M.; Frąc, M.; Rogalski, J. Genetic and metabolic intraspecific biodiversity of *Ganoderma lucidum*. *BioMed Res. Int.*, **2015**, *2015*. https://doi.org/10.1155/2015/726149
98. Young, J. P. W. Genome diversity in arbuscular mycorrhizal fungi. *Curr. Op. Plant Biol.*, **2015**, *26*, 113-119. https://doi.org/10.1016/j.pbi.2015.06.005
99. Glynou, K.; Ali, T.; Kia, S. H.; Thines, M.; Maciá-Vicente, J. G. Genotypic diversity in root-endophytic fungi reflects efficient dispersal and environmental adaptation. *Mol. Ecol.*, **2017**, *26*, 4618-4630. https://doi.org/10.1111/mec.14231
100. van den Brink, J.; Samson, R. A.; Hagen, F.; Boekhout, T.; de Vries, R. P. Phylogeny of the industrial relevant, thermophilic genera *Myceliophthora* and *Corynascus*. *Fungal Divers.*, **2012**, *52(1)*, 197-207. https://doi.org/10.1007/s13225-011-0107-z
101. Pawlik, A.; Deptula, T.; Targonski, Z.; Rogalski, J.; Ohga, S. AFLP fingerprinting of *Tricoderma reesei* strains. *J. Fac. Agric. Kyushu Univ.*, **2012**, *57(1)*, 1-6. https://doi.org/10.5109/22041
102. Rola, B.; Pawlik, A.; Frąc, M.; Małek, W.; Targoński, Z.; Rogalski, J.; Janusz, G. The phenotypic and genomic diversity of *Aspergillus* strains producing glucose dehydrogenase. *Acta Biochim. Pol.*, **2015**, *62(4)*, 747–755. http://dx.doi.org/10.18388/abp.2015_1125

103. Okuda, Y.; Shimomura, N.; Funato, C.; Nagasawa, E.; Matsumoto, T. Genetic variation among natural isolates of the ectomycorrhizal hypogenous fungus, *Rhizopogon roseolus* from Japanese pine forests inferred using AFLP markers. *Mycoscience*, **2013**, *54(1)*, 13-18.
104. Cai, B.; Chen, J.; Zhang, Q.; Guo, L.; Huang, Y.; Wang, Z. DNA polymorphism difference between root system and rhizosphere soil arbuscular mycorrhizal fungi of Prunus mume by AFLP analysis. *Mycosystema*, **2015**, *34(6)*, 1118-1127. ISSN : 1672-6472
105. Marvasi, M.; Donnarumma, F.; Frandi, A.; Mastromei, G.; Sterflinger, K.; Tiano, P.; Perito, B. Black microcolonial fungi as deteriogens of two famous marble statues in Florence, Italy. *Int. Biodeterior. Biodegradation*, **2012**, *68*, 36-44. https://doi.org/10.1016/j.ibiod.2011.10.011

References: Supplementary Table S4

1. Abdel-Fatah, B. E.; Moharram, A. M.; Moubasher, A. E. D. A.; Al-Ryani, M. A. Genetic relationships and isozyme profile of dermatophytes and *Candida* strains from Egypt and Libya. *Afr. J. Biotechnol.*, **2013**, *12(29)*, 4554-4568. eISSN: 1684-5315
2. Lopes, R. B.; Mesquita, A. L. M.; Tigano, M. S.; Souza, D. A.; Martins, I.; Faria, M. Diversity of indigenous *Beauveria* and *Metarhizium* spp. in a commercial banana field and their virulence toward *Cosmopolites sordidus* (Coleoptera: Curculionidae). *Fungal Ecol.*, **2013**, *6(5)*, 356-364. https://doi.org/10.1016/j.funeco.2013.06.007
3. Luan, F.; Zhang, S.; Wang, B.; Huang, B.; Li, Z. Genetic diversity of the fungal pathogen *Metarhizium* spp., causing epizootics in Chinese burrower bugs in the Jingting Mountains, eastern China. *Mol. Biol. Rep.*, **2013**, *40(1)*, 515-523. https://doi.org/10.1007/s11033-012-2088-8
4. Gallou, A.; Serna-Domínguez, M. G.; Berlanga-Padilla, A. M.; Ayala-Zermeño, M. A.; Mellín-Rosas, M. A.; Montesinos-Matías, R.; Arredondo-Bernal, H. C. Species clarification of *Isaria* isolates used as biocontrol agents against *Diaphorina citri* (Hemiptera: Liviidae) in Mexico. *Fungal Biol.*, **2016**, *120(3)*, 414-423. https://doi.org/10.1016/j.funbio.2015.11.009
5. Tiago, P. V.; Medeiros, L. V.; Carneiro Leão, M. P.; Santos, A. C. D. S.; da Costa, A. F.; de Oliveira, N. T. Polymorphisms in entomopathogenic fusaria based on inter simple sequence repeats. *Biocontrol Sci. Technol.*, **2016**, *26(10)*, 1401-1410. https://doi.org/10.1080/09583157.2016.1210084
6. Zhang, S.; Chen, X.; Luan, F.; He, L.; Pu, S.; Li, Z. Genetic diversity and population structure of the Chinese fungus *Metarhizium rileyi* causing green muscardine in silkworm. *J. Invertebr. Pathol.*, **2016**, *140*, 16-24. https://doi.org/10.1016/j.jip.2016.08.005
7. Mitina, G., Kazartsev, I., Vasileva, A., Yli-Mattila, T. Multilocus genotyping based species identification of entomopathogenic fungi of the genus *Lecanicillium* (= *Verticillium lecanii sl*). *J. Basic Microbiol.*, **2017**, *57(11)*, 950-961. https://doi.org/10.1002/jobm.201700092
8. Toledo, A. V., Franco, M. E., Medina, R., de Remes Lenicov, A. M. M., Balatti, P. A. Assessment of the genetic diversity of Argentinean isolates of *Beauveria bassiana* (Ascomycota: Hypocreales) using ISSR markers. *J. King Saud Univ. Sci.*, **2019**, *31(4)*, 1264-1270. https://doi.org/10.1016/j.jksus.2018.02.006
9. Vianna, M. F., Pelizza, S., Russo, M. L., Toledo, A., Mourelos, C., Scorsetti, A. C. ISSR markers to explore entomopathogenic fungi genetic diversity: Implications for biological control of tobacco pests. *J. Biosci.*, **2020**, *45(1)*, 1-11. https://doi.org/10.1007/s12038-020-00108-4
10. Baioumy, A. A.; Swelim, H. H.; Ibrahim, A. A.; Mohamed, F. E. S.; Marzouk, A. S.; El-Alfy, S. H. Acaricidal and pathogenic effects of the entomopathogenic fungus *Beauveria bassiana* on engorged females of the fowl tick, *Argas persicus* (Argasidae). *Exp. Appl. Acarol.*, **2021**, *85(2)*, 331-354. https://doi.org/10.1007/s10493-021-00671-x
11. Abadio, A. K. R., Lima, S. S., Santana, M. F., Salomão, T. M. F., Sartorato, A., Mizubuti, E. S. G., Araújo, E. F.; de Queiroz, M. V. Genetic diversity analysis of isolates of the fungal bean pathogen *Pseudocercospora griseola* from central and southern Brazil. *Genet. Mol. Res.*, **2012**, *11*, 1272-1279
12. Arabi, M. I. E.; Nabulsi, I.; MirAli, N. Molecular phylogeny of *Pyrenophora graminea* as determined by RAPD and ISSR fingerprints. *J. Plant Biol. Res.*, **2012**, *1(2)*, 25-35. ISSN : 2233-0275
13. Mahdizadeh, V., Safaie, N., Goltapeh, E. M. Genetic diversity of sesame isolates of *Macrophomina phaseolina* using RAPD and ISSR markers. *Trakia J. Sci.*, **2012**, *10(2)*, 65-74. ISSN 1313-7050
14. Shen, W., Xi, P., Li, M., Liu, R., Sun, L., Jiang, Z., Zhang, L. Genetic diversity of *Ustilago scitaminea* Syd. in Southern China revealed by combined ISSR and RAPD analysis. *Afr. J. Biotechnol.*, **2012**,*11(54)*, 11693-11703. eISSN: 1684-5315
15. Yan, K.; Yin, Y.; Fu, C. E.; Guo, X.; Chen, Y. Genetic diversity among 21 *Inonotus obliquus* strains revealed by ISSR analysis. *Acta Edulis Fungi*, **2012**, *19(2)*, 26-30. ISSN : 1005-9873
16. Fu, D.; Zhuang, J.; Zhang, R.; Sun, G. Optimization of ISSR-PCR reaction system and genetic diversity analysis of *Colletotrichum* species causing bitter rot of apple. *Acta Phytophylacica Sinica*, **2013**, *40(3)*, 231-236. ISSN : 0577-7518
17. Martínez-Martínez, S.; Alarcón, A.; Urrieta-Velázquez, J. A.; Rojas-Martínez, R. I.; Ferrera-Cerrato, R.; Alvarado-Rosales, D. Detection of genetic variability of *Bremia lactucae* Regel. by RAPD and ISSR in lettuce plantations. Span. *J. Rural Dev.*, **2013**, *4(2)*, 19-30. ISSN : 2171-1216
18. Rampersad, S. N. Genetic structure of *Colletotrichum gloeosporioides sensu lato* isolates infecting papaya inferred by multilocus'' ISSR markers. *Phytopathol.*, **2013**, *103(2)*, 182-189. https://doi.org/10.1094/PHYTO-07-12-0160-R
19. Sserumaga, J. P.; Biruma, M.; Akwero, A.; Okori, P.; Edema, R. Genetic characterisation of Ugandan strains of *Colletotrichum sublineolum* using ISSR makers. *Uganda J. Agric. Sci.*, **2013**, *14(1)*, 111-123. eISSN: 2410-6909
20. Guimarães, T. S.; Barelli, M. A. A.; Corrêa, C. L.; da Silva, V. P.; Floriano, A. J. S.; Sander, N. L; ; da Silva, D. D. Genetic variability of *Colletotrichum sublineolum* through ISSR markers. *Res., Soc. Dev.*, **2021**, *10*, e20210212223. http://dx.doi.org/10.33448/rsd-v10i2.12223

21. Xu, Z., Ali, Z., Hou, X., Li, H., Yi, J. X., & Abbasi, P. A. Characterization of Chinese eggplant isolates of the fungal pathogen *Verticillium dahliae* from different geographic origins. *Gen. Mol. Res.*, **2013**, *12(1)*,183-195. http://dx.doi.org/10.4238/2013.January.24.11
22. Zheng, L.; Shi, F.; Hsiang, T. Genetic structure of a population of *Rhizoctonia solani* AG 2-2 IIIB from *Agrostis stolonifera* revealed by inter-simple sequence repeat (ISSR) markers. *Can. J. Plant Pathol.*, **2013**, *35(4)*, 476-481. https://doi.org/10.1080/07060661.2013.830148
23. Archana, B., Kini, K. R., Prakash, H. S. Genetic diversity and population structure among isolates of the brown spot fungus, *Bipolaris oryzae*, as revealed by inter-simple sequence repeats (ISSR). *Afr. J. Biotechnol*., **2014**, *13(2)*, 238-244. https://doi.org/10.5897/AJB2013.12063
24. Aylward, J.; Dreyer, L. L.; Steenkamp, E. T.; Wingfield, M. J.; Roets, F. Development of polymorphic microsatellite markers for the genetic characterisation of *Knoxdaviesia proteae* (Ascomycota: Microascales) using ISSR-PCR and pyrosequencing. *Mycol. Prog.*, **2014**, *13(2)*, 439-444. https://doi.org/10.1007/s11557-013-0951-1
25. Gramaje, D.; León, M.; Santana, M.; Crous, P. W.; Armengol, J. Multilocus ISSR markers reveal two major genetic groups in Spanish and South African populations of the grapevine fungal pathogen *Cadophora luteo-olivacea*. *PloS One*, **2014**, *9(10)*, e110417. https://doi.org/10.1371/journal.pone.0110417
26. Haghighi, M. T.; Shahdoust, E. Molecular analysis of genetic diversity of endophytic *Myrothecium* spp. by RAPD and ISSR markers. *Intl. J. Sci. Inven. Today*, **2014**, *3(6)*, 692-704. ISSN 2319 - 5436
27. Mahmodi, F.; Kadir, J. B.; Puteh, A.; Pourdad, S. S.; Nasehi, A.; Soleimani, N. Genetic diversity and differentiation of *Colletotrichum* spp. isolates associated with Leguminosae using multigene loci, RAPD and ISSR. *Plant Pathol. J.*, 2014, *30(1)*, 10. https://doi.org/10.5423/PPJ.OA.05.2013.0054
28. Mei, Z.; Yang, L.; Khan, M. A.; Yang, M.; Wei, C.; Yang, W.; ;Fu, J. Genotyping of *Ganoderma* species by improved random amplified polymorphic DNA (RAPD) and inter-simple sequence repeat (ISSR) analysis. *Biochem. Syst. Ecol.*, **2014**, *56*, 40-48. https://doi.org/10.1016/j.bse.2014.04.012
29. Shahnazi, S.; Meon, S.; Vadamalai, G.; Yazdani, D.; Shabanimofrad, M. Genetic Diversity of *Fusarium solani* isolates from black pepper (*Piper nigrum* L.) in Malaysia by ISSR marker. *Pertanika J. Trop. Agric. Sci.*, **2014**, *37(3)*, 363-374. ISSN: 1511-3701
30. Abbasi, K.; Abbasi, S.; Fotouhifar, K. B.; Zebarjadi, A. R. Study of genetic diversity in *Cytospora chrysosperma* isolates obtained from walnut trees in Iran using inter simple sequence repeat (ISSR) markers. *Arch. Phytopathol. Pflanzenschutz*, **2015**, *48(4)*, 327-335. https://doi.org/10.1080/03235408.2014.888235
31. Bagherabadi, S., Zafari, D., Soleimani, M. J. Genetic diversity of *Alternaria alternata* isolates causing potato brown leaf spot, using ISSR markers in Iran. *J. Plant Pathol. Microbiol.*, **2015**, *6(7)*, 1-6. https://doi.org/10.4172/2157-7471.1000286
32. Michalecka, M.; Bryk, H.; Poniatowska, A.; Seliga, P.; Puławska, J. Identification and characterization of *Neofabraea* fungi causing bull's eye rot on apple in Poland. *Acta Hortic.*, **2016**, *1144*, 183-188. https://doi.org/10.17660/ActaHortic.2016.1144.26
33. Michalecka, M.; Bryk, H.; Poniatowska, A.; Puławska, J. Identification of *Neofabraea* species causing bull's eye rot of apple in Poland and their direct detection in apple fruit using multiplex PCR. *Plant Pathol.*, **2016**, *65(4)*, 643-654. https://doi.org/10.1111/ppa.12449
34. Santiago, D. G. S.; Moctezuma, E. V.; Tovar, D. C. Molecular identification of the *Botryosphaeria* sp. complex related to cankers and drying of buds in *Eucalyptus* sp. *Rev. Mex. Cienc. Forestales*, *2015*, *6(32)*, 93-106. ISSN 2007-1132
35. Yugander, A.; Ladhalakshmi, D.; Prakasham, V.; Mangrauthia, S. K.; Prasad, M. S.; Krishnaveni, D.; Madhav, M. S.; Sundaram, R. M.; Laha, G. S. Pathogenic and genetic variation among the isolates of *Rhizoctonia solani* (AG 1-IA), the rice sheath blight pathogen. *J. Phytopathol*., **2015**, *163(6)*, 465-474. https://doi.org/10.1111/jph.12343
36. Kandan, A.; Akhtar, J.; Singh, B.; Pal, D.; Chand, D.; Rajkumar, S.; Agarwal, P. C. Genetic diversity analysis of fungal pathogen *Bipolaris sorghicola* infecting *Sorghum bicolor* in India. *J. Environ. Biol.*, **2016**, *37(6)*, 1323. ISSN: 0254-8704
37. Piyaboon, O., Unartngam, A., Unartngam, J. Genetic relationships of *Myrothecium roridum* isolated from water hyacinth in Thailand using ISSR markers and ITS sequence analysis. *J. Agric. Technol.*, **2016**, *12(4)*, 643-655. ISSN 1686-9141
38. Poniatowska, A.; Michalecka, M.; Puławska, J. Genetic diversity and pathogenicity of *Monilinia polystroma* – the new pathogen of cherries. *Plant Pathol.*, **2016**, *65(5)*, 723-733. https://doi.org/10.1111/ppa.12463
39. Poonkothai, M. Diversity of *Fusarium* spp associated with sugarcane wilt. History, **2015**, *15(47)*, 149-163. ISSN 2319 – 7730
40. Yamada, K.; Sonoda, R.; Ishikawa, K. Population genetic structure of QoI-resistant *Pestalotiopsis longiseta* isolates causing tea gray blight. *Plant Dis.*, **2016**, *100(8)*, 1686-1691. https://doi.org/10.1094/PDIS-09-15-1114-RE
41. Azimi, M.; Rezaee, S.; Baigi, S. Genetic diversity of *Fusarium oxysporum f.sp. ciceri*, the causal agent of Iranian chickpea vascular wilt in ILAM Province using ISSR markers. **2017**, *5(2)*, 39-54.
42. Montakhabi, M. K.; Bonjar, G. H. S.; Talebi, R. Genetic diversity and population structure of Iranian isolates of *Fusarium oxysporum f. sp. ciceris*, the causal agent of chickpea wilt, using ISSR and DAMD-PCR markers. *Exp. Biol.*, **2018**, *16*, 291-298. https://doi.org/10.22364/eeb.16.20
43. Kang, L.; Cui, H.; Zhang, Y.; Zhang, Z.; Yu, X.; Ye, Z. Identification and ISSR polymorphism analysis of *Ustilago esculenta* isolated from *Zizania latifolia*. *Mycosystema*, **2017**, *36(9)*, 1210-1221. ISSN : 1672-6472
44. Khazaeli, P.; Rezaee, S.; Mirabolfathy, M.; Zamanizadeh, H.; Kiadaliri, H. Genetic and phenotypic variation of *Calonectria pseudonaviculata* isolates causing boxwood blight disease in the Hyrcanian forest of Iran. *Agric. Res. Technol. Open Access J.*, **2018**, *19(1)*, 556081. https://doi.org/10.19080/ARTOAJ.2018.19.556081
45. Shanjani, P. S.; Javadi, H.; Rasoulzadeh, L.; Amirkhani, M. Evaluation of genetic differentiation among healthy and infected *Buxus hyrcana* with boxwood blight using RAPD and ISSR markers. *N. Z. J. For. Sci.*, **2018**, *48(1)*, 1-10. https://doi.org/10.1186/s40490-018-0120-z

46. Michalecka, M.; Bryk, H.; Seliga, P. Identification and characterization of *Diaporthe vaccinii* Shear causing upright dieback and viscid rot of cranberry in Poland. *Eur. J. Plant Pathol.*, **2017**, *148(3)*, 595-605. https://doi.org/10.1007/s10658-016-1114-4
47. Nawade, B.; Talaviya, J. R.; Vyas, U. M.; Jadeja, K. B.; Golakiya, B. A. Diversity analysis among *Fusarium oxysporum f. sp. cumini* isolates using ISSR markers, spore morphology and pathogenicity. *Int. J. Curr. Microbiol. Appl. Sci.*, **2017**, *6*, 79-87. https://doi.org/10.20546/ijcmas.2017.604.010
48. Parthasarathy, S.; Muthamilan, M.; Harish, S.; Alice, D.; Raguchander, T. Natural incidence and genetic variability of *Erysiphe pisi*, the causal agent of powdery mildew on peas in the Nilgiris district, Tamil Nadu, India. *Curr. Appl. Sci. Technol.*, **2017**, *245*, 1-11. https://doi.org/10.9734/CJAST/2017/37933
49. Yang, C. L., Wu, X. P., Chen, B., Deng, S. S., Chen, Z. E., Huang, Y. Y., Jin, S. S. Comparative analysis of genetic polymorphisms among *Monascus* strains by ISSR and RAPD markers. *J. Sci. Food Agric.*, **2017**, *97(2)*, 636-640. https://doi.org/10.1002/jsfa.7780
50. Anand, G., Kapoor, R. Population structure and virulence analysis of *Alternaria carthami* isolates of India using ISSR and SSR markers. *World J. Microbiol. Biotechnol.*, **2018**, *34(9)*, 1-14. https://doi.org/10.1007/s11274-018-2524-6
51. Dalvand, M., Zafari, D., Soleimani Pari, M. J., Roohparvar, R., Tabib Ghafari, S. M. Studying genetic diversity in *Zymoseptoria tritici*, causal agent of *Septoria tritici* blotch, by using ISSR and SSR markers. *J. Agric. Sci. Technol.*, **2018**, *20(6)*, 1307-1316.
52. Hamdi, N.; Benfradj, N.; Salem, I. B.; Abad-Campos, P. Genetic diversity of *Fusarium solani f. sp. cucurbitae* the causal agent of crown and root rot of watermelon in Tunisia using ISSR markers. *NRMJ*, **2019**, *3(1)*, 271-280. https://doi.org/10.21608/NRMJ.2019.28113
53. Luo, Q. Genetic diversity of endophytic fungi strains *Fusarium proliferatum* isolated from *Belamcanda chinensis* using ISSR and SRAP markers. *Zhong Cao Yao*, **2019**, 5847-5857
54. Salahlou, R.; Safaie, N.; Shams-Bakhsh, M. Using ISSR and URP-PCR markers in detecting genetic diversity among *Macrophomina phaseolina* isolates of sesame in Iran. *J. Crop Prot.*, **2019**, *8(3)*, 293-309.
55. Sushma, S.; Shanmugam, V.; Singh, B. G.; Neelam, T.; Sapna, T.; Priyanka, T., Sumitra, P.; Nath, Y. A. Genetic diversity and phylogenetic profiling of *Fusarium* sp., the causing storage rot of ginger (*Zingiber officinale*) in Himachal Pradesh and their potential environmental eco-friendly management strategies. *Res. J. Biotechnol.*, **2019**, *14*.
56. Upadhyay, P.; Ganaie, S. H.; Singh, N. Diversity assessment among *Alternaria solani* isolates causing early blight of tomato in India. *Proc. Natl. Acad. Sci. India Sect. B Biol. Sci.*, **2019**, *89(3)*, 987-997. https://doi.org/10.1007/s40011-018-1017-6
57. Ul Haq, I.; Ijaz, S. Assessment of genetic diversity based on ISSR markers in *Neopestalotiosis* species collected from guava (*Psidium guajava* L.) plants affected with canker disease in Pakistan. *Appl. Ecol. Environ. Res.*, **2019**, *17(5)*, 11803-11811. : http://dx.doi.org/10.15666/aeer/1705_1180311811
58. Xie, H. H.; Wei, J. G.; Huang, R. S.; Yang, X. B. Genetic diversity analyses of *Lasiodiplodia theobromae* on *Morus alba* and *Agave sisalana* based on RAPD and ISSR molecular markers. *Mycology*, **2016**, *7(4)*, 155-164. https://doi.org/10.1080/21501203.2016.1232762
59. Xie, H.; Wang, L.; Huang, X.; Li, J. ISSR analysis of genetic diversity for mulberry *Lasiodiplodia* root rot pathogen. *South. J. Agric.*, **2019**, *50(7)*, 1505-1512. ISSN : 2095-1191
60. Yao, Z.; Qin, D.; Chen, D.; Liu, C.; Chen, W.; Liu, T.; Chen W.; Liu, T.; Liu, B.; Gao, L. Development of ISSR-derived SCAR marker and SYBR green I real-time PCR method for detection of teliospores of *Tilletia laevis* Kühn. *Sci. Rep.*, **2019**, *9(1)*, 1-7. https://doi.org/10.1038/s41598-019-54163-5
61. Bhatt, P.; Rakhashiya, P.; Thaker, V. Molecular marker development from ISSR for fungal pathogens of *Mangifera indica* L. *Indian Phytopathol.*, **2020**, *73(2)*, 257-265. https://doi.org/10.1007/s42360-020-00211-4
62. Nguyen, D. Q.; Li, H.; Tran, T. T.; Sivasithamparam, K.; Jones, M. G. K.; Wylie, S. J. Four *Tulasnella* taxa associated with populations of the Australian evergreen terrestrial orchid *Cryptostylis ovata*. *Fungal Biol.*, **2020**, *124(1)*, 24-33. https://doi.org/10.1016/j.funbio.2019.10.006
63. Das, A.; Roy, B.; Jangra, S.; Chowdhury, A.; Kamil, D.; Devi, T. P. Analysis of genetic diversity of *Colletotrichum* population causing anthracnose in fruit crops using ISSR markers. *Indian Phytopathol.*, **2021**, *74(1)*, 69-80. https://doi.org/10.1007/s42360-020-00295-y
64. Deewong, C.; Thamthurasan, W.; Unartngam, J. Evaluation of genetic variation of *Sarocladium oryzae* causal agent of rice sheath rot disease based on ISSR markers. *J. Agric. Sci.*, **2021. ISSN : 2697-5378**
65. Natino, W.; Thanyasiriwat, T.; Sangdee, A.; Boonruangrod, R.; Kawicha, P. Detection of genetic variation of *Fusarium oxysporum f. sp. cubense*, the causal agent of fusarium wilt in banana by ISSR markers. *Songklanakarin Journal of Plant Science*, **2021**, *8(2)*, 97-104. ISSN: 2351-0846
66. Naveen, J.; Navya, H. M.; Hithamani, G.; Hariprasad, P.; Niranjana, S. R. Pathological, biochemical and molecular variability of *Colletotrichum truncatum* incitant of anthracnose disease in chilli (*Capsicum annuum* L.). *Microb. Pathog.*, **2021**, *152*, 104611. https://doi.org/10.1016/j.micpath.2020.104611
67. Quoc, N. B., Trang, H. T. T., Phuong, N. D. N., Chau, N. N. B., Jantasuriyarat, C. Development of a SCAR marker linked to fungal pathogenicity of rice blast fungus *Magnaporthe oryzae*. *Int. J. Microbiol.*, **2021**, *24(2)*, 149-156. https://doi.org/10.1007/s10123-020-00150-0
68. Rajput, L. S.; Kumar, S.; Bhati, H.; Nair, K.; Akhtar, J.; Kumar, P.; Dubey, S. C. Diversity assessment of indigenous and exotic Diaporthe species associated with various crops using ISSR, URP and SRAP markers. *Indian Phytopathol.*, **2021**, *74(3)*, 615-624. https://doi.org/10.1007/s42360-020-00313-z
69. Bincader, S.; Pongpisutta, R.; Rattanakreetakul, C. Diversity of *Colletotrichum* species causing anthracnose disease from mango cv. Nam Dork Mai See Tong based on ISSR-PCR. *Indian J. Agric. Res.*, **2022**, *56(1)*, 81-90. https://doi.org/10.18805/IJARe.AF-691

70. Sun, L.; Pei, K.; Zhang, Y.; Zhao, J.; Yang, G.; Qin, G.; Song, Y.; Song, R. Genetic diversity of *Armillaria gallica* isolates from China and Europe revealed with ISSR analysis. *Sheng Wu Duo Yang Xing (Biodiversity Science)*, **2012**, *20(2)*, 224. https://doi.org/10.3724/SP.J.1003.2012.07214
71. Hu, Z.; Zhao, L.; Kong, B.; Chen, H.; Fan, J.; Liu, F.; Li, X; Cai, H.; Yang, G; Qin X.; Fang, D. Comparison and analysis of molecular methods ISSR and RAPD used for assessment of genetic diversity in *Alternaria alternata*. *Southwest China Journal of Agricultural Sciences*, **2012**, *25(3)*, 917-921. ISSN 1001 - 4829
72. Lin, Y.; Fuping, L.; Shanshan, L.; Xiao, W.; Ruixuan, Z.; Ziqin, L.; Hui, Z. ISSR analysis of *Fusarium oxysporum* Schl. in Hebei province. *Procedia Environ. Sci.*, **2012**, *12*, 1237-1242. https://doi.org/10.1016/j.proenv.2012.01.414
73. Villarino, M.; Larena, I.; Martinez, F.; Melgarejo, P.; De Cal, A. Analysis of genetic diversity in *Monilinia fructicola* from the Ebro Valley in Spain using ISSR and RAPD markers. *Eur. J. Plant Pathol.*, **2012**, *132(4)*, 511-524. https://doi.org/10.1007/s10658-011-9895-y
74. Gawande, S. P.; Borkar, S. G.; Chimote, V. P.; Sharma, A. K. Determination of genetic diversity in *Sclerotium rolfsii* and *Sclerotium delphinii* by using RAPD and ISSR markers. *Vegetos*, **2013**, *26(2s)*, 39-44. https://doi.org/10.5958/j.2229-4473.26.2s.118
75. Queiroz, C. B.; Miranda, E. C.; Hanada, E.; Sousa, N. R.; Gasparotto, L.; Soares, M. A.; Silva, G. F. (2013). Distribution of mating-type alleles and M13 PCR markers in the black leaf spot fungus *Mycosphaerella fijiensis* of bananas in Brazil. *Genet. Mol. Res., 2013*, *12*, 443-452. https://doi.org/10.4238/2013.February.8.8
76. Qiu, C.; Yan, W.; Li, P.; Deng, W.; Song, B.; Li, T. Evaluation of growth characteristics and genetic diversity of commercial and stored lines of *Hypsizygus marmoreus*. *Int. J. Agric. Biol.*, **2013**, *15(3)*. ISSN: 1560-8530
77. Zhao, M.; Chen, Q.; Zhang, J.; Wu, X.; Huang, C. Comparison studies of genetic diversity of *Pleurotus eryngii var. tuoliensis* by IGS2-RFLP, SCoT and ISSR markers. *Mycosystema*, **2013**, *32(4)*, 682-689. ISSN : 1672-6472
78. Abdulateef, S. M.; Aljubori, M. H.; Abdulbaqi, N. J. Genetic diversity among some *Aspergillus flavus* isolates by using Inter simple sequence repeats (ISSR). *Iraqi J. Sci.*, **2014**, *55(3)*, 986-993
79. Palmero, D.; Rubio-Moraga, A.; Galvez-Patón, L.; Nogueras, J.; Abato, C.; Gómez-Gómez, L.; Ahrazem, O. Pathogenicity and genetic diversity of *Fusarium oxysporum* isolates from corms of *Crocus sativus*. *Ind. Crops Prod.*, **2014**, *61*, 186-192. https://doi.org/10.1016/j.indcrop.2014.06.051
80. Wang, Q.; Wu, S.; Wei, S.; Wang, C. Genetic diversity of *Tricholoma lobayense* revealed by ISSR analysis. *Southwest China Journal of Agricultural Sciences*, **2014**, *27(2)*, 768-771. ISSN : 1001-4829
81. Xu, L.; Lu, Y.; You, Q.; Liu, X.; Grisham, M. P.; Pan, Y.; Que, Y. Biogeographical variation and population genetic structure of *Sporisorium scitamineum* in Mainland China: Insights from ISSR and SP-SRAP markers. *Sci. World*, **2014**, *2014*. https://doi.org/10.1155/2014/296020
82. Aiyaz, M.; Divakara, S. T.; Parthasarathy, S.; Hariprasad, P.; Nayaka, S. C.; Niranjana, S. R. Genetic diversity and biocontrol potential of rhizospheric microbes isolated from tomato and maize. *J. Pure Appl. Microbiol.*, **2015**, *9*, 239-248. ISSN: 0973-7510
83. ElSharawy, A. A.; Yang, G.; Hu, X.; Yang, J. Genetic relationships between virulence, vegetative compatibility and ISSR marker of *Verticillium dahliae* isolated from cotton. *Arch. Phytopathol. Pflanzenschutz*, **2015**, *48(8)*, 646-663. https://doi.org/10.1080/03235408.2015.1091164
84. Liu, N.; Liu, Z. L.; Gong, G.; Zhang, M.; Wang, X.; Zhou, Y.; Qi, X.; Chen, H.; Yang, J.; Luo, P. ;Yang, C. Virulence structure of *Blumeria graminis f. sp. tritici* and its genetic diversity by ISSR and SRAP profiling analyses. *PloS One*, **2015**, *10(6)*, e0130881. https://doi.org/10.1371/journal.pone.0130881
85. Qi, S. S.; Fan, Y.; Gong, Z. N.; Yan, S. Z.; Zhao, B. T.; Chen, S. L. Genetic diversity of *Shiraia bambusicola* from East China assessed using ISSR markers. *Biochem. Syst. Ecol.*, **2015**, *59*, 239-245. https://doi.org/10.1016/j.bse.2015.01.007
86. Rassin, N. K.; Nemat; J. A.; Dheeb, B. I. Molecular identification of *Aspergillus fumigatus* using ISSR and RAPD Markers. *Iraqi J. Sci.*, **2015**, *56(4A)*, 2788-2797.
87. Renu, T.; Pooja, K.; Sharma, P. N.; Sharma, B. M. Assessment of genetic diversity in *Ganoderma lucidum* using RAPD and ISSR markers. *Indian Phytopathol.*, **2015**, *68(3)*, 316-320. ISSN : 0367-973X
88. Abed-Ashtiani, F.; Kadir, J.; Nasehi, A.; Hashemian-Rahaghi, S. R.; Vadamalai, G.; Rambe, S. K. Characterisation of *Magnaporthe oryzae* isolates from rice in peninsular Malaysia. *Czech J. Genet. Plant Breed.*, **2016**, *52(4)*, 145-156. https://doi.org/10.17221/102/2016-CJGPB
89. Albayrak, G.; Yörük, E.; Gazdağli, A.; Sharifnabi, B. Genetic diversity among *Fusarium graminearum* and *F. culmorum* isolates based on ISSR markers. *Arch. Biol. Sci.*, **2016**, *68(2)*, 333-343. https://doi.org/10.2298/ABS150630025A
90. Fedrigo, K.; Giacomin, R. M.; Faria, C. M.; Da-Silva, P. R. ISSR primers for analysis of genetic variability of *Stenocarpella maydis*. *Trop. Plant Pathol.*, **2016**, *41(4)*, 270-275. https://doi.org/10.1007/s40858-016-0089-1
91. Mahmoud, M. A.; El-Samawaty, A. M. A.; Yassin, M. A.; Abd El-Aziz, A. R. M. Genetic diversity analysis of *Aspergillus flavus* isolates from plants and air by ISSR markers. *Genet. Mol. Res.*, **2016**, *15(2)*, 1-9. http://dx.doi.org/10.4238/gmr.15028081
92. Parreira, D. F.; Zambolim, L.; Gomes, E. A.; da Costa, R. V.; da Silva, D. D.; Lana, U. D. P.; Neves, W. S.; Figueiredo, J. E. F.; Cota, L. V. Genetic diversity estimated through ISSR markers of *Colletotrichum graminicola* in Brazil. *Rev. Bras. Milho Sorgo*, **2016**, *15(2)*, 186-194. http://dx.doi.org/10.18512/1980-6477/rbms.v15n2p186-194
93. Velez, P.; Quintero, C. A.; Merino, G.; Gasca-Pineda, J.; González, M. C. An ISSR-based approach to assess genetic diversity in the marine arenicolous fungus *Corollospora maritima sensu lato*. *Mycoscience*, **2016**, *57(3)*, 187-195. https://doi.org/10.1016/j.myc.2016.01.002
94. Jebaraj, M. D.; Aiyanathan, K. E. A.; Nakkeeran, S. Virulence and genetic diversity of *Sclerotium rolfsii* Sacc., infecting groundnut using nuclear (RAPD & ISSR) markers. *J. Environ. Biol.*, **2017**, *38(1)*, 147.

95. Altinok, H. H.; Can, C.; Altinok, M. A. Characterization of *Fusarium oxysporum f. sp. melongenae* isolates from Turkey with ISSR markers and DNA sequence analyses. *Eur. J. Plant Pathol.*, **2018**, *150(3)*, 609-621. https://doi.org/10.1007/s10658-017-1305-7
96. Kumar, P.; Akhtar, J.; Kandan, A.; Singh, B.; Kiran, R.; Nair, K.; Dubey, S. C. Efficacy of URP and ISSR markers to determine diversity of indigenous and exotic isolates of *Curvularia lunata*. *Indian Phytopathol.*, **2018**, *71(2)*, 235-242. https://doi.org/10.1007/s42360-018-0038-7
97. Salim, R. G.; Aly, S. E. S.; Abo-Sereh, N. A.; Hathout, A. S.; Sabry, B. A. Molecular identification and Inter-Simple Sequence Repeat (ISSR) differentiation of toxigenic *Aspergillus* strains. *Jordan J. Biol. Sci.*, **2019**, *12(5)*, 609 – 616. ISSN 1995-6673
98. Longya, A.; Talumphai, S.; Jantasuriyarat, C. Morphological characterization and genetic diversity of rice blast fungus, *Pyricularia oryzae*, from Thailand using ISSR and SRAP markers. *J. Fungi*, **2020**, *6(1)*, 38. https://doi.org/10.3390/jof6010038
99. Mazrou, Y. S.; Makhlouf, A. H.; Elseehy, M. M.; Awad, M. F.; Hassan, M. M. Antagonistic activity and molecular characterization of biological control agent *Trichoderma harzianum* from Saudi Arabia. *Egypt. J. Biol. Pest Control*, **2020**, *30(1)*, 1-8. https://doi.org/10.1186/s41938-020-0207-8
100. Mehri, Z.; Khodaparast, S. A.; Aalami, A.; Mousanejad, S. Genetic diversity of *Athelia rolfsii* populations in the north of Iran. *Rostaniha*, **2020**, *21(1)*, 14-26. https://dx.doi.org/10.22092/botany.2020.127939.1175
101. Fekrikohan, S.; Sharifnabi, B.; Javan-Nikkhah, M. Genetic diversity of *Botrytis cinerea* isolates in different plant hosts and localities using ISSR molecular markers. *Mycol. Iran.*, **2021**, *8(1)*, 59-68. https://dx.doi.org/10.22043/MI.2022.357601.1209
102. Du, P.; Cui, B. K.; Dai, Y. C. Assessment of genetic diversity among wild *Auricularia polytricha* populations in China using ISSR markers. *Cryptogam. Mycol.*, **2012**, *33(2)*, 191-201. https://doi.org/10.7872/crym.v33.iss2.2012.191
103. Wang, S.; Yin, Y.; Liu, Y.; Xu, F. Evaluation of genetic diversity among Chinese *Pleurotus eryngii* cultivars by combined RAPD/ISSR marker. *Curr. Microbiol.*, **2012**, *65(4)*, 424-431. https://doi.org/10.1007/s00284-012-0177-5
104. Yang, H.; Su, W.; Tan, Y.; Qin, Y.; Ma, T.; Pu, H.; Zhou, Z. Germplasm evaluation of *Pleurotus eryngii* based on ISSR markers. *Southwest China Journal of Agricultural Sciences*, **2019**, *32(7)*, 1480-1486. ISSN: 1001-4829
105. Du, P.; Cui, B. K.; Zhang, C. F.; Dai, Y. C. Genetic diversity of wild *Auricularia auricula-judae* revealed by ISSR analysis. *Biochem. Syst. Ecol.*, **2013**, *48*, 199-205. https://doi.org/10.1016/j.bse.2012.11.011
106. Prasad, M. P.; Agarwal, K. DNA fingerprinting of commercial mushrooms by ISSR and SSR markers for genetic discrimination. *Int. J. Pharma Bio Sci.*, **2013**, *4(4)*. ISSN: 0975-6299
107. Divakara, S. T.; Santosh, P.; Aiyaz, M.; Venkata Ramana, M.; Hariprasad, P.; Nayaka, S. C.; Niranjana, S. R. Molecular identification and characterization of *Fusarium* spp. associated with sorghum seeds. *J. Sci. Food Agric.*, **2014**, *94(6)*, 1132-1139. https://doi.org/10.1002/jsfa.6380
108. Mahmoud, M. A.; El-Zaidy, M.; Al-Othman, M. R.; Abd El-Aziz, A. R.; Al-Wadai, A. S. Molecular characterization of *Aspergillus flavus* contamination of barley grains using RAPD and ISSR. *J. Pure Appl. Microbiol.*, **2014**, *8*, 77-85. https://doi.org/10.4238/2013.September.3.10
109. Mahmoud, M. A.; Ali, H. M.; El-Aziz, A. R. M.; Al-Othman, M. R.; Al-Wadai, A. S. Molecular characterization of aflatoxigenic and non-aflatoxigenic *Aspergillus flavus* isolates collected from corn grains. *Genet. Mol. Res.*, **2014**, *13(4)*, https://doi.org/9352-9370. 10.4238/2014.November.11.2
110. Malekzadeh, K.; Mohsenifard, E.; Jalalzadeh, M. S. B.; Farsi, M. Identification and strain-typing of button mushroom using ISSR, ITS and IGS markers, *Mod. Genet.*, **2014**, *9*, 343-352
111. Priyanka, S. R.; Uppalapati, S. R.; Kingston, J. J.; Murali, H. S.; Batra, H. V. Development of ISSR-derived SCAR marker-targeted PCR for identification of *Aspergillus* section Flavi members. *Lett. Appl. Microbiol.*, **2014**, *58(5)*, https://doi.org/414-422. 10.1111/lam.12207
112. Sahoo, A. K.; Mohapatra, K. B.; Behera, B. B.; Jadhao, K. R.; Rout, G. R. Phylogenetic analysis of high yielding strain of paddy straw mushroom (*Volvariella volvacea*) by using ISSR markers. *Middle East J. Sci. Res.*, **2014**, *21(8)*, 1197-1202. https://doi.org/10.5829/idosi.mejsr.2014.21.08.21639
113. Yin, Y.; Liu, Y.; Li, H.; Zhao, S.; Wang, S.; Liu, Y.; Wu, D.; Xu, F. Genetic diversity of *Pleurotus pulmonarius* revealed by RAPD, ISSR, and SRAP fingerprinting. *Curr. Microbiol.*, **2014**, *68*, 397-403. https://doi.org/10.1007/s00284-013-0489-0
114. Liu, M. M.; Xing, Y. M.; Guo, S. X. Diversity analysis of *Polyporus umbellatus* in China using inter-simple sequence repeat (ISSR) markers. *Biol. Pharm. Bull.*, **2015**, *38(10)*, 1512-1517. https://doi.org/10.1248/bpb.b15-00274
115. Gomes-Silva, F.; Almeida, C.; Silva, A. G.; Leão, M. P.; Silva, K. P.; Oliveira, L. G.; Silva, M. V.; Costa, A. F.; Lima, V. Genetic diversity of isolates of *Macrophomina phaseolina* associated with cowpea from Brazil semi-arid region. *J. Agric. Sci.*, **2017**, *9(11)*, 112-116. https://doi.org/10.5539/jas.v9n11p112
116. Mahmoud, M. A.; Shehata, S. M. Molecular identification and characterization of *Fusarium* spp. associated with wheat grains. *Int. J. Adv. Res. Biol. Sci.*, **2017**, *4*, 77-87. https://doi.org/10.22192/ijarbs
117. Popa, G.; Cuica, M.; Cornea, C. P.; Nicolcioiu, B., Toma, R. Evaluation of the genetic diversity of *Pleurotus ostreatus* strains using combinatorial tubulin based polymorphism (cTBP) and ISSR markers. *Scientific Bulletin. Series F. Biotechnologies*, **2017**, *21*. ISSN: 2285-1364
118. Yang, C. L.; Wu, X. P.; Chen, B.; Deng, S. S.; Chen, Z. E.; Huang, Y. Y.; Jin, S. S. Comparative analysis of genetic polymorphisms among *Monascus* strains by ISSR and RAPD markers. *J. Sci. Food Agric.*, **2017**, *97(2)*, 636-640. https://doi.org/10.1002/jsfa.7780
119. Du, J.; Guo, H. B.; Li, Q.; Forsythe, A.; Chen, X. H.; Yu, X. D. Genetic diversity of *Lepista nuda* (Agaricales, Basidiomycota) in Northeast China as indicated by SRAP and ISSR markers. *PLoS One*, **2018**, *13(8)*, e0202761. https://doi.org/10.1371/journal.pone.0202761

120. Patel, P.; Rajkumar, B. K.; Parmar, P.; Shah, R.; Krishnamurthy, R. Assessment of genetic diversity in *Colletotrichum falcatum* Went accessions based on RAPD and ISSR markers. *J. Genet. Eng. Biotechnol.*, **2018**, *16*, 153-159. https://doi.org/10.1016/j.jgeb.2017.11.006
121. Mohammadi, A.; Bahramikia, S. Molecular identification and genetic variation of *Alternaria* species isolated from tomatoes using ITS1 sequencing and inter simple sequence repeat methods. *Curr. Med. Mycol.*, **2019**, *5(2)*, 1. https://doi.org/10.18502/cmm.5.2.1154
122. Wang, Y.; Wan, L.; Huang, C.; Han, J.; Li, J.; Yang, P.; QiAng, Y; ZhiYaun, G.; Xie, H. Genetic diversity of *Grifola frondosa* based on integrated ISSR and SRAP analysis. *Acta Edulis Fungi*, **2019**, *26(3)*, 26-36. ISSN : 1005-9873
123. Yang, Y. Q.; Sun, Q.; Li, C. M.; Chen, H. F.; Zhao, F.; Huang, J. H.; Zhou, J. S.; Lan, B. Biological characteristics and genetic diversity of *Phomopsis asparagi*, causal agent of asparagus stem blight. *Plant Dis.*, **2020**, *104(11)*, 2898-2904. https://doi.org/10.1094/PDIS-07-19-1484-RE
124. Estaji, A.; Khazaei, Z. Assessment of genetic diversity in some of black and brown Iranian truffles by ISSR markers. *J. Plant Mol. Breed.*, **2021**. https://doi.org/10.22058/JPMB.2021.531465.1235
125. Sun, Q.; Wei, W.; Zhu, W.; Zhang, S.; Song, J.; Zheng, Y.; Chen, P. Genetic diversity of Chinese *Wolfiporia cocos* cultivars revealed by phenotypic traits and ISSR markers. *Mycol. Prog.*, **2015**, *14(8)*, 1-7. https://doi.org/10.1007/s11557-015-1088-1
126. Wang, W.; Yuan, W.; Lu, N.; Song, J.; Yan, J. Genetic diversity of wild *Sanghuangporus sanghuang* based on ISSR molecular markers. *Acta Agriculturae Zhejiangensis*, **2019**, *31(3)*, 414-419. https://doi.org/10.3969/j.issn.1004-1524.2019.03.10
127. He, L.; Yao, K.; Xu, X.; Li, F.; Luo, X. Intraspecific identification of Lingzhi Medicinal Mushroom, *Ganoderma lingzhi* (Agaricomycetes), using ISSR markers. Int. J. Med. Mushrooms, **2022**, *24(4)*, 43-52. https://doi.org/10.1615/IntJMedMushrooms.2022043141
128. Zhang, C. S.; Xing, F. G.; Selvaraj, J. N.; Yang, Q. L.; Zhou, L.; Zhao, Y. J.; Liu, Y. The effectiveness of ISSR profiling for studying genetic diversity of *Aspergillus flavus* from peanut-cropped soils in China. *Biochem. Syst. Ecol.*, **2013**, *50*, 147-153. https://doi.org/10.1016/j.bse.2013.03.046
129. Shahid, M.; Singh, A.; Srivastava, M.; Srivastava, D. K. Molecular characterization of *Trichoderma viride* isolated from rhizospheric soils of Uttar Pradesh based on rDNA markers and analysis of their PCR-ISSR profiles. *J. Mol .Biomark. Diagn.*, **2014**, *5(170)*, 2. https://doi.org/10.4172/2155-9929.1000170
130. Garrido-Jurado, I.; Fernández-Bravo, M.; Campos, C.; Quesada-Moraga, E. Diversity of entomopathogenic Hypocreales in soil and phylloplanes of five Mediterranean cropping systems. *J. Invertebr. Pathol.*, **2015**, *130*, 97-106. https://doi.org/10.1016/j.jip.2015.06.001

References: Supplementary Table S5

1. Amouri, I.; Sellami, H.; Abbes, S.; Hadrich, I.; Mahfoudh, N.; Makni, H.; Ayadi, A. Microsatellite analysis of *Candida* isolates from recurrent vulvovaginal candidiasis. *J. Med. Microbiol.*, **2012**, *61(8)*, 1091-1096. https://doi.org/10.1099/jmm.0.043992-0
2. Freire, A. K. L.; dos Santos Bentes, A.; de Lima Sampaio, I.; Matsuura, A. B. J.; Ogusku, M. M.; Salem, J. I.; Wanke, B.; de Souza, J. V. B. Molecular characterisation of the causative agents of cyptococcosis in patients of a tertiary healthcare facility in the state of Amazonas-Brazil. *Mycoses*, **2012**, *55(3)*, e145-e150. https://doi.org/10.1111/j.1439-0507.2012.02173.x
3. Pan, W.; Khayhan, K.; Hagen, F.; Wahyuningsih, R.; Chakrabarti, A.; Chowdhary, A.; Ikeda, R.; Taj-Aldeen, S. J.; Khan, Z.; Imran, D.; Sjam, R.; Sriburee, P.; Liao, W.; Chaicumpar, K.; Ingviya, N.; Mouton, J. W.; Curfs-Breuker, I.; Boekhout, T.; Meis, J. F.; Klaassen, C. H. Resistance of Asian *Cryptococcus neoformans* serotype A is confined to few microsatellite genotypes. *PloS One*, **2012**, *7(3)*, e32868. https://doi.org/10.1371/journal.pone.0032868
4. Pulcrano, G.; Roscetto, E.; Iula, V. D.; Panellis, D.; Rossano, F.; Catania, M. R. MALDI-TOF mass spectrometry and microsatellite markers to evaluate *Candida parapsilosis* transmission in neonatal intensive care units. *Eur. J. Clin. Microbiol. Infect. Dis.*, **2012**, *31(11)*, 2919-2928. https://doi.org/10.1007/s10096-012-1642-6
5. Wang, D. Y.; Hadj-Henni, L.; Thierry, S.; Arné, P.; Chermette, R.; Botterel, F.; Hadrich, I.; Makni, F.; Ayadi, A.; Ranque, S.; Huang, W. Y.; Guillot, J. Simple and highly discriminatory VNTR-based multiplex PCR for tracing sources of *Aspergillus flavus* isolates. *PLoS One*, **2012**, *7(9)*, e44204. https://doi.org/10.1371/journal.pone.0044204
6. Romeo, O.; Delfino, D.; Cascio, A.; Passo, C. L.; Amorini, M.; Romeo, D.; Pernice, I. Microsatellite-based genotyping of *Candida parapsilosis sensu stricto* isolates reveals dominance and persistence of a particular epidemiological clone among neonatal intensive care unit patients. *Infect. Genet. Evol.*, **2013**, *13*, 105-108. https://doi.org/10.1016/j.meegid.2012.09.006
7. Spesso, M. F.; Nuncira, C. T.; Burstein, V. L.; Masih, D. T.; Dib, M. D.; Chiapello, L. S. Microsatellite-primed PCR and random primer amplification polymorphic DNA for the identification and epidemiology of dermatophytes. *Eur. J. Clin. Microbiol. Infect. Dis.*, **2013**, *32(8)*, 1009-1015. https://doi.org/10.1007/s10096-013-1839-3
8. Ahmad, K. M.; Kokošar, J.; Guo, X.; Gu, Z.; Ishchuk, O. P.; Piškur, J. Genome structure and dynamics of the yeast pathogen *Candida glabrata*. *FEMS Yeast Res.*, **2014**, *14(4)*, 529-535. https://doi.org/10.1111/1567-1364.12145
9. Dehghan, P.; Bui, T.; Campbell, L. T.; Lai, Y. W.; Tran-Dinh, N.; Zaini, F.; Carter, D. A. Multilocus variable-number tandem-repeat analysis of clinical isolates of *Aspergillus flavus* from Iran reveals the first cases of *Aspergillus minisclerotigenes* associated with human infection. *BMC Infect. Dis.*, **2014**, *14(1)*, 1-14. https://doi.org/10.1186/1471-2334-14-358
10. Rudramurthy, S. M.; Paul, R. A.; Chakrabarti, A.; Mouton, J. W.; Meis, J. F. Invasive aspergillosis by *Aspergillus flavus*: epidemiology, diagnosis, antifungal resistance, and management. *J. Fungi*, **2019**, *5(3)*, 55. https://doi.org/10.3390/jof5030055.

11. Drira, I.; Hadrich, I.; Neji, S.; Mahfouth, N.; Trabelsi, H.; Sellami, H.; Makni, F.; Ayadi, A. Highly discriminatory variable-number tandem-repeat markers for genotyping of *Trichophyton interdigitale* strains. *J. Clin. Microbiol.*, **2014**, *52(9)*, 3290-3296. https://doi.org/10.1128/JCM.00828-14
12. Nascimento, E.; Bonifácio da Silva, M. E. N.; Martinez, R.; von Zeska Kress, M. R. Primary cutaneous cryptococcosis in an immunocompetent patient due to *Cryptococcus gattii* molecular type VGI in Brazil: a case report and review of literature. *Mycoses*, **2014**, *57(7)*, 442-447. https://doi.org/10.1111/myc.12176
13. Wu, Y.; Zhou, H. J.; Che, J.; Li, W. G.; Bian, F. N.; Yu, S. B.; Zhang, L.; Lu, J. Multilocus microsatellite markers for molecular typing of *Candida tropicalis* isolates. *BMC Microbiol.*, **2014**, *14(1)*, 1-12. https://doi.org/10.1186/s12866-014-0245-z
14. Cariani, L.; Biffi, A.; Guarneri, D.; Girelli, D.; Teri, A.; D'Accico, M.; Beltrami, B.; Arghittu, M.; Torresani, E.; Colombo, C. 56 Colonization by *Rasamsonia argillacea* in cystic fibrosis patients: a two-year retrospective study. *J. Cyst. Fibros.*, **2015**, *14(1)*, S71. https://doi.org/10.1016/S1569-1993(15)30233-2
15. Kathuria, S.; Sharma, C.; Singh, P. K.; Agarwal, P.; Agarwal, K.; Hagen, F.; Meis, J. F.; Chowdhary, A. Molecular epidemiology and in-vitro antifungal susceptibility of *Aspergillus terreus* species complex isolates in Delhi, India: evidence of genetic diversity by amplified fragment length polymorphism and microsatellite typing. *PLoS One*, **2015**, *10(3)*, e0118997. https://doi.org/10.1371/journal.pone.0118997
16. Lackner, M.; Coassin, S.; Haun, M.; Binder, U.; Kronenberg, F.; Haas, H. D.; Maurer, M.; Meis, J. F.; Hagen, F.; Lass-Flörl, C. Geographically predominant genotypes of *Aspergillus terreus* species complex in Austria: a microsatellite typing study. *Clin. Microbiol. Infec.*, **2016**, *22(3)*, 270-276. https://doi.org/10.1016/j.cmi.2015.10.021
17. Gong, J.; Wu, W.; Ran, M.; Wang, X.; Liu, W.; Wan, Z.; Yao, L.; Li, R. Population differentiation and genetic diversity of *Trichophyton rubrum* as revealed by highly discriminatory microsatellites. *Fungal Genet. Biol.*, **2016**, *95*, 24-29. https://doi.org/10.1016/j.fgb.2016.08.002
18. Ngouana, T. K.; Drakulovski, P.; Krasteva, D.; Kouanfack, C.; Reynes, J.; Delaporte, E.; ; Bertout, S. *Cryptococcus neoformans* isolates from Yaoundé human immunodeficiency virus-infected patients exhibited intra-individual genetic diversity and variation in antifungal susceptibility profiles between isolates from the same patient. *J. Med. Microbiol.*, **2016**, *65(7)*, 579-589. https://doi.org/10.1099/jmm.0.000265
19. Badali, H.; Rezaie, S.; Meis, J. F.; Afshari, S. A. K.; Modiri, M.; Hagen, F.; Moazeni, M.; Mohammadi, R.; Khodavaisy, S. Microsatellite genotyping of clinical *Candida parapsilosis* isolates. *Curr. Med. Mycol.*, **2017**, *3(4)*, 15. https://doi.org/10.29252/cmm.3.4.15
20. Loeffert, S. T.; Melloul, E.; Dananché, C.; Hénaff, L.; Bénet, T.; Cassier, P.; Dupont, D.; Guillot, J.; Botterel, F.; Wallon, M.; Gustin, M.-P.; Vanhems, P. Monitoring of clinical strains and environmental fungal aerocontamination to prevent invasive aspergillosis infections in hospital during large deconstruction work: a protocol study. *BMJ Open*, **2017**, *7(11)*, e018109. http://dx.doi.org/10.1136/bmjopen-2017-018109
21. Neji, S.; Hadrich, I.; Ilahi, A.; Trabelsi, H.; Chelly, H.; Mahfoudh, N.; Cheikhrouhou, F.; Sellami, H.; Makni, F.; Ayadi, A. Molecular genotyping of *Candida parapsilosis* species complex. *Mycopathol.*, **2018**, *183(5)*, 765-775. https://doi.org/10.1007/s11046-018-0278-1
22. Al-Sweih, N.; Ahmad, S.; Khan, S.; Joseph, L.; Asadzadeh, M.; Khan, Z. *Cyberlindnera fabianii* fungaemia outbreak in preterm neonates in Kuwait and literature review. *Mycoses*, **2019**, *62(1)*, 51-61. https://doi.org/10.1111/myc.12846
23. Desoubeaux, G.; Nourrisson, C.; Moniot, M.; De Kyvon, M. A.; Bonnin, V.; De La Bretonniére, M. E.; De La Bretonniére, M. E.; Morange, V.; Bailly, É.; Lemaignen, A.; Morio, F.; Poirier, P. Genotyping approach for potential common source of *Enterocytozoon bieneusi* infection in hematology unit. *Emerg. Infect. Dis.*, **2019**, 25(9), 1625. https://doi.org/10.3201/eid2509.190311
24. Gong, J.; Zhang, M.; Li, R.; He, L.; Wan, Z.; Li, F.; Zhang, J. Population structure and genetic diversity of *Sporothrix globosa* in China according to 10 novel microsatellite loci. *J. Med. Microbiol.*, **2019**, *68(2)*, 248-254. https://doi.org/10.1099/jmm.0.000896
25. Lim, W.; Eadie, K.; Horst-Kreft, D.; Ahmed, S. A.; Fahal, A. H.; van de Sande, W. W. VNTR confirms the heterogeneity of *Madurella mycetomatis* and is a promising typing tool for this mycetoma causing agent. *Med. Mycol.*, **2019**, *57(4)*, 434-440. https://doi.org/10.1093/mmy/myy055
26. Lim, W., Eadie, K., Konings, M., Rijnders, B., Fahal, A. H., Oliver, J. D., Birch, M.; Verbon, A.; van de Sande, W. *Madurella mycetomatis*, the main causative agent of eumycetoma, is highly susceptible to olorofim. *J. Antimicrob. Chemother.*, **2020**, *75(4)*, 936-941. https://doi.org/10.1093/jac/dkz529
27. Nyuykonge, B.; Eadie, K.; Zandijk, W. H.; Ahmed, S. A.; Desnos-Ollivier, M.; Fahal, A. H.; de Hoog, S.; Verbon, A.;. van de Sande. W. W. J.; Klaassen, C. H. A Short-Tandem-Repeat assay (Mmy STR) for studying genetic variation in *Madurella mycetomatis*. *J. Clin. Microbiol.*, **2021**, *59(3)*, e02331-20. https://doi.org/10.1128/JCM.02331-20
28. Loeffert, S. T.; Melloul, E.; Gustin, M. P.; Hénaff, L.; Guillot, C.; Dupont, D.; Wallon, M.; Cassier, P.; Dananché, C.; Bénet, T.; Botterel, F.; Guillot, J.; Vanhems, P. Investigation of the relationships between clinical and environmental isolates of *Aspergillus fumigatus* by multiple-locus variable number tandem repeat analysis during major demolition work in a French hospital. *Clin. Infect. Dis.*, **2019**, *68(2)*, 321-329. https://doi.org/10.1093/cid/ciy498
29. Ahmad, S.; Khan, Z.; Al-Sweih, N.; Alfouzan, W.; Joseph, L.; Asadzadeh, M. *Candida kefyr* in Kuwait: Prevalence, antifungal drug susceptibility and genotypic heterogeneity. *PloS One*, **2020**, *15(10)*, e0240426. https://doi.org/10.1371/journal.pone.0240426
30. Gabaldón, T.; Gómez-Molero, E.; Bader, O. Molecular typing of *Candida glabrata*. *Mycopathol.*, **2020**, *185(5)*, 755-764. https://doi.org/10.1007/s11046-019-00388-x
31. Gits-Muselli, M.; Campagne, P.; Desnos-Ollivier, M.; Le Pape, P.; Bretagne, S.; Morio, F.; Alanio, A. Comparison of multilocus sequence typing (MLST) and microsatellite length polymorphism (MLP) for *Pneumocystis jirovecii* genotyping. *Comput. Struct. Biotechnol. J.*, **2020**, *18*, 2890-2896. https://doi.org/10.1016/j.csbj.2020.10.005

32. Almeida, J. N. D; Francisco, E. C.; Hagen, F.; Brandão, I. B.; Pereira, F. M.; Presta Dias, P. H.; Costa, M. M. M.; Jordão, R. T. S.; de Groot, T.; Colombo, A. L. Emergence of *Candida auris* in Brazil in a COVID-19 intensive care. *J. Fungi*, **2021**, *7(3)*, 220. https://doi.org/10.3390/jof7030220
33. Borgio, J. F.; Rasdan, A. S.; Sonbol, B.; Alhamid, G.; Almandil, N. B.; AbdulAzeez, S. Emerging status of multidrug-resistant bacteria and fungi in the Arabian Peninsula. *Biology*, **2021**, *10(11)*, 1144. https://doi.org/10.3390/biology10111144
34. van der Torre, M. H.; Shen, H.; Rautemaa-Richardson, R.; Richardson, M. D.; Novak-Frazer, L. Molecular epidemiology of *Aspergillus fumigatus* in chronic pulmonary aspergillosis patients. *J. Fungi*, **2021**, *7(2)*, 152. https://doi.org/10.3390/jof7020152
35. Yadav, A.; Singh, A.; Wang, Y.; van Haren, M. H.; Singh, A.; de Groot, T.; Meis; J. F.; Xu, J.; Chowdhary, A. Colonisation and transmission dynamics of *Candida auris* among chronic respiratory diseases patients hospitalised in a chest hospital, Delhi, India: a comparative analysis of whole genome sequencing and microsatellite typing. *J. Fungi*, **2021**, *7(2)*, 81. https://doi.org/10.3390/jof7020081
36. Hare, R. K.; Arastehfar, A.; Rosendahl, S.; Charsizadeh, A.; Daneshnia, F.; Eshaghi, H.; Mirhendi, H.; Boekhout, T.; Hagen, F.; Arendrup, M. C. Candidemia among hospitalized pediatric patients caused by several clonal lineages of *Candida parapsilosis*. *J. Fungi*, **2022**, *8(2)*, 183. https://doi.org/10.3390/jof8020183
37. Torky, H.; Khalifa, E. *Trichoderma pseudokoningii* Rifai isolation from Egyptian immunocompromised cattle with *Mycobacterium bovis* infection. *Nat. Preced.*, **2012**, 1-1. https://doi.org/10.1038/npre.2012.6928.1
38. Wang, D. Y. Genetic diversity and antifungal susceptibility of *Aspergillus* spp. isolates from avian farms in Guangxi, China. Doctoral Thesis, University of Guangxi, 2012
39. Thierry, S.; Durand, B.; Melloul, E.; Tafani, J. P.; Wang, D. Y.; Deville, M.; Cordonnier, N.; Chermette, R.; Guillot, J.; Arné, P. Assessment of *Aspergillus fumigatus* pathogenicity in aerosol-challenged chickens (*Gallus gallus*) belonging to two lineages. *Comp. Immunol. Microbiol. Infect. Dis.*, **2013**, *36(4)*, 379-385. https://doi.org/10.1016/j.cimid.2013.01.003
40. Miao, Z.; Li, S.; Li, D.; Cai, C.; Cai, Y. Rapid detection for rabbit-derived dermatophytes using microsatellite-primed polymerase chain reaction. *Microb. Physiol.*, **2014**, *24(1)*, 53-58. https://doi.org/10.1159/000356295
41. Pérez-González, V. H.; Guzmán-Franco, A. W.; Alatorre-Rosas, R.; Hernández-López, J.; Hernández-López, A.; Carrillo-Benítez, M. G.; Baverstock, J. Specific diversity of the entomopathogenic fungi *Beauveria* and *Metarhizium* in Mexican agricultural soils. *J. Invertebr. Pathol.*, **2014**, *119*, 54-61. https://doi.org/10.1016/j.jip.2014.04.004
42. Reineke, A.; Bischoff-Schaefer, M.; Rondot, Y.; Galidevara, S.; Hirsch, J.; Devi, K. U. Microsatellite markers to monitor a commercialized isolate of the entomopathogenic fungus *Beauveria bassiana* in different environments: Technical validation and first applications. *Biol. Control*, **2014**, *70*, 1-8. https://doi.org/10.1016/j.biocontrol.2013.11.012
43. Fischer, D.; Lierz, M. Diagnostic procedures and available techniques for the diagnosis of aspergillosis in birds. *J. Exot. Pet Med.*, **2015**, *24(3)*, 283-295. https://doi.org/10.1053/j.jepm.2015.06.016
44. Kepler, R. M.; Ugine, T. A.; Maul, J. E.; Cavigelli, M. A.; Rehner, S. A. Community composition and population genetics of insect pathogenic fungi in the genus *Metarhizium* from soils of a long-term agricultural research system. *Environ. Microbiol.*, **2015**, *17(8)*, 2791-2804. https://doi.org/10.1111/1462-2920.12778
45. Mayerhofer, J.; Lutz, A.; Widmer, F.; Rehner, S. A.; Leuchtmann, A.; Enkerli, J. Multiplexed microsatellite markers for seven *Metarhizium* species. *J. Invertebr. Pathol.*, **2015**, *132*, 132-134. https://doi.org/10.1016/j.jip.2015.09.006
46. Álvarez-Pérez, S.; García, M. E.; Peláez, T.; Blanco, J. L. Genotyping and antifungal susceptibility testing of multiple *Malassezia pachydermatis* isolates from otitis and dermatitis cases in pets: is it really worth the effort?. *Med. Mycol.*, **2016**, *54(1)*, 72-79. https://doi.org/10.1093/mmy/myv070
47. Deng, L.; Li, W.; Zhong, Z.; Gong, C.; Liu, X.; Huang, X.; Xiao, L:, Zhao, R.; Wang, W.; Feng, F.; Zhang, Y.; Hu, Y.; Fu, H.; He, M.; Zhang, Y.; Wu, K.; Peng, G. Molecular characterization and multilocus genotypes of *Enterocytozoon bieneusi* among horses in southwestern China. *Parasites Vectors*, **2016**, *9(1)*, 1-6. https://doi.org/10.1186/s13071-016-1844-3
48. Lee, S. H.; Oem, J. K.; Lee, S. M.; Son, K.; Jo, S. D.; Kwak, D. Molecular detection of *Enterocytozoon bieneusi* from bats in South Korea. *Med. Mycol.*, **2018**, *56(8)*, 1033-1037. https://doi.org/10.1093/mmy/myx136
49. Bovio, E.; Sfecci, E.; Poli, A.; Gnavi, G.; Prigione, V.; Lacour, T.; Mehiri, M.; Varese, G. C. The culturable mycobiota associated with the Mediterranean sponges *Aplysina cavernicola*, *Crambe crambe* and *Phorbas tenacior*. *FEMS Microbiol. Lett.*, **2019**, *366(24)*, fnaa014. https://doi.org/10.1093/mmy/myx136
50. Dool, S.; Altewischer, A.; Fischer, N. M.; Drees, K. P.; Foster, J. T.; Fritze, M.; Puechmaille, S. J. Mating type determination within a microsatellite multiplex for the fungal pathogen *Pseudogymnoascus destructans*, the causative agent of white-nose disease in bats. *Conserv. Genet. Res.*, **2020**, *12(1)*, 45-48. https://doi.org/10.1007/s12686-018-1064-6
51. Wakil, W.; Kavallieratos, N. G.; Ghazanfar, M. U.; Usman, M.; Habib, A.; El-Shafie, H. A. Efficacy of different entomopathogenic fungal isolates against four key stored-grain beetle species. *J. Stored Prod. Res.*, *2021*, *93*, 101845. https://doi.org/10.1016/j.jspr.2021.101845
52. Hadrich, I.; Amouri, I.; Neji, S.; Mahfoud, N.; Ranque, S.; Makni, F.; Ayadi, A. Genetic structure of *Aspergillus flavus* populations in human and avian isolates. *Eur. J. Clin. Microbiol. Infect. Dis.*, **2013**, *32(2)*, 277-282. https://doi.org/10.1007/s10096-012-1740-5
53. Pasquetti, M.; Peano, A.; Soglia, D.; Min, A. R. M.; Pankewitz, F.; Ohst, T.; Gräser, Y. Development and validation of a microsatellite marker-based method for tracing infections by *Microsporum canis*. *J. Dermatol. Sci.*, **2013**, *70(2)*, 123-129. https://doi.org/10.1016/j.jdermsci.2013.01.003
54. Asran-Amal, A. Molecular identification of *Macrophomina phaseolina* by microsatellite-based fingerprint. *Plant Pathol. Quar.*, **2012**, *2*, 143-151. https://doi.org/10.5943/ppq/2/2/7
55. Bengtsson, S. B.; Vasaitis, R.; Kirisits, T.; Solheim, H.; Stenlid, J. Population structure of *Hymenoscyphus pseudoalbidus* and its genetic relationship to *Hymenoscyphus albidus*. *Fungal Ecol.*, **2012**, *5(2)*, 147-153. https://doi.org/10.1016/j.funeco.2011.10.004

56. Bills, G. F.; González-Menéndez, V.; Martín, J.; Platas, G.; Fournier, J.; Peršoh, D.; Stadler, M. *Hypoxylon pulicicidum* sp. nov. (Ascomycota, Xylariales), a pantropical insecticide-producing endophyte. *PLoS One*, **2012**, *7(10)*, e46687. https://doi.org/10.1016/j.funeco.2011.10.004
57. Dilmaghani, A.; Gladieux, P.; Gout, L.; Giraud, T.; Brunner, P. C.; Stachowiak, A.; Rouxel, T. B. Migration patterns and changes in population biology associated with the worldwide spread of the oilseed rape pathogen *Leptosphaeria maculans*. *Mol. Ecol.*, **2012**, *21(10)*, 2519-2533. https://doi.org/10.1111/j.1365-294X.2012.05535.x
58. Everhart, S. E.; Askew, A.; Seymour, L.; Glenn, T. C.; Scherm, H. Spatial patterns of brown rot epidemics and development of microsatellite markers for analyzing fine-scale genetic structure of *Monilinia fructicola* populations within peach tree canopies. *Plant Health Prog.*, **2012**, *13(1)*, 28. https://doi.org/10.1094/PHP-2012-0723-04-RS
59. Ferreira, C. F.; Rocha, H. S.; Moreira, R. F. C.; Gomes, L. I. S. Genomics of *Mycosphaerella fijiensis* - the causal agent of black sigatoka in *Musa* spp. Genetics, Genomics, and Breeding of Bananas, 2012, 217-230
60. Frenkel, O.; Portillo, I.; Brewer, M. T.; Peros, J. P.; Cadle-Davidson, L.; Milgroom, M. G. Development of microsatellite markers from the transcriptome of *Erysiphe necator* for analysing population structure in North America and Europe. *Plant Pathol.*, **2012**, *61(1)*, 106-119. https://doi.org/10.1111/j.1365-3059.2011.02502.x
61. Hadziabdic, D.; Wadl, P. A.; Vito, L. M.; Boggess, S. L.; Scheffler, B. E.; Windham, M. T.; Trigiano, R. N. Development and characterization of sixteen microsatellite loci for *Geosmithia morbida*, the causal agent of thousand canker disease in black walnut (*Juglans nigra*). *Conserv. Genet. Res.*, **2012**, *4(2)*, 287-289. https://doi.org/10.1007/s12686-011-9526-0
62. Luzaran, R. T.; Cueva, F. M.; Cumagun, C. J. R.; Velasco, L. R. I.; Dalisay, T. U. Variability of sugarcane smut pathogen, *Ustilago scitaminea Sydow* in the Philippines. *Philipp. J. Crop. Sci.*, **2012**, *37(2)*, 38-51. ISSN: 0115-463X
63. Purnima, S.; Sundeep, K.; Uttam, K.; Lakshman, P.; Singh, A. K.; Rakesh, S.; Joshi, A. K. Pathological and molecular characterizations of slow leaf rusting in fifteen wheat (*Triticum aestivum* L. em Thell) genotypes. *Afr. J. Biotechnol.*, **2012**, *11(84)*, 14956-14966. https://doi.org/10.5897/AJB12.1894
64. Gultyaeva, E. I.; Shaidayuk, E. L.; Kazartsev, I. A. Structure of *Puccinia triticina* populations on tetraploid wheat species. *Mikol. Fitopatol.*, **2017**, *51(5)*, 299-304. ISSN: 0026-3648
65. Rouxel, M.; Papura, D.; Nogueira, M.; Machefer, V.; Dezette, D.; Richard-Cervera, S.; Carrere, S.; Mestre, P.; Delmotte, F.. Microsatellite markers for characterization of native and introduced populations of *Plasmopara viticola*, the causal agent of grapevine downy mildew. *Appl. Environ. Microbiol.*, **2012**, *78(17)*, 6337-6340. https://doi.org/10.1128/AEM.01255-12
66. Simpson, M. C. Microsatellite analysis of *Ceratocystis fimbriata*. Doctoral Thesis, University of Pretoria, **2012**.
67. Simpson, M. C., Wilken, P. M., Coetzee, M. P., Wingfield, M. J., Wingfield, B. D. Analysis of microsatellite markers in the genome of the plant pathogen *Ceratocystis fimbriata*. *Fungal Biol.*, **2013**, *117(7-8)*, 545-555. https://doi.org/10.1016/j.funbio.2013.06.004
68. Trigiano, R. N.; Wadl, P. A.; Dean, D.; Hadziabdic, D.: Scheffler, B. E.; Runge, F.; Ristaino, J.; Spring, O. Ten polymorphic microsatellite loci identified from a small insert genomic library for *Peronospora tabacina*. *Mycologia*, **2012**, *104(3)*, 633-640. https://doi.org/10.3852/11-288
69. Asadollahi, M.; Fekete, E.; Karaffa, L.; Flipphi, M.; Árnyasi, M.; Esmaeili, M.; Váczy, K. Z.; Sándor, E. Comparison of *Botrytis cinerea* populations isolated from two open-field cultivated host plants. *Microbiol. Res.*, **2013**, *168(6)*, 379-388. https://doi.org/10.1016/j.micres.2012.12.008
70. Borah, N.; Albarouki, E.; Schirawski, J. Comparative methods for molecular determination of host-specificity factors in plant-pathogenic fungi. *Int. J. Mol. Sci.*, **2018**, *19(3)*, 863. https://doi.org/10.3390/ijms19030863
71. Berlin, A.; Kyaschenko, J.; Justesen, A. F.; Yuen, J. Rust fungi forming aecia on *Berberis* spp. in Sweden. *Plant Dis.*, **2013**, *97(10)*, 1281-1287. https://doi.org/10.1094/PDIS-10-12-0989-RE
72. Burgos, M. R. G.; Katimbang, M. L. B.; Dela Paz, M. A. G.; Beligan, G. A.; Goodwin, P. H.; Ona, I. P.; Mauleon, R. P.; Ardales, E. Y.; Vera Cruz, C. M. Genotypic variability and aggressiveness of *Bipolaris oryzae* in the Philippines. *Eur. J. Plant Pathol.*, **2013**, *137(2)*, 415-429. https://doi.org/10.1007/s10658-013-0256-x
73. Ahmadpour, A.; Castell-Miller, C.; Javan-Nikkhah, M.; Naghavi, M. R.; Dehkaei, F. P.; Leng, Y.; Puri, K. D.; Zhong, S. Population structure, genetic diversity, and sexual state of the rice brown spot pathogen *Bipolaris oryzae* from three Asian countries. *Plant Pathol.*, **2018**, *67(1)*, 181-192. https://doi.org/10.1111/ppa.12714
74. Chaijuckam, P.; Songkumarn, P.; Guerrero, J. J. G. Genetic diversity and aggressiveness of *Bipolaris oryzae* in North-Central Thailand. *Appl. Sci. Eng. Prog.*, **2019**, *12(2)*, 116-125.
75. Cai, G.; Leadbetter, C. W.; Muehlbauer, M. F.; Molnar, T. J.; Hillman, B. I. Genome-wide microsatellite identification in the fungus *Anisogramma anomala* using Illumina sequencing and genome assembly. *PLoS One*, **2013**, *8(11)*, e82408. https://doi.org/10.1371/journal.pone.0082408
76. Halas, W. Molecular genetic characterization of fungal isolates representing biogeographic diversity in the *Colletotrichum*-bean pathosystem. MSc thesis, University of Bedfordshire, 2013
77. Peixoto Junior, R. F.; Figueira, A. V. O.; Landell, M. G. A.; Nunes, D. S.; Pinto, L. R.; Sanguino, A.; Anjos, A. I.; Creste, S. Development and characterisation of microsatellite markers for *Puccinia melanocephala*, causal agent of sugarcane brown rust. In International Society of Sugar Cane Technologists: Proceedings of the XXVIIIth Congress, June 24 to June 27, 2013, São Paulo, Brazil. Sociedade dos Técnicos Açucareiros e Alcooleiros do Brasil (STAB) & The XXVIIIth ISSCT Organising Committee. 2013
78. Schoebel, C. N.; Jung, E.; Prospero, S. Development of new polymorphic microsatellite markers for three closely related plant-pathogenic *Phytophthora* species using 454-pyrosequencing and their potential applications. *Phytopathology*, **2013**, *103(10)*, 1020-1027. https://doi.org/10.1094/PHYTO-01-13-0026-R
79. Aylward, J. Diversity and dispersal of the ophiostomatoid fungus, *Knoxdaviesia proteae*, within *Protea repens* infructescences. Doctoral Thesis, Stellenbosch University, 2014

80. Bühlmann, A.; Dreo, T.; Rezzonico, F.; Pothier, J. F.; Smits, T. H.; Ravnikar, M.; Frey, J. E.; Duffy, B. Phylogeography and population structure of the biologically invasive phytopathogen *Erwinia amylovora* inferred using minisatellites. *Environ. Microbiol.*, **2014**, *16(7)*, 2112-2125. https://doi.org/10.1111/1462-2920.12289
81. Burchhardt, K. M. Microsatellite markers reveal population structure, genetic diversity, and the reproductive biology of the blueberry pathogen *Monilinia vaccinii-corymbosi*. North Carolina State University, 2014
82. Elefsen, S. E.; Frey, P.; Sverrisson, H.; Hallsson, J. H. Microsatellite analysis of Icelandic populations of the poplar fungal pathogen *Melampsora larici-populina* shows evidence of repeated colonization events. *Eur. J. Plant Pathol.*, **2014**, *139(3)*, 597-608. https://doi.org/10.1007/s10658-014-0416-7
83. Haasbroek, M. P.; Craven, M.; Barnes, I.; Crampton, B. G. Microsatellite and mating type primers for the maize and sorghum pathogen, *Exserohilum turcicum*. *Australas. Plant Pathol.*, **2014**, *43(5)*, 577-581. https://doi.org/10.1007/s13313-014-0289-4
84. Janoušek, J.; Krumböck, S.; Kirisits, T.; Bradshaw, R. E.; Barnes, I.; Jankovský, L.; Stauffer, C. Development of microsatellite and mating type markers for the pine needle pathogen *Lecanosticta acicola*. Australas. *Plant Pathol.*, **2014**, *43(2)*,161-165. https://doi.org/10.1007/s13313-013-0256-5
85. Leyva-Madrigal, K. Y., Larralde-Corona, C. P., Calderón-Vázquez, C. L., Maldonado-Mendoza, I. E. Genome distribution and validation of novel microsatellite markers of *Fusarium verticillioides* and their transferability to other *Fusarium* species. *J. Microbiol. Methods*, **2014**, *101*, 18-23. https://doi.org/10.1016/j.mimet.2014.03.011
86. Li, W.; Feng, Y.; Sun, H.; Deng, Y.; Yu, H.; Chen, H. Analysis of simple sequence repeats in the *Gaeumannomyces graminis* var. *tritici* genome and the development of microsatellite markers. *Curr. Genet.*, **2014**, *60(4)*, 237-245. https://doi.org/10.1007/s00294-014-0428-z
87. Marulanda, M. L.; López, A. M.; Isaza, L.; López, P. Microsatellite isolation and characterization for *Colletotrichum* spp, causal agent of anthracnose in Andean blackberry. *Genet. Mol. Res.*, **2014**, *13*, 7673-7685. http://dx.doi.org/10.4238/2014.September.26.5
88. Rondot, Y.; Reineke, A. Potential of the entomopathogenic fungus *Beauveria bassiana* as an endophyte in grapevine *Vitis vinifera* (L.) plants., **2014**, *105*, 35-43.
89. Sharma, G.; Kumar Pinnaka, A.; Damodara Shenoy, B. Infra-specific diversity of *Colletotrichum truncatum* associated with chilli anthracnose in India based on microsatellite marker analysis. *Arch. Phytopathol. Pflanzenschutz*, **2014**, *47(20)*, 2509-2523. https://doi.org/10.1080/03235408.2014.880577
90. Singh, R.; Kumar, S.; Kashyap, P. L.; Srivastava, A. K.; Mishra, S.; Sharma, A. K. Identification and characterization of microsatellite from *Alternaria brassicicola* to assess cross-species transferability and utility as a diagnostic marker. *Mol. Biotechnol.*, **2014**, *56(11)*, 1049-1059. https://doi.org/10.1007/s12033-014-9784-7
91. Tansian, P.; Sreewongchai, T.; Parinthawong, N. Genetic diversity analysis of rice blast pathogen in Thailand using *Magnaporthe grisea* microsatellite (MGM) markers. In Agricultural sciences: leading Thailand to world class standards. Proceedings of the 52nd Kasetsart University Annual Conference, 4-7 February 2014, Kasetsart University, Thailand. Vol. 1: Plants (pp. 400-406). Kasetsart University
92. von Cräutlein, M.; Korpelainen, H.; Helander, M.; Öhberg, A.; Saikkonen, K. Development and characterization of nuclear microsatellite markers in the endophytic fungus *Epichloë festucae* (Clavicipitaceae). *Appl. Plant Sci.*, **2014**, *2(12)*,1400093. https://doi.org/10.3732/apps.1400093
93. Wang, M.; Xue, F; Yang, P.; Duan, X. Y., Zhou, Y. L., Shen, C. Y., Zhang, G.; Wang, B. T. Development of SSR markers for a phytopathogenic fungus, *Blumeria graminis f. sp. tritici*, using a FIASCO protocol. *J. Integr. Agric.*, **2014**, *13(1)*, 100-104. https://doi.org/10.1016/S2095-3119(13)60510-9
94. Wang, Y.; Hou, Y.; Bo, H.; Zhou, H.; Jing, L.; Zhao, J. Mycelial compatibility groups and microsatellite markers reveal genetic diversity within and among populations of sunflower *Sclerotinia sclerotiorum* in China. *Int. J. Phytopathol.*, **2014**, *3(1)*, 21-31.
95. Brewer, M. T.; Rath, M.; Li, H. X. Genetic diversity and population structure of cucurbit gummy stem blight fungi based on microsatellite markers. *Phytopathol.*, **2015**, *105(6)*, 815-824. https://doi.org/10.1094/PHYTO-10-14-0282-R
96. Cardozo Burgos, C.; Silva Aguilar, B.; Salazar Yepes, M.; Gonzalo Morales Osorio, J. Genetic diversity of *Ralstonia solanacearum* isolates from three Colombian regions. *Rev. Prot. Veg.*, **2015**, *30(3)*, 213-224. ISSN: 1010-2752
97. Chowdhury, M. T. I.; Mian, M. S.; Mia, M. T.; Rafii, M. Y.; Latif, M. A. Agro-ecological variations of sheath rot disease of rice caused by *Sarocladium oryzae* and DNA fingerprinting of the pathogen's population structure. *Gen. Mol. Res.*, **2015**, *14(4)*, 18140-18152. http://dx.doi.org/10.4238/2015.December.23.1
98. Gonzaga, L. L.; Costa, L. E. O.; Santos, T. T.; Araújo, E. F.; Queiroz, M. V. Endophytic fungi from the genus *Colletotrichum* are abundant in the *Phaseolus vulgaris* and have high genetic diversity. *J. App. Microbiol.*, **2015**, *118(2)*, 485-496. https://doi.org/10.1111/jam.12696
99. Jia, Q.; Gu, Q.; Zheng, L.; Hsiang, T.; Luo, C.; Huang, J. Genetic analysis of the population structure of the rice false smut fungus, *Villosiclava virens*, in China using microsatellite markers mined from a genome assembly. *Plant Pathol.*, **2012**, *64(6)*, 1440-1449. https://doi.org/10.1111/ppa.12373
100. Mercière, M.; Laybats, A.; Carasco-Lacombe, C.; Tan, J. S.; Klopp, C.; Durand-Gasselin, T.; Alwee, S. S. R. S.; Camus-Kulandaivelu, L.; Breton, F. Identification and development of new polymorphic microsatellite markers using genome assembly for *Ganoderma boninense*, causal agent of oil palm basal stem rot disease. *Mycol. Prog.*, **2015**, *14(11)*, 1-11. https://doi.org/10.1007/s11557-015-1123-2
101. Michalecka, M.; Bryk, H.; Poniatowska, A.; Seliga, P.; Puławska, J. Identification and characterization of *Neofabraea* fungi causing bull's eye rot on apple in Poland. In III International Symposium on Postharvest Pathology: Using Science to Increase Food Availability, **2015**, *1144*, 183-188.

102. Arango Isaza, R. E.; Diaz-Trujillo, C.; Dhillon, B.; Aerts, A.; Carlier, J.; Crane, C. F.; de Jong, T. V.; de Vries, I.; Dietrich, R.; Farmer, A. D.; Fereira, C. F.; Garcia, S.; Guzman, M.; Hamelin, R. C.; Lindquist, E. A.; Mehrabi, R.; Quiros, O.; Schmutz, J.; Shapiro, H.; Reynolds, E.; Scalliet, G.; Souza Jr., M.; Stergiopoulos, I.; Van der Lee, T. A. J.; De Wit, P. J. G. M.; Zapater, M.-F.; Zwiers, L.-H.; Grigoriev, I. V.; Kema, G. H. Combating a global threat to a clonal crop: banana black Sigatoka pathogen *Pseudocercospora fijiensis* (synonym *Mycosphaerella fijiensis*) genomes reveal clues for disease control. *PLoS Genet.*, **2016**, *12(8)*, e1005876.
103. Arzanlou, M.; Narmani, A. Genetic diversity in Iranian populations of *Togninia minima*, one of the causal agents of leaf stripe disease on grapevines. *EurAsian J. Biosci.*, **2016**, *10(1)*, 41-50. https://doi.org/10.1371/journal.pgen.1005876
104. Boiko, S. M. Population structure of the wood-decay fungus *Trichaptum abietinum* (J. Dicks.) Ryvarden in the Carpathian National Nature Park (Ukraine). *Biodivers. Res. Conserv.*, **2016**, *43(1)*, 1. https://doi.org/10.1515/biorc-2016-0017
105. Demers, J. E.; Jiménez-Gasco, M. D. M. Evolution of nine microsatellite loci in the fungus *Fusarium oxysporum*. *J. Mol. Evol.*, **2016**, *82(1)*, 27-37. https://doi.org/10.1007/s00239-015-9725-5
106. dos Santos, T. T.; de Souza Leite, T.; de Queiroz, C. B.; de Araújo, E. F.; Pereira, O. L.; de Queiroz, M. V. High genetic variability in endophytic fungi from the genus *Diaporthe* isolated from common bean (*Phaseolus vulgaris* L.) in Brazil. *J. App. Microbiol.*, **2016**, *120(2)*, 388-401. https://doi.org/10.1111/jam.12985
107. Fortuna, T. M.; Snirc, A.; Badouin, H.; Gouzy, J.; Siguenza, S.; Esquerre, D.; Le Prieur, S.; Shykoff, J. A.; Giraud, T. Polymorphic microsatellite markers for the tetrapolar anther-smut fungus *Microbotryum saponariae* based on genome sequencing. *PloS One*, **2016**, *11(11)*, e0165656. https://doi.org/10.1371/journal.pone.0165656
108. Kandan, A.; Akhtar, J.; Singh, B.; Pal, D.; Chand, D.; Rajkumar, S.; Agarwal, P. C. Genetic diversity analysis of fungal pathogen *Bipolaris sorghicola* infecting *Sorghum bicolor* in India. *J. Environ. Biol.*, **2016**, *37(6)*, 1323. ISSN : 0254-8704
109. Li, H. X.; Brewer, M. T. Spatial genetic structure and population dynamics of gummy stem blight fungi within and among watermelon fields in the southeastern United States. *Phytopathol.*, **2016**, *106(8)*, 900-908. https://doi.org/10.1094/PHYTO-01-16-0006-R
110. Moges, A. D.; Admassu, B.; Belew, D.; Yesuf, M.; Njuguna, J.; Kyalo, M.; Ghimire, S. R. Development of microsatellite markers and analysis of genetic diversity and population structure of *Colletotrichum gloeosporioides* from Ethiopia. *PloS One*, **2016**, *11(3)*, e0151257. https://doi.org/10.1371/journal.pone.0151257
111. Mehta, N.; Hagen, F.; Aamir, S.; Singh, S. K.; Baghela, A. Development of a high-resolution multi-locus microsatellite typing method for *Colletotrichum gloeosporioides*. *Mycobiol.*, **2017**, *45(4)*, 401-408. https://doi.org/10.5941/MYCO.2017.45.4.401
112. Moni, Z. R.; Ali, M. A.; Alam, M. S.; Rahman, M. A.; Bhuiyan, M. R.; Mian, M. S.; Iftekharuddaula, K.; Latif, A.; Khan, M. A. I. Morphological and genetical variability among *Rhizoctonia solani* isolates causing sheath blight disease of rice. *Rice Sci.*, **2016**, *23(1)*, 42-50. https://doi.org/10.1016/j.rsci.2016.01.005
113. Wang, J.; Chilvers, M. I. Development and characterization of microsatellite markers for *Fusarium virguliforme* and their utility within clade 2 of the *Fusarium solani* species complex. *Fungal Ecol.*, **2016**, *20*, 7-14. https://doi.org/10.1016/j.funeco.2015.09.013
114. Yu, M.; Yu, J.; Li, H.; Wang, Y.; Yin, X.; Bo, H.; Dingac, H.; Zhoua, Y.; Liu, Y. Survey and analysis of simple sequence repeats in the *Ustilaginoidea virens* genome and the development of microsatellite markers. *Gene*, **2016**, *585(1)*, 28-34. https://doi.org/10.1016/j.gene.2016.03.016
115. Laraba, I.; Boureghda, H.; Abdallah, N.; Bouaicha, O.; Obanor, F.; Moretti, A.; Geiser, D. M.; Kim, H.-S.; McCormick, S. P.; Proctor, R. H.; Kelly, A. C.; Ward, T. J.; O'Donnell, K. Population genetic structure and mycotoxin potential of the wheat crown rot and head blight pathogen *Fusarium culmorum* in Algeria. *Fungal Genet. Biol.*, **2017**, *103*, 34-41. https://doi.org/10.1016/j.fgb.2017.04.001
116. Nourollahi, K.; Madahjalali, M. Analysis of population genetic structure of Iranian *Fusarium oxysporum f. sp. lentis* isolates using microsatellite markers. *Australas. Plant Pathol.*, **2017**, *46(1)*, 35-42. https://doi.org/10.1007/s13313-016-0458-8
117. Siah, A.; Bomble, M.; Reignault, P.; Halama, P. Characterization of the population of *Zymoseptoria tritici*, the causal agent of *Septoria tritici* blotch of wheat, in northern France. In 6e COMAPPI, Conférence sur les Moyens Alternatifs de Protection pour une Production Intégrée, Lille, France, 21-23 mars 2017 (pp. 45-52). Association Française de Protection des Plantes (AFPP), 2017
118. Abbate, J. L.; Gladieux, P.; Hood, M. E.; de Vienne, D. M.; Antonovics, J.; Snirc, A.; Giraud, T. Co-occurrence among three divergent plant-castrating fungi in the same *Silene* host species. *Mol. Ecol.*, **2018**, *27(16)*, 3357-3370. https://doi.org/10.1111/mec.14805
119. Capador, H.; Samils, B.; Olson, Å. Development of microsatellite markers for *Thekopsora areolata*, the causal agent of cherry spruce rust. *For. Pathol.*, **2018**, *48(3)*, e12413. https://doi.org/10.1111/efp.12413
120. Gultyaeva, E. I; Shaydayuk, E. L.; Kazartsev, I. A.; Akhmetova, A.; Kosman, E. Microsatellite analysis of *Puccinia triticina* from *Triticum* and *Aegilops* hosts. *Australas. Plant Pathol.*, **2018**, *47(2)*, 163-170. https://doi.org/10.1007/s13313-018-0542-3
121. Jahan, S. B.; Ali, M. A.; Alam, M. S.; Moni, Z. R.; Latif, M. A. Morphological and molecular characterization of *Rhizoctonia oryzaesativae* in Bangladesh. *SAARC J. Agric.*, **2018**, *16(2)*, 119-128. https://doi.org/10.3329/sja.v16i2.40264
122. Mirzapur, S. K. M. T.; Tangail, N. D., Mirzapur; S. K. M. T.; Dhanbari, S.; Jamalpur, N. D.; Dhanbari, S.; ; Tangail, S. T. S. Microsatellite loci for isolates of *Sclerotinia sclerotiorum* collected from ten locations in Bangladesh and one in Ohio, USA. Doctoral Thesis, Ohio State University, **2018**.
123. Mlonyeni, X. O.; Wingfield, M. J.; Greeff, J. M.; Wingfield, B. D.; Slippers, B. Genetic diversity of *Amylostereum areolatum*, the fungal symbiont of the invasive woodwasp *Sirex noctilio* in South Africa. *For. Pathol.*, **2018**, *48(6)*, e12449. https://doi.org/10.1111/efp.12449

124. Rafiei, V.; Banihashemi, Z.; Jiménez-Díaz, R. M.; Navas-Cortés, J. A.; Landa, B. B.; Jiménez-Gasco, M. M.; Turgeon, B. G.; Milgroom, M. G. Comparison of genotyping by sequencing and microsatellite markers for unravelling population structure in the clonal fungus *Verticillium dahliae*. *Plant Pathol.*, **2018**, *67(1)*, 76-86. https://doi.org/10.1111/ppa.12713
125. Sharma, P.; Pandey, B.; Muthusamy, S. K.; Kumar, S.; Saharan, M. S.; Kumar, S.; Singroha, G.; Sharma, I.; Singh, G. P. Development and validation of microsatellite markers for Karnal bunt (*Tilletia indica*) and loose smut (*Ustilago segetum tritici*) of wheat from related fungal species. *J. Phytopathol.*, **2018**, *166(10)*, 729-738. https://doi.org/10.1111/jph.12756
126. Tran-Dinh, N.; Pitt, J. I.; Markwell, P. J. Use of microsatellite markers to assess the competitive ability of nontoxigenic *Aspergillus flavus* strains in studies on biocontrol of aflatoxins in maize in Thailand. *Biocontrol Sci. Technol.*, **2018**, *28(3)*, 215-225. https://doi.org/10.1080/09583157.2018.1436694
127. Xiang, Y. Identification of fungal species causing powdery mildew on cucurbits, determination of the genetic and pathogenic variations of the fungi and their sensitivity to major powdery mildew fungicides. Doctoral Thesis, University of Illinois, 2018
128. Zhang, Y.; He, W.; Yan, D. H. Genomewide identification and development of microsatellite markers for *Marssonina brunnea* and their applications in two populations. *For. Pathol.*, **2018**, *48(4)*, e12433. https://doi.org/10.1111/efp.12433
129. Coleman, C. E.; Meyer, S. E.; Ricks, N. Mating system complexity and cryptic speciation in the seed bank pathogen *Pyrenophora semeniperda*. *Plant Pathol.*, **2019**, *68(2)*, 369-382. https://doi.org/10.1111/ppa.12948
130. Maria, L. R.; Carlucci, A.; Ciccarone, C.; Abderraouf, S.; Lops, F. Identification and pathogenicity of lignicolous fungi associated with grapevine trunk diseases in southern Italy. *Phytopathol. Mediterr.*, **2019**, *58*, 639-662.
131. Najari, G.; Nourollahi, K. Genetic diversity of *Alternaria alternata* the causal agent of saffron corm rot in southern and Razavi Khorasan provinces using microsatellite markers. *JSAT*, **2019**, *7(3)*. https://doi.org/10.22048/jsat.2018.113230.1277
132. Nakamura, N.; Tanaka, C.; Takeuchi-Kaneko, Y. Recombination and local population structure of the root endophytic fungus *Glutinomyces brunneus* based on microsatellite analyses. *Fungal Ecol.*, **2019**, *41*, 56-64. https://doi.org/10.1016/j.funeco.2019.03.009
133. Si, E.; Meng, Y.; Ma, X.; Li, B.; Wang, J.; Ren, P.; Yao, L.; Yang, K.; Zhang, Y.; Shang, X. Wang, H. Development and characterization of microsatellite markers based on whole-genome sequences and pathogenicity differentiation of *Pyrenophora graminea*, the causative agent of barley leaf stripe. *Eur. J. Plant Pathol.*, **2019**, *154(2)*, 227-241. https://doi.org/10.1007/s10658-018-01651-5
134. Udayashankar, A. C.; Chandra Nayaka, S.; Archana, B.; Lakshmeesha, T. R.; Niranjana, S. R.; Lund, O. S.; Prakash, H. S. Specific PCR-based detection of *Phomopsis vexans* the cause of leaf blight and fruit rot pathogen of *Solanum melongena* L. *Lett. Appl. Microbiol.*, **2019**, *69(5)*, 358-365. https://doi.org/10.1111/lam.13214
135. Islam, M. M.; Alam, K. M.; Momtaz, R.; Arifunnahar, M.; Karim, M. M. Molecular Characterization of *Phomopsis* blight and fruit rot resistant and susceptible cultivars of eggplant. *Int. J. Res. Stud. Biosci.*, **2020**, *8(7)*, 21-27. I: https://doi.org/10.20431/2349-0365.0807004
136. Kashyap, P. L.; Kumar, S.; Kumar, R. S.; Tripathi, R.; Sharma, P.; Sharma, A.; Sharma, A.; Jasrotia, P.; Singh, G. P. Identification of novel microsatellite markers to assess the population structure and genetic differentiation of *Ustilago hordei* causing covered smut of barley. *Front. Microbiol.*, **2020**, *10*, 2929. https://doi.org/10.3389/fmicb.2019.02929
137. Liu, J. X., Cai, Y. N., Jiang, W. Y., Li, Y. G., Zhang, Q. F., Pan, H. Y. Population structure and genetic diversity of fungi causing rice seedling blight in Northeast China based on microsatellite markers. *Plant Dis.*, **2020**, *104(3)*, 868-874. https://doi.org/10.1094/PDIS-08-19-1620-RE
138. Savadi, S.; Prasad, P.; Bhardwaj, S. C.; Rathore, R.; Sharma, K.; Lal Kashyap, P.; Gangwar, O. P.; Kumar, S. Development and characterization of novel microsatellite markers in *Puccinia striiformis f. sp. tritici* and their transferability in *Puccinia* species. *J. Phytopathol.*, **2020**, *168(2)*, 120-128. https://doi.org/10.1111/jph.12876
139. Tafifet, L.; Raio, A.; Holeva, M. C.; Dikhai, R.; Kouskoussa, C. O.; Cesbron, S.; Krimi, Z. Molecular characterization of Algerian *Erwinia amylovora* strains by VNTR analysis and biocontrol efficacy of *Bacillus* spp. and *Pseudomonas brassicacearum* antagonists. *Eur. J. Plant Pathol.*, **2020**, *156(3)*, 867-883. https://doi.org/10.1007/s10658-020-01938-6
140. Bich, G. A.; Castrillo, M. L.; Kramer, F. L.; Villalba, L. L.; Zapata, P. D. Morphological and molecular identification of entomopathogenic fungi from agricultural and forestry crops. *Floresta Ambient.*, **2021**, *28(2)*. https://doi.org/10.1590/2179-8087-FLORAM-2018-0086
141. Czajowski, G.; Kosman, E.; Słowacki, P.; Park, R. F.; Czembor, P. Assessing new SSR markers for utility and informativeness in genetic studies of brown rust fungi on wheat, triticale, and rye. *Plant Pathol.*, **2021**, *70(5)*, 1110-1122. https://doi.org/10.1111/ppa.13347
142. Iwakiri, A.; Sakaue, D.; Matsushita, N.; Fukuda, K. New microsatellite markers for the population studies of *Racodium therryanum*, a causal agent of snow blight in Japan. *For. Pathol.*, **2021**, *51(2)*, e12666. https://doi.org/10.1111/efp.12666
143. Daverdin, G.; Rouxel, T.; Gout, L.; Aubertot, J. N.; Fudal, I.; Meyer, M.; Parlange, F.; Carpezat, J.; Balesdent, M. H. Genome structure and reproductive behaviour influence the evolutionary potential of a fungal phytopathogen. *PLoS Pathog.*, **2012**, *8(11)*, e1003020. https://doi.org/10.1371/journal.ppat.1003020
144. Dilmaghani, A.; Gout, L.; Moreno-Rico, O.; Dias, J. S.; Coudard, L.; Castillo-Torres, N.; Balesdent, M.-H.; Rouxel, T. Clonal populations of *Leptosphaeria maculans* contaminating cabbage in Mexico. *Plant Pathol.*, **2013**, *62(3)*, 520-532. https://doi.org/10.1111/j.1365-3059.2012.02668.x
145. Nepal, A. Genetic structure of *Leptosphaeria maculans* populations in North Dakota and identification of genes associated with resistance to *L. maculans* in *Brassica juncea*. Doctoral Thesis, North Dakota State University, **2013**
146. Kaczmarek, J.; Latunde-Dada, A. O.; Irzykowski, W.; Cools, H. J.; Stonard, J. F.; Brachaczek, A.; Jedryczka, M. Molecular screening for avirulence alleles AvrLm1 and AvrLm6 in airborne inoculum of *Leptosphaeria maculans* and winter oilseed rape (Brassica napus) plants from Poland and the UK. *J. App. Genet.*, **2014**, *55(4)*, 529-539. https://doi.org/10.1007/s13353-014-0235-8

147. Franceschi Fernández, J. Phenotypic characterization of *Leptosphaeria maculans* pathogenicity groups aggressiveness on *Brassica napus*. Master of Science Thesis. North Dakota State University, **2015**
148. Thiéry, O.; Moora, M.; Vasar, M.; Zobel, M.; Öpik, M. Inter-and intrasporal nuclear ribosomal gene sequence variation within one isolate of arbuscular mycorrhizal fungus, *Diversispora* sp. *Symbiosis*, **2012**, *58(1)*, 135-147. https://doi.org/10.1007/s13199-012-0212-0
149. Guzow-Krzemińska, B.; Stocker-Wörgötter, E. Development of microsatellite markers in *Protoparmeliopsis muralis* (lichenized Ascomycete)–a common lichen species. *Lichenol.*, **2013**, *45(6)*, 791-798. https://doi.org/10.1017/S002428291300042X
150. Kumar, S.; Rai, S.; Maurya, D. K.; Kashyap, P. L.; Srivastava, A. K.; Anandaraj, M. Cross-species transferability of microsatellite markers from *Fusarium oxysporum* for the assessment of genetic diversity in *Fusarium udum*. Phytoparasitica, **2013**, *41(5)*, 615-622. https://doi.org/10.1007/s12600-013-0324-y
151. LeClere, A. R.; Yang, J. K.; Kirkpatrick, D. T. The role of CSM3, MRC1, and TOF1 in minisatellite stability and large loop DNA repair during meiosis in yeast. *Fungal Genet. Biol.*, **2013**, *50*, 33-43. https://doi.org/10.1016/j.fgb.2012.10.007
152. Li, Z.; Feng, Z.; Zhao, L.; Shi, Y.; Li, C.; Wang, L.; Liu. Y.; Zhu, H. Frequency and distribution of microsatellites in whole genome of *Verticillium albo-atrum*. *Cotton Sci.*, **2013**, *25(2)*, 135-141. ISSN : 1002-7807
153. Queiroz, C. B.; Miranda, E. C.; Hanada, E.; Sousa, N. R.; Gasparotto, L.; Soares, M. A.; Silva, G. F. Distribution of mating-type alleles and M13 PCR markers in the black leaf spot fungus *Mycosphaerella fijiensis* of bananas in Brazil. *Genet. Mol. Res.*, **2013**, *12(1)*, 443-452. https://doi.org/10.4238/2013.February.8.8
154. Youssef, N. H.; Couger, M. B.; Struchtemeyer, C. G.; Liggenstoffer, A. S.; Prade, R. A.; Najar, F. Z.; Atiyeh, H. K.; Wilkins, M. R.; Elshahed, M. S. The genome of the anaerobic fungus *Orpinomyces* sp. strain C1A reveals the unique evolutionary history of a remarkable plant biomass degrader. *App. Environ. Microbiol.*, **2013**, *79(15)*, 4620-4634. https://doi.org/10.1128/AEM.00821-13
155. Chadha, S.; Sharma, M. Transposable elements as stress adaptive capacitors induce genomic instability in fungal pathogen *Magnaporthe oryzae*. *PLoS One*, **2014**, *9(4)*, e94415. https://doi.org/10.1371/journal.pone.0094415
156. Chen, X.; Yan, Q.; Li, R.; Li, K.; Wei, S.; Huang, F.; Wei, L. Physiological races identification and genetic diversity analysis of *Magnaporthe oryzae* in Guangxi Province. *Southwest China Journal of Agricultural Sciences*, **2017**, *30(4)*, 767-772. ISSN : 1001-4829
157. Seifollahi, E.; Fotouhifar, K. B.; Javan-Nikkhah, M. Genetic diversity of *Valsa malicola* isolates assessed by microsatellite-primed PCR (MP-PCR). *Arch. Phytopathol. Pflanzenschutz*, **2014**, *47(16)*, 2003-2013. https://doi.org/10.1080/03235408.2013.866433
158. Wang, D. Y.; Gricourt, M.; Arné, P.; Thierry, S.; Seguin, D.; Chermette, R.; Huang, W. Y.; Dannaoui, E.; Botterel, F.; Guillot, J. Mutations in the Cyp51A gene and susceptibility to itraconazole in *Aspergillus fumigatus* isolated from avian farms in France and China. *Poult. Sci.*, **2014**, *93(1)*, 12-15. https://doi.org/10.3382/ps.2013-03541
159. Badouin, H.; Hood, M. E.; Gouzy, J.; Aguileta, G.; Siguenza, S.; Perlin, M. H.; Cuomo, C. A.; Fairhead, C.; Branca, A.; Giraud, T. Chaos of rearrangements in the mating-type chromosomes of the anther-smut fungus *Microbotryum lychnidis-dioicae*. Genetics, **2015**, *200(4)*, 1275-1284. https://doi.org/10.1534/genetics.115.177709
160. Cheng, J.; Kang, Z.; Huang, L.; Wang, M.; Wan, A.; Cheng, P. Molecular identification of somatic recombination of stripe rust pathogen (*Puccinia striiformis*) in the greenhouse. *Mycosystema*, **2015**, *34(6)*, 1128-1142. ISSN : 1672-6472
161. da S Machado, P.; Alfenas, A. C.; Alfenas, R. F.; Mohammed, C. L.; Glen, M. Microsatellite analysis indicates that *Puccinia psidii* in Australia is mutating but not recombining. *Australas. Plant Pathol.*, **2015**, *44(4)*, 455-462. https://doi.org/10.1007/s13313-015-0364-5
162. Durkin, J.; Bissett, J.; Pahlavani, M.; Mooney, B.; Buchwaldt, L. IGS minisatellites useful for race differentiation in *Colletotrichum lentis* and a likely site of small RNA synthesis affecting pathogenicity. *PloS One*, **2015**, *10(9)*, e0137398. https://doi.org/10.1371/journal.pone.0137398
163. Henry, J. L. Mating-type Locus characterization and variation in *Pyrenophora semeniperda*. Master Degree Thesis. Brigham Young University, **2015**.
164. Houshyarfard, M.; Rouhani, H.; Falahati-Rastegar, M.; Malekzadeh-Shafaroudi, S.; Mahdikhani-Moghaddam, E. Characterization of Iranian nonaflatoxigenic strains of *Aspergillus flavus* based on microsatellite-primed PCR. *Mol. Biol. Res. Commun.*, **2015**, *4(1)*, 43. PMID: 27843995
165. Schirrmann, M. K.; Zoller, S.; Fior, S.; Leuchtmann, A. Genetic evidence for reproductive isolation among sympatric *Epichloë* endophytes as inferred from newly developed microsatellite markers. *Microb. Ecol.*, **2015**, *70(1)*, 51-60. https://doi.org/10.1007/s00248-014-0556-5
166. Mironenko, N. V.; Baranova, O. A.; Kovalenko, N. M.; Mikhailova, L. A.; Rosseva, L. P. Genetic structure of the Russian populations of Pyrenophora tritici-repentis, determined by using microsatellite markers. *Russ. J. Genet.*, **2016**, *52(8)*, 771-779. https://doi.org/10.1134/S1022795416080093
167. Zhang, Y. J.; Hou, J. X.; Zhang, S.; Hausner, G.; Liu, X. Z.; Li, W. J. The intronic minisatellite OsMin1 within a serine protease gene in the Chinese caterpillar fungus *Ophiocordyceps sinensis*. *App. Microbiol. Biotechnol.*, **2016**, *100(8)*, 3599-3610. https://doi.org/10.1007/s00253-016-7287-0
168. Hamberg, L.; de la Bastide, P.; Hintz, W.; Shamoun, S. F.; Brandtberg, M.; Hantula, J. Interfertility and genetic variability among European and North American isolates of the basidiomycete fungus *Chondrostereum purpureum*. *Fungal Biol.*, **2018**, *122(7)*, 659-667. https://doi.org/10.1016/j.funbio.2018.03.009
169. Przemieniecki, S. W.; Kurowski, T. P.; Korzekwa, K. Chemotypes and geographic distribution of the *Fusarium graminearum* species complex. *Environ. Biotechnol.*, **2014**, *10(2)*, 45-59. https://doi.org/10.14799/ebms241
170. Ruiz-Jiménez, A. L.; Ruiz-Sánchez, E.; Heredia, G.; Tapia-Tussell, R.; González-Coloma, A.; Peraza-Jiménez, K.; Moo-Koh, F. A.; Medina-Baizabal, I. L.; Hernández-Romero, Y.; Mena-Rejón, G. J.; Quijano-Quiñones, R. F.; Gamboa-Angulo, M.

Identification of insect-deterrent metabolites from *Acremonium masseei* strain CICY026, a saprophytic fungus from a Sinkhole in Yucatán. *Microorganisms*, **2019**, *7(12)*, 712. https://doi.org/10.3390/microorganisms7120712
171. Semenov, A. N.; Divashuk, M. G.; Bazhenov, M. S.; Karlov, G. I.; Leunov, V. I.; Khovrin, A. N.; Egorova, A.A.; Sokolova, L.M.; Tereshonkova, T.A.; Alekseeva, K.L.; Leunova, V. M. Comparative analysis of polymorphism of microsatellite markers in several species of *Fusarium*. *Izvestiya of Timiryazev Agricultural Academy*, **2016**, *1*, 40-50.
172. Zou, Z.; Zhang, X.; Fernando, W. G. D. Distribution of mating-type alleles and genetic variability in field populations of *Leptosphaeria maculans* in western Canada. *J. Phytopathol.*, **2018**, *166(6)*, 438-447. https://doi.org/10.1111/jph.12706
173. Choi, M. J.; Won, E. J.; Joo, M. Y.; Park, Y. J.; Kim, S. H.; Shin, M. G.; Shin, J. H. Microsatellite typing and resistance mechanism analysis of voriconazole-resistant *Aspergillus flavus* isolates in South Korean hospitals. *Antimicrob. Agents Chemother.*, **2019**, *63(2)*, e01610-18. https://doi.org/10.1128/AAC.01610-18
174. Ahangarkani, F.; Badali, H.; Abbasi, K.; Nabili, M.; Khodavaisy, S.; de Groot, T.; Meis, J. F. Clonal expansion of environmental triazole resistant *Aspergillus fumigatus* in Iran. *J. Fungi*, **2020**, *6(4)*, 199. https://doi.org/10.3390/jof6040199
175. Degtjarenko, P.; Mark, K.; Moisejevs, R.; Himelbrant, D.; Stepanchikova, I.; Tsurykau, A.; Randlane, T.; Scheidegger, C. Low genetic differentiation between apotheciate *Usnea florida* and sorediate *Usnea subfloridana* (Parmeliaceae, Ascomycota) based on microsatellite data. *Fungal Biol.*, **2020**, *124(10)*, 892-902. https://doi.org/10.1016/j.funbio.2020.07.007
176. Jeanvoine, A.; Godeau, C.; Laboissière, A.; Reboux, G.; Millon, L.; Rocchi, S. Molecular epidemiology of azole-resistant *Aspergillus fumigatus* in sawmills of eastern France by microsatellite genotyping. *J. Fungi*, **2020**, *6(3)*, 120. https://doi.org/10.3390/jof6030120
177. Lee, D. H.; Wingfield, B. D.; Roux, J.; Wingfield, M. J. Quantification of outcrossing events in haploid fungi using microsatellite markers. *J. Fungi*, **2020**, *6(2)*, 48. https://doi.org/10.3390/jof6020048
178. Trabelsi, H.; Hadrich, I.; Neji, S.; Khemakhem, N.; Hammami, B.; Makni, F.; Sellami, H.; Ayadi, A. Microsatellite analysis of the population structure in *Rhizopus arrhizus*. *J. App. Microbiol.*, **2020**, *128(6)*, 1793-1801. https://doi.org/10.1111/jam.14583
179. Kaur, R. Mating Incompatibility genes in fungal pathogen *Colletotrichum lentils*. Doctoral Thesis, University of Saskatchewan, 2021
180. Foulongne-Oriol, M.; Murat, C.; Castanera, R.; Ramírez, L.; Sonnenberg, A. S. Genome-wide survey of repetitive DNA elements in the button mushroom *Agaricus bisporus*. *Fungal Gen. Biol.*, **2013**, *55*, 6-21. https://doi.org/10.1016/j.fgb.2013.04.003
181. Molinier, V.; Murat, C.; Morin, E.; Gollotte, A.; Wipf, D.; Martin, F. First identification of polymorphic microsatellite markers in the Burgundy truffle, *Tuber aestivum* (Tuberaceae). *App. Plant Sci.*, **2013**, *1(2)*, 1200220. https://doi.org/10.3732/apps.1200220
182. Salvador, C.; Martins, M. R.; Arteiro, J. M.; Caldeira, A. T. Molecular evaluation of some *Amanita ponderosa* and the fungal strains living in association with these mushrooms in the southwestern Iberian Peninsula. *Ann. Microbiol.*, **2014**, *64(3)*, 1179-1187. https://doi.org/10.1007/s13213-013-0757-z
183. Wang, Y.; Chen, M.; Wang, H.; Wang, J. F.; Bao, D. Microsatellites in the genome of the edible mushroom, *Volvariella volvacea*. *BioMed Res. Int.*, **2014**, *2014*. https://doi.org/10.1155/2014/281912
184. Canonico, L.; Comitini, F.; Ciani, M. TdPIR minisatellite fingerprinting as a useful new tool for *Torulaspora delbrueckii* molecular typing. *Int. J. Food Microbiol.*, **2015**, *200*, 47-51. https://doi.org/10.1016/j.ijfoodmicro.2015.01.020
185. Banilas, G.; Sgouros, G.; Nisiotou, A. Development of microsatellite markers for *Lachancea thermotolerans* typing and population structure of wine-associated isolates. *Microbiol. Res.*, **2016**, *193*, 1-10. https://doi.org/10.1016/j.micres.2016.08.010
186. Rokni, N.; Goltapeh, E. M.; Shafeinia, A.; Safaie, N. Evaluation of genetic diversity among some commercial cultivars and Iranian wild strains of *Agaricus bisporus* by microsatellite markers. *Botany*, **2016**, *94(1)*, 9-13. https://doi.org/10.1139/cjb-2015-0131
187. Baik, S. H.; Song, Y. R.; Song, N. E.; Lim, B. W.; Lee, C. M.; Choi, S. Y.; Lee, D. B. Distribution of autochthonous yeast strains isolated from different regional Makgeolli, a Korean traditional fermented rice wine and their biochemical and genetical characterization. Proceedings of the Korean Society of Microbiology Conference, 2017, 88-88
188. Burlakoti, R. R.; Tamburic-Ilincic, L.; Limay-Rios, V.; Burlakoti, P. Comparative population structure and trichothecene mycotoxin profiling of *Fusarium graminearum* from corn and wheat in Ontario, central Canada. *Plant Pathol.*, **2017**, *66(1)*, 14-27. https://doi.org/10.1111/ppa.12559
189. Kurokochi, H.; Zhang, S.; Takeuchi, Y.; Tan, E.; Asakawa, S.; Lian, C. Local-level genetic diversity and structure of matsutake mushroom (*Tricholoma matsutake*) populations in Nagano Prefecture, Japan, Revealed by 15 microsatellite markers. *J. Fungi*, **2017**, *3(2)*, 23. https://doi.org/10.3390/jof3020023
190. Mang, S. M.; Zotta, T.; Camele, I.; Racioppi, R.; D'Auria, M.; Rana, G. L. Morphological, physico-chemical and molecular investigations on *Tuber bellonae* from Basilicata-Italy. *J. Anim. Plant Sci.*, **2017**, *27*, 528-541. ISSN: 1018-7081
191. Kim, S.; Song, Y.; Ha, B.; Moon, Y. J.; Kim, M.; Ryu, H.; Ro, H. S. Variable number tandem repeats in the mitochondrial DNA of *Lentinula edodes*. Genes, **2019**, *10(7)*, 542. https://doi.org/10.3390/genes10070542
192. Owati, A., Agindotan, B.; Burrows, M. First microsatellite markers developed and applied for the genetic diversity study and population structure of *Didymella pisi* associated with ascochyta blight of dry pea in Montana. *Fungal Biol.*, **2019**, *123(5)*, 384-392. https://doi.org/10.1016/j.funbio.2019.02.004
193. Uncu, A. Ö.; Uncu, A. T. Genome-wide identification and annotation of microsatellite markers in white truffle (*Tuber magnatum*). *Mediterr. Agric. Sci.*, **2019**, *32(1)*, 31-34. https://doi.org/10.29136/mediterranean.487250
194. Varady, E. S.; Bodaghi, S.; Vidalakis, G.; Douhan, G. W. Microsatellite characterization and marker development for the fungus *Penicillium digitatum*, causal agent of green mold of citrus. *Microbiologyopen*, **2019**, *8(7)*, e00788. https://doi.org/10.1002/mbo3.788
195. Wang, L. N.; Wei, G. A. O., Wang, Q. Y.; Qu, J. B., Zhang, J. X.; Huang, C. Y. Identification of commercial cultivars of *Agaricus bisporus* in China using genome-wide microsatellite markers. *J. Integr. Agric.*, **2019**, *18(3)*, 580-589. https://doi.org/10.1016/S2095-3119(18)62126-4

196. Tantikachornkiat, M.; Morgan, S. C.; Lepitre, M.; Cliff, M. A.; Durall, D. M. Yeast and bacterial inoculation practices influence the microbial communities of barrel-fermented Chardonnay wines. *Aust. J. Grape Wine Res.*, **2020**, *26(3)*, 279-289. https://doi.org/10.1111/ajgw.12438
197. Meng, X.; Wang, G. S.; Wu, G.; Wang, P. M.; Yang, Z. L.; Li, Y. C. The genus *Leccinum* (Boletaceae, Boletales) from China Based on morphological and molecular data. *J. Fungi*, **2021**, *7(9)*, 732. https://doi.org/10.3390/jof7090732
198. Devkota, S.; Cornejo, C.; Werth, S.; Chaudhary, R. P.; Scheidegger, C. Characterization of microsatellite loci in the Himalayan lichen fungus *Lobaria pindarensis* (Lobariaceae). *App. Plant Sci.*, **2014**, *2(5)*, 1300101. https://doi.org/10.3732/apps.1300101
199. Prieto, M.; Romera, L.; Merinero, S.; Aragón, G.; Martínez, I. Development and characterization of fungal specific microsatellite markers in the lichen *Lobarina scrobiculata* (Lobariaceae, Ascomycota). *Lichenol.*, **2015**, *47(3)*, 183-186. https://doi.org/10.1017/S0024282915000109
200. Lindgren, H.; Leavitt, S. D.; Lumbsch, T. Characterization of microsatellite markers in the cosmopolitan lichen-forming fungus *Rhizoplaca melanophthalma* (Lecanoraceae). *MycoKeys*, **2016**, *14*, 31. https://doi.org/10.3897/mycokeys.14.9729
201. Nadyeina, O.; Zarabska-Bozejewicz, D.; Wiedmer, A.; Cornejo, C.; Scheidegger, C. Polymorphic fungus-specific microsatellite markers of *Bactrospora dryina* reveal multiple colonizations of trees. *Lichenol.*, **2017**, *49(6)*, 561-577. https://doi.org/10.1017/S0024282917000548
202. Lagostina, E.; Dal Grande, F.; Andreev, M.; Printzen, C. The use of microsatellite markers for species delimitation in Antarctic *Usnea* subgenus *Neuropogon*. *Mycologia*, **2018**, *110(6)*, 1047-1057. https://doi.org/10.1017/S0024282917000548
203. Degtjarenko, P.; Jüriado, I.; Mandel, T.; Tõrra, T.; Saag, A.; Scheidegger, C.; Randlane, T. Microsatellite based genetic diversity of the widespread epiphytic lichen *Usnea subfloridana* (Parmeliaceae, Ascomycota) in Estonia: comparison of populations from the mainland and an island. *MycoKeys*, **2019**, *58*, 27. https://doi.org/10.3897/mycokeys.58.36557
204. Werth, S.; Meidl, P.; Scheidegger, C. Deep divergence between island populations in lichenized fungi. *Sci. Rep.*, **2021**, *11(1)*, 1-13. https://doi.org/10.1038/s41598-021-86448-z
205. Foulongne-Oriol, M.; Spataro, C.; Moinard, M.; Cabannes, D.; Callac, P.; Savoie, J. M. Development of polymorphic microsatellite markers issued from pyrosequencing technology for the medicinal mushroom *Agaricus subrufescens*. *FEMS Microbiol. Lett.*, **2012**, *334(2)*, 119-126. https://doi.org/10.1111/j.1574-6968.2012.02627.x
206. Shnyreva, A. V.; Shnyreva, A. A. Intraspecies genetic variability, mating compatibility and clonality in two medicinal mushrooms, *Ganoderma applanatum* and *Fomitopsis pinicola*. In The 9th International Medicinal Mushrooms Conference (pp. 24-29), 2017.
207. Wentzel, L. C. P.; Inforsato, F. J.; Montoya, Q. V.; Rossin, B. G.; Nascimento, N. R.; Rodrigues, A.; Sette, L. D. Fungi from admiralty bay (King George Island, Antarctica) soils and marine sediments. *Microb. Ecol.*, **2019**, *77(1)*, 12-24. https://doi.org/10.1007/s00248-018-1217-x
208. Beiler, K. J.; Simard, S. W.; LeMay, V.; Durall, D. M. Vertical partitioning between sister species of *Rhizopogon* fungi on mesic and xeric sites in an interior Douglas-fir forest. *Mol. Ecol.*, **2012**, *21(24)*, 6163-6174. https://doi.org/10.1111/mec.12076
209. Thompson, T. A.; Thorn, R. G.; Smith, K. T. *Hypholoma lateritium* isolated from coarse woody debris, the forest floor, and mineral soil in a deciduous forest in New Hampshire. *Botany*, **2012**, *90(6)*, 457-464. https://doi.org/10.1139/b2012-011
210. Vohnik, M.; Sadowsky, J. J.; Kohout, P.; Lhotakova, Z.; Nestby, R.; Kolařík, M. Novel root-fungus symbiosis in Ericaceae: sheathed ericoid mycorrhiza formed by a hitherto undescribed basidiomycete with affinities to Trechisporales. *PLoS One*, **2012**, *7(6)*, e39524. https://doi.org/10.1371/journal.pone.0039524
211. Dunham, S. M.; Mujic, A. B.; Spatafora, J. W.; Kretzer, A. M. Within-population genetic structure differs between two sympatric sister-species of ectomycorrhizal fungi, *Rhizopogon vinicolor* and *R. vesiculosus*. *Mycol.*, **2013**, *105(4)*, 814-826. https://doi.org/10.3852/12-265
212. Ruibal, M. P.; Peakall, R.; Smith, L. M.; Linde, C. C. Phylogenetic and microsatellite markers for *Tulasnella* (Tulasnellaceae) mycorrhizal fungi associated with Australian orchids. *Appl. Plant Sci.*, **2013**, *1(3)*, 1200394. https://doi.org/10.3732/apps.1200394
213. Ciesielska, A.; Bohacz, J.; Korniłłowicz-Kowalska, T.; Staczek, P. Microsatellite-primed PCR for intra-species genetic relatedness in *Trichophyton ajelloi* strains isolated in Poland from various soil samples. *Microbes Environ.*, **2014**, ME13160. https://doi.org/10.1264/jsme2.ME13160
214. Grubisha, L. C., Brewer, J. D., Dowie, N. J., Miller, S. L., Trowbridge, S. M., Klooster, M. R. Microsatellite primers for the fungi *Rhizopogon kretzerae* and *R. salebrosus* (Rhizopogonaceae) from 454 shotgun pyrosequencing. *Appl. Plant Sci.*, **2014**, *2(7)*, 1400029. https://doi.org/10.3732/apps.1400029
215. Sheedy, E. M.; Van de Wouw, A. P.; Howlett, B. J.; May, T. W. Mitochondrial microsatellite markers for the Australian ectomycorrhizal fungus *Laccaria* sp. A (Hydnangiaceae). *App. Plant Sci.*, **2014**, *2(3)*, 1300086. https://doi.org/10.3732/apps.1300086
216. Wadud, M.; Nara, K.; Lian, C.; Ishida, T. A.; Hogetsu, T. Genet dynamics and ecological functions of the pioneer ectomycorrhizal fungi *Laccaria amethystina* and *Laccaria laccata* in a volcanic desert on Mount Fuji. *Mycorrhiza*, **2014**, *24(7)*, 551-563. https://doi.org/10.1007/s00572-014-0571-x
217. Rivera, Y.; Kretzer, A. M.;Horton, T. R. New microsatellite markers for the ectomycorrhizal fungus *Pisolithus tinctorius sensu stricto* reveal the genetic structure of US and Puerto Rican populations. *Fungal Ecol.*, **2015**, *13*, 1-9. https://doi.org/10.1016/j.funeco.2014.07.001
218. Peter, M.; Kohler, A.; Ohm, R. A.; Kuo, A.; Krützmann, J.; Morin, E.; Arend, M.; Barry, K. W.; Binder, M.; Choi, C.; Clum, A.; Copeland, A.; Grisel, N.; Haridas, S.; Kipfer, T.; LaButti, K.; Lindquist, E.; Lipzen, A.; Maire, R.; Meier, B.; Mihaltcheva, S.; Molinier, V.; Murat, C.; Pöggeler, S.; Quandt, C. A.; Sperisen, C.; Tritt, A.; Tisserant, E.; Crous, P. W.; Henrissat, B.; Nehls, U.; Egli, S.; Spatafora, J. W.; Grigoriev, I. V.; Martin, F. M.. Ectomycorrhizal ecology is imprinted in the genome of the dominant symbiotic fungus *Cenococcum geophilum*. *Nat. Comm.*, **2016**, *7(1)*, 1-15. https://doi.org/10.1038/ncomms12662

219. Colancecco, R. Diversity of *Aspergillus flavus* isolates from corn and soil in three different Tuscany areas treated with the biological product AF-X1. Master Degree Thesis. University of Pisa, 2018
220. Stroheker, S.; Dubach, V.; Vögtli, I.; Sieber, T. N. Investigating host preference of root endophytes of three European tree species, with a focus on members of the *Phialocephala fortinii—Acephala applanata* species complex (PAC). *J. Fungi*, **2021**, *7(4)*, 317. https://doi.org/10.3390/jof7040317
221. Silva-Bedoya, L. M.; Ramírez-Castrillón, M.; Osorio-Cadavid, E. Yeast diversity associated to sediments and water from two Colombian artificial lakes. Braz. *J. Microbiol.*, **2014**, *45(1)*, 135-142. https://doi.org/10.1590/S1517-83822014005000035
222. McTaggart, L. R.; Brown, E. M.; Richardson, S. E. Phylogeographic analysis of *Blastomyces dermatitidis* and *Blastomyces gilchristii* reveals an association with North American freshwater drainage basins. *PLoS One*, **2016**, *11(7)*, e0159396. https://doi.org/10.1371/journal.pone.0159396
223. Gonçalves, M. F; Santos, L.; Silva, B. M.; Abreu, A. C.; Vicente, T. F.; Esteves, A. C.; Alves, A. Biodiversity of *Penicillium* species from marine environments in Portugal and description of *Penicillium lusitanum* sp. nov., a novel species isolated from sea water. *Int. J. Syst. Evol. Microbiol.*, **2019**, *69(10)*, 3014-3021. https://doi.org/10.1371/journal.pone.0159396
224. Gonçalves, M. F.; Esteves, A. C.; Alves, A. Revealing the hidden diversity of marine fungi in Portugal with the description of two novel species, *Neoascochyta fuci* sp. nov. and *Paraconiothyrium salinum* sp. nov. *Int. J. Syst. Evol. Microbiol.*, **2020**, *70(10)*, 5337-5354. https://doi.org/10.1099/ijsem.0.004410
225. Gonçalves, M. F.; Abreu, A. C.; Hilário, S.; Alves, A. Diversity of marine fungi associated with wood baits in the estuary Ria de Aveiro, with descriptions of *Paralulworthia halima*, comb. nov., *Remispora submersa*, sp. nov., and *Zalerion pseudomaritima*, sp. nov. *Mycol.*, **2021**, *113(3)*, 664-683. https://doi.org/10.1080/00275514.2021.1875710
226. Vicente, T. F.; Gonçalves, M. F.; Brandão, C.; Fidalgo, C.; Alves, A. Diversity of fungi associated with macroalgae from an estuarine environment and description of *Cladosporium rubrum* sp. nov. and *Hypoxylon aveirense* sp. nov. *Int. J. Syst. Evol. Microbiol.*, **2021**, *71(2)*, 004630. https://doi.org/10.1099/ijsem.0.004630

References: Supplementary Table S6

1. Caramalho, R.; Gusmao, L.; Lackner, M.; Amorim, A.; Araujo, R. SNaPAfu: a novel single nucleotide polymorphism multiplex assay for *Aspergillus fumigatus* direct detection, identification and genotyping in clinical specimens. *PLoS One*, **2013**, *8(10)*, e75968. https://doi.org/10.1371/journal.pone.0075968
2. Oliveira, M.; Lackner, M.; Amorim, A.; Araujo, R. Feasibility of mitochondrial single nucleotide polymorphisms to detect and identify *Aspergillus fumigatus* in clinical samples. *Diagn. Microbiol. Infect. Dis.*, **2014**, *80(1)*, 53-58. https://doi.org/10.1016/j.diagmicrobio.2014.05.007
3. Teixeira, J.; Amorim, A.; Araujo, R. Recombination detection in *Aspergillus fumigatus* through single nucleotide polymorphisms typing. *Environ. Microbiol. Rep.*, **2015**, *7(6)*, 881-886. https://doi.org/10.1111/1758-2229.12321
4. Brown, E. M.; McTaggart, L. R.; Zhang, S. X.; Low, D. E.; Stevens, D. A.; Richardson, S. E. Phylogenetic analysis reveals a cryptic species *Blastomyces gilchristii*, sp. nov. within the human pathogenic fungus *Blastomyces dermatitidis*. *PLoS One*, **2013**, *8(3)*, e59237. https://doi.org/10.1371/journal.pone.0059237
5. Hamzehei, H.; Yazdanparast, S. A.; Mohammad Davoudi, M.; Khodavaisy, S.; Golehkheyli, M.; Ansari, S.; de Hoog, G. S.; Badali, H. Use of rolling circle amplification to rapidly identify species of *Cladophialophora* potentially causing human infection. *Mycopathologia*, **2013**, *175(5)*, 431-438. https://doi.org/10.1007/s11046-013-9630-7
6. Najafzadeh, M. J.; Dolatabadi, S.; Keisari, M. S.; Naseri, A.; Feng, P.; De Hoog, G. S. Detection and identification of opportunistic *Exophiala* species using the rolling circle amplification of ribosomal internal transcribed spacers. *J. Microbiol. Methods*, **2013**, *94(3)*, 338-342. https://doi.org/10.1016/j.mimet.2013.06.026
7. Litvintseva, A. P.; Hurst, S.; Gade, L.; Frace, M. A.; Hilsabeck, R.; Schupp, J. M.; Gillece, J. D.; Roe, C.; Smith, D.; Keim, P.; Lockhart, S. R.; Changayil, S.; Weil, M. R.; MacCannell, D. R.; Brandt, M. E.; Engelthaler, D. M. Whole-genome analysis of *Exserohilum rostratum* from an outbreak of fungal meningitis and other infections. *J. Clin. Microbiol.*, **2014**, *52(9)*, 3216-3222. https://doi.org/10.1128/JCM.00936-14
8. Muñoz, J. F.; Gallo, J. E.; Misas, E.; Priest, M.; Imamovic, A.; Young, S.; Zeng, Q.; Clay, O. K.; McEwen, J: G.; Cuomo, C. A. Genome update of the dimorphic human pathogenic fungi causing paracoccidioidomycosis. *PLOS Negl. Trop. Dis.*, **2014**, *8(12)*, e3348. https://doi.org/10.1371/journal.pntd.0003348
9. Muñoz, J. F.; Farrer, R. A.; Desjardins, C. A.; Gallo, J. E.; Sykes, S.; Sakthikumar, S.; Misas, E.; Whiston, E. A.; Bagagli, E.; Soares, C. M. A.; de M. Teixeira, M.; Taylor, J. W.; Clay, O. K.; McEwen, J. G.; Cuomo, C. A. Genome diversity, recombination, and virulence across the major lineages of *Paracoccidioides*. *MSphere*, **2016**, *1(5)*, e00213-16. https://doi.org/10.1128/mSphere.00213-16
10. Chibucos, M. C.; Soliman, S.; Gebremariam, T.; Lee, H.; Daugherty, S.; Orvis, J.; Shetty, A. C.; Crabtree, J.; Hazen, T. H.; Etienne, K. A.; Kumari, P.; O'Connor, T. D.; Rasko, D. A.; Filler, S. G.; Fraser, C. M.; Lockhart, S. R.; Skory, C. D.; Ibrahim, A. S.; Bruno, V. M. An integrated genomic and transcriptomic survey of mucormycosis-causing fungi. *Nat. Commun.*, **2016**, *7(1)*, 1-11. https://doi.org/10.1038/ncomms12218
11. Etienne, K. A.; Roe, C. C.; Smith, R. M.; Vallabhaneni, S.; Duarte, C.; Escandón, P.; Castañeda, E.; Gómez, B. L.; de Bedout, C.; López, L. F.; Salas, V.; Hederra, L. M.; Fernández, J.; Pidal, P.; Hormazabel, J. C. ; Otaíza-O'Ryan, F.; Vannberg, F. O.; Gillece, J.; Lemmer, D.; Driebe, E. M.; Engelthaler, D. M.; Litvintseva, A. P. Whole-genome sequencing to determine origin of multinational outbreak of *Sarocladium kiliense* bloodstream infections. *Emerg. Infect. Dis.*, **2016**, *22(3)*, 476. https://doi.org/10.3201/eid2203.151193

12. Fisher, C. E.; Hohl, T. M.; Fan, W.; Storer, B. E.; Levine, D. M.; Zhao, L. P.; Martin, P. J.; Warren, E. H.; Boeckh, M.; Hansen, J. A. Validation of single nucleotide polymorphisms in invasive aspergillosis following hematopoietic cell transplantation. *Blood*, **2017**, *129(19)*, 2693-2701. https://doi.org/10.1182/blood-2016-10-743294
13. Ballard, E.; Weber, J.; Melchers, W. J.; Tammireddy, S.; Whitfield, P. D.; Brakhage, A. A.; Brown, A. J. P.; Verweij, P. E.; Warris, A. Recreation of in-host acquired single nucleotide polymorphisms by CRISPR-Cas9 reveals an uncharacterised gene playing a role in *Aspergillus fumigatus* azole resistance via a non-cyp51A mediated resistance mechanism. *Fungal Genet. Biol.*, **2019**, *130*, 98-106. https://doi.org/10.1016/j.fgb.2019.05.005
14. Weber, J.; Valiante, V.; Nødvig, C. S.; Mattern, D. J.; Slotkowski, R. A.; Mortensen, U. H.; Brakhage, A. A. Functional reconstitution of a fungal natural product gene cluster by advanced genome editing. *ACS Synth. Biol.*, **2017**, *6(1)*, 62-68. https://doi.org/10.1021/acssynbio.6b00203
15. Rhodes, J.; Abdolrasouli, A.; Farrer, R. A.; Cuomo, C. A.; Aanensen, D. M.; Armstrong-James, D.; Fisher, M. C.; Schelenz, S. Genomic epidemiology of the UK outbreak of the emerging human fungal pathogen *Candida auris*. *Emerg. Microbes Infect.*, **2018**, *7(1)*, 1-1. https://doi.org/10.1038/s41426-018-0045-x
16. Chow, N. A.; de Groot, T.; Badali, H.; Abastabar, M.; Chiller, T. M.; Meis, J. F. Potential fifth clade of *Candida auris*, Iran, 2018. *Emerg. Infect. Dis.*, **2019**, *25(9)*, 1780. https://doi.org/10.3201/eid2509.190686
17. Safari, F.; Madani, M.; Badali, H.; Kargoshaie, A. A.; Fakhim, H.; Kheirollahi, M.; Meis, J. F.; Mirhendi, H. A chronic autochthonous fifth clade case of *Candida auris* otomycosis in Iran. *Mycopathologia*, **2022**, *187(1)*, 121-127. https://doi.org/10.1007/s11046-021-00605-6
18. Carreté, L.; Ksiezopolska, E.; Gómez-Molero, E.; Angoulvant, A.; Bader, O.; Fairhead, C.; Gabaldón, T. Genome comparisons of *Candida glabrata* serial clinical isolates reveal patterns of genetic variation in infecting clonal populations. *Front. Microbiol.*, **2019**, *10*, 112. https://doi.org/10.3389/fmicb.2019.00112
19. Gusa, A.; Jinks-Robertson, S. Mitotic recombination and adaptive genomic changes in human pathogenic fungi. *Genes*, **2019**, *10(11)*, 901. https://doi.org/10.3390/genes10110901
20. Pchelin, I. M.; Mochalov, Y. V.; Azarov, D. V.; Romanyuk, S. A.; Chilina, G. A.; Vybornova, I. V.; Bogdanova, T. V.; Zlatogursky, V. V.; Apalko, S. V.; Vasilyeva, N. V.; Taraskina, A. E. Genotyping of Russian isolates of fungal pathogen *Trichophyton rubrum*, based on simple sequence repeat and single nucleotide polymorphism. *Mycoses*, **2020**, *63(11)*, 1244-1254. https://doi.org/10.1111/myc.13162
21. Zhang, Y.; Qiao, M.; Xu, J.; Cao, Y.; Zhang, K. Q.; Yu, Z. F. Genetic diversity and recombination in natural populations of the nematode-trapping fungus *Arthrobotrys oligospora* from China. *Ecol. Evol.*, **2013**, *3(2)*, 312-325. https://doi.org/10.1002/ece3.450
22. Ojeda, D. I.; Dhillon, B.; Tsui, C. K.; Hamelin, R. C. Single-nucleotide polymorphism discovery in *Leptographium longiclavatum*, a mountain pine beetle-associated symbiotic fungus, using whole-genome resequencing. *Mol. Ecol. Resour.*, **2014**, *14(2)*, 401-410. https://doi.org/10.1111/1755-0998.12191
23. Ojeda Alayon, D. I.; Tsui, C. K.; Feau, N.; Capron, A.; Dhillon, B.; Zhang, Y.; Alamouti, S. M.; Boone, C. K.; Carroll, A. L.; Cooke, J. E. K.; Roe, A. D.; Sperling, F. A. H.; Hamelin, R. C. Genetic and genomic evidence of niche partitioning and adaptive radiation in mountain pine beetle fungal symbionts. *Mol. Ecol.*, **2017**, *26(7)*, 2077-2091. https://doi.org/10.1111/mec.14074
24. Tsui, C. K. M.; Beauseigle, S.; Ojeda Alayon, D. I.; Rice, A. V.; Cooke, J. E.; Sperling, F. A.; Roe, A. D.; Hamelin, R. C. Fine-scale genetic diversity and relatedness in fungi associated with the mountain pine beetle. *Can. J. For. Res.*, **2019**, *48(8)*, 933-941. https://doi.org/10.1139/cjfr-2018-0418
25. Shuey, M. M.; Drees, K. P.; Lindner, D. L.; Keim, P.; Foster, J. T. Highly sensitive quantitative PCR for the detection and differentiation of *Pseudogymnoascus destructans* and other *Pseudogymnoascus* species. *Appl. Environ. Microbiol.*, **2014**, *80(5)*, 1726-1731. https://doi.org/10.1128/AEM.02897-13
26. Talbot, J. J.; Johnson, L. R.; Martin, P.; Beatty, J. A.; Sutton, D. A.; Billen, F.; Halliday, C. L.; Gibson, J. S.; Kidd, S.; Steiner, J. M.; Ujvar, B.;Barrs, V. R. What causes canine sino-nasal aspergillosis? A molecular approach to species identification. *Vet. J.*, **2014**, *200(1)*, 17-21. https://doi.org/10.1016/j.tvjl.2014.01.009
27. Bohuski, E.; Lorch, J. M.; Griffin, K. M.; Blehert, D. S. TaqMan real-time polymerase chain reaction for detection of *Ophidiomyces ophiodiicola*, the fungus associated with snake fungal disease. *BMC Vet. Res.*, **2015**, *11(1)*, 1-10. https://doi.org/10.1186/s12917-015-0407-8
28. O'hanlon, S. J.; Rieux, A.; Farrer, R. A.; Rosa, G. M.; Waldman, B.; Bataille, A.; ; Fisher, M. C. Recent Asian origin of chytrid fungi causing global amphibian declines. *Science*, **2018**, *360(6389)*, 621-627. https://doi.org/1126/science.aar1965
29. Kobmoo, N.; Mongkolsamrit, S.; Arnamnart, N.; Luangsa-Ard, J. J.; Giraud, T. Population genomics revealed cryptic species within host-specific zombie-ant fungi (*Ophiocordyceps unilateralis*). *Mol. Phylogenet. Evol.*, **2019**, *140*, 106580. https://doi.org/10.1016/j.ympev.2019.106580
30. Rujirawat, T.; Sridapan, T.; Lohnoo, T.; Yingyong, W.; Kumsang, Y.; Sae-Chew, P.; Tonpitak, W.; Krajaejun, T. Single nucleotide polymorphism-based multiplex PCR for identification and genotyping of the oomycete *Pythium insidiosum* from humans, animals and the environment. *Infect. Genet. Evol.*, **2017**, *54*, 429-436. https://doi.org/10.1016/j.meegid.2017.08.004
31. Broders, K. D.; Boraks, A.; Sanchez, A. M.; Boland, G. J. Population structure of the butternut canker fungus, *Ophiognomonia clavigignenti-juglandacearum*, in North American forests. *Ecol. Evol.*, **2012**, *2(9)*, 2114-2127. https://doi.org/10.1002/ece3.332
32. Daverdin, G.; Rouxel, T.; Gout, L.; Aubertot, J. N.; Fudal, I.; Meyer, M.; Parlange, F.; Carpezat, J.; Balesdent, M. H. Genome structure and reproductive behaviour influence the evolutionary potential of a fungal phytopathogen. *PLoS Pathog.*, **2012**,*8(11)*, e1003020. https://doi.org/10.1371/journal.ppat.1003020

33. Van de Wouw, A. P.,; Lowe, R. G.; Elliott, C. E.; Dubois, D. J.; Howlett, B. J. An avirulence gene, AvrLmJ1, from the blackleg fungus, *Leptosphaeria maculans*, confers avirulence to *Brassica juncea* cultivars. *Mol. Plant Pathol.*, **2014**, *15(5)*, 523-530. https://doi.org/10.1111/mpp.12105
34. Ghanbarnia, K.; Fudal, I.; Larkan, N. J.; Links, M. G.; Balesdent, M. H.; Profotova, B.; Dilantha Fernando, W. G.; Rouxel, T.; Borhan, M. H. Rapid identification of the *Leptosphaeria maculans* avirulence gene AvrLm2 using an intraspecific comparative genomics approach. *Mol. Plant Pathol.*, **2015**, *16(7)*, 699-709. https://doi.org/10.1111/mpp.12228
35. Tollenaere, C.; Susi, H.; Nokso-Koivisto, J.; Koskinen, P.; Tack, A.; Auvinen, P.; Paulin, L.; Frilander, M. J.; Lehtonen, R. ; Laine, A. L.. SNP design from 454 sequencing of *Podosphaera plantaginis* transcriptome reveals a genetically diverse pathogen metapopulation with high levels of mixed-genotype infection. *PLoS One*, **2012**, *7(12)*, e52492. https://doi.org/10.1371/journal.pone.0052492
36. Chen, C.; Lian, B.; Hu, J.; Zhai, H.; Wang, X.; Venu, R. C.; Liu, E.; Wang, Z.; Chen, M.; Wang, B.; Wang, G.-L.; Wang, Z.; Mitchell, T. K. Genome comparison of two *Magnaporthe oryzae* field isolates reveals genome variations and potential virulence effectors. *BMC Genom.*, **2013**, *14(1)*, 1-12. https://doi.org/10.1186/1471-2164-14-887
37. Gowda, M.; Shirke, M. D.; Mahesh, H. B.; Chandarana, P.; Rajamani, A.; Chattoo, B. B. Genome analysis of rice-blast fungus *Magnaporthe oryzae* field isolates from southern India. *Genom. Data*, **2015**, *5*, 284-291. https://doi.org/10.1016/j.gdata.2015.06.018
38. Mutiga, S. K.; Rotich, F.; Ganeshan, V. D.; Mwongera, D. T.; Mgonja, E. M.; Were, V. M.; Harvey, J. W.; Zhou, B.; Wasilwa, L.; Feng, C.; Ouédraogo, I.; Wang, G.-L.; Mitchell, T. K.; Talbot, N. J.; Correll, J. C. Assessment of the virulence spectrum and its association with genetic diversity in *Magnaporthe oryzae* populations from sub-Saharan Africa. *Phytopathol.*, **2017**, *107(7)*, 852-863. https://doi.org/10.1094/PHYTO-08-16-0319-R
39. Wu, Q.; Wang, Y.; Liu, L. N.; Shi, K.; Li, C. Y. Comparative genomics and gene pool analysis reveal the decrease of genome diversity and gene number in rice blast fungi by stable adaption with rice. *J. Fungi*, **2021**, *8(1)*, 5. https://doi.org/10.3390/jof8010005
40. Dalman, K.; Himmelstrand, K.; Olson, Å.; Lind, M.; Brandström-Durling, M.; Stenlid, J. A genome-wide association study identifies genomic regions for virulence in the non-model organism *Heterobasidion annosum ss*. *PLoS One*, **2013**, *8(1)*, e53525. https://doi.org/10.1371/journal.pone.0053525
41. Lu, S.; Edwards, M. C.; Friesen, T. L. Genetic variation of single nucleotide polymorphisms identified at the mating type locus correlates with form-specific disease phenotype in the barley net blotch fungus *Pyrenophora teres*. *Eur. J. Plant Pathol.*, **2013**, *135(1)*, 49-65. https://doi.org/10.1007/s10658-012-0064-8
42. Ahmed Lhadj, W.; Boungab, K.; Righi Assia, F.; Çelik Oğuz, A.; Karakaya, A.; Ölmez, F. Genetic diversity of *Pyrenophora teres* in Algeria. *J. Plant Pathol.*, **2022**, *104(1)*, 305-315. https://doi.org/10.1007/s42161-021-01010-0
43. Singh, S. K.; Doshi, A.; Pancholy, A.; Pathak, R. Biodiversity in wood-decay macro-fungi associated with declining arid zone trees of India as revealed by nuclear rDNA analysis. *Eur. J. Plant Pathol.*, *2013*, *136(2)*, 373-382. https://doi.org/10.1007/s10658-013-0172-0
44. Sun, X.; Kang, S.; Zhang, Y.; Tan, X.; Yu, Y.; He, H.; Zhang, X.; Liu, Y.; Wang, S.; Sun, W.; Cai, L.; Li, S. Genetic diversity and population structure of rice pathogen *Ustilaginoidea virens* in China. *PLoS One*, **2013**, *8(9)*, e76879. https://doi.org/10.1371/journal.pone.0076879
45. Tran, V. T.; Braus-Stromeyer, S. A.; Timpner, C.; Braus, G. H. Molecular diagnosis to discriminate pathogen and apathogen species of the hybrid *Verticillium longisporum* on the oilseed crop *Brassica napus*. *App. Microbiol. Biotechnol.*, **2013**, *97(10)*, 4467-4483. https://doi.org/10.1007/s00253-012-4530-1
46. Hane, J. K.; Anderson, J. P.; Williams, A. H.; Sperschneider, J.; Singh, K. B. Genome sequencing and comparative genomics of the broad host-range pathogen *Rhizoctonia solani* AG8. *PLoS Genet.*, **2014**, *10(5)*, e1004281. https://doi.org/10.1371/journal.pgen.1004281
47. Lendenmann, M. H.; Croll, D.; Stewart, E. L.; McDonald, B. A. Quantitative trait locus mapping of melanization in the plant pathogenic fungus *Zymoseptoria tritici*. *G3*, **2014**, *4(12)*, 2519-2533. https://doi.org/10.1534/g3.114.015289
48. Lendenmann, M. H.; Croll, D.; Palma-Guerrero, J.; Stewart, E. L.; McDonald, B. A. QTL mapping of temperature sensitivity reveals candidate genes for thermal adaptation and growth morphology in the plant pathogenic fungus *Zymoseptoria tritici*. *Heredity*, **2016**, *116(4)*, 384-394. https://doi.org/10.1038/hdy.2015.111
49. Zhong, Z.; Marcel, T. C.; Hartmann, F. E.; Ma, X.; Plissonneau, C.; Zala, M.; Ducasse, A.; Confais, J.; Compain, J.; Lapalu, N.; Amselem, J.; McDonald, B. A.; Croll, D.; Palma-Guerrero, J. A small secreted protein in *Zymoseptoria tritici* is responsible for avirulence on wheat cultivars carrying the Stb6 resistance gene. *New Phytol.*, **2017**, *214(2)*, 619-631. https://doi.org/10.1111/nph.14434
50. Mallik, I.; Arabiat, S.; Pasche, J. S.; Bolton, M. D.; Patel, J. S.; Gudmestad, N. C. Molecular characterization and detection of mutations associated with resistance to succinate dehydrogenase-inhibiting fungicides in *Alternaria solani. Phytopathol.*, **2014**, *104(1)*, 40-49. https://doi.org/10.1094/PHYTO-02-13-0041-R
51. Milgroom, M. G.; Jimenez-Gasco, M. D. M.; Olivares García, C.; Drott, M. T.; Jimenez-Diaz, R. M. Recombination between clonal lineages of the asexual fungus *Verticillium dahliae* detected by genotyping by sequencing. *PloS One*, **2014**, *9(9)*, e106740. https://doi.org/10.1371/journal.pone.0106740
52. Faino, L.; Seidl, M. F.; Shi-Kunne, X.; Pauper, M.; van den Berg, G. C.; Wittenberg, A. H.; Thomma, B. P. Transposons passively and actively contribute to evolution of the two-speed genome of a fungal pathogen. *Genome Res.*, **2016**, *26(8)*, 1091-1100. https://doi.org/10.1101/gr.204974.116

53. Persoons, A.; Morin, E.; Delaruelle, C.; Payen, T.; Halkett, F.; Frey, P.; Frey, P.; De Mita, S.; Duplessis, S. Patterns of genomic variation in the poplar rust fungus *Melampsora larici-populina* identify pathogenesis-related factors. *Front. Plant Sci.*, **2014**, *5*, 450. https://doi.org/10.3389/fpls.2014.00450
54. Takaoka, S.; Kurata, M.; Harimoto, Y.; Hatta, R.; Yamamoto, M.; Akimitsu, K.; Tsuge, T. Complex regulation of secondary metabolism controlling pathogenicity in the phytopathogenic fungus *Alternaria alternata*. *New Phytol.*, **2014**, *202(4)*, 1297-1309. https://doi.org/10.1111/nph.12754
55. Leboldus, J. M.; Kinzer, K.; Richards, J.; Ya, Z.; Yan, C.; Friesen, T. L.; Brueggeman, R. Genotype-by-sequencing of the plant-pathogenic fungi *Pyrenophora* teres and *Sphaerulina musiva* utilizing Ion Torrent sequence technology. *Mol. Plant Pathol.*, **2015**, *16(6)*, 623-632. https://doi.org/10.1111/mpp.12214
56. Summers, C. F.; Gulliford, C. M.; Carlson, C. H.; Lillis, J. A.; Carlson, M. O.; Cadle-Davidson, L.; Gent, D. H.; Smart, C. D. Identification of genetic variation between obligate plant pathogens *Pseudoperonospora cubensis* and *P. humuli* using RNA sequencing and genotyping-by-sequencing. *PLoS One*, **2015**, *10(11)*, e0143665. https://doi.org/10.1371/journal.pone.0143665
57. Anderson, C., Khan, M. A.; Catanzariti, A. M.; Jack, C. A.; Nemri, A.; Lawrence, G. J.; ; Jones, D. A. Genome analysis and avirulence gene cloning using a high-density RADseq linkage map of the flax rust fungus, *Melampsora lini*. *BMC Genom.*, **2016**, *17(1)*, 1-20. https://doi.org/10.1186/s12864-016-3011-9
58. Gao, Y.; Liu, Z.; Faris, J. D.; Richards, J.; Brueggeman, R. S.; Li, X.; Oliver, R. P.;. McDonald, B. A; Friesen, T. L. Validation of genome-wide association studies as a tool to identify virulence factors in *Parastagonospora nodorum*. *Phytopathol.*, **2016**, *106(10)*, 1177-1185. https://doi.org/10.1094/PHYTO-02-16-0113-FI
59. Li, H.; Zhou, G. Y.; Liu, J. A.; Xu, J. Population genetic analyses of the fungal pathogen *Colletotrichum fructicola* on tea-oil trees in China. *PloS One*, **2016**, *11(6)*, e0156841. https://doi.org/10.1371/journal.pone.0156841
60. Malapi-Wight, M.; Salgado-Salazar, C.; Demers, J. E.; Clement, D. L.; Rane, K. K.; Crouch, J. A. Sarcococca blight: Use of whole-genome sequencing for fungal plant disease diagnosis. *Plant Dis.*, **2016**, *100(6)*, 1093-1100. https://doi.org/10.1094/PDIS-10-15-1159-RE
61. Mohd-Assaad, N.; McDonald, B. A.; Croll, D. Multilocus resistance evolution to azole fungicides in fungal plant pathogen populations. *Mol. Ecol.*, **2016**, *25(24)*, 6124-6142. https://doi.org/10.1111/mec.13916
62. Walkowiak, S.; Rowland, O.; Rodrigue, N.; Subramaniam, R. Whole genome sequencing and comparative genomics of closely related *Fusarium* Head Blight fungi: *Fusarium graminearum*, *F. meridionale* and *F. asiaticum*. *BMC Genom.*, **2016**, *17(1)*, 1-15. https://doi.org/10.1186/s12864-016-3371-1
63. Xia, C.; Wan, A.; Wang, M.; Jiwan, D. A.; See, D. R.; Chen, X. Secreted protein gene derived-single nucleotide polymorphisms (SP-SNPs) reveal population diversity and differentiation of *Puccinia striiformis f. sp. tritici* in the United States. *Fungal Biol.*, **2016**, *120(5)*, 729-744. https://doi.org/10.1016/j.funbio.2016.02.007
64. Xia, C.; Wang, M.; Wan, A.; Jiwan, D. A.; See, D. R.; Chen, X. Association analysis of SP-SNPs and avirulence genes in *Puccinia striiformis f. sp. tritici*, the wheat stripe rust pathogen. *Am. J. Plant Sci.*, **2016**, *7(01)*, 126. https://doi.org/10.4236/ajps.2016.71014
65. Zhu, X. Q.; Niu, C. W.; Chen, X. Y.; Guo, L. Y. *Monilinia* species associated with brown rot of cultivated apple and pear fruit in China. *Plant Dis.*, *2016*, *100(11)*, 2240-2250.
66. Ayukawa, Y.; Hanyuda, S.; Fujita, N.; Komatsu, K.; Arie, T. Novel loop-mediated isothermal amplification (LAMP) assay with a universal QProbe can detect SNPs determining races in plant pathogenic fungi. *Sci. Rep.*, **2017**, *7(1)*, 1-9. https://doi.org/10.1038/s41598-017-04084-y
67. Chebil, S.; Fersi, R.; Bouzid, M.; Quaglino, F.; Chenenaoui, S.; Melki, I.; Durante, G.; Zacchi, E.; Bahri, B. A.; Bianco, P. A.; Rhouma, A. Fungi from the Diaporthaceae and Botryosphaeriaceae families associated with grapevine decline in Tunisia. *Cienc Investig Agrar.*, **2017**, *44(2)*, 127-138. https://doi.org/10.1094/PDIS-03-16-0325-RE
68. Chung, C. L.; Lee, T. J.; Akiba, M.; Lee, H. H.; Kuo, T. H.; Liu, D.; Ke, H.-M.; Yokoi, T.; Roa, M. B.; Lu, M.-Y. J.; Chang, Y.-Y.; Ann, P.-J.; Tsai, J.-N.; Chen, C.-Y.; Tzean, S.-S.; Ota, Y.; Hattori, T.; Sahashi, N.; Liou, R.-F.; Kikuchi, T.; Tsai, I. J. Comparative and population genomic landscape of *Phellinus noxius*: A hypervariable fungus causing root rot in trees. *Mol. Ecol.*, **2017**, *26(22)*, 6301-6316. https://doi.org/10.1111/mec.14359
69. Franco-Orozco, B.; Berepiki, A.; Ruiz, O.; Gamble, L.; Griffe, L. L.; Wang, S.; Birch, P. R. J.; Kanyuka, K.; Avrova, A. A new proteinaceous pathogen-associated molecular pattern (PAMP) identified in Ascomycete fungi induces cell death in Solanaceae. *New Phytol.*, **2017**, *214(4)*, 1657-1672. https://doi.org/10.1111/nph.14542
70. Laurent, B.; Moinard, M.; Spataro, C.; Ponts, N.; Barreau, C.; Foulongne-Oriol, M. Landscape of genomic diversity and host adaptation in *Fusarium graminearum*. *BMC Genom.*, **2017**, *18(1)*, 1-19. https://doi.org/10.1186/s12864-017-3524-x
71. Praz, C. R.; Bourras, S.; Zeng, F.; Sánchez-Martín, J.; Menardo, F.; Xue, M.; Yang, L.; Roffler, S.; Böni, R.; Herren, G.; McNally, K. E.; Ben-David, R.; Parlange, F.; Oberhaensli, S.; Flückiger, S.; Schäfer, L. K. ; Wicker, T.; Yu, D.; Keller, B. AvrPm2 encodes an RNase-like avirulence effector which is conserved in the two different specialized forms of wheat and rye powdery mildew fungus. *New Phytol.*, **2017**, *213(3)*, 1301-1314. https://doi.org/10.1111/nph.14372
72. Saleh, A. A.; Sharafaddin, A. H.; El_Komy, M. H.; Ibrahim, Y. E.; Hamad, Y. K.; Molan, Y. Y. *Fusarium* species associated with date palm in Saudi Arabia. *Eur. J. Plant Pathol.*, **2017**, *148(2)*, 367-377. https://doi.org/10.1007/s10658-016-1095-3
73. Hartmann, F. E.; Rodríguez de la Veja; R. C., Brandenburg; J. T., Carpentier, F.; Giraud, T. Gene presence–absence polymorphism in castrating anther-smut fungi: recent gene gains and phylogeographic structure. *Genome Biol. Evol.*, **2018**, *10(5)*, 1298-1314. https://doi.org/10.1093/gbe/evy089
74. Hartmann, F. E.; Snirc, A.; Cornille, A.; Godé, C.; Touzet, P.; Van Rossum, F.; Fournier, E.; Le Prieur, S.; Shykoff, J.; Giraud, T. Congruent population genetic structures and divergence histories in anther-smut fungi and their host plants *Silene italica* and the *Silene nutans* species complex. *Mol. Ecol.*, **2020**, *29(6)*, 1154-1172. https://doi.org/10.1111/mec.15387

75. McDonald, M. C.; Ahren, D.; Simpfendorfer, S.; Milgate, A.; Solomon, P. S. The discovery of the virulence gene ToxA in the wheat and barley pathogen *Bipolaris sorokiniana*. *Mol. Plant Pathol.*, **2018**, *19(2)*, 432-439. https://doi.org/10.1111/mpp.12535
76. Orton, E. S.; Brasier, C. M.; Bilham, L. J.; Bansal, A.; Webber, J. F.; Brown, J. K. Population structure of the ash dieback pathogen, *Hymenoscyphus fraxineus*, in relation to its mode of arrival in the UK. *Plant Pathol.*, **2018**, *67(2)*, 255-264. https://doi.org/10.1111/ppa.12762
77. Silva, D. N.; Várzea, V.; Paulo, O. S.; Batista, D. Population genomic footprints of host adaptation, introgression and recombination in coffee leaf rust. *Mol. Plant Pathol.*, **2018**, *19(7)*, 1742-1753. https://doi.org/10.1111/mpp.12657
78. Vieira, A.; Silva, D. N.; Várzea, V.; Paulo, O. S.; Batista, D. Novel insights on colonization routes and evolutionary potential of *Colletotrichum kahawae*, a severe pathogen of *Coffea arabica*. *Mol. Plant Pathol.*, **2018**, *19(11)*, 2488-2501. https://doi.org/10.1111/mpp.12726
79. Aoun, M.; Kolmer, J. A.; Breiland, M.; Richards, J.; Brueggeman, R. S.; Szabo, L. J.; Acevedo, M.. Genotyping-by-sequencing for the study of genetic diversity in *Puccinia triticina*. *Plant Dis.*, **2020**, *104(3)*, 752-760. https://doi.org/10.1094/PDIS-09-19-1890-RE
80. Dussert, Y.; Legrand, L.; Mazet, I. D.; Couture, C.; Piron, M. C.; Serre, R. F.; Bouchez, O.; Mestre, P.; Toffolatti, S. L.; Giraud, T.; Delmotte, F. Identification of the first oomycete mating-type locus sequence in the grapevine downy mildew pathogen, *Plasmopara viticola*. *Curr. Biol.*, **2020**, *30(20)*, 3897-3907. https://doi.org/10.1016/j.cub.2020.07.057
81. Maymon, M.; Sela, N.; Shpatz, U.; Galpaz, N.; Freeman, S. The origin and current situation of *Fusarium oxysporum f. sp. cubense* tropical race 4 in Israel and the Middle East. *Sci. Rep.*, **2020**, *10(1)*, 1-11. https://doi.org/10.1038/s41598-020-58378-9
82. Gurjar, M. S.; Aggarwal, R.; Jain, S.; Sharma, S.; Singh, J.; Gupta, S.; Agarwal, S.; Saharan, M. S. Multilocus sequence typing and single nucleotide polymorphism analysis in *Tilletia indica* isolates inciting karnal bunt of wheat. *J. Fungi*, **2021**, *7(2)*, 103. https://doi.org/10.3390/jof7020103
83. Cui, L.; Deng, J.; Zhao, L.; Hu, Y.; Liu, T. Genetic diversity and population genetic structure of *Setosphaeria turcica* from sorghum in three Provinces of China using single nucleotide polymorphism markers. *Front. Microbiol.*, **2022**, *13*. https://doi.org/10.3389/fmicb.2022.853202
84. Palma-Guerrero, J.; Hall, C. R.; Kowbel, D.; Welch, J.; Taylor, J. W.; Brem, R. B.; Glass, N. L. Genome wide association identifies novel loci involved in fungal communication. *PLoS Genet.*, **2013**, *9(8)*, e1003669. https://doi.org/10.1371/journal.pgen.1003669
85. Malbreil, M.; Tisserant, E.; Martin, F.; Roux, C. Genomics of arbuscular mycorrhizal fungi: out of the shadows. In Advances in Botanical Research, Academic Press, Vol. 70, 2014. pp. 259-290.
86. Ropars, J.; Corradi, N. Homokaryotic vs heterokaryotic mycelium in arbuscular mycorrhizal fungi: different techniques, different results?. *New Phytol.*, **2015**, *208(3)*, 638-641.
87. Ropars, J.; Toro, K. S.; Noel, J.; Pelin, A.; Charron, P.; Farinelli, L.; Marton, T.; Krüger, M.; Fuchs, J.; Brachmann, A.; Corradi, N. Evidence for the sexual origin of heterokaryosis in arbuscular mycorrhizal fungi. *Nat. Microbiol.*, **2016**, *1(6)*, 1-9. https://doi.org/10.1038/nmicrobiol.2016.33
88. Young, J. P. W. Genome diversity in arbuscular mycorrhizal fungi. *Curr. Opin. Plant Biol.*, **2015**, *26*, 113-119. https://doi.org/10.1016/j.pbi.2015.06.005
89. Chen, E. C.; Mathieu, S.; Hoffrichter, A.; Sedzielewska-Toro, K.; Peart, M.; Pelin, A.; Ndikumana, S.; Ropars, J.; Dreissig, S.; Fuchs, J.; Brachmann, A.; Corradi, N. Single nucleus sequencing reveals evidence of inter-nucleus recombination in arbuscular mycorrhizal fungi. *Elife*, **2018**, *7*, e39813. https://doi.org/10.7554/eLife.39813.001
90. Reinhardt, D., Roux, C., Corradi, N.; Di Pietro, A. Lineage-specific genes and cryptic sex: parallels and differences between arbuscular mycorrhizal fungi and fungal pathogens. *Trends Plant Sci.*, **2021**, *26(2)*, 111-123. https://doi.org/10.1016/j.tplants.2020.09.006
91. Suga, H.; Kitajima, M.; Nagumo, R.; Tsukiboshi, T.; Uegaki, R.; Nakajima, T.; Kushiro, M.; Nakagawa, H.; Shimizu, M.; Kageyama, K.; Hyakumachi, M. A single nucleotide polymorphism in the translation elongation factor 1α gene correlates with the ability to produce fumonisin in Japanese *Fusarium fujikuroi*. *Fungal Biol.*, **2014**, *118(4)*, 402-412. https://doi.org/10.1016/j.funbio.2014.02.005
92. Badet, T.; Peyraud, R.; Mbengue, M.; Navaud, O.; Derbyshire, M.; Oliver, R. P.; Barbacci, A.; Raffaele, S. Codon optimization underpins generalist parasitism in fungi. *Elife*, **2017**, *6*, e22472. https://doi.org/10.7554/eLife.22472
93. Tsykun, T.; Rellstab, C.; Dutech, C.; Sipos, G.; Prospero, S. Comparative assessment of SSR and SNP markers for inferring the population genetic structure of the common fungus *Armillaria cepistipes*. *Heredity*, **2017**, *119(5)*, 371-380. https://doi.org/10.1038/hdy.2017.48
94. Castiblanco, V., Castillo, H. E.; Miedaner, T. Candidate genes for aggressiveness in a natural *Fusarium culmorum* population greatly differ between wheat and rye head blight. *J. Fungi*, **2018**, *4(1)*, 14. https://doi.org/10.3390/jof4010014
95. Kubosaki, A.; Kobayashi, N.; Watanabe, M.; Yoshinari, T.; Takatori, K.; Kikuchi, Y.; Hara-Kudo, Y.; Terajima, J.; Sugita-Konishi, Y. A new protocol for the detection of sterigmatocystin-producing *Aspergillus* section versicolores using a high discrimination polymerase. *Biocontrol Science*, **2020**, *25(2)*, 113-118. https://doi.org/10.4265/bio.25.113
96. Au, C. H.; Cheung, M. K.; Wong, M. C.; Chu, A. K. K.; Law, P. T. W.; Kwan, H. S. Rapid genotyping by low-coverage resequencing to construct genetic linkage maps of fungi: a case study in *Lentinula edodes*. *BMC Res. Notes*, **2013**, *6(1)*, 1-8. https://doi.org/10.1186/1756-0500-6-307
97. Grubisha, L. C.; Cotty, P. J. Genetic analysis of the *Aspergillus flavus* vegetative compatibility group to which a biological control agent that limits aflatoxin contamination in US crops belongs. *App. Environm. Microbiol.*, **2015**, *81(17)*, 5889-5899. https://doi.org/10.1128/AEM.00738-15

98. Payen, T.; Murat, C.; Gigant, A.; Morin, E.; De Mita, S.; Martin, F. A survey of genome-wide single nucleotide polymorphisms through genome resequencing in the Périgord black truffle (*Tuber melanosporum* Vittad.). *Mol. Ecol. Resour.*, **2015**, *15(5)*, 1243-1255. https://doi.org/10.1111/1755-0998.12391
99. Julca, I.; Droby, S.; Sela, N.; Marcet-Houben, M.; Gabaldón, T. Contrasting genomic diversity in two closely related postharvest pathogens: *Penicillium digitatum* and *Penicillium expansum*. *Genome Biol. Evol.*, **2016**, *8(1)*, 218-227. https://doi.org/10.1093/gbe/evv252
100. An, H.; Lee, H. Y.; Shim, D.; Choi, S. H.; Cho, H.; Hyun, T. K.; Jo, I.-H. ; Chung, J. W. Development of CAPS markers for evaluation of genetic diversity and population structure in the germplasm of button mushroom (*Agaricus bisporus*). *J. Fungi*, **2021**, *7(5)*, 375. https://doi.org/10.3390/jof7050375
101. 101. Leavitt, S. D.; Lumbsch, H. T.; Stenroos, S.; Clair, L. L. S. Pleistocene speciation in North American lichenized fungi and the impact of alternative species circumscriptions and rates of molecular evolution on divergence estimates. *PLoS One*, **2013**, *8(12)*, e85240. https://doi.org/10.1371/journal.pone.0085240
102. Altermann, S.; Leavitt, S. D.; Goward, T.; Nelsen, M. P.; Lumbsch, H. T. How do you solve a problem like *Letharia*? A new look at cryptic species in lichen-forming fungi using Bayesian clustering and SNPs from multilocus sequence data. *PLoS One*, **2014**, *9(5)*, e97556. https://doi.org/10.1371/journal.pone.0097556
103. Allen, J. L.; McKenzie, S. K.; Sleith, R. S.; Alter, S. E. First genome-wide analysis of the endangered, endemic lichen *Cetradonia linearis* reveals isolation by distance and strong population structure. *Am. J. Bot.*, **2018**, *105(9)*, 1556-1567. https://doi.org/10.1002/ajb2.1150
104. Tuovinen, V.; Ekman, S.; Thor, G.; Vanderpool, D.; Spribille, T.; Johannesson, H. Two basidiomycete fungi in the cortex of wolf lichens. *Curr. Biol.*, **2019**, *29(3)*, 476-483. https://doi.org/10.1016/j.cub.2018.12.022
105. Keuler, R.; Garretson, A.; Saunders, T.; Erickson, R. J.; St Andre, N.; Grewe, F.; Smith, H.; Lumbsch, H. T.; Huang, J.-P.; St. Clair, L. L.; Leavitt, S. D. Genome-scale data reveal the role of hybridization in lichen-forming fungi. *Sci. Rep.*, **2020**, *10(1)*, 1-14. https://doi.org/10.1038/s41598-020-58279-x
106. Li, Y.; Jiao, L.; Yao, Y. J. Non-concerted ITS evolution in fungi, as revealed from the important medicinal fungus *Ophiocordyceps sinensis*. *Mol. Phylogenetics Evol.*, **2013**, *68(2)*, 373-379. https://doi.org/10.1016/j.ympev.2013.04.010
107. Li, Y. L.; Yao, Y. S.; Xie, W. D.; Zhu, J. S. The molecular heterogeneity of natural *Cordyceps sinensis* with multiple *Ophiocordyceps sinensis* fungi challenges the anamorph-teleomorph connection hypotheses. *Am. J. Biomed. Sci.*, **2016**, *8(2)*. https://doi.org/10.5099/aj160200123
108. Wang, M.; Jiang, X.; Wu, W.; Hao, Y.; Su, Y.; Cai, L.; Xiang, M.; Liu, X. Psychrophilic fungi from the world's roof. *Pers. Mol. Phylogeny Evol. Fungi*, **2015**, *34(1)*, 100-112. https://doi.org/10.3767/003158515X685878
109. Knox, B. P.; Blachowicz, A.; Palmer, J. M.; Romsdahl, J.; Huttenlocher, A.; Wang, C. C.; Keller, N. P.; Venkateswaran, K. Characterization of *Aspergillus fumigatus* isolates from air and surfaces of the international space station. *Msphere*, **2016**, *1(5)*, e00227-16. https://doi.org/10.1128/mSphere.00227-16
110. Sun, L.; Fu, Y.; Yang, Y.; Wang, X.; Cui, W.; Li, D.; Yuan, X.; Zhang, Z.; Fu, Y.; Li, Y. Genomic analyses reveal evidence of independent evolution, demographic history, and extreme environment adaptation of Tibetan Plateau *Agaricus bisporus*. *Front. Microbiol.*, **2019**, *1786*. https://doi.org/10.3389/fmicb.2019.01786
111. Formey, D.; Molès, M.; Haouy, A.; Savelli, B.; Bouchez, O.; Bécard, G.; Roux, C. Comparative analysis of mitochondrial genomes of *Rhizophagus irregularis* - syn. *Glomus irregulare* - reveals a polymorphism induced by variability generating elements. *New Phytol.*, **2012**, *196(4)*, 1217-1227. https://doi.org/10.1111/j.1469-8137.2012.04283.x
112. Tisserant, E.; Malbreil, M.; Kuo, A.; Kohler, A.; Symeonidi, A.; Balestrini, R.; Charron, P.; Duensing, N.; dit Frey, N. F.; Gianinazzi-Pearson, V.; Gilbert, L. B.; Handa, Y.; Herr, J. R.; Hijri, M.; Koul, R.; Kawaguchi, M.; Krajinski, F.; Lammers, P. J.; Masclaux, F. G.; Murat, C.; Morin, E.; Ndikumana, S.; Pagni, M.; Petitpierre, D.; Requena, N.; Rosikiewicz, P.; Riley, R.; Saito, K.; San Clemente, H.; Shapiro, H.; van Tuinen, D.; Bécard, G.; Bonfante, P.; Paszkowski, U.; Shachar-Hill, Y. Y.; Tuskan, G. A.; Young, J. P. W.; Sanders, I. R.; Henrissat, B.; Rensing, S. A.; Grigoriev, I. V.; Corradi, N.; Roux, C.;Martin, F. Genome of an arbuscular mycorrhizal fungus provides insight into the oldest plant symbiosis. *PNAS*, **2013**, *110(50)*, 20117-20122. https://doi.org/10.1073/pnas.1313452110
113. Wyss, T.; Masclaux, F. G.; Rosikiewicz, P.; Pagni, M.; Sanders, I. R. Population genomics reveals that within-fungus polymorphism is common and maintained in populations of the mycorrhizal fungus *Rhizophagus irregularis*. *ISME J.*, **2016**, *10(10)*, 2514-2526. https://doi.org/10.1038/ismej.2016.29
114. Grubisha, L. C.; Levsen, N.; Olson, M. S.; Lee Taylor, D. Intercontinental divergence in the *Populus*-associated ectomycorrhizal fungus, *Tricholoma populinum*. *New Phytol.*, **2012**, *194(2)*, 548-560. https://doi.org/10.1111/j.1469-8137.2012.04066.x
115. Branco, S.; Gladieux, P.; Ellison, C. E.; Kuo, A.; LaButti, K.; Lipzen, A.; Grigoriev, I. V. ; Liao, H.-L.; Vilgalys, R.; Peay, K. G.; Taylor, J. W.; Bruns, T. D. Genetic isolation between two recently diverged populations of a symbiotic fungus. *Mol. Ecol.*, **2015**, *24(11)*, 2747-2758. https://doi.org/10.1111/mec.13132
116. Thiéry, O.; Vasar, M.; Jairus, T.; Davison, J.; Roux, C.; Kivistik, P. A.; Metspalu, A.; Milani, L.; Saks, Ü.; Moora, M.; Zobel, M.; Öpik, M. Sequence variation in nuclear ribosomal small subunit, internal transcribed spacer and large subunit regions of *Rhizophagus irregularis* and *Gigaspora margarita* is high and isolate-dependent. *Mol. Ecol.*, **2016**, *25(12)*, 2816-2832. https://doi.org/10.1111/mec.13655
117. Corradi, N.; Brachmann, A. Fungal mating in the most widespread plant symbionts?. *Trends Plant Sci.*, **2017**, *22(2)*, 175-183
118. Bergelson, J.; Mittelstrass, J.; Horton, M. W. Characterizing both bacteria and fungi improves understanding of the *Arabidopsis* root microbiome. *Sci. Rep.*, **2019**, *9(1)*, 1-11. https://doi.org/10.1038/s41598-018-37208-z

119. Bazzicalupo, A. L.; Ruytinx, J.; Ke, Y. H.; Coninx, L.; Colpaert, J. V.; Nguyen, N. H.; Vilgalys, R.; Branco, S. Fungal heavy metal adaptation through single nucleotide polymorphisms and copy-number variation. *Mol. Ecol.*, **2020**, *29(21)*, 4157-4169. https://doi.org/10.1111/mec.15618
120. Porciuncula, J. D. O.; Furukawa, T.; Mori, K.; Shida, Y.; Hirakawa, H.; Tashiro, K.; Kuhara, S.; Nakagawa, S.; Morikawa, Y.; Ogasawara, W. Single nucleotide polymorphism analysis of a *Trichoderma reesei* hyper-cellulolytic mutant developed in Japan. *Biosci. Biotechnol. Biochem.*, N, *77(3)*, 534-543. https://doi.org/10.1271/bbb.120794

References: Supplementary Table S7

1. Griffiths, E. J; Hu, G.; Fries, B.; Caza, M.; Wang, J.; Gsponer, J.; ; Kronstad, J. W. A defect in ATP-citrate lyase links acetyl-CoA production, virulence factor elaboration and virulence in *Cryptococcus neoformans*. *Mol. Microbiol.*, **2012**, *86(6)*, 1404-1423. https://doi.org/10.1111/mmi.12065
2. Janbon, G.; Ormerod, K. L.; Paulet, D.; Byrnes III, E. J.; Yadav, V.; Chatterjee, G.; Mullapudi, N.; Hon, C.-C.; Billmyre, R. B.; Brunel, F.; Bahn, Y.-S.; Chen, W.; Chen, Y.; Chow, E. W. L.; Coppée, J.-Y.; Floyd-Averette, A.; Gaillardin, C.; Gerik, K. J.; Goldberg, J.; Gonzalez-Hilarion, S.; Gujja, S.; Hamlin, J. L.; Hsueh, Y.-P.; Ianiri, G.; Jones, S.; Kodira, C. D.; Kozubowski, L.; Lam, W.; Marra, M.; Mesner, L. D.; Mieczkowski, P. A.; Moyrand, F.; Nielsen, K.; Proux, C.; Rossignol, T.; Schein, J. E.; Sun, S.; Wollschlaeger, C.; Wood, I. A.; Zeng, Q.; Neuvéglise, C.; Newlon, C. S.; Perfect, J. R.; Lodge, J. K.; Idnurm, A.; Stajich, J. E.; Kronstad, J. W.; Sanyal, K.; Heitman, J.; Fraser, J. A.; Cuomo, C. A.; Dietrich, F. S. Analysis of the genome and transcriptome of *Cryptococcus neoformans* var. *grubii* reveals complex RNA expression and microevolution leading to virulence attenuation. *PLoS Genet.*, **2014**, *10(4)*, e1004261. https://doi.org/10.1371/journal.pgen.1004261
3. Beale, M. A.; Sabiiti, W.; Robertson, E. J.; Fuentes-Cabrejo, K. M.; O'Hanlon, S. J.; Jarvis, J. N.; Loyse, A.; Meintjes, G.; Harrison, T. S.; May, R. C.; Fisher, M. C.; Bicanic, T. Genotypic diversity is associated with clinical outcome and phenotype in cryptococcal meningitis across Southern Africa. *PLoS Negl. Trop. Dis.*, **2015**, *9(6)*, e0003847. https://doi.org/10.1371/journal.pntd.0003847
4. Klaassen, C. H.; Gibbons, J. G.; Fedorova, N. D.; Meis, J. F.; Rokas, A. Evidence for genetic differentiation and variable recombination rates among Dutch populations of the opportunistic human pathogen *Aspergillus fumigatus*. *Mol. Ecol.*, **2012**, *21(1)*, 57-70. https://doi.org/10.1111/j.1365-294X.2011.05364.x
5. Ashu, E. E.; Hagen, F.; Chowdhary, A.; Meis, J. F.; Xu, J. Global population genetic analysis of *Aspergillus fumigatus*. *MSphere*, **2017**, *2(1)*, e00019-17. https://doi.org/10.1128/mSphere.00019-17
6. Jones, J. T.; Liu, K. W.; Wang, X.; Kowalski, C. H.; Ross, B. S.; Mills, K. A.; Kerkaert, J. D.; Hohl, T. M.; Lofgren, L. A. ; Stajich, J. E.; Obar, J. J.; Cramer, R. A. *Aspergillus fumigatus* strain-specific conidia lung persistence causes an allergic broncho-pulmonary aspergillosis-like disease phenotype. *Msphere*, **2021**, *6(1)*, e01250-20. https://doi.org/10.1128/mSphere.01250-20
7. Li, W.; Cama, V.; Feng, Y.; Gilman, R. H.; Bern, C.; Zhang, X.; Xiao, L. Population genetic analysis of *Enterocytozoon bieneusi* in humans. *Int. J. Parasitol.*, **2012**, *42(3)*, 287-293. https://doi.org/10.1016/j.ijpara.2012.01.003
8. Li, W.; Feng, Y.; Zhang, L.; Xiao, L. Potential impacts of host specificity on zoonotic or interspecies transmission of *Enterocytozoon bieneusi*. *Infect. Genet. Evol.*, **2019**, *75*, 104033. https://doi.org/10.1016/j.meegid.2019.104033
9. Bartelli, T. F.; Ferreira, R. C.; Colombo, A. L.; Briones, M. R. Intraspecific comparative genomics of *Candida albicans* mitochondria reveals non-coding regions under neutral evolution. *Infect. Genet. Evol.*, **2013**, *14*, 302-312. https://doi.org/10.1016/j.meegid.2012.12.012
10. Alves, F. L.; Ribeiro, M. A.; Hahn, R. C.; de Melo Teixeira, M.; de Camargo, Z. P.; Cisalpino, P. S.; Marini, M. M. Transposable elements and two other molecular markers as typing tools for the genus *Paracoccidioides*. *Sabouraudia*, **2014**, *53(2)*, 165-170. https://doi.org/10.1093/mmy/myu074
11. Mirhendi, H.; Makimura, K.; de Hoog, G. S.; Rezaei-Matehkolaei, A.; Najafzadeh, M. J.; Umeda, Y.; Ahmadi, B. Translation elongation factor 1-α gene as a potential taxonomic and identification marker in dermatophytes. *Med. Mycol.*, **2015**, *53(3)*, 215-224. https://doi.org/10.1093/mmy/myu088
12. Farrer, R. A.; Voelz, K.; Henk, D. A.; Johnston, S. A.; Fisher, M. C.; May, R. C.; Cuomo, C. A. Microevolutionary traits and comparative population genomics of the emerging pathogenic fungus *Cryptococcus gattii*. *Philos. Trans. R. Soc. Lond., B, Biol. Sci.*, **2016**, *371(1709)*, 20160021. https://doi.org/10.1098/rstb.2016.0021
13. Honnavar, P.; Prasad, G. S.; Ghosh, A.; Dogra, S.; Handa, S.; Rudramurthy, S. M. *Malassezia arunalokei* sp. nov., a novel yeast species isolated from seborrheic dermatitis patients and healthy individuals from India. *J. Clin. Microbiol.*, **2016**, *54(7)*, 1826-1834. https://doi.org/10.1128/JCM.00683-16
14. Billmyre, R. B.; Clancey, S. A.; Heitman, J. Natural mismatch repair mutations mediate phenotypic diversity and drug resistance in *Cryptococcus deuterogattii*. *Elife*, **2017**, *6*, e28802. https://doi.org/10.7554/eLife.28802
15. Kiss, N.; Homa, M.; Manikandan, P.; Mythili, A.; Krizsán, K.; Revathi, R.; Varga, M.; Papp, T.; Vágvölgyi, C.; Kredics, L.; Kocsubé, S. New species of the genus *Curvularia*: *C. tamilnaduensis* and *C. coimbatorensis* from fungal keratitis cases in South India. *Pathogens*, **2019**, *9(1)*, 9. https://doi.org/10.3390/pathogens9010009
16. Carolus, H.; Jacobs, S.; Lobo Romero, C.; Deparis, Q.; Cuomo, C. A.; Meis, J. F.; Van Dijck, P. Diagnostic allele-specific PCR for the identification of *Candida auris* clades. *J. Fungi*, **2021**, *7(9)*, 754. https://doi.org/10.3390/jof7090754
17. Čmoková, A.; Rezaei-Matehkolaei, A.; Kuklová, I.; Kolařík, M.; Shamsizadeh, F.; Ansari, S.; Gharaghani, M.; Miňovská, V.; Najafzadeh, M. J.; Nouripour-Sisakht, S.; Yaguchi, T.; Zomorodian, K.; Zarrinfar, H.; Hubka, V. Discovery of new *Trichophyton* members, *T. persicum* and *T. spiraliforme* spp. nov., as a cause of highly inflammatory tinea cases in Iran and Czechia. *Microbiol. Spectr.*, **2021**, *9(2)*, e00284-21. https://doi.org/10.1128/Spectrum.00284-21

18. Liang, G.; Zhang, M.; Xu, W.; Wang, X.; Zheng, H.; Mei, H.; Liu, W. Characterization of mitogenomes from four Mucorales species and insights into pathogenicity. *Mycoses*, **2022**, *65(1)*, 45-56. https://doi.org/10.1111/myc.13374
19. Leopardi, S.; Blake, D.; Puechmaille, S. J. White-nose syndrome fungus introduced from Europe to North America. *Curr. Biol.*, **2015**, *25(6)*, R217-R219. https://doi.org/10.1016/j.cub.2015.01.047
20. Trivedi, J.; Lachapelle, J.; Vanderwolf, K. J.; Misra, V.; Willis, C. K.; Ratcliffe, J. M.; Ness, R. W.; Anderson, J. B.; Kohn, L. M. Fungus causing white-nose syndrome in bats accumulates genetic variability in North America with no sign of recombination. *Msphere*, **2017**, *2(4)*, e00271-17. https://doi.org/10.1128/mSphereDirect.00271-17
21. Dąbrowska, J.; Karamon, J.; Kochanowski, M.; Sroka, J.; Zdybel, J.; Cencek, T. *Tritrichomonas foetus* as a causative agent of tritrichomonosis in different animal hosts. J. Vet. Res., 63(4), 2019533. https://doi.org/10.2478/jvetres-2019-0072
22. Zhang, S.; Zhang, Y. J.; Li, Z. L. Complete mitogenome of the entomopathogenic fungus *Sporothrix insectorum* RCEF 264 and comparative mitogenomics in Ophiostomatales. *App. Microbiol. Biotechnol.*, **2019**, *103(14)*, 5797-5809. https://doi.org/10.1007/s00253-019-09855-3
23. Zhang, Z.; Lu, Y.; Xu, W.; Sui, L.; Du, Q.; Wang, Y.; Zhao, Y.; Li, Q. Influence of genetic diversity of seventeen *Beauveria bassiana* isolates from different hosts on virulence by comparative genomics. *BMC Genom.*, **2020**, *21(1)*, 1-12. https://doi.org/10.1186/s12864-020-06791-9
24. Čmoková, A.; Kolařík, M.; Dobiáš, R.; Hoyer, L. L.; Janouškovcová, H.; Kano, R.; Kuklová, I.; Lysková, P.; Machová, L.; Maier, T.; Mallátová, N.; Man, M.; Mencl, K.; Nenoff, P.; Peano, A.; Prausová, H.; Stubbe, D.; Uhrlaß, S.; Větrovský, T.; Wiegand C.; Hubka, V. Resolving the taxonomy of emerging zoonotic pathogens in the *Trichophyton benhamiae* complex. *Fungal Divers.*, **2020**, *104(1)*, 333-387. https://doi.org/10.1007/s13225-020-00465-3
25. Rezaei-Matehkolaei, A.; Makimura, K.; De Hoog, G. S.; Shidfar, M. R.; Satoh, K.; Najafzadeh, M. J.; Mirhendi, H. Discrimination of *Trichophyton tonsurans* and *Trichophyton equinum* by PCR-RFLP and by β-tubulin and Translation Elongation Factor 1-α sequencing. *Med. Mycol.*, **2012**, *50(7)*, 760-764. https://doi.org/10.3109/13693786.2012.661885
26. Wan, Q.; Xiao, L.; Zhang, X.; Li, Y.; Lu, Y.; Song, M.; Li, W. Clonal evolution of *Enterocytozoon bieneusi* populations in swine and genetic differentiation in subpopulations between isolates from swine and humans. *PLoS Negl. Trop. Dis.*, 2016, *10(8)*, e0004966. https://doi.org/10.1371/journal.pntd.0004966
27. Li, W.; Xiao, L. Multilocus sequence typing and population genetic analysis of *Enterocytozoon bieneusi*: host specificity and its impacts on public health. *Front. Genet.*, **2019**, *10*, 307. https://doi.org/10.3389/fgene.2019.00307
28. Leroch, M.; Plesken, C.; Weber, R. W.; Kauff, F.; Scalliet, G.; Hahn, M. Gray mold populations in German strawberry fields are resistant to multiple fungicides and dominated by a novel clade closely related to *Botrytis cinerea*. *Appl. Environ. Microbiol.*, **2013**, *79(1)*, 159-167. https://doi.org/10.1128/AEM.02655-12
29. Van Kan, J. A.; Stassen, J. H.; Mosbach, A.; Van Der Lee, T. A.; Faino, L.; Farmer, A. D.; Faino, L.; Farmer, A. D.; Papasotiriou, D. G.; Zhou, S.; Seidl, M. F.; Cottam, E.; Edel, D.; Hahn,. Schwartz, M.; D. C; Dietrich, R. A.; S. Widdison, S.; Scalliet, G. A gapless genome sequence of the fungus *Botrytis cinerea*. *Mol. Plant Pathol.*, **2017**, *18(1)*, 75-89. https://doi.org/10.1111/mpp.12384
30. Plesken, C.; Pattar, P.; Reiss, B.; Noor, Z. N.; Zhang, L.; Klug, K.; Huettel, B.; Hahn, M. Genetic diversity of *Botrytis cinerea* revealed by multilocus sequencing, and identification of B. cinerea populations showing genetic isolation and distinct host adaptation. *Front. Plant Sci.*, **2021**, *12*. https://doi.org/10.3389/fpls.2021.663027
31. Cao, W. Level and structure of SNP and indel variation between individuals on large (North America and Sweden) and small (within Sweden) geographic scale of *Phlebiopsis gigantea*. International Bachelor's Program in Biology - Conservation Biology, Mid Sweden University, Sundsvall, 2014
32. Rampersad, S. N. ITS1, 5.8 S and ITS2 secondary structure modelling for intra-specific differentiation among species of the *Colletotrichum gloeosporioides sensu lato* species complex. *SpringerPlus*, **2014**, *3(1)*, 1-10. https://doi.org/10.1186/2193-1801-3-684
33. Liang, X.; Tian, X.; Liu, W.; Wei, T.; Wang, W.; Dong, Q.; Wang, B.; Meng, Y.; Zhang, R.; Gleason, M. L.; Sun, G. Comparative analysis of the mitochondrial genomes of *Colletotrichum gloeosporioides sensu lato*: insights into the evolution of a fungal species complex interacting with diverse plants. *BMC Genom.*, **2017**, *18(1)*, 1-16. https://doi.org/10.1186/s12864-016-3480-x
34. Fourie, A.; Wingfield, M. J.; Wingfield, B. D.; Barnes, I. Molecular markers delimit cryptic species in *Ceratocystis sensu stricto*. *Mycol. Prog.*, **2015**, *14(1)*, 1-18. https://doi.org/10.1007/s11557-014-1020-0
35. Jorge, V. R.; Silva, M. R., Guillin; E. A., Freire; M. C. M., Schuster, I.; Almeida, A. M. R.; Oliveira, L. O. The origin and genetic diversity of the causal agent of Asian soybean rust, *Phakopsora pachyrhizi*, in South America. *Plant Pathol.*, **2015**, *64(3)*, 729-737. https://doi.org/10.1111/ppa.12300
36. Luo, H.,; Wang, X.; Zhan, G.; Wei, G.; Zhou, X.; Zhao, J.; Huang. L.; Kang, Z. Genome-wide analysis of simple sequence repeats and efficient development of polymorphic SSR markers based on whole genome re-sequencing of multiple isolates of the wheat stripe rust fungus. *PLoS One*, **2015**, *10(6)*, e0130362. https://doi.org/10.1371/journal.pone.0130362
37. Xia, C.; Lei, Y.; Wang, M.; Chen, W.; Chen, X. An avirulence gene cluster in the wheat stripe rust pathogen (*Puccinia striiformis f. sp. tritici*) identified through genetic mapping and whole-genome sequencing of a sexual population. *Msphere*, **2020**, *5(3)*, e00128-20. https://doi.org/10.1128/mSphere.00128-20
38. Ding, Y.; Cuddy, W. S.; Wellings, C. R.; Zhang, P.; Thach, T.; Hovmøller, M. S.; Qutob, D.; Brar, G. S.; Kutcher, H. R.; Park, R. F. Incursions of divergent genotypes, evolution of virulence and host jumps shape a continental clonal population of the stripe rust pathogen *Puccinia striiformis*. *Mol. Ecol.*, **2021**, *30(24)*, 6566-6584. https://doi.org/10.1111/mec.16182. https://doi.org/10.1111/mec.16182
39. Komínková, E.; Dreiseitl, A.; Malečková, E.; Doležel, J.; Valárik, M. Genetic diversity of *Blumeria graminis f. sp. hordei* in Central Europe and its comparison with Australian population. *PloS One*, **2016**, *11(11)*, e0167099. https://doi.org/10.1371/journal.pone.0167099

40. See, P. T.; Moffat, C. S.; Morina, J.; Oliver, R. P. Evaluation of a multilocus indel DNA region for the detection of the wheat tan spot pathogen *Pyrenophora tritici-repentis*. *Plant Dis.*, **2016**, *100(11)*, 2215-2225. https://doi.org/10.1094/PDIS-03-16-0262-RE
41. Depotter, J. R.; Seidl, M. F.; van den Berg, G. C.; Thomma, B. P.; Wood, T. A. A distinct and genetically diverse lineage of the hybrid fungal pathogen *Verticillium longisporum* population causes stem striping in British oilseed rape. *Environ. Microbiol.*, **2017**, *19(10)*, 3997-4009. https://doi.org/10.1111/1462-2920.13801
42. Shinozuka, H.; Hettiarachchige, I. K.; Shinozuka, M.; Cogan, N. O.; Spangenberg, G. C.; Cocks, B. G.; Forster, J. W.; Sawbridge, T. I. Horizontal transfer of a ß-1, 6-glucanase gene from an ancestral species of fungal endophyte to a cool-season grass host. *Sci. Rep.*, **2017**, *7(1)*, 1-11. https://doi.org/10.1038/s41598-017-07886-2
43. Laurent, B.; Palaiokostas, C.; Spataro, C.; Moinard, M.; Zehraoui, E.; Houston, R. D.; Foulongne-Oriol, M. High-resolution mapping of the recombination landscape of the phytopathogen *Fusarium graminearum* suggests two-speed genome evolution. *Mol. Plant Pathol.*, **2018**, *19(2)*, 341-354. https://doi.org/10.1111/mpp.12524
44. McTaggart, A. R.; Aime M. C. The species of *Coleosporium* (Pucciniales) on *Solidago* in North America. *Fungal Biol.*, **2018**, *122(8)*, 800-809. https://doi.org/10.1016/j.funbio.2018.04.007
45. Zhong, Z.; Chen, M.; Lin, L.; Han, Y.; Bao, J.; Tang, W.; Lin, L.; Lin, Y.; Somai, R.; Lu, L.; Zhang, W.; Chen, J.; Hong, Y.; Chen, X.; Wang, B.; Shen, W.-C.; Lu, G.; Norvienyeku, J.; Ebbole, D. J.; Wang, Z. Population genomic analysis of the rice blast fungus reveals specific events associated with expansion of three main clades. *ISME J.*, **2018**, *12(8)*, 1867-1878. https://doi.org/10.1038/s41396-018-0100-6. https://doi.org/10.1038/s41396-018-0100-6
46. Zubaer, A.; Wai, A.; Hausner, G. The mitochondrial genome of *Endoconidiophora resinifera* is intron rich. *Sci. Rep.*, **2018**, *8(1)*, 1-12. https://doi.org/10.1038/s41598-018-35926-y
47. Coleman, C. E.; Meyer, S. E.; Ricks, N. Mating system complexity and cryptic speciation in the seed bank pathogen *Pyrenophora semeniperda*. *Plant Pathol.*, **2019**, *68(2)*, 369-382. https://doi.org/10.1111/ppa.12948
48. Kim, W.; Lichtenzveig, J.; Syme, R. A.; Williams, A. H.; Peever, T. L.; Chen, W. Identification of a polyketide synthase gene responsible for ascochitine biosynthesis in *Ascochyta fabae* and its abrogation in sister taxa. *Msphere*, **2019**, *4(5)*, e00622-19. https://doi.org/10.1128/mSphere.00622-19
49. Liu, X.; Xing, M.; Kong, C.; Fang, Z.; Yang, L.; Zhang, Y.; Wang, Y.; Ling, J.; Yang, Y.; Lv, H. Genetic diversity, virulence, race profiling, and comparative genomic analysis of the *Fusarium oxysporum f. sp. conglutinans* strains infecting cabbages in China. *Front. Microbiol.*, **2019**, 1373. https://doi.org/10.3389/fmicb.2019.01373
50. Richards, J. K.; Stukenbrock, E. H.; Carpenter, J.; Liu, Z.; Cowger, C.; Faris, J. D.; Friesen, T. L. Local adaptation drives the diversification of effectors in the fungal wheat pathogen *Parastagonospora nodorum* in the United States. *PLoS Genet.*, **2019**, *15(10)*, e1008223. https://doi.org/10.1371/journal.pgen.1008223
51. Kulik, T.; Brankovics, B.; Van Diepeningen, A. D.; Bilska, K.; Żelechowski, M.; Myszczyński, K.; Molcan, T:; Stakheev, A.; Stenglein, S.; Beyer, M.; Pasquali, M.; Sawicki, J.; Wyrębek, J.; Baturo-Cieśniewska, A. Diversity of mobile genetic elements in the mitogenomes of closely related *Fusarium culmorum* and *F. graminearum sensu stricto* strains and its implication for diagnostic purposes. *Front. Microbiol.*, **2020**, *11*, 1002. https://doi.org/10.3389/fmicb.2020.01002
52. Yang, M.; Zhang, H.; Van der Lee, T. A.; Waalwijk, C.; Van Diepeningen, A. D.; Feng, J.; Brankovics, B.; Chen, W. Population genomic analysis reveals a highly conserved mitochondrial genome in *Fusarium asiaticum*. *Front. Microbiol.*, **2020**, *11*, 839. https://doi.org/10.3389/fmicb.2020.00839
53. Chen, Q.; Peng, G.; Kutcher, R.; Yu, F. Genetic diversity and population structure of *Leptosphaeria maculans* isolates in Western Canada. *J. Genet. Genom.*, **2021**, *48(11)*, 994-1006. https://doi.org/10.1016/j.jgg.2021.06.019
54. Li, S.; Deng, Y. Mitochondrial genome resource of *Phomopsis longicolla*, a fungus causing phomopsis seed decay in soybean. *PhytoFrontiers*, **2021**, *1(2)*, 120-122. https://doi.org/10.1094/PHYTOFR-10-20-0027-A
55. Weldon, W. A.; Knaus, B. J.; Grünwald, N. J.; Havill, J. S.; Block, M. H.; Gent, D. H.; Cadle-Davidson, L. E.; Gadoury, D. M. Transcriptome-derived amplicon sequencing markers elucidate the US *Podosphaera macularis* population structure across feral and commercial plantings of *Humulus lupulus*. *Phytopathology*, **2021**, *111(1)*, 194-203. https://doi.org/10.1094/PHYTO-07-20-0299-FI
56. Ahmad, F.; Baric, S. Genetic diversity of *Cryphonectria parasitica* causing chestnut blight in South Tyrol (northern Italy). *Eur. J. Plant Pathol.*, **2022**, *162*, 621–635. https://doi.org/10.1007/s10658-021-02425-2
57. McCluskey, K. Variation in mitochondrial genome primary sequence among whole-genome-sequenced strains of *Neurospora crassa*. *IMA fungus*, **2012**, *3(1)*, 93-98. https://doi.org/10.5598/imafungus.2012.03.01.10
58. Hansen, K.; Perry, B. A.; Dranginis, A. W.; Pfister, D. H. A phylogeny of the highly diverse cup-fungus family Pyronemataceae (Pezizomycetes, Ascomycota) clarifies relationships and evolution of selected life history traits. *Mol. Phylogenet. Evol.*, **2013**, *67(2)*, 311-335. https://doi.org/10.1016/j.ympev.2013.01.014
59. Tóth, A.; Hausknecht, A.; Krisai-Greilhuber, I.; Papp, T.; Vágvölgyi, C.; Nagy, L. G. Iteratively refined guide trees help improving alignment and phylogenetic inference in the mushroom family Bolbitiaceae. *PLoS One*, **2013**, *8(2)*, e56143. https://doi.org/10.1371/journal.pone.0056143
60. Fan, X.; Zhou, Y.; Bian, Y. Rapid identification of protoplast-regenerated monokaryotic isolates of *Auricularia auricula-judae* based on an allele InDel marker. *Mycosystema*, **2014**, *33(2)*, 273-279. ISSN : 1672-6472
61. Gambhir, N.; Kamvar, Z. N.; Higgins, R.; Amaradasa, B. S.; Everhart, S. E. Spontaneous and fungicide-induced genomic variation in *Sclerotinia sclerotiorum*. *Phytopathol.*, **2021**, *111(1)*, 160-169. https://doi.org/10.1094/PHYTO-10-20-0471-FI
62. Solieri, L.; Dakal, T. C.; Giudici, P. *Zygosaccharomyces sapae* sp. nov., isolated from Italian traditional balsamic vinegar. *Int. J. Syst. Evol. Microbiol.*, **2013**, *63(Pt_1)*, 364-371. https://doi.org/10.1099/ijs.0.043323-0

63. Gong, W. B.; Liu, W.; Lu, Y. Y.; Bian, Y. B.; Zhou, Y.; Kwan, H. S.; Cheung, M. K.;Xiao, Y. Constructing a new integrated genetic linkage map and mapping quantitative trait loci for vegetative mycelium growth rate in *Lentinula edodes*. *Fungal Biol.*, **2014**, *118(3)*, 295-308. https://doi.org/10.1016/j.funbio.2014.01.001
64. Li, C.; Gong, W.; Zhang, L.; Yang, Z.; Nong, W.; Bian, Y.; Kwan, H.-S.; Cheung, M.-K.; Xiao, Y. Association mapping reveals genetic loci associated with important agronomic traits in *Lentinula edodes*, shiitake mushroom. *Front. Microbiol.*, **2017**, *8*, 237. https://doi.org/10.3389/fmicb.2017.00237
65. Dong, H.; Shang, X.; Zhao, X.; Yu, H.; Jiang, N.; Zhang, M.; Tan, Q.; Zhou, C.; Zhang, L. Construction of a genetic linkage map of *Lentinula edodes* based on SSR, SRAP and TRAP markers. *Breed. Sci.*, **2019**, 18123. https://doi.org/10.1270/jsbbs.18123
66. Zhang, L.; Gong, W.; Li, C.; Shen, N.; Gui, Y.; Bian, Y.; Kwan, H. S.; Cheung, M. K.; Xiao, Y. RNA-Seq-based high-resolution linkage map reveals the genetic architecture of fruiting body development in shiitake mushroom, *Lentinula edodes*. *Comput. Struct. Biotechnol. J.*, **2021**, *19*, 1641-1653. https://doi.org/10.1016/j.csbj.2021.03.016
67. Callicott, K. A.; Cotty, P. J. Method for monitoring deletions in the aflatoxin biosynthesis gene cluster of *Aspergillus flavus* with multiplex PCR. *Lett. App. Microbiol.*, **2015**, *60(1)*, 60-65. https://doi.org/10.1111/lam.12337
68. Im, C. H.; Park, Y. H.; Hammel, K. E.; Park, B.; Kwon, S. W.; Ryu, H.; Ryu, J. S. Construction of a genetic linkage map and analysis of quantitative trait loci associated with the agronomically important traits of *Pleurotus eryngii*. *Fungal Genet. Biol.*, **2016**, *92*, 50-64. https://doi.org/10.1016/j.fgb.2016.05.002
69. Xiang, X.; Li, C.; Li, L.; Bian, Y.; Kwan, H. S.; Nong, W.; Cheung, M. K.; Xiao, Y. Genetic diversity and population structure of Chinese *Lentinula edodes* revealed by InDel and SSR markers. *Mycol. Prog.*, **2016**, *15(4)*, 1-13
70. Song, X.; Zhao, Y.; Song, C.; Chen, M.; Huang, J.; Bao, D.; Tan, Q.; Yang, R. Mitogenome types of *two Lentinula edodes sensu lato* populations in China. *Sci. Rep.*, **2019**, *9(1)*, 1-11. https://doi.org/10.1038/s41598-019-45922-5
71. Moon, S.; Hong, C. P.; Ryu, H.; Lee, H. Y. Development of cleaved amplified polymorphic sequence markers of *Lentinula edodes* Cultivars Sanbaekhyang and Sulbaekhyang. *Kor. J. Mycol.*, **2021**, *49(1)*, 33-44. https://doi.org/10.4489/KJM.20210004
72. Faustinelli, P. C.; Palencia, E. R.; Sobolev, V. S.; Horn, B. W.; Sheppard, H. T.; Lamb, M. C.; Wang , X. M.; Scheffler, B. E.; Martinez Castillo, J.; Arias, R. S. Study of the genetic diversity of the aflatoxin biosynthesis cluster in *Aspergillus* section Flavi using insertion/deletion markers in peanut seeds from Georgia, USA. *Mycologia*, **2017**, *109(2)*, 200-209, https://doi.org/10.1080/00275514.2017.1307095
73. Arias, R. S.; Sobolev, V. S.; Massa, A. N.; Orner, V. A.; Walk, T. E.; Ballard, L. L.; Simpson, S. A.; Puppala, N.; Scheffler, B. E.; de Blas, F.; Seijo, G. J. New tools to screen wild peanut species for aflatoxin accumulation and genetic fingerprinting. *BMC Plant Biol.*, **2018**, *18(1)*, 1-13. https://doi.org/10.1186/s12870-018-1355-9
74. Acur, A.; Arias, R.S.; Odongo, S.; Tuhaise, S.; Ssekandi, J.; Adriko, J.; Muhanguzi, D.; Buah, S.; Kiggundu, A.. Genetic diversity of aflatoxin-producing *Aspergillus flavus* isolated from selected groundnut growing agro-ecological zones of Uganda. *ResearchSquare*
75. Acur, A.; Arias, R.S.; Odongo, S.; Tuhaise, S.; Ssekandi, J.; Adriko, J.; Muhanguzi, D.; Buah, S.; Kiggundu, A.. Genetic diversity of aflatoxin-producing *Aspergillus flavus* isolated from selected groundnut growing agro-ecological zones of Uganda. *BMC Microbiol.*, **2020**, *20*, 252. https://doi.org/10.1186/s12866-020-01924-2
76. Mohammed, A.; Faustinelli, P. C.; Chala, A.; Dejene, M.; Fininsa, C.; Ayalew, A.; Ojiewo, C. O.; Hoisington, D. A.; Sobolev, V. S.; Martínez-Castillo, J.; Arias, R. S. Genetic fingerprinting and aflatoxin production of *Aspergillus* section Flavi associated with groundnut in eastern Ethiopia. *BMC Microbiol.*, **2021**, *21(1)*, 1-12. https://doi.org/10.1186/s12866-021-02290-3
77. Zhong, Y.; Lu, X.; Xing, L.; Ho, S. W. A.; Kwan, H. S. Genomic and transcriptomic comparison of *Aspergillus oryzae* strains: a case study in soy sauce koji fermentation. *J. Ind. Microbiol. Biotechnol.*, **2018**, *45(9)*, 839-853. https://doi.org/10.1007/s10295-018-2059-8
78. Dai, Y., Li, X., Song, B., Sun, L., Yang, C., Zhang, X.; Wang, Y.; Zhang, Z.; Fu, Y.; Li, Y. Genomic analyses provide insights into the evolutionary history and genetic diversity of *Auricularia* species. *Front. Microbiol.*, **2019**, *2255*. https://doi.org/10.3389/fmicb.2019.02255
79. Liu, W.; Zhang, Q.; Shu, F.; Cai, Y.; Ma, X.; Bian, Y. Genome-wide SNP/Indel analysis and the construction of genetic linkage maps based on Indel markers of *Morchella importuna*. *Mycosystema*, **2019**, *38(12)*, 2195-2204. ISSN : 1672-6472
80. Guttová, A.; Zozomová-Lihová, J.; Timdal, E.; Kučera, J.; Slovák, M.; Piknová, K.; Paoli, L. First insights into genetic diversity and relationships of European taxa of *Solenopsora* (Catillariaceae, Ascomycota) with implications for their delimitation. *Bot. J. Linn. Soc.*, **2014**, *176(2)*, 203-223. https://doi.org/10.1111/boj.12200
81. Miadlikowska, J.; Richardson, D.; Magain, N.; Ball, B.; Anderson, F.; Cameron, R.; Lendemer, J.; Truong, C.; Lutzoni, F. Phylogenetic placement, species delimitation, and cyanobiont identity of endangered aquatic *Peltigera* species (lichen-forming Ascomycota, Lecanoromycetes). *Am. J. Botany*, **2014**, *101(7)*, 1141-1156. https://doi.org/10.3732/ajb.1400267
82. Simon, A.; Goffinet, B.; Magain, N.; Sérusiaux, E. High diversity, high insular endemism and recent origin in the lichen genus *Sticta* (lichenized Ascomycota, Peltigerales) in Madagascar and the Mascarenes. *Mol. Phylogenetics Evol.*, **2018**, *122*, 15-28. https://doi.org/10.1016/j.ympev.2018.01.012
83. Wang, Y. Y.; Liu, R. D.; Wang, W. C.; Wei, X.; Wei, J. Nucleotide differences in the mbf1 gene of the lichenized fungus *Umbilicaria decussata* collected in polar and non-polar regions. *Adv. Polar Sci.*, **2017**, *28(1)*, 43-49. https://doi.org/10.13679/j.advps.2017.1.00043
84. Jiang, S. H.; Lücking, R.; Xavier-Leite, A. B.; Cáceres, M. E.; Aptroot, A.; Portilla, C. V.; Wei, J. C. Reallocation of foliicolous species of the genus *Strigula* into six genera (lichenized Ascomycota, Dothideomycetes, Strigulaceae). *Fungal Divers.*, **2020**, *102(1)*, 257-291. https://doi.org/10.1007/s13225-020-00445-7

85. Moncada, B.; Lücking, R.; Lumbsch, H. T. Rewriting the evolutionary history of the lichen genus *Sticta* (Ascomycota: Peltigeraceae subfam. Lobarioideae) in the Hawaiian islands. *Plant Fungal Syst.*, **2020**, *65(1)*, 95-119. https://doi.org/10.35535/pfsyst-2020-0005
86. Zhang, Y. J.; Yang, X. Q.; Zhang, S.; Humber, R. A.; Xu, J. Genomic analyses reveal low mitochondrial and high nuclear diversity in the cyclosporin-producing fungus *Tolypocladium inflatum*. *App. Microbiol. Biotechnol.*, **2017**, *101(23)*, 8517-8531. https://doi.org/10.1007/s00253-017-8574-0
87. Zhang, S.; Hao, A. J.; Zhao, Y. X.; Zhang, X. Y.; Zhang, Y. J. Comparative mitochondrial genomics toward exploring molecular markers in the medicinal fungus *Cordyceps militaris*. *Sci. Rep.*, **2017**, *7(1)*, 1-9. https://doi.org/10.1038/srep40219
88. Zhang, X.; Xu, Z.; Pei, H.; Chen, Z.; Tan, X.; Hu, J.; Yang, B.; Sun, J. Intraspecific variation and phylogenetic relationships are revealed by ITS1 secondary structure analysis and single-nucleotide polymorphism in *Ganoderma lucidum*. *PLoS One*, **2017**, *12(1)*, e0169042. https://doi.org/10.1371/journal.pone.0169042
89. Wang, G.; Lin, J.; Shi, Y.; Chang, X.; Wang, Y.; Guo, L; Wang, W.; Dou, M.; Deng, Y.; Ming, R.; Zhang, J. Mitochondrial genome in *Hypsizygus marmoreus* and its evolution in Dikarya. *BMC Genom.*, **2019**, *20(1)*, 1-11. https://doi.org/10.1186/s12864-019-6133-z
90. Gong, W.; Wang, Y.; Xie, C.; Zhou, Y.; Zhu, Z.; Peng, Y. Whole genome sequence of an edible and medicinal mushroom, *Hericium erinaceus* (Basidiomycota, Fungi). *Genomics*, **2020**, *112(3)*, 2393-2399. https://doi.org/10.1016/j.ygeno.2020.01.011
91. Jiang, W. Z., Yao, F. J., Lu, L. X., Fang, M., Wang, P., Zhang, Y. M.; Meng, J.-J.; Lu, J.; Ma, X.-X.; He, Q.; Shao, K. S. Genetic linkage map construction and quantitative trait loci mapping of agronomic traits in *Gloeostereum incarnatum*. *J. Microbiol.*, **2021**, *59(1)*, 41-50. https://doi.org/10.1007/s12275-021-0242-5
92. Jiang, W. Z.; Yao, F. J.; Fang, M.; Lu, L. X.; Zhang, Y. M.; Wang, P.; Meng, J.-J.; Lu, J.; Ma, X.-X.; He, Q.; Shao, K.-S.; Khan, A. A.; Wei, Y. H. Analysis of the genome sequence of strain GiC-126 of *Gloeostereum incarnatum* with genetic linkage map. *Mycobiol.*, **2021**, *49(4)*, 406-420. https://doi.org/10.1080/12298093.2021.1954321
93. Nagel, J. H.; Gryzenhout, M.; Slippers, B.; Wingfield, M. J.; Hardy, G. E. S. J.; Stukely, M. J.; Burgess, T. I. Characterization of *Phytophthora* hybrids from ITS clade 6 associated with riparian ecosystems in South Africa and Australia. *Fungal Biol.*, **2013**, *117(5)*, 329-347. https://doi.org/10.1016/j.funbio.2013.03.004
94. Burgess, T. I. Molecular characterization of natural hybrids formed between five related indigenous clade 6 *Phytophthora* species. *PLoS One*, **2015**, *10(8)*, e0134225. https://doi.org/10.1371/journal.pone.0134225
95. Stamler, R. A.; Sanogo, S.; Goldberg, N. P.; Randall, J. J. *Phytophthora* species in rivers and streams of the southwestern United States. *App. Environ. Microbiol.*, **2016**, *82(15)*, 4696-4704. https://doi.org/10.1128/AEM.01162-16
96. Grubisha, L. C.; Levsen, N.; Olson, M. S.; Lee Taylor, D. Intercontinental divergence in the *Populus*-associated ectomycorrhizal fungus, *Tricholoma populinum*. *New Phytol.*, **2012**, *194(2)*, 548-560. https://doi.org/10.1111/j.1469-8137.2012.04066.x
97. Thiéry, O.; Moora, M.; Vasar, M.; Zobel, M.; Öpik, M. Inter-and intrasporal nuclear ribosomal gene sequence variation within one isolate of arbuscular mycorrhizal fungus, *Diversispora* sp. *Symbiosis*, **2012**, *58(1)*, 135-147. https://doi.org/10.1007/s13199-012-0212-0
98. Borriello, R.; Bianciotto, V.; Orgiazzi, A.; Lumini, E.; Bergero, R. Sequencing and comparison of the mitochondrial COI gene from isolates of Arbuscular Mycorrhizal Fungi belonging to Gigasporaceae and Glomeraceae families. *Mol. Phylogenetics Evol.*, **2014**, *75*, 1-10. https://doi.org/10.1016/j.ympev.2014.02.012
99. Błaszkowski, J.; Niezgoda, P.; de Paiva, J. N.; da Silva, K. J. G.; Theodoro, R. C.; Jobim, K.; Orfanoudakis, M.; Goto, B. T. *Sieverdingia* gen. nov., *S. tortuosa* comb. nov., and *Diversispora peloponnesiaca* sp. nov. in the Diversisporaceae (Glomeromycota). *Mycol. Prog.*, **2019**, *18(11)*, 1363-1382. https://doi.org/10.1007/s11557-019-01534-x
100. Błaszkowski, J.; Niezgoda, P.; Zubek, S.; Meller, E.; Milczarski, P.; Malicka, M.; Goto, B. T.; Woźniak, G.; Moreira, H.; Magurno, F. *Dominikia bonfanteae* and *Glomus atlanticum*, two new species in the Glomeraceae (phylum Glomeromycota) with molecular phylogenies reconstructed from two unlinked loci. *Mycol. Prog.*, **2021**, *20(2)*, 131-148. https://doi.org/10.1007/s11557-020-01659-4
101. Yu, F.; Goto, B. T.; Magurno, F.; Błaszkowski, J.; Wang, J.; Ma, W.; Feng, H.; Liu, Y. *Glomus chinense* and *Dominikia gansuensis*, two new Glomeraceae species of arbuscular mycorrhizal fungi from high altitude in the Tibetan Plateau. *Mycol. Prog.*, **2022**, *21(2)*, 1-11. https://doi.org/10.1007/s11557-022-01799-9
102. Chen, L.; Gong, Y.; Cai, Y.; Liu, W.; Zhou, Y.; Xiao, Y.; Xu, Z.; Liu, Y.; Lei, X.; Wang, G.; Guo, M.; Ma, X.; Bian, Y. Genome sequence of the edible cultivated mushroom *Lentinula edodes* (Shiitake) reveals insights into lignocellulose degradation. *PloS One*, **2016**, *11(8)*, e0160336. https://doi.org/10.1371/journal.pone.0160336
103. Aamri, L. E.; Hafidi, M.; Scordino, F.; Krasowska, A.; Lebrihi, A.; Orlando, M. G.; Barresi, C.; Criseo, G.; Barreca, D.; Romeo, O. *Arthrographis curvata* and *Rhodosporidium babjevae* as new potential fungal lipase producers for biotechnological applications. *Braz. Arch. Biol. Technol.*, **2020**, *63*. https://doi.org/10.1590/1678-4324-2020180444
104. Brandt, S. C.; Ellinger, B.; Van Nguyen, T.; Harder, S.; Schlüter, H.; Hahnke, R. L.; Rühl, M.; Schäfer, W.; Gand, M. *Aspergillus sydowii*: genome analysis and characterization of two heterologous expressed, non-redundant xylanases. *Front. Microbiol.*, **2020**, 2154. https://doi.org/10.3389/fmicb.2020.573482
105. Li, W. C.; Lee, C. Y.; Lan, W. H.; Woo, T. T.; Liu, H. C.; Yeh, H. Y.; Chang, H.-Y.; Chuang, Y.-C.; Chen, C.-Y.; Chuang, C.-N. ; Chen, C.-L.; Hsueh, Y.-P.; Li, H.-W.;Wang, T. F. *Trichoderma reesei* Rad51 tolerates mismatches in hybrid meiosis with diverse genome sequences. *PNAS*, **2021**, *118(8)*, https://doi.org/10.1073/pnas.2007192118

References: Supplementary Table S8

1. Huang, C. H.; Chang, M. T.; Huang, L. Species identification of *Wickerhamomyces anomalus* and related taxa using β-tubulin (β-tub) DNA barcode marker. *Yeast*, **2012**, *29(12)*, 531-535. https://doi.org/10.1002/yea.2933
2. Walther, G.; Pawłowska, J.; Alastruey-Izquierdo, A.; Wrzosek, M.; Rodriguez-Tudela, J. L.; Dolatabadi, S.; Chakrabarti, A.; De Hoog, G. S. DNA barcoding in Mucorales: an inventory of biodiversity. *Pers.: Mol. Phylogeny Evol. Fungi*, **2013**, *30(1)*, 11-47. https://doi.org/10.3767/003158513X665070
3. Cleland, E. J.; Bassioni, A.; Boase, S.; Dowd, S.; Vreugde, S.; Wormald, P. J. The fungal microbiome in chronic rhinosinusitis: richness, diversity, postoperative changes and patient outcomes. *Int. Forum Allergy Rhinol.*, **2014**, *4(4)*, 259-265. https://doi.org/10.1002/alr.21297
4. Yu, J.; Walther, G.; Van Diepeningen, A. D.; Gerrits Van Den Ende, A. H. G.; Li, R. Y.; Moussa, T. A. A.; Almaghrabi, O. A.; De Hoog, G. S. DNA barcoding of clinically relevant *Cunninghamella* species. *Med. Mycol.*, **2015**, *53(2)*, 99–106. https://doi.org/10.1093/mmy/myu079
5. Zeng, J.; Feng, P.; van den Ende, A. H. G.; Xi, L.; Harrak, M. J.; De Hoog, G. S. Multilocus analysis of the *Exophiala jeanselmei* clade containing black yeasts involved in opportunistic disease in humans. *Fungal Divers*., **2014**, *65(1)*, 3-16. https://doi.org/10.1007/s13225-013-0226-9
6. Al-Hatmi, A. M.; Van Den Ende, A. G.; Stielow, J. B.; Van Diepeningen, A. D.; Seifert, K. A.; McCormick, W.; Assabgui, R.; Gräfenhand, T.; De Hoog, G. S.; Levesque, C. A. Evaluation of two novel barcodes for species recognition of opportunistic pathogens in *Fusarium*. *Fungal Biol.*, **2016**, *120(2)*, 231-245. https://doi.org/10.1016/j.funbio.2015.08.006
7. Chen, M.; Zeng, J.; De Hoog, G. S.; Stielow, B.; Van Den Ende, A. G.; Liao, W.; Lackner, M. The 'species complex'issue in clinically relevant fungi: a case study in *Scedosporium apiospermum*. *Fungal Biol.*, **2016**, *120(2)*, 137-146. https://doi.org/10.1016/j.funbio.2015.09.003
8. Fidler, G.; Kocsube, S.; Leiter, E.; Biro, S.; Paholcsek, M. DNA barcoding coupled with high resolution melting analysis enables rapid and accurate distinction of *Aspergillus* species. *Med. Mycol.*, **2017**, *55(6)*, 642-659. https://doi.org/10.1093/mmy/myw127
9. Normand, A. C.; Packeu, A.; Cassagne, C.; Hendrickx, M.; Ranque, S.; Piarroux, R. Nucleotide sequence database comparison for routine dermatophyte identification by internal transcribed spacer 2 genetic region DNA barcoding. *J. Clin. Microbiol.*, **2018**, *56(5)*, e00046-18. https://doi.org/10.1128/JCM.00046-18
10. Hoang, M. T. V.; Irinyi, L.; Chen, S. C.; Sorrell, T. C.; ISHAM Barcoding of medical fungi working group; Meyer, W. Dual DNA barcoding for the molecular identification of the agents of invasive fungal infections. *Front. Microbiol.*, **2019**, *10*, 1647. https://doi.org/10.3389/fmicb.2019.01647
11. Hung, N.; Yeh, L. K.; Ma, D. H. K.; Lin, H. C.; Tan, H. Y.; Chen, H. C.; Sun, P.-L.; Hsiao, C. H. Filamentous fungal keratitis in Taiwan: based on molecular diagnosis. *Transl. Vis. Sci. Technol.*, **2020**, *9(8)*,32-32. https://doi.org/10.1167/tvst.9.8.32
12. Pinheiro, B. G.; Hahn, R. C.; Camargo, Z. P. D.; Rodrigues, A. M. Molecular tools for detection and identification of *Paracoccidioides* species: Current status and future perspectives. *J. Fungi*, **2020**, *6(4)*, 293. https://doi.org/10.3390/jof6040293
13. Chen, S. C. A.; Halliday, C. L.; Hoenigl, M.; Cornely, O. A.; Meyer, W. *Scedosporium* and *Lomentospora* infections: contemporary microbiological tools for the diagnosis of invasive disease. *J. Fungi*, **2021**, *7(1)*, 23. https://doi.org/10.3390/jof7010023
14. Gunasekaran, R.; Janakiraman, D.; Rajapandian, S. G. K.; Appavu, S. P.; Venkatesh, P. N.; Prajna, L. *Periconia* species-An unusual fungal pathogen causing mycotic keratitis. *Indian J. Med. Microbiol.*, **2021**, *39(1)*, 36-40. https://doi.org/10.1016/j.ijmmb.2020.10.006
15. Mar Htun, Z.; Laikul, A.; Pathomsakulwong, W.; Yurayart, C.; Lohnoo, T.; Yingyong, W.; Kumsang, Y.; Payattikul, P.; Sae-Chew, P.; Rujirawat, T.; Jittorntam, P.; Jaturapaktrarak, C.; Chongtrakool, P.; Krajaejun, T. Identification and biotyping of *Pythium insidiosum* isolated from urban and rural areas of Thailand by multiplex PCR, DNA barcode, and proteomic analyses. *J. Fungi*, **2021**, *7(4)*, 242. https://doi.org/10.3390/jof7040242
16. Attili-Angelis, D.; Duarte, A. P. M.; Pagnocca, F. C.; Nagamoto, N. S.; De Vries, M.; Stielow, J. B.; De Hoog, G. S. Novel *Phialophora* species from leaf-cutting ants (tribe Attini). *Fungal Divers.*, **2014**, *65(1)*, 65-75. https://doi.org/10.1007/s13225-013-0275-0
17. Gouliamova, D. E.; Dimitrov, R. A.; Smith, M. T.; Groenewald, M.; Stoilova-Disheva, M. M.; Guéorguiev, B. V.; Boekhout, T. DNA barcoding revealed *Nematodospora valgi* gen. nov., sp. nov. and *Candida cetoniae* sp. nov. in the Lodderomyces clade. *Fungal Biol.*, **2016**, *120(2)*, 179-190. https://doi.org/10.1016/j.funbio.2015.05.008
18. More, S. N.; Hernandez, O.; Castleman, W. L. Mycotic rhinitis and sinusitis in Florida horses. *Vet. Pathol.*, **2019**, *56(4)*,586-598. https://doi.org/10.1177/0300985818817046
19. Misra, P. K.; Gautam, N. K.; Elangovan, V. Morphological and molecular characterizations of guanophilic fungi of bats. *Proc. Natl. Acad. Sci. India Sect B Biol. Sci.*, **2020**, *90(3)*, 595-604. https://doi.org/10.1007/s40011-019-01129-2
20. Irinyi, L.; Serena, C.; Garcia-Hermoso, D.; Arabatzis, M.; Desnos-Ollivier, M.; Vu, D.; Cardinali, G.; Arthur, I.; Normand, A.-C.; Giraldo, A.; da Cunha, K. C.; Sandoval-Denis, M.; Hendrickx, M.; Nishikaku, A. S.; Melo, A. S. A.; Merseguel, K. B.; Khan, A.; Rocha, J. A. P.; Sampaio, P.; Briones, M. R. S.; Ferreira, R. C.; Muniz, M. M.; Castañón-Olivares, L. R.; Estrada-Barcenas, D.; Cassagne, C.; Mary, C.; Duan, S. Y.; Kong, F.; Sun, A. Y.; Zeng, X.; Zhao, Z.; Gantois, N.; Botterel, F.; Robbertse, B.; Schoch, C.; Gams, W.; Ellis, D.; Halliday, C.; Chen, S.; Sorrell, T. C.; Piarroux, R.; Colombo, A. L.; Pais, C.; de Hoog, S.; Zancopé-Oliveira, R. M.; Taylor, M. L.; Toriello, C.; Soares, C. M. A.; Delhaes, L.; Stubbe, D.; Dromer, F.; Ranque, S.; Guarro, J.; Cano-Lira, J. F.; Robert, V.; Velegraki, A.; Meyer, W. (2015). International Society of Human and Animal Mycology (ISHAM)-ITS reference DNA barcoding database—the quality controlled standard tool for routine identification of human and animal pathogenic fungi. *Med. Mycol.*, **2015**, *53(4)*, 313-337. https://doi.org/10.1093/mmy/myv008

21. Quaedvlieg, W.; Groenewald, J. Z.; de Jesús Yáñez-Morales, M.; Crous, P. W. DNA barcoding of *Mycosphaerella* species of quarantine importance to Europe. *Pers.: Mol. Phylogeny Evol. Fungi*, **2012**, *29(1)*, 101-115. https://doi.org/10.3767/003158512X661282
22. Zeng, Z,; Zhao, P.; Luo, J.; Zhuang, W.; Yu, Z. Selection of a DNA barcode for Nectriaceae from fungal whole-genomes. *Sci. China Life Sci.*, **2012**, *55(1)*, 80-88. https://doi.org/10.1007/s11427-012-4266-2
23. Gao, R.; Zhang, G. Potential of DNA barcoding for detecting quarantine fungi. *Phytopathol.*, **2013**, *103(11)*, 1103-1107. https://doi.org/10.1094/PHYTO-12-12-0321-R
24. Glen, M.; Yuskianti, V.; Puspitasari, D.; Francis, A.; Agustini, L.; Rimbawanto, A.; Indrayadi, H.; Gafur, A.; Mohammed, C. L. Identification of basidiomycete fungi in Indonesian hardwood plantations by DNA barcoding. *Forest Pathol.*, **2014**, *44(6)*, 496-508. https://doi.org/10.1111/efp.12146
25. Al-Hatmi, A. M.; Mirabolfathy, M.; Hagen, F.; Normand, A. C.; Stielow, J. B.; Karami-Osbo, R.; van Diepeningen, A. D.; Meis, J. F.; De Hoog, G. S. DNA barcoding, MALDI-TOF, and AFLP data support *Fusarium ficicrescens* as a distinct species within the *Fusarium fujikuroi* species complex. *Fungal Biol.*, **2016**, *120(2)*, 265-278. https://doi.org/10.1016/j.funbio.2015.08.001
26. Khemmuk, W.,; Shivas, R. G.; Henry, R. J.; Geering, A. D. Fungi associated with foliar diseases of wild and cultivated rice (*Oryza* spp.) in northern Queensland. *Australas. Plant Pathol.*, **2016**, *45(3)*, 297-308. https://doi.org/10.1007/s13313-016-0418-3
27. Lei, Y.; Kuang, W.; Zheng, C.; Li, X.; Gao, W.; Li, C. Detection and identification of the genus *Fusarium* by DNA barcoding. *Acta Phytophylacica Sinica*, **2016**, *43(4)*, 544-551. ISSN : 0577-7518
28. Lutz, M.; Piątek, M. Phylogenetic placement, DNA barcoding, morphology and evidence for the spreading of *Entyloma cosmi*, a species attacking *Cosmos bipinnatus* in temperate climate gardens. *Eur. J. Plant Pathol.*, **2016**, *145(4)*, 857-869. https://doi.org/10.1007/s10658-016-0874-1
29. Stokholm, M. S.; Wulff, E. G.; Zida, E. P.; Thio, I. G.; Néya, J. B.; Soalla, R. W.; Głazowska, S. E.; Henrik, M. A.; Topbjerg, B.; Boelt, B.; Lund, O. S. DNA barcoding and isolation of vertically transmitted ascomycetes in sorghum from Burkina Faso: *Epicoccum sorghinum* is dominant in seedlings and appears as a common root pathogen. *Microbiol. Res.*, **2016**, *191*, 38-50. https://doi.org/10.1007/s10658-016-0874-1
30. Beenken, L.; Lutz, M.; Scholler, M. DNA barcoding and phylogenetic analyses of the genus *Coleosporium* (Pucciniales) reveal that the North American goldenrod rust *C. solidaginis* is a neomycete on introduced and native *Solidago* species in Europe. *Mycol. Prog.*, **2017**, *16(11)*, 1073-1085. https://doi.org/10.1007/s11557-017-1357-2
31. Katoch, A.; Sharma, P.; Sharma, P. N. Identification of *Colletotrichum* spp. associated with fruit rot of *Capsicum annuum* in North Western Himalayan region of India using fungal DNA barcode markers. *J. Plant Biochem. Biotechnol.*, **2017**, *26(2)*, 216-223. https://doi.org/10.1007/s13562-016-0384-4
32. Marin-Felix, Y.; Hernández-Restrepo, M.; Iturrieta-González, I.; García, D.; Gené, J.; Groenewald, J. Z.; Cai, L.; Chen, Q.; Quaedvlieg, W.; Schumacher, R. K.; Taylor, P.W.J.; Ambers, C.; Bonthond, G.; Edwards, J.;. Krueger-Hadfield, S.A; Luangsaard, J. J.; Morton, L.; Moslemi, A.; Sandoval-Denis, M.; Tan, Y.P.; Thangavel, R.; Vaghefi, N.; Cheewangkoon, R.; Crous, P. W. Genera of phytopathogenic fungi: GOPHY 3. *Stud. Mycol.*, **2019**, *94*, 1-124. https://doi.org/10.1016/j.simyco.2019.05.001
33. Nagy, T.; Pfliegler, W. P.; Takács, A.; Tökölyi, J.; Molnár, A. Distribution, infection rates and DNA barcoding of *Uromyces erythronii* (Pucciniaceae), a parasite of *Erythronium* (Liliaceae) in Europe. *Willdenowia*, **2019**, *49(1)*, 13-20. https://doi.org/10.3372/wi.49.49103
34. Félix, C. R.; Andrade, D. A.; Almeida, J. H.; Navarro, H. M. C.; Fell, J. W.; Landell, M. F. *Vishniacozyma alagoana* sp. nov. a tremellomycetes yeast associated with plants from dry and rainfall tropical forests. *Int. J. Syst. Evol. Microbiol.*, **2020**, *70(5)*, 3449-3454. https://doi.org/10.1099/ijsem.0.004193
35. Choudhary, P.; Singh, B. N.; Chakdar, H.; Saxena, A. K. DNA barcoding of phytopathogens for disease diagnostics and bio-surveillance. *World J. Microbiol. Biotechnol.*, **2021**, *37(3)*, 1-20. https://doi.org/10.1007/s11274-021-03019-0
36. Feau, N.; Ramsfield, T. D.; Myrholm, C. L.; Tomm, B.; Cerezke, H. F.; Benowicz, A.; Samis, E.; Romano, A.; Dale, A. L.; Capron, A.; Heinzelmann, R.; Hamelin, R. C. DNA-barcoding identification of *Dothistroma septosporum* on *Pinus contorta* var. *latifolia*, *P. banksiana* and their hybrid in northern Alberta, Canada. *Can. J. Plant Pathol.*, **2021**, *43(3)*, 472-479. https://doi.org/10.1080/07060661.2020.1829065
37. Hami, A.; Rasool, R. S.; Khan, N. A.; Mansoor, S.; Mir, M. A.; Ahmed, N.; Masoodi, K. Z. Morpho-molecular identification and first report of *Fusarium equiseti* in causing chilli wilt from Kashmir (Northern Himalayas). *Sci. Rep.*, **2021**, *11(1)*, 1-14. https://doi.org/10.1038/s41598-021-82854-5
38. Le, X. T.; Nguyen Le, Q. H.; Pham, N. D.; Duong, V. H.; Dentinger, B.; Moncalvo, J. M. *Tomophagus cattienensis* sp. nov., a new Ganodermataceae species from Vietnam: Evidence from morphology and ITS DNA barcodes. *Mycol. Prog.*, **2012**, *11(3)*, 775-780. https://doi.org/10.1007/s11557-011-0789-3
39. Udayanga, D.; Liu, X.; Crous, P. W.; McKenzie, E. H.; Chukeatirote, E.; Hyde, K. D. A multi-locus phylogenetic evaluation of *Diaporthe* (*Phomopsis*). *Fungal Divers.*, **2012**, *56(1)*, 157-171. https://doi.org/10.1007/s13225-012-0190-9
40. Suwannasai, N.; Martin, M. P.; Phosri, C.; Sihanonth, P.; Whalley, A. J.; Spouge, J. L. Fungi in Thailand: a case study of the efficacy of an ITS barcode for automatically identifying species within the *Annulohypoxylon* and *Hypoxylon* genera. *PLoS One*, **2013**, *8(2)*, e54529. https://doi.org/10.1371/journal.pone.0054529
41. Wagner, L.; Stielow, B.; Hoffmann, K.; Petkovits, T.; Papp, T.; Vágvölgyi, C.; de Hoog, G.S.; Verkley, G.; Voigt, K. A comprehensive molecular phylogeny of the Mortierellales (Mortierellomycotina) based on nuclear ribosomal DNA. *Pers.: Mol. Phylogeny Evol. Fungi*, **2013**, *30(1)*, 77-93. https://doi.org/10.3767/003158513X666268
42. Dolatabadi, S.; Walther, G.; Gerrits Van Den Ende, A. H. G.; De Hoog, G. S. Diversity and delimitation of *Rhizopus microsporus*. *Fungal Divers.*, **2014**, *64(1)*, 145-163. https://doi.org/10.1007/s13225-013-0229-6

43. Zhou, X.; Rodrigues, A. M.; Feng, P.; De Hoog, G. S. Global ITS diversity in the *Sporothrix schenckii* complex. *Fungal Divers.*, **2014**, *66(1)*, 153-165. https://doi.org/10.1007/s13225-013-0220-2
44. Balasundaram, S. V.; Engh, I. B.; Skrede, I.; Kauserud, H. How many DNA markers are needed to reveal cryptic fungal species?. *Fungal Biol.*, **2015**, *119(10)*, 940-945. https://doi.org/10.1016/j.funbio.2015.07.006
45. Patel, R. S.; Vasava, A. M.; Rajput, K. S. New distribution record of *Cyathus stercoreus* (Schwein.) De Toni (Nidulariaceae) for India from Gujarat state. *Stud. Mycol.*, **2018**, *3(1)*, 227-233. https://doi.org/10.5943/sif/3/1/22
46. Aloi, F.; Giambra, S.; Schena, L.; Surico, G.; Pane, A.; Gusella, G.; Stracquadanio, C.; Burruano, S.; Cacciola, S.O. New insights into scabby canker of *Opuntia ficus-indica*, caused by *Neofusicoccum batangarum*. *Phytopathol. Mediterr.*, **2020**, *59(2)*, 269-284. https://doi.org/10.14601/Phyto-11225
47. Bagi, B.; Nagy, C.; Tóth, A.; Palkovics, L.; Petróczy M. *Plenodomus biglobosus* on oilseed rape in Hungary. *Phytopathol. Mediterr.*, **2020**, *59(2)*, 345-351.
48. Bertetti, D.; Pensa, P.; Matić, S.; Gullino, M.L.; Garibaldi A. Stem rot caused by *Fusarium oxysporum f. sp. opuntiarum* on *Mammillaria painteri* in Italy. *Phytopathol. Mediterr.*, **2020**, *59(2)*, 365-369.
49. Brahmanage, R. S.; Liu, M.; Wanasinghe, D. N.; Dayarathne, M. C.; Mei, L.; Jeewon, R.; Li, X.; Hyde, K. D. *Heterosporicola beijingense* sp. nov. (Leptosphaeriaceae, Pleosporales) associated with *Chenopodium quinoa* leaf spots. *Phytopathol. Mediterr.*, **2020**, *59(2)*, 219-227.
50. Guarnaccia, V.; Martino, I.; Tabone, G.; Brondino, L.; Gullino, M.L.. Fungal pathogens associated with stem blight and dieback of blueberry in northern Italy. *Phytopathol. Mediterr.*, **2020**, *59(2)*, 229-245.
51. Matić, S.; Tabone, G.; Guarnaccia, V.; Gullino, M. L.; Garibaldi, A. Emerging leafy vegetable crop diseases caused by the *Fusarium incarnatum-equiseti* species complex. *Phytopathol. Mediterr.*, **2020**, *59(2)*, 303-317.
52. Pereira, D.R.S.; Phillips, A.J.L. A new leaf spot disease of *Chamaerops humilis* caused by *Palmeiromyces chamaeropicola* gen. et sp. nov.. *Phytopathol. Mediterr.*, **2020**, *59(2)*, 353-363.
53. Réblová, M.; Kolařík, M.; Nekvindová, J.; Réblová, K.; Sklenář, F.; Miller, A. N.; Hernández-Restrepo, M. Phylogenetic reassessment, taxonomy, and biogeography of *Codinaea* and similar fungi. *J. Fungi*, **2021**, *7(12)*, 1097. https://doi.org/10.3390/jof7121097
54. Réblová, M.; Nekvindová, J.; Kolařík, M.; Hernández-Restrepo, M. Delimitation and phylogeny of *Dictyochaeta*, and introduction of *Achrochaeta* and *Tubulicolla*, genera nova. *Mycologia*, **2021**, *113(2)*, 390-433. https://doi.org/10.1080/00275514.2020.1822095
55. Visagie, C. M.; Goodwell, M.; Nkwe, D. O. *Aspergillus* diversity from the Gcwihaba Cave in Botswana and description of one new species. *Fungal Syst. Evol.*, **2021**, *8(1)*, 81-89. https://doi.org/10.3114/fuse.2021.08.07
56. Gouliamova, D. E.; Dimitrov, R. A.; Stoilova-Disheva, M. M. DNA barcoding of yeasts from selected Bulgarian food products. *Biotechnol. Biotechnol. Equip.*, **2012**, *26(sup1)*, 32-34. https://doi.org/10.5504/50YRTIMB.2011.0006
57. Panelli, S.; Buffoni, J. N.; Bonacina, C.; Feligini, M. Identification of moulds from the Taleggio cheese environment by the use of DNA barcodes. *Food Control*, **2012**, *28(2)*, 385-391. https://doi.org/10.1016/j.foodcont.2012.05.022
58. Panelli, S.; Brambati, E.; Bonacina, C.; Feligini, M. Diversity of fungal flora in raw milk from the Italian Alps in relation to pasture altitude. *SpringerPlus*, **2013**, *2(1)*, 1-9. https://doi.org/10.1186/2193-1801-2-405
59. Qing, C. A.; Li-Ping, T. A. N. G.; Zhu-Liang, Y. A. N. G. DNA Barcoding of economically important mushrooms: A case study on lethal amanitas from China. *Plant Divers.*, **2012**, *34(6)*, 614. ISSN: 2095-0845
60. Ainsworth, A.; Cannon, P.; Dentinger, B. DNA barcoding and morphological studies reveal two new species of waxcap mushrooms (Hygrophoraceae) in Britain. *MycoKeys*, **2013**, *7*, 45. http://dx.doi.org/10.3897/mycokeys.7.5860
61. Cunha, S. C.; Faria, M. A.; Pereira, V. L.; Oliveira, T. M.; Lima, A. C.; Pinto, E. Patulin assessment and fungi identification in organic and conventional fruits and derived products. *Food Control*, **2014**, *44*, 185-190. https://doi.org/10.1016/j.foodcont.2014.03.043
62. Khaund, P.; Joshi, S. R. DNA barcoding of wild edible mushrooms consumed by the ethnic tribes of India. *Gene*, **2014**, *550(1)*, 123-130. https://doi.org/10.1016/j.gene.2014.08.027
63. Kasper-Pakosz, R.; Pietras, M.; Łuczaj, Ł. Wild and native plants and mushrooms sold in the open-air markets of south-eastern Poland. *J. Ethnobiol. Ethnomedicine*, **2016**, *12(1)*, 1-17. https://doi.org/10.1186/s13002-016-0117-8
64. Buehler, A. J.; Evanowski, R. L.; Martin, N. H.; Boor, K. J.; Wiedmann, M. Internal transcribed spacer (ITS) sequencing reveals considerable fungal diversity in dairy products. *Int. J. Dairy Sci.*, **2017**, *100(11)*, 8814-8825. https://doi.org/10.3168/jds.2017-12635
65. Carroll, E.; Trinh, T. N.; Son, H.; Lee, Y. W.; Seo, J. A. Comprehensive analysis of fungal diversity and enzyme activity in nuruk, a Korean fermenting starter, for acquiring useful fungi. *J. Microbiol.*, **2017**, *55(5)*, 357-365. https://doi.org/10.1007/s12275-017-7114-z
66. Li, J.; He, X.; Liu, X. B.; Yang, Z. L.; Zhao, Z. W. Species clarification of oyster mushrooms in China and their DNA barcoding. *Mycol. Prog.*, **2017**, *16(3)*, 191-203. https://doi.org/10.1007/s11557-016-1266-9
67. Raja, H. A.; Baker, T. R.; Little, J. G.; Oberlies, N. H. DNA barcoding for identification of consumer-relevant mushrooms: A partial solution for product certification?. *Food Chem.*, **2017**, *214*, 383-392. https://doi.org/10.1016/j.foodchem.2016.07.052
68. Mešić, A.; Šamec, D.; Jadan, M.; Bahun, V.; Tkalčec, Z. Integrated morphological with molecular identification and bioactive compounds of 23 Croatian wild mushrooms samples. *Food Biosci.*, **2020**, *37*, 100720. https://doi.org/10.1016/j.fbio.2020.100720
69. Kakoti, M.; Hazarika, D. J.; Kumar, A.; Barooah, M.; Modi, M. K.; Bhattacharyya, A.; Boro, R. C. Genetic diversity and DNA barcoding of wild mushrooms from Northeast India. *Iran. J. Sci. Technol. Trans. A: Sci.*, **2021**, *45(2)*, 469-479. https://doi.org/10.1007/s40995-021-01067-7

70. Leavitt, S.; Fernández-Mendoza, F.; Pérez-Ortega, S.; Sohrabi, M.; Divakar, P.; Lumbsch, T.; Clair, L. S. DNA barcode identification of lichen-forming fungal species in the *Rhizoplaca melanophthalma* species-complex (Lecanorales, Lecanoraceae), including five new species. *MycoKeys*, **2013**, *7*, 1. https://doi.org/10.3897/mycokeys.7.4508
71. Leavitt, S. D.; Esslinger, T. L.; Hansen, E. S.; Divakar, P. K.; Crespo, A.; Loomis, B. F.; Lumbsch, H. T. DNA barcoding of brown *Parmeliae* (Parmeliaceae) species: a molecular approach for accurate specimen identification, emphasizing species in Greenland. *Org. Divers. Evol.*, **2014**, *14(1)*, 11-20. 10.1007/s13127-013-0147-1
72. Parnmen, S.; Leavitt, S. D.; Rangsiruji, A.; Lumbsch, H. T. Identification of species in the *Cladia aggregata* group using DNA barcoding (Ascomycota: Lecanorales). *Phytotaxa*, **2013**, *115(1)*, 1-14. https://doi.org/10.11646/phytotaxa.115.1.1
73. Pino-Bodas, R.; Martin, M. P.; Burgaz, A. R.; Lumbsch, H. T. Species delimitation in *Cladonia* (Ascomycota): a challenge to the DNA barcoding philosophy. *Mol. Ecol. Res.*, **2013**, *13(6)*, 1058-1068. https://doi.org/10.1111/1755-0998.12086
74. Kanz, B.; von Brackel, W.; Cezanne, R.; Eichler, M.; Hohmann, M. L.; Teuber, D.; Printzen, C. DNA barcodes for the distinction of reindeer lichens: a case study using *Cladonia rangiferina* and *C. stygia*. Herzogia, **2015**, *28(2)*, 445-464. https://doi.org/10.13158/heia.28.2.2015.445
75. Divakar, P. K., Leavitt, S. D.; Molina, M. C.; Del-Prado, R.; Lumbsch, H. T.; Crespo, A. A DNA barcoding approach for identification of hidden diversity in Parmeliaceae (Ascomycota): *Parmelia sensu stricto* as a case study. *Bot. J. Linn. Soc.*, **2016**, *180(1)*, 21-29. https://doi.org/10.1111/boj.12358
76. Lücking, R.; Nadel, M. R. A.; Araujo, E.; Gerlach, A. Two decades of DNA barcoding in the genus *Usnea* (Parmeliaceae): how useful and reliable is the ITS?. *Plant Fungal Syst.*, **2020**, *65(2)*, 303-357. https://doi.org/10.35535/pfsyst-2020-0025
77. Dal Forno, M.; Lawrey, J. D.; Moncada, B.; Bungartz, F.; Grube, M.; Schuettpelz, E.; Lücking, R. DNA barcoding of fresh and historical collections of lichen-forming Basidiomycetes in the genera *Cora* and *Corella* (Agaricales: Hygrophoraceae): a success story?. *Diversity*, **2022**, *14(4)*, 284. https://doi.org/10.3390/d14040284
78. Li, Y.; Jiao, L.; Yao, Y. J. Non-concerted ITS evolution in fungi, as revealed from the important medicinal fungus *Ophiocordyceps sinensis*. *Mol. Phylogenetics Evol.*, **2013**, *68(2)*, 373-379. https://doi.org/10.3390/d14040284
79. Xiang, L.; Song, J.,; Xin, T.; Zhu, Y.; Shi, L.; Xu, X.; Pang, X.; Yao, H.; Li, W.; Chen, S. DNA barcoding the commercial Chinese caterpillar fungus. *FEMS Microbiol. Lett.*, 2013, 347(2), 156-162. https://doi.org/10.1111/1574-6968.12233
80. Yap, H. Y. Y.; Shin-Yee, F.; Tan, N. H.; Ng, S. T.; Tan, C. S. DNA barcode markers for two new species of tiger milk mushroom: *Lignosus tigris* and *L. cameronensis*. *Int. J. Agric. Biol. Eng.*, **2014**, *16(4)*. ISSN Print: 1560–8530
81. Mukhin, V. A.; Zhuykova, E. V.; Badalyan, S. M. Genetic variability of the medicinal tinder bracket polypore, *Fomes fomentarius* (Agaricomycetes), from the Asian part of Russia. *Int. J. Med. Mushrooms*, **2018**, *20(6)*. https://doi.org/10.1615/IntJMedMushrooms.2018026278
82. Singh, P.; Raghukumar, C.; Meena, R. M.; Verma, P.; Shouche, Y. Fungal diversity in deep-sea sediments revealed by culture-dependent and culture-independent approaches. *Fungal Ecol.*, **2012**, *5(5)*, 543-553. https://doi.org/10.1016/j.funeco.2012.01.001
83. Xu, M.; Heidmarsson, S.; Thorsteinsdottir, M.; Eiriksson, F. F.; Omarsdottir, S.; Olafsdottir, E. S. DNA barcoding and LC-MS metabolite profiling of the lichen-forming genus *Melanelia*: Specimen identification and discrimination focusing on Icelandic taxa. *PloS One*, **2017**, *12(5)*, e0178012. https://doi.org/10.1371/journal.pone.0178012
84. Fotedar, R.; Kolecka, A.; Boekhout, T.; Fell, J. W.; Al-Malki, A.; Zeyara, A.; Al Marri, M. Fungal diversity of the hypersaline Inland Sea in Qatar. *Botanica Marina*, **2018**, *61(6)*, 595-609. https://doi.org/10.1515/bot-2018-0048
85. Yu, N. H.; Park, S. Y.; Kim, J. A.; Park, C. H.; Jeong, M. H.; Oh, S. O.; Hong, S.G.; Talavera, M.; Divakar, P.K.;Hur, J. S. Endophytic and endolichenic fungal diversity in maritime Antarctica based on cultured material and their evolutionary position among Dikarya. *Fungal Syst. Evol.*, **2018**, *2(1)*, 263-272. https://doi.org/10.3114/fuse.2018.02.07
86. Andreakis, N.; Høj, L.; Kearns, P.; Hall, M. R.; Ericson, G.; Cobb, R. E.; Gordon, B. R.; Evans-Illidge, E. Diversity of marine-derived fungal cultures exposed by DNA barcodes: the algorithm matters. *PloS One*, **2015**, *10(8)*, e0136130. https://doi.org/10.1371/journal.pone.0136130
87. Ding, M. Y.; Chen, W.; Ma, X. C.; Lv, B. W.; Jiang, S. Q.; Yu, Y. N.; Rahimi, M.J.; Gao, R.-W.; Zhao, Z.; Cai, F.; Druzhinina, I. S. Emerging salt marshes as a source of *Trichoderma arenarium* sp. nov. and other fungal bioeffectors for biosaline agriculture. *J. App. Microbiol.*, **2021**, *130(1)*, 179-195. https://doi.org/10.1111/jam.14751
88. 88.Taha, H.; Shivanand, P.; Zainudin, M. A. A.; Hadanan, N. A. Identification of culturable marine fungi and bacteria from coastal region in Brunei Darussalam: Identification of culturable marine microbes. *Biodiversitas*, **2021**, *22(3)*. https://doi.org/10.13057/biodiv/d220332
89. Duarte, S.; Batista, D.; Bärlocher, F.; Cássio, F.; Pascoal, C. Some new DNA barcodes of aquatic hyphomycete species. *Mycoscience*, **2015**, *56(1)*, 102-108. https://doi.org/10.1016/j.myc.2014.04.002
90. Hamad, I. S.; Arif, H. H.; Amin, H. S. M.; Khidir, K. A. Exploring fungal diversity and distribution in Dukan freshwater lake using ITS rDNA-based PCR cloning/sequencing. JZS-A, **2017**, *19(3-4)*, 1-8.
91. López-Quintero, C. A.; Atanasova, L.; Franco-Molano, A. E.; Gams, W.; Komon-Zelazowska, M.; Theelen, B.; Müller, W. H.; Boekhout, T.; Druzhinina, I. DNA barcoding survey of Trichoderma diversity in soil and litter of the Colombian lowland Amazonian rainforest reveals *Trichoderma strigosellum* sp. nov. and other species. *Antonie van Leeuwenhoek*, **2013**, *104(5)*, 657-674. https://doi.org/10.1007/s10482-013-9975-4 https://doi.org/10.1007/s10482-013-9975-4
92. Teasdale, S. E.; Beulke, A. K.; Guy, P. L.; Orlovich, D. A. Environmental barcoding of the ectomycorrhizal fungal genus *Cortinarius*. *Fungal Divers.*, **2013**, *58(1)*, 299-310. https://doi.org/10.1007/s13225-012-0218-1
93. Garnica, S.; Schön, M. E.; Abarenkov, K.; Riess, K.; Liimatainen, K.; Niskanen, T.; Dima, B.; Soop, K.; Frøslev, T. G.; Jeppesen, T. S.; Peintner, U.; Kuhnert-Finkernagel, R.; Brandrud, T. E.; Saar, G.; Oertel, B.;Ammirati, J. F. Determining threshold values for

barcoding fungi: lessons from *Cortinarius* (Basidiomycota), a highly diverse and widespread ectomycorrhizal genus. *FEMS Microbiol. Ecol.*, **2016**, *92(4)*, fiw045. https://doi.org/10.1093/femsec/fiw045
94. Li, G. J.; Zhao, R. L.; Zhang, C. L.; Lin, F. C. A preliminary DNA barcode selection for the genus *Russula* (Russulales, Basidiomycota). *Mycol.*, **2019**, *10(2)*, 61-74. https://doi.org/10.1080/21501203.2018.1500400
95. Martins, M. R.; Santos, C.; Soares, C.; Santos, C.; Lima, N. *Gongronella eborensis* sp. nov., from vineyard soil of Alentejo (Portugal). *Int. J. Syst. Evol. Microbiol.*, **2020**, *70(5)*, 3475-3482. https://doi.org/10.1099/ijsem.0.004201
96. Yang, X. Q.; Feng, M. Y.; Yu, Z. F. *Exophiala pseudooligosperma* sp. nov., a novel black yeast from soil in southern China. *Int. J. Syst. Evol. Microbiol.*, **2021**, *71(11)*, 005116. https://doi.org/10.1099/ijsem.0.005116
97. Waing, K. G. D.; Gutierrez, J. M.; Galvez, C. T.; Undan, J. R. Molecular identification of leaf litter fungi potential for cellulose degradation. *Mycosphere*, **2015**, *6(2)*, 139-144. https://doi.org/10.5943/mycosphere/6/2/3
98. Fahmi, A. I.; Eissa, R. A.; El-Halfawi, K. A.; Hamza, H. A.; Helwa, M. S. Identification of *Trichoderma* spp. by DNA barcode and screening for cellulolytic activity. *J. Microb. Biochem. Technol.*, **2016**, *8(3)*, 202-209. https://doi.org/10.4172/1948-5948.1000286
99. M'barek, H. N.; Taidi, B.; Smaoui, T.; Aziz, M. B.; Mansouri, A.; Hajjaj, H. Isolation, screening and identification of ligno-cellulolytic fungi from northern central Morocco. *Biotechnol. Agron. Soc. Environ.*, **2019**, *23(4)*, 207-217. https://doi.org/10.25518/1780-4507.18182
100. Ben Ali, W.; Chaduli, D.; Navarro, D.; Lechat, C.; Turbé-Doan, A.; Bertrand, E.; ;Mechichi, T. Screening of five marine-derived fungal strains for their potential to produce oxidases with laccase activities suitable for biotechnological applications. *BMC Biotechnol.*, **2020**, *20(1)*, 1-13. https://doi.org/10.1186/s12896-020-00617-y
101. Páramo-Aguilera, L.; Ortega-Morales, B. O.; Narváez-Zapata, J. A. Culturable fungi associated with urban stone surfaces in Mexico City. *Electron. J. Biotechnol.*, **2012**, *15(4)*, 4-4. https://doi.org/10.2225/vol15-issue4-fulltext-6
102. Savković, Ž.; Unković, N.; Stupar, M.; Franković, M.; Jovanović, M.; Erić, S.; Šarić, K.; Stankovićalvic, S.; Dimkića, I; Vukojevića, J.; Grbić, M. L. Diversity and biodeteriorative potential of fungal dwellers on ancient stone stela. *Int. Biodeterior. Biodegradation*, **2016**, *115*, 212-223. https://doi.org/10.1016/j.ibiod.2016.08.027
103. Stoyancheva, G.; Krumova, E.; Kostadinova, N.; Miteva-Staleva, J.; Grozdanov, P.; Ghaly, M. F.; Sakr, A. A.; Angelova, M. Biodiversity of contaminant fungi at different coloured materials in ancient Egypt tombs and mosques. *C. R. Acad. Bulg. Sci.*, **2018**, *71(7)*. https://doi.org/10.7546/CRABS.2018.07.06
104. Ponizovskaya, V. B.; Rebrikova, N. L.; Kachalkin, A. V.; Antropova, A. B.; Bilanenko, E. N.; Mokeeva, V. L. Micromycetes as colonizers of mineral building materials in historic monuments and museums. *Fungal Biol.*, **2019**, *123(4)*, 290-306. https://doi.org/10.1016/j.funbio.2019.01.002
105. Gámez-Espinosa, E.; Bellotti, N.; Deyá, C.; Cabello, M. Mycological studies as a tool to improve the control of building materials biodeterioration. *J. Build. Eng.*, **2020**, *32*, 101738. https://doi.org/10.1016/j.jobe.2020.101738
106. Sun, W.; Su, L.,; Yang, S.; Sun, J.; Liu, B.; Fu, R., Wu, B.; Liu, X.; Cai, L.; Guo, L.; Xiang, M. Unveiling the hidden diversity of rock-inhabiting fungi: Chaetothyriales from China. *J. Fungi*, **2020**, *6(4)*, 187. https://doi.org/10.3390/jof6040187
107. Avchar, R.; Groenewald, M.; Baghela, A. *Wickerhamiella shivajii* sp. nov., a thermotolerant yeast isolated from distillery effluent. *Int. J. Syst. Evol. Microbiol.*, **2019**, *69(10)*, 3262-3267. https://doi.org/10.1099/ijsem.0.003616
108. Ezeonuegbu, B. A.; Abdullahi, M. D.; Whong, C. M.; Sohunago, J. W.; Kassem, H. S.; Yaro, C. A.; Hetta, H. F.; Mostafa-Hedeab, G.; Zouganelis, G. D.; Batiha, G. E. S. Characterization and phylogeny of fungi isolated from industrial wastewater using multiple genes. *Sci. Rep.*, **2022**, *12(1)*, 1-11. https://doi.org/10.1038/s41598-022-05820-9
109. 109.Sanyal, O.; Shinde, V. L.; Meena, R. M.; Damare, S.; Shenoy, B. D. The ITS-based phylogeny of fungi associated with tarballs. *Mar. Pollut. Bull.*, **2016**, *113(1-2)*, 277-281. https://doi.org/10.1016/j.marpolbul.2016.09.052
110. Mohammadian, E.; Arzanlou, M.; Babai-Ahari, A. Diversity of culturable fungi inhabiting petroleum-contaminated soils in Southern Iran. Antonie Van Leeuwenhoek, **2017**, *110(7)*, 903-923. https://doi.org/10.1007/s10482-017-0863-1
111. Dou, K.; Lu, Z.; Wu, Q.; Ni, M.; Yu, C.; Wang, M.; Xie, H.; Chen, J.; Zhang, C. MIST: a multilocus identification system for *Trichoderma*. *App. Environ. Microbiol.*, **2020**, *86(18)*, e01532-20. https://doi.org/10.1128/AEM.01532-20
112. Taha, H.; Shivanand, P.; Khoo, D. H.; Mohammad, Y. H.; Matussin, N. B. A.; Metali, F. Identification of culturable petroleum-degrading bacteria and fungi from petroleum-contaminated sites in Brunei Darussalam. *J. Environ. Sci. Health, Part A*, **2020**, *55(13)*, 1542-1547. https://doi.org/10.1080/10934529.2020.1826238
113. Ramdass, A. C.; Rampersad, S. N. Diversity and oil degradation potential of culturable microbes isolated from chronically contaminated soils in Trinidad. *Microorganisms*, **2021**, *9(6)*, 1167. https://doi.org/10.3390/microorganisms9061167

References: Supplementary Table S9

1. Hamad, I.; Abou Abdallah, R.; Ravaux, I.; Mokhtari, S.; Tissot-Dupont, H.; Michelle, C.; Stein, A.; Lagier, J.-C.; Raoult, D.; Bittar, F. Metabarcoding analysis of eukaryotic microbiota in the gut of HIV-infected patients. *PLoS One*, **2018**, *13(1)*, e0191913. https://doi.org/10.1371/journal.pone.0191913
2. Thongsripong, P.; Chandler, J. A.; Green, A. B.; Kittayapong, P.; Wilcox, B. A.; Kapan, D. D.; Bennett, S. N. Mosquito vector-associated microbiota: metabarcoding bacteria and eukaryotic symbionts across habitat types in Thailand endemic for dengue and other arthropod-borne diseases. *Ecol. Evol.*, **2018**, *8(2)*, 1352-1368. https://doi.org/10.1002/ece3.3676
3. Durmaz, R.; Durmaz, B.; Arı, O.; Abdulmajed, O.; Çelik, S.; Kalcıoğlu, M. T. Mycobiome in the middle ear cavity with and without otitis media with effusion. *Turk. Arch. Otorhinolaryngol.*, **2021**, *59(4)*, 261. https://doi.org/10.4274/tao.2021.2021-10-4
4. Willis, J. R.; Iraola-Guzmán, S.; Saus, E.; Ksiezopolska, E.; Cozzuto, L.; Bejarano, L. A.; Andreu-Somavilla, N.; Alloza-Trabado, M.; Puig-Sola, A.; Blanco, A.; Broglio, E.; Carolis, C.; Hechta, J.; Ponomarenko, J.; Gabaldón, T. Oral microbiome in down

syndrome and its implications on oral health. *J. Oral Microbiol.*, **2021**, *13(1)*, 1865690. https://doi.org/10.1080/20002297.2020.1865690
5. Willis, J. R.; Saus, E.; Iraola-Guzmán, S.; Cabello-Yeves, E.; Ksiezopolska, E.; Cozzuto, L.; Bejarano, L. A.; Andreu-Somavilla, N.; Alloza-Trabado, M.; Blanco, A.; Puig-Sola, A.; Broglio, E; Carolis, C.; Ponomarenko, J.; Hecht, J.; Gabaldón, T. Citizen-science based study of the oral microbiome in cystic fibrosis and matched controls reveals major differences in diversity and abundance of bacterial and fungal species. *J. Oral Microbiol.*, **2021**, *13(1)*, 1897328. https://doi.org/10.1080/20002297.2021.1897328
6. Speicher, D.J.; Aziz, R.K. Profiling the human oral mycobiome in tissue and saliva using ITS2 DNA metabarcoding compared to a fungal-specific database. In: Adami, G.R. (eds) The oral microbiome. methods in molecular biology, vol 2327. Humana, New York, **2021**, 253–269. https://doi.org/10.1007/978-1-0716-1518-8_15
7. Irinyi, L.; Roper, M.; Meyer, W. In depth search of the Sequence Read Archive database reveals global distribution of the emerging pathogenic fungus *Scedosporium aurantiacum*. *Med. Mycol.*, **2022**, *60(4)*, myac019. https://doi.org/10.1093/mmy/myac019
8. Willis, J. R.; Saus, E.; Iraola-Guzmán, S.; Ksiezopolska, E.; Cozzuto, L.; Bejarano, L. A.; Andreu-Somavilla, N.; Alloza-Trabado, M.; Blanco, A.; Puig-Sola, A.; Broglio, E.; Carolis, C.; Ponomarenko, J.; Hecht, J.; Gabaldón, T. Citizen-science reveals changes in the oral microbiome in Spain through age and lifestyle factors. *NPJ Biofilms Microbiomes*, 2022, 8(1), 1-11. https://doi.org/10.1007/978-1-0716-1518-8_15
9. You, L.; Simmons, D. R.; Bateman, C. C.; Short, D. P.; Kasson, M. T.; Rabaglia, R. J.; Hulcr, J. New fungus-insect symbiosis: culturing, molecular, and histological methods determine saprophytic Polyporales mutualists of *Ambrosiodmus ambrosia* beetles. *PLoS One*, **2015**, *10(9)*, e0137689. https://doi.org/10.1371/journal.pone.0137689
10. Miller, K. E.; Hopkins, K.; Inward, D. J.; Vogler, A. P. Metabarcoding of fungal communities associated with bark beetles. *Ecol. Evol.*, **2016**, *6(6)*, 1590-1600. https://doi.org/10.1002/ece3.1925
11. Malacrinò, A.; Schena, L.; Campolo, O.; Laudani, F.; Mosca, S.; Giunti, G.; Strano, C. P.; Palmeri, V. A metabarcoding survey on the fungal microbiota associated to the olive fruit fly. *Microb. Ecol.*, **2017**, *73(3)*, 677-684. https://doi.org/10.1007/s00248-016-0864-z
12. Brentassi, M. E.; Medina, R.; de la Fuente, D.; Franco, M. E.; Toledo, A. V.; Saparrat, M. C.; Balatti, P. A. Endomycobiome associated with females of the planthopper *Delphacodes kuscheli* (Hemiptera: Delphacidae): a metabarcoding approach. *Heliyon*, **2020**, *6(8)*, e04634. https://doi.org/10.1016/j.heliyon.2020.e04634
13. Poissant, J.; Gavriliuc, S.; Bellaw, J.; Redman, E. M.; Avramenko, R. W.; Robinson, D.; Shury, M. L. K.; Jenkins, E. J.; McLoughlin, P. D.; Nielsen, M. K.; Gilleard, J. S. A repeatable and quantitative DNA metabarcoding assay to characterize mixed strongyle infections in horses. *Int. J. Parasitol.*, **2021**, *51(2-3)*, 183-192. https://doi.org/10.1016/j.ijpara.2020.09.003
14. Bradshaw, A. J.; Autumn, K. C.; Rickart, E. A.; Dentinger, B. T. On the origin of feces: Fungal diversity, distribution, and conservation implications from feces of small mammals. *Environ. DNA*, **2022**, *4(3)*, 608-626. https://doi.org/10.1002/edn3.281
15. Tawidian, P.; Coon, K. L.; Jumpponen, A.; Cohnstaedt, L. W.; Michel, K. Host-environment interplay shapes fungal diversity in mosquitoes. *Msphere*, **2021**, *6(5)*, e00646-21. https://doi.org/10.1128/mSphere.00646-21
16. Ceballos-Escalera, A.; Richards, J.; Arias, M. B.; Inward, D. J.; Vogler, A. P. Metabarcoding of insect-associated fungal communities: a comparison of internal transcribed spacer (ITS) and large-subunit (LSU) rRNA markers. *MycoKeys, 2022*, *88*, 1. https://doi.org/10.3897/mycokeys.88.77106
17. Abdelfattah, A.; Li Destri Nicosia, M. G.; Cacciola, S. O.; Droby, S.; Schena, L. Metabarcoding analysis of fungal diversity in the phyllosphere and carposphere of olive (*Olea europaea*). *PLoS One*, **2015**, *10(7)*, e0131069. https://doi.org/10.1371/journal.pone.0131069
18. Jakuschkin, B.; Fievet, V.; Schwaller, L.; Fort, T.; Robin, C.; Vacher, C. Deciphering the pathobiome: intra-and interkingdom interactions involving the pathogen *Erysiphe alphitoides*. *Microb. Ecol.*, **2016**, *72(4)*, 870-880. https://doi.org/10.1007/s00248-016-0777-x
19. Morales-Cruz, A.; Figueroa-Balderas, R.; García, J. F.; Tran, E.; Rolshausen, P. E.; Baumgartner, K.; Cantu, D. Profiling grapevine trunk pathogens in planta: a case for community-targeted DNA metabarcoding. *BMC Microbiol.*, **2018**, *18(1)*, 1-14. https://doi.org/10.1186/s12866-018-1343-0
20. 20.Vanga, B. R.; Panda, P.; Shah, A. S.; Thompson, S.; Woolley, R. H.; Ridgway, H. J.; Mundy; D. C.; Bulman, S. DNA metabarcoding reveals high relative abundance of trunk disease fungi in grapevines from Marlborough, New Zealand. *BMC Microbiol.*, **2022**, *22(1)*, 1-11. https://doi.org/10.1186/s12866-022-02520-2
21. Schön, M. E.; Nieselt, K.; Garnica, S. Belowground fungal community diversity and composition associated with Norway spruce along an altitudinal gradient. *PloS One*, **2018**, *13(12)*, e0208493. https://doi.org/10.1371/journal.pone.0208493
22. Boutigny, A. L.; Gautier, A.; Basler, R.; Dauthieux, F.; Leite, S.; Valade, R.; Aguayo, J.; Ioos, R.; Laval, V. Metabarcoding targeting the EF1 alpha region to assess *Fusarium* diversity on cereals. *PloS One*, **2019**, *14(1)*, e0207988. https://doi.org/10.1371/journal.pone.0207988
23. Chi, W. C.; Chen, W.; He, C. C.; Guo, S. Y.; Cha, H. J.; Tsang, L. M.; Ho, T. W.; Pang, K. L. A highly diverse fungal community associated with leaves of the mangrove plant *Acanthus ilicifolius* var. *xiamenensis* revealed by isolation and metabarcoding analyses. *PeerJ*, **2019**, 7, e7293. https://doi.org/10.7717/peerj.7293
24. Cobo-Díaz, J. F.; Baroncelli, R.; Le Floch, G.; Picot, A. Combined metabarcoding and co-occurrence network analysis to profile the bacterial, fungal and *Fusarium* communities and their interactions in maize stalks. *Front. Microbiol.*, **2019**, *10*, 261. https://doi.org/10.3389/fmicb.2019.00261
25. Knorr, K.; Jørgensen, L. N.; Nicolaisen, M. Fungicides have complex effects on the wheat phyllosphere mycobiome. *PloS One*, **2019**, *14(3)*, e0213176. https://doi.org/10.1371/journal.pone.0213176

26. Ruiz Gómez, F. J.; Navarro-Cerrillo, R. M.; Pérez-de-Luque, A.; Oβwald, W.; Vannini, A.; Morales-Rodríguez, C. Assessment of functional and structural changes of soil fungal and oomycete communities in holm oak declined dehesas through metabarcoding analysis. *Sci. Rep.*, **2019**, *9(1)*, 1-16. https://doi.org/10.1038/s41598-019-41804-y
27. Sapp, M.; Tyborski, N.; Linstädter, A.; Lopez Sanchez, A.; Mansfeldt, T.; Waldhoff, G.; Bareth, G.; Bonkowski, M.; Rose, L. E. Site-specific distribution of oak rhizosphere-associated oomycetes revealed by cytochrome c oxidase subunit II metabarcoding. *Ecol. Evol.*, **2019**, *9(18)*, 10567-10581. https://doi.org/10.1002/ece3.5577
28. Schiro, G.; Colangeli, P.; Müller, M. E. A metabarcoding analysis of the mycobiome of wheat ears across a topographically heterogeneous field. *Front. Microbiol.*, **2019**, *2095*. https://doi.org/10.3389/fmicb.2019.02095
29. Laurent, B.; Marchand, M.; Chancerel, E.; Saint-Jean, G.; Capdevielle, X.; Poeydebat, C.; Bellée, A.; Comont, G.; Villate, L.;Desprez-Loustau, M. L. A richer community of Botryosphaeriaceae within a less diverse community of fungal endophytes in grapevines than in adjacent forest trees revealed by a mixed metabarcoding strategy. *Phytobiomes J.*, **2020**, *4(3)*, 252-267. https://doi.org/10.1094/PBIOMES-01-20-0004-R
30. Wemheuer, F.; Berkelmann, D.; Wemheuer, B.; Daniel, R.; Vidal, S.; Bisseleua Daghela, H. B. Agroforestry management systems drive the composition, diversity, and function of fungal and bacterial endophyte communities in *Theobroma cacao* leaves. *Microorganisms*, **2020**, *8(3)*, 405. https://doi.org/10.3390/microorganisms8030405
31. Costa, D.; Fernandes, T.; Martins, F.; Pereira, J. A.; Tavares, R. M.; Santos, P. M.; Baptista, P.; Lino-Neto, T. Illuminating *Olea europaea* l. endophyte fungal community. *Microbiol. Res.*, **2021**, *245*, 126693. https://doi.org/10.1016/j.micres.2020.126693
32. Durán, M.; San Emeterio, L.; Canals, R. M. Comparison of culturing and metabarcoding methods to describe the fungal endophytic assemblage of *Brachypodium rupestre* growing in a range of anthropized disturbance regimes. *Biology*, **2021**, *10(12)*, 1246. https://doi.org/10.3390/biology10121246
33. Migliorini, D.; Messal, M.; Santini, A.; Ramos, A. P.; Talhinhas, P.; Wingfield, M. J.; Burgess, T. Metabarcoding reveals southern hemisphere fungal endophytes within wood of cultivated Proteaceae in Portugal. *Eur. J. Plant Pathol.*, **2021**, *160(1)*, 173-184. https://doi.org/10.1007/s10658-021-02233-8
34. Milazzo, C.; Zulak, K. G.; Muria-Gonzalez, M. J.; Jones, D.; Power, M.; Bransgrove, K.; Bunce, M.; Lopez-Ruiz, F. J. High-throughput metabarcoding characterizes fungal endophyte diversity in the phyllosphere of a barley crop. *Phytobiomes J.*, **2021**, *5(3)*, 316-325. https://doi.org/10.1094/PBIOMES-09-20-0066-R
35. Piombo, E.; Abdelfattah, A.; Droby, S.; Wisniewski, M.; Spadaro, D.; Schena, L. Metagenomics approaches for the detection and surveillance of emerging and recurrent plant pathogens. *Microorganisms*, **2021**, *9(1)*, 188. https://doi.org/10.3390/microorganisms9010188
36. Runnel, K.; Drenkhan, R.; Adamson, K.; Lõhmus, P.; Rosenvald, K.; Rosenvald, R.; Rosenvald, R.; Rähn, E.; Tedersoo, L. The factors and scales shaping fungal assemblages in fallen spruce trunks: a DNA metabarcoding study. *For. Ecol. Manage.*, **2021**, *495*, 119381. https://doi.org/10.1016/j.foreco.2021.119381
37. Chen, W.; Radford, D.; Hambleton, S. Towards improved detection and identification of rust fungal pathogens in environmental samples using a metabarcoding approach. *Phytopathology*, **2022**, *112(3)*, 535-548. https://doi.org/10.1094/PHYTO-01-21-0020-R
38. Guo, M.; Yu, J.; Dao, Y.; Jiang, W.; Pang, X. DNA metabarcoding of fungal communities in Heshouwu (*Polygonum multiflorum* Thunb.). *Food Control*, **2022**, *136*, 108850. https://doi.org/10.1016/j.foodcont.2022.108850
39. Perotto, S.; Daghino, S.; Martino, E.; Voyron, S. Metabarcoding reveals diverse endophytic fungal communities in *Vaccinium myrtillus* plant organs and suggests systemic distribution of some ericoid mycorrhizal and DSE fungi. *ResearchSquare*, **2022**
40. Triolet, M.; Edel-Hermann, V.; Gautheron, N.; Mondy, S.; Reibel, C.; André, O.; Guillemin, J.-P.; Steinberg, C. Weeds harbor an impressive diversity of fungi, which offers possibilities for biocontrol. *App. Environ. Microbiol.*, **2022**, aem-02177. https://doi.org/10.1128/aem.02177-21
41. Prigigallo, M. I.; Abdelfattah, A.; Cacciola, S. O.; Faedda, R.; Sanzani, S. M.; Cooke, D. E.; Schena, L. Metabarcoding analysis of *Phytophthora* diversity using genus-specific primers and 454 pyrosequencing. *Phytopathology*, **2016**, *106(3)*, 305-313. https://doi.org/10.1128/aem.02177-21
42. La Spada, F.; Cock, P. J.; Randall, E.; Pane, A.; Cooke, D. E.; Cacciola, S. O. DNA Metabarcoding and isolation by baiting complement each other in revealing phytophthora diversity in anthropized and natural ecosystems. *J. Fungi*, **2022**, *8(4)*, 330. https://doi.org/10.3390/jof8040330
43. Arroyo-López, F. N., Medina, E.; Ruiz-Bellido, M. Á.; Romero-Gil, V.; Montes-Borrego, M.; Landa, B. B. Enhancement of the knowledge on fungal communities in directly brined Aloreña de Málaga green olive fermentations by metabarcoding analysis. *PLoS One*, **2016**, *11(9)*, e0163135. https://doi.org/10.1371/journal.pone.0163135
44. Sternes, P. R.; Lee, D.; Kutyna, D. R.; Borneman, A. R. A combined meta-barcoding and shotgun metagenomic analysis of spontaneous wine fermentation. *Gigascience*, **2017**, *6(7)*, gix040. https://doi.org/10.1093/gigascience/gix040
45. Yu, S. M.; Yu, G. M. Study of the microbiological composition of dairy products and mayonnaise using DNA barcoding and metabarcoding. *Foods Raw Mater.*, **2018**, *6(1)*, 144-153. https://doi.org/10.21603/2308-4057-2018-1-144-153
46. Sha, S. P.; Suryavanshi, M. V.; Tamang, J. P. Mycobiome diversity in traditionally prepared starters for alcoholic beverages in India by high-throughput sequencing method. *Front. Microbiol.*, **2019**, *10*, 348. https://doi.org/10.3389/fmicb.2019.00348
47. Zhao, M.; Su, X. Q.; Nian, B.; Chen, L. J.; Zhang, D. L.; Duan, S. M.; Wang, L. Y.; Shi, X. Y.; Jiang, B.; Jiang, W. W.; Lv, C. Y.; Wang, P.; Shi, Y.; Xiao, Y.; Wu, J.-L.; Pan, Y. H.; Ma, Y. Integrated meta-omics approaches to understand the microbiome of spontaneous fermentation of traditional Chinese pu-erh tea. *Msystems*, **2019**, *4(6)*, e00680-19. https://doi.org/10.1128/mSystems.00680-19

48. Balzan, S.; Carraro, L.; Merlanti, R.; Lucatello, L.; Capolongo, F.; Fontana, F.; Novelli, E.; Larini, I.; Vitulo, N.; Cardazzo, B. Microbial metabarcoding highlights different bacterial and fungal populations in honey samples from local beekeepers and market in north-eastern Italy. *Int. J. Food Microbiol.*, **2020**, *334*, 108806. https://doi.org/10.1016/j.ijfoodmicro.2020.108806
49. Igor, K.; Tatiana, G.; Olga, G.; Philipp, G. Fungal microbiome of barley grain revealed by NGS and mycological analysis. *Foods Raw Mater.*, **2020**, *8(2)*, 286-297. https://doi.org/10.21603/2308-4057-2020-2-286-297
50. Jiang, W.; Guo, M.; Yang, M.; Mantri, N.; Chen, X.; Pang, X. High-throughput analysis of fungal communities in Myristicae Semen. *LWT*, **2020**, *128*, 109499. https://doi.org/10.1016/j.lwt.2020.109499
51. Luo, Q.; Zhu, Y.; Zhang, Z.; Cao, Y.; Zhang, W. Variations in fungal community and diversity in Doushen with different flavors. *Front. Microbiol.*, **2020**, *11*, 447. https://doi.org/10.3389/fmicb.2020.00447
52. von Gastrow, L.; Madec, M. N.; Chuat, V.; Lubac, S.; Morinière, C.; Lê, S.; Santoni, S; Sicard, D.; Valence, F. Microbial diversity associated with gwell, a traditional french mesophilic fermented milk inoculated with a natural starter. *Microorganisms*, **2020**, *8(7)*, 982. https://doi.org/10.3390/microorganisms8070982
53. Abekhti, A.; Taminiau, B.; Nezer, C.; Hiligsman, S.; Mabrouk, K.; Daube, G. Metabarcoding analysis and fermentation performance of the dominant fungal microbiota associated with the Algerian traditional date product "Btana". *Int. Microbiol.*, **2021**, *24(3)*, 351-361. https://doi.org/10.1007/s10123-021-00166-0
54. Bösch, Y.; Britt, E.; Perren, S.; Naef, A.; Frey, J. E.; Bühlmann, A. Dynamics of the apple fruit microbiome after harvest and implications for fruit quality. *Microorganisms*, **2021**, *9(2)*, 272. https://doi.org/10.3390/microorganisms9020272
55. Coton, M.; Deniel, F.; Mounier, J.; Joubrel, R.; Robieu, E.; Pawtowski, A.; Jeuge, S.; Taminiau, B.; Daube, G.; Coton, E.; Frémaux, B. Microbial ecology of french dry fermented sausages and mycotoxin risk evaluation during storage. *Front. Microbiol.*, **2021**, *12*. https://doi.org/10.3389/fmicb.2021.737140
56. Cutler II, W. D.; Bradshaw, A. J.; Dentinger, B. T. What's for dinner this time?: DNA authentication of "wild mushrooms" in food products sold in the USA. *PeerJ*, **2021**, *9*, e11747. https://doi.org/10.7717/peerj.11747
57. Degenhardt, R.; Sobral Marques Souza, D.; Acordi Menezes, L. A.; de Melo Pereira, G. V.; Rodríguez-Lázaro, D.; Fongaro, G.; De Dea Lindner, J. Detection of enteric viruses and core microbiome analysis in artisanal colonial salami-type dry-fermented sausages from Santa Catarina, Brazil. *Foods*, **2021**, *10(8)*, 1957. https://doi.org/10.3390/foods10081957
58. Harrison, K.; Curtin, C. Microbial composition of SCOBY starter cultures used by commercial kombucha brewers in North America. *Microorganisms*, **2021**, *9(5)*, 1060. https://doi.org/10.3390/microorganisms9051060
59. Kaashyap, M.; Cohen, M.; Mantri, N. Microbial Diversity and characteristics of kombucha as revealed by metagenomic and physicochemical analysis. *Nutrients*, **2021**, *13(12)*, 4446. https://doi.org/10.3390/nu13124446
60. Inacio, L. D. J.; Merlanti, R.; Lucatello, L.; Bisutti, V.; Carraro, L.; Larini, I.; Vitulo, N.; Cardazzo, B.; Capolongo, F. Natural contaminants in bee pollen: DNA metabarcoding as a tool to identify floral sources of pyrrolizidine alkaloids and fungal diversity. *Food Res. Int.*, **2021**, *146*, 110438. https://doi.org/10.1016/j.foodres.2021.110438
61. Michailidou, S.; Pavlou, E.; Pasentsis, K.; Rhoades, J.; Likotrafiti, E.; Argiriou, A. Microbial profiles of Greek PDO cheeses assessed with amplicon metabarcoding. *Food Microbiol.*, **2021**,*99*, 103836. https://doi.org/10.1016/j.fm.2021.103836
62. Penland, M.; Mounier, J.; Pawtowski, A.; Tréguer, S.; Deutsch, S. M.; Coton, M. Use of metabarcoding and source tracking to identify desirable or spoilage autochthonous microorganism sources during black olive fermentations. *Food Res. Int.*, **2021**, *144*, 110344. https://doi.org/10.1016/j.foodres.2021.110344
63. Suwannarach, N.; Kumla, J.; In-On, A.; Lumyong, S. Soil metabarcoding offers a new tool for the investigation and hunting of truffles in Northern Thailand. *J. Fungi*, **2021**, *7(4)*, 293. https://doi.org/10.3390/jof7040293
64. Varela, C.; Cuijvers, K.; Borneman, A. Temporal comparison of microbial community structure in an Australian winery. *Fermentation*, **2021**, *7(3)*,134. https://doi.org/10.3390/fermentation7030134
65. Wirta, H.; Abrego, N.; Miller, K.; Roslin, T.; Vesterinen, E. DNA traces the origin of honey by identifying plants, bacteria and fungi. *Sci. Rep.*, **2021**, *11(1)*, 1-14. https://doi.org/10.1038/s41598-021-84174-0
66. Gutiérrez-Sarmiento, W.; Peña-Ocaña, B. A.; Lam-Gutiérrez, A.; Guzmán-Albores, J. M.; Jasso-Chávez, R.; Ruíz-Valdiviezo, V. M. Microbial community structure, physicochemical characteristics and predictive functionalities of the Mexican tepache fermented beverage. *Microbiol. Res.*, **2022**, *260*, 127045. https://doi.org/10.3390/foods10102390
67. Rollemberg, N. D. C.; Hassemer, G. D. S.; Pierezan, M. D.; Maran, B. M.; Dalla Nora, F. M.; Verruck, S. Identification of fungi in flaxseed (*L. usitatissimum* L.) using the ITS1 and ITS2 intergenic regions. *Microbiol. Res.*, **2022**, *13(2)*, 315-322. https://doi.org/10.3390/microbiolres13020024
68. Banchi, E.; Stankovic, D.; Fernández Mendoza, F.; Pallavicini, A.; Muggia, L. Intrathalline diversity of lichen-inhabiting fungi assessed by metabarcoding of ITS region. *Notiziario della società lichenologica italiana*, **2016**
69. Banchi, E.; Stankovic, D.; Fernández Mendoza, F.; Pallavicini, A.; Muggia, L. ITS2 metabarcoding analysis complements data of lichen mycobiome diversity. *Notiziario della società lichenologica italiana*, **2017**
70. Banchi, E.; Stankovic, D.; Fernández-Mendoza, F.; Gionechetti, F.; Pallavicini, A.; Muggia, L. ITS2 metabarcoding analysis complements lichen mycobiome diversity data. *Mycol. Prog.*, **2018**, *17(9)*, 1049-1066. https://doi.org/10.1007/s11557-018-1415-4
71. Fernández-Mendoza, F.; Fleischhacker, A.; Kopun, T.; Grube, M.; Muggia, L. ITS 1 metabarcoding highlights low specificity of lichen mycobiomes at a local scale. *Mol. Ecol.*, **2017**, *26(18)*, 4811-4830. https://doi.org/10.1111/mec.14244
72. Paul, F.; Otte, J.; Schmitt, I.; Dal Grande, F. Comparing Sanger sequencing and high-throughput metabarcoding for inferring photobiont diversity in lichens. *Sci. Rep.*, **2018**, *8(1)*, 1-7. https://doi.org/10.1038/s41598-018-26947-8
73. Gueidan, C.; Elix, J. A.; McCarthy, P. M.; Roux, C.; Mallen-Cooper, M.; Kantvilas, G. PacBio amplicon sequencing for metabarcoding of mixed DNA samples from lichen herbarium specimens. *MycoKeys*, **2019**, *53*, 73. https://doi.org/10.3897/mycokeys.53.34761

74. Gueidan, C.; Li, L. A long-read amplicon approach to scaling up the metabarcoding of lichen herbarium specimens. *MycoKeys*, **2022**, *86*, 195., https://doi.org/10.3897/mycokeys.86.77431
75. Wright, B.; Clair, L. L. S.; Leavitt, S. D. Is targeted community DNA metabarcoding suitable for biodiversity inventories of lichen-forming fungi?. *Ecol. Indic.*, **2019**, *98*, 812-820. https://doi.org/10.1016/j.ecolind.2018.11.061
76. Yang, J. H.; Oh, S. Y.; Kim, W.; Hur, J. S. Endolichenic fungal community analysis by pure culture isolation and metabarcoding: a case study of *Parmotrema tinctorum*. *Mycobiology*, **2022**, *50(1)*, 55-65. https://doi.org/10.1080/12298093.2022.2040112
77. Reva, O. N.; Zaets, I. E.; Ovcharenko, L. P.; Kukharenko, O. E.; Shpylova, S. P.; Podolich, O. V.; de Vera, J.-P.; Kozyrovska, N. O. Metabarcoding of the kombucha microbial community grown in different microenvironments. *AMB Express*, **2015**, *5(1)*, 1-8. https://doi.org/10.1186/s13568-015-0124-5
78. Zaets, I. Y.; Podolich, O. V.; Reva, O. N.; Kozyrovska, N. O. DNA metabarcoding of microbial communities for healthcare. *Biopolymers and Cell*, **2016**, *32(1)*, 3-8. http://dx.doi.org/10.7124/bc.000906
79. Loyd, A. L.; Richter, B. S.; Jusino, M. A.; Truong, C.; Smith, M. E.; Blanchette, R. A.; Smith, J. A. Identifying the "mushroom of immortality": assessing the *Ganoderma* species composition in commercial Reishi products. *Front. Microbiol.*, **2018**, *9*, 1557. https://doi.org/10.3389/fmicb.2018.01557
80. Guo, M.; Jiang, W.; Yang, M.; Dou, X.; Pang, X. Characterizing fungal communities in medicinal and edible Cassiae Semen using high-throughput sequencing. *Int. J. Food Microbiol.*, **2020**, *319*, 108496. https://doi.org/10.1016/j.ijfoodmicro.2019.108496
81. Xie, H.; Zhao, Q.; Shi, M.; Kong, W.; Mu, W.; Li, B.; Zhao, J.; Zhao, C.; Jia, J.; Liu, J.; Shi, L. Biological ingredient analysis of traditional herbal patent medicine fuke desheng wan using the shotgun metabarcoding approach. *Front. Pharmacol.*, **2021**, *2158. https://doi.org/10.3389/fphar.2021.607197*
82. Bellemain, E.; Davey, M. L.; Kauserud, H.; Epp, L. S.; Boessenkool, S.; Coissac, E.; Geml, J.; Edwards, M.; Willerslev, E.; Gussarova, G.; Taberlet, P.;Brochmann, C. Fungal palaeodiversity revealed using high-throughput metabarcoding of ancient DNA from arctic permafrost. *Environ. Microbiol.*, **2013**, *15(4)*, 1176-1189. https://doi.org/10.1111/1462-2920.12020
83. Coleine, C.; Stajich, J. E.; Zucconi, L.; Onofri, S.; Pombubpa, N.; Egidi, E.; Franks, A.; Buzzini, P.; Selbmann, L. Antarctic cryptoendolithic fungal communities are highly adapted and dominated by Lecanoromycetes and Dothideomycetes. *Front. Microbiol.*, **2018**, *9*, 1392. https://doi.org/10.3389/fmicb.2018.01392
84. Coleine, C.; Zucconi, L.; Onofri, S.; Pombubpa, N.; Stajich, J. E.; Selbmann, L. Sun exposure shapes functional grouping of fungi in cryptoendolithic Antarctic communities. *Life*, **2018**, *8(2)*, 19. https://doi.org/10.3390/life8020019
85. Coleine, C.; Pombubpa, N.; Zucconi, L.; Onofri, S.; Stajich, J. E.; Selbmann, L. Endolithic fungal species markers for harshest conditions in the McMurdo Dry valleys, Antarctica. *Life*, **2020**, *10(2)*, 13. https://doi.org/10.3390/life10020013
86. Saito, R. D.; Connell, L.; Rodriguez, R.; Redman, R.; Libkind, D.; de Garcia, V. Metabarcoding analysis of the fungal biodiversity associated with Castaño Overa glacier–Mount Tronador, Patagonia, Argentina. *Fungal Ecol.*, **2018**, *36*, 8-16. https://doi.org/10.1016/j.funeco.2018.07.006
87. Pang, K. L.; Guo, S. Y.; Chen, I. A.; Burgaud, G.; Luo, Z. H.; Dahms, H. U.; Hwang, J.-S.; Lin, Y.-L.; Huang, J.-S.; Ho, T.-W.; Tsang, L.-M.; M. Chiang, W.-L.; Cha, H. J. Insights into fungal diversity of a shallow-water hydrothermal vent field at Kueishan Island, Taiwan by culture-based and metabarcoding analyses. *PLoS One*, **2019**, *14(12)*, e0226616. https://doi.org/10.1371/journal.pone.0226616
88. Garrido-Benavent, I.; Pérez-Ortega, S.; Durán, J.; Ascaso, C.; Pointing, S. B.; Rodríguez-Cielos, R.; Navarro, F.; de Los Ríos, A. Differential colonization and succession of microbial communities in rock and soil substrates on a maritime antarctic glacier forefield. *Front. Microbiol.*, **2020**, *11*, 126. https://doi.org/10.3389/fmicb.2020.00126
89. Rosa, L. H.; da Silva, T. H.; Ogaki, M. B.; Pinto, O. H. B.; Stech, M.; Convey, P.; Carvalho-Silva, M.; Rosa, C. A.; Câmara, P. E. DNA metabarcoding uncovers fungal diversity in soils of protected and non-protected areas on Deception Island, Antarctica. *Sci. Rep.*, **2020**, *10(1)*, 1-9. https://doi.org/10.1038/s41598-020-78934-7
90. Rosa, L. H.; Pinto, O. H. B.; Convey, P.; Carvalho-Silva, M.; Rosa, C. A.; Câmara, P. E. A. S. DNA metabarcoding to assess the diversity of airborne fungi present over Keller Peninsula, King George Island, Antarctica. *Microb. Ecol.*, **2021**, *82(1)*, 165-172. https://doi.org/10.1007/s00248-020-01627-1
91. Azpiazu-Muniozguren, M.; Perez, A.; Rementeria, A.; Martinez-Malaxetxebarria, I.; Alonso, R.; Laorden, L.; Gamboa, J.; Bikandi, J.; Garaizar, J.; Martinez-Ballesteros, I. Fungal diversity and composition of the continental solar saltern in Añana Salt Valley (Spain). *J. Fungi*, **2021**, 7(12), 1074. https://doi.org/10.3390/jof7121074
92. de Menezes, G. C. A.; Câmara, P. E.; Pinto, O. H. B.; Carvalho-Silva, M.; Oliveira, F. S.; Souza, C. D.; Schaefer, C. E. G. R.; Convey, P.; Rosa, C. A.; Rosa, L. H. Fungal diversity present on rocks from a polar desert in continental Antarctica assessed using DNA metabarcoding. *Extremophiles*, **2021**, *25(2)*, 193-202. https://doi.org/10.1007/s00792-021-01221-4
93. de Souza, L.; Ogaki, M. B.; Câmara, P. E.; Pinto, O. H.; Convey, P.; Carvalho-Silva, M.; Rosa, C. A.; Rosa, L. H. Assessment of fungal diversity present in lakes of Maritime Antarctica using DNA metabarcoding: a temporal microcosm experiment. *Extremophiles*, **2021**, *25(1)*, 77-84. https://doi.org/10.1007/s00792-020-01212-x
94. de Souza, L. M. D.; Lirio, J. M.; Coria, S. H.; Lopes, F. A. C.; Convey, P.; Carvalho-Silva, M.; Soares de Oliveira, F.; Rosa, C. A.; Câmara, P. E. A. S.; Rosa, L. H. Diversity, distribution and ecology of fungal communities present in Antarctic lake sediments uncovered by DNA metabarcoding. *Sci. Rep.*, **2022**, *12(1)*, 1-8. https://doi.org/10.1007/s00792-020-01212-x
95. Ogaki, M. B.; Pinto, O. H. B.; Vieira, R.; Neto, A. A.; Convey, P.; Carvalho-Silva, M.; Rosa, C. A.; Câmara, P. E. A. S.; Rosa, L. H. Fungi present in Antarctic deep-sea sediments assessed using DNA metabarcoding. *Microb. Ecol.*, **2021**, *82(1)*, 157-164. https://doi.org/10.1007/s00248-020-01658-8

96. da Silva, T. H.; Câmara, P. E.; Pinto, O. H. B.; Carvalho-Silva, M.; Oliveira, F. S.; Convey, P.; Rosa, L. H. Diversity of fungi present in permafrost in the South Shetland Islands, maritime Antarctic. *Microbial Ecol.*, **2022**, *83(1)*, 58-67. https://doi.org/10.1007/s00248-021-01735-6
97. Seeber, P. A.; von Hippel, B.; Kauserud, H.; Löber, U.; Stoof-Leichsenring, K. R.; Herzschuh, U.; Epp, L. S. Evaluation of lake sedimentary ancient DNA metabarcoding to assess fungal biodiversity in Arctic paleoecosystems. *Environ. DNA*. **2022**. https://doi.org/10.1002/edn3.315
98. Geml, J.; Gravendeel, B.; van der Gaag, K. J.; Neilen, M.; Lammers, Y.; Raes, N.; Semenova, T. A.; de Knijff, P.; Noordeloos, M. E. The contribution of DNA metabarcoding to fungal conservation: diversity assessment, habitat partitioning and mapping red-listed fungi in protected coastal *Salix repens* communities in the Netherlands. *PloS One*, **2014**, *9(6)*, e99852. https://doi.org/10.1371/journal.pone.0099852
99. Chariton, A. A.; Stephenson, S.; Morgan, M. J.; Steven, A. D.; Colloff, M. J.; Court, L. N.; Hardy, C. M. Metabarcoding of benthic eukaryote communities predicts the ecological condition of estuaries. *Environ. Pollut.*, **2015**, *203*, 165-174. https://doi.org/10.1016/j.envpol.2015.03.047
100. Guardiola, M.; Uriz, M. J.; Taberlet, P.; Coissac, E.; Wangensteen, O. S.; Turon, X. Deep-sea, deep-sequencing: metabarcoding extracellular DNA from sediments of marine canyons. *PloS One*, **2015**, *10(10)*, e0139633. https://doi.org/10.1371/journal.pone.0139633
101. Li, W.; Wang, M. M.; Wang, X. G.; Cheng, X. L.; Guo, J. J.; Bian, X. M.; Cai, L. Fungal communities in sediments of subtropical Chinese seas as estimated by DNA metabarcoding. *Sci. Rep.*, **2016**, *6(1)*,1-9. https://doi.org/10.1038/srep26528
102. Lanzen, A.; Lekang, K.; Jonassen, I.; Thompson, E. M.; Troedsson, C. DNA extraction replicates improve diversity and compositional dissimilarity in metabarcoding of eukaryotes in marine sediments. *PLoS One*, **2017**, *12(6)*, e0179443. https://doi.org/10.1371/journal.pone.0179443
103. Li, P. D.; Jeewon, R.; Aruna, B.; Li, H. Y.; Lin, F. C.; Wang, H. K. Metabarcoding reveals differences in fungal communities between unflooded versus tidal flat soil in coastal saline ecosystem. *Sci. Total Environ.*, **2019**, *690*, 911-922. https://doi.org/10.1016/j.scitotenv.2019.06.473
104. Polinski, J. M.; Bucci, J. P.,.; Gasser, M.; Bodnar, A. G. Metabarcoding assessment of prokaryotic and eukaryotic taxa in sediments from Stellwagen Bank National Marine Sanctuary. *Sci. Rep.*, **2019**, *9(1)*,1-8. https://doi.org/10.1038/s41598-019-51341-3
105. Retter, A.; Nilsson, R. H.; Bourlat, S. J. Exploring the taxonomic composition of two fungal communities on the Swedish west coast through metabarcoding. *Biodivers. Data J.*, **2019**, *7*, e35332. https://doi.org/10.3897/BDJ.7.e35332
106. Góes-Neto, A.; Marcelino, V. R.; Verbruggen, H.; da Silva, F. F.; Badotti, F. Biodiversity of endolithic fungi in coral skeletons and other reef substrates revealed with SSU rDNA metabarcoding. *Coral Reefs*, **2020**, *39(1)*, 229-238. https://doi.org/10.1007/s00338-019-01880-y
107. Luo, Y., Wei, X.; Yang, S.; Gao, Y. H.; Luo, Z. H. Fungal diversity in deep-sea sediments from the Magellan seamounts as revealed by a metabarcoding approach targeting the ITS2 regions. *Mycology*, **2020**, *11(3)*, 214-229. https://doi.org/10.1080/21501203.2020.1799878
108. Wurzbacher, C.; Grimmett, I. J.; Bärlocher, F. Metabarcoding-based fungal diversity on coarse and fine particulate organic matter in a first-order stream in Nova Scotia, Canada. *F1000Research*, **2015**, *4*. 1378. https://doi.org/10.12688/f1000research.7359.2
109. Duarte, S.; Cássio, F.; Pascoal, C.; Bärlocher, F. Taxa-area relationship of aquatic fungi on deciduous leaves. *PloS One*, **2017**, *12(7)*, e0181545. https://doi.org/10.1371/journal.pone.0181545
110. Matsuoka, S.; Sugiyama, Y.; Sato, H.; Katano, I.; Harada, K.; Doi, H. Spatial structure of fungal DNA assemblages revealed with eDNA metabarcoding in a forest river network in western Japan. *Metabarcoding Metagenom.*, **2019**, *3*, e36335. https://doi.org/10.3897/mbmg.3.36335
111. Franco Ortega, S.; Ferrocino, I.; Adams, I.; Silvestri, S.; Spadaro, D.; Gullino, M. L.; Boonham, N. Monitoring and surveillance of aerial mycobiota of rice paddy through DNA metabarcoding and qPCR. *J. Fungi*, **2020**, *6(4)*,372. https://doi.org/10.3390/jof6040372
112. Matsuoka, S.; Sugiyama, Y.; Shimono, Y.; Ushio, M.; Doi, H. Evaluation of seasonal dynamics of fungal DNA assemblages in a flow-regulated stream in a restored forest using eDNA metabarcoding. *Environ. Microbiol.*, **2021**, *23(8)*, 4797-4806. https://doi.org/10.1111/1462-2920.15669
113. Zhang, A.; Wang, J.; Hao, Y.; Xiao, S.; Luo, W.; Wang, G.; Zhou, Z. Community characteristics analysis of eukaryotic microplankton via ITS gene metabarcoding based on environmental DNA in lower reaches of Qiantang River, China. *Open J. Anim. Sci.*, **2021**, *11(2)*, 105-124. https://doi.org/10.4236/ojas.2020.112009
114. Ossowski, S.; Bezdan, D.; MetaSUB International Consortium. The metagenomics and metadesign of the subways and urban biomes (MetaSUB) international consortium inaugural meeting report. *Microbiome*, **2016**, *4*, 24. https://doi.org/10.1186/s40168-016-0168-z
115. Reese, A. T.; Savage, A.; Youngsteadt, E.; McGuire, K. L.; Koling, A.; Watkins, O.; Frank, S. D.; Dunn, R. R. Urban stress is associated with variation in microbial species composition—but not richness—in Manhattan. *ISME J.*, **2016**, *10(3)*, 751-760. https://doi.org/10.1038/ismej.2015.152
116. Danko, D.; Bezdan, D.; Afshin, E. E.; Ahsanuddin, S.; Bhattacharya, C.; Butler, D. J.; Chng, K. R.; Donnellan, D.; Hecht, J.; Jackson, K.; Kuchin, K.; Karasikov, M.; Lyons, A.; Mak, L.; Meleshko, D.; Mustafa, H.; Mutai, B.; Neches, R. Y.; Ng, A.; Nikolayeva, O.; Nikolayeva, T.; Png, E.; Ryon, K. A.; Sanchez, J. L.; Shaaban, H.; Sierra, M. A.; Thomas, D.; Young, B.; Abudayyeh, O. O.; Alicea, J.; Bhattacharyya, M.; Blekhman, R.; Castro-Nallar, E.; Cañas, A. M.; Chatziefthimiou, A. D.; Crawford, R. W.; De Filippis, F.; Deng, Y.; Desnues, C.; Dias-Neto, E.; Dybwad, M.; Elhaik; E.; Ercolini, D.; Frolova, A.; Gankin, D.; Gootenberg, J. S.; Graf, A. B.;

Green, D. C.; Hajirasouliha, I.; Hastings, J. J. A.; Hernandez, M.; Iraola, G.; Jang, S.; Kahles, A.; Kelly, F. J.; Knights, K.; Kyrpides, N. C.; Łabaj, P. P.; Lee, P. K. H.; Leung, M. H. Y.; Ljungdahl, P. O.; Mason-Buck, G.; McGrath, K.; Meydan, C.; Mongodin, E F.; Moraes, M. O.; Nagarajan, N.; Nieto-Caballero, M.; Noushmehr, H.; Oliveira, M.; Ossowski, S.; Osuolale, O. O.; Özcan, O.; Paez-Espino, D.; Rascovan, N.; Richard, H.; Rätsch, G.; Schriml, L. M.; Semmler, T.; Sezerman, O. U.; Shi, L.; Shi, T.; Siam, R.; Song, L. H.; Suzuki, H.; Syndercombe Court, D.; Tighe, S. W.; Tong, X.; Udekwu, K. I.; Ugalde, J. A.; Valentine, B.; Vassilev, D. I.; Vayndorf, E. M.; Velavan, T. P.; Wu, J.; Zambrano, M. M.; Zhu, J.; Zhu, S.; Mason, C. E.; The International MetaSUB Consortium. A global metagenomic map of urban microbiomes and antimicrobial resistance. *Cell*, *2021*, *184(13)*, 3376-3393. https://doi.org/10.1016/j.cell.2021.05.002

117. Dale, A. L.; Feau, N.; Berube, J. A.; Ponchart, J.; Bilodeau, G. J.; Hamelin, R. C. Urban environments harbor greater oomycete and Phytophthora diversity, creating a bridgehead for potential new pathogens to natural ecosystems. *Environ. DNA*, **2022**. https://doi.org/10.1002/edn3.300
118. Jiang, S.; Sun, B.; Zhu, R.; Che, C.; Ma, D.; Wang, R.; Dai, H. Airborne microbial community structure and potential pathogen identification across the PM size fractions and seasons in the urban atmosphere. *Sci. Total Environ.*, **2022**, *831*, 154665. https://doi.org/10.1016/j.scitotenv.2022.154665
119. De Beeck, M. O.; Ruytinx, J., Smits, M. M.; Vangronsveld, J.; Colpaert, J. V.; Rineau, F. Belowground fungal communities in pioneer Scots pine stands growing on heavy metal polluted and non-polluted soils. *Soil Biol. Biochem.*, **2015**, *86*, 58-66. https://doi.org/10.1016/j.soilbio.2015.03.007
120. Davidov, K.; Iankelevich-Kounio, E.; Yakovenko, I.; Koucherov, Y.; Rubin-Blum, M.; Oren, M. Identification of plastic-associated species in the Mediterranean Sea using DNA metabarcoding with Nanopore MinION. *Sci. Rep.*, **2020**, *10(1)*, 1-11. https://doi.org/10.1038/s41598-020-74180-z
121. Gkoutselis, G.;, Rohrbach, S.; Harjes, J.; Obst, M.; Brachmann, A.; Horn, M. A.; Rambold, G. Microplastics accumulate fungal pathogens in terrestrial ecosystems. *Sci. Rep.*, **2021**, *11(1)*, 1-13. https://doi.org/10.1038/s41598-021-92405-7
122. Mejia, M. P.; Rojas, C.; Curd, E.; Renshaw, M.; Edalati, K.; Shih, B.; Vincent, N.; Lin, M.; Nguyen, P.; Wayne, R.; Jessup, K.; Parker, S. Soil microbial community composition and tolerance to contaminants in an urban brownfield site. *ResearchSquare*, **2022**
123. Pollegioni, P.; Mattioni, C.; Ristorini, M.; Occhiuto, D.; Canepari, S.; Korneykova, M. V.; Gavrichkova, O. Diversity and Source of Airborne Microbial Communities at Differential Polluted Sites of Rome. *Atmosphere*, **2022**, *13(2)*, 224. https://doi.org/10.3390/atmos13020224
124. Crognale, S.; D'Annibale, A.; Pesciaroli, L.; Stazi, S. R.; Petruccioli, M. Fungal community structure and as-resistant fungi in a decommissioned gold mine site. *Front. Microbiol.*, **201**, *8*, 72202. https://doi.org/10.3389/fmicb.2017.02202
125. Villarreal-Ruiz, L.; Neri-Luna, C.; Tedersoo, L.; Koljalg, U. Global and Mexican local diversity of mycorrhizal fungi in forest soils revealed by Next-Generation Sequencing: A preliminary aproach. Exploring Microorganisms: Recent Advances in Applied Microbiology. Brown Walker Press, Irvine & Boca Raton, **2018**, 2-5.
126. Durand, A.; Maillard, F.; Foulon, J.; Gweon, H. S.; Valot, B.; Chalot, M. Environmental metabarcoding reveals contrasting belowground and aboveground fungal communities from poplar at a Hg phytomanagement site. *Microb. Ecol.*, **2017**, *74(4)*, 795-809. https://doi.org/10.1007/s00248-017-0984-0
127. Jimu, L.; Kemler, M.; Mujuru, L.; Mwenje, E. Illumina DNA metabarcoding of *Eucalyptus* plantation soil reveals the presence of mycorrhizal and pathogenic fungi. *Int. J. For. Res.*, **2018**, *91(2)*, 238-245. https://doi.org/10.1093/forestry/cpx046
128. Landinez-Torres, A.; Panelli, S.; Picco, A. M.; Comandatore, F.; Tosi, S.; Capelli, E. A meta-barcoding analysis of soil mycobiota of the upper Andean Colombian agro-environment. *Sci. Rep.*, **2019**, *9(1)*, 1-12. https://doi.org/10.1038/s41598-019-46485-1
129. Riddell, C. E.; Frederickson-Matika, D.; Armstrong, A. C.; Elliot, M.; Forster, J.; Hedley, P. E.; Morris, J.; Thorpe, P.; Cooke, D. E. L.; Pritchard, L.; Sharp, P. M.; Green, S. Metabarcoding reveals a high diversity of woody host-associated *Phytophthora* spp. in soils at public gardens and amenity woodlands in Britain. *PeerJ*, **2019**, *7*, e6931. https://doi.org/10.7717/peerj.6931
130. Abrego, N.; Crosier, B.; Somervuo, P.; Ivanova, N.; Abrahamyan, A.; Abdi, A.; Hämäläinen, K; Junninen, K.; Maunula, M.; Purhonen, J.; Ovaskainen, O. Fungal communities decline with urbanization—more in air than in soil. *ISME J.*, **2020**, *14(11)*, 2806-2815. https://doi.org/10.1038/s41396-020-0732-1
131. Baruch, Z.; Liddicoat, C.; Laws, M.; Marker, L. K.; Morelli, H.; Yan, D.; Young, J. M.; Breed, M. F. Characterising the soil fungal microbiome in metropolitan green spaces across a vegetation biodiversity gradient. *Fungal Ecol.*, **2020**, *47*, 100939. https://doi.org/10.1016/j.funeco.2020.100939
132. Kepler, R. M.; Epp Schmidt, D. J.; Yarwood, S. A.; Cavigelli, M. A.; Reddy, K. N.; Duke, S. O.; Bradley, C. A.; Williams Jr., M. M.; Buyer, J. S.; Maul, J. E. Soil microbial communities in diverse agroecosystems exposed to the herbicide glyphosate. *App. Environ. Microbiol.*, **2020**, *86(5)*, e01744-19. https://doi.org/10.1128/AEM.01744-19
133. Rao, M. V.; Rice, R. A.; Fleischer, R. C.; Muletz-Wolz, C. R. Soil fungal communities differ between shaded and sun-intensive coffee plantations in El Salvador. *PloS One*, **2020**, *15(4)*, e0231875. https://doi.org/10.1371/journal.pone.0231875
134. Alimi, A. A.; Ezeokoli, O. T.; Adeleke, R.; Moteetee, A. Arbuscular mycorrhizal fungal communities colonising the roots of indigenous legumes of South Africa as revealed by high-throughput DNA metabarcoding. *Rhizosphere*, **2021**, *19*, 100405. https://doi.org/10.1016/j.rhisph.2021.100405
135. Marczylo, E. L.; Macchiarulo, S.; Gant, T. W. Metabarcoding of soil fungi from different urban greenspaces around Bournemouth in the UK. *EcoHealth*, **2021**, *18(3)*, 315-330. https://doi.org/10.1007/s10393-021-01523-1
136. Victorino, Í. M. M.; Voyron, S.; Caser, M.; Orgiazzi, A.; Demasi, S.; Berruti, A.; Lumini, E. Metabarcoding of soil fungal communities associated with alpine field-grown saffron (*Crocus sativus* L.) inoculated with AM fungi. *J. Fungi*, **2021**, *7(1)*, 45. https://doi.org/10.3390/jof7010045

137. Navarro-Noya, Y. E.; Montoya-Ciriaco, N.; Muñoz-Arenas, L. C.; Hereira-Pacheco, S.; Estrada-Torres, A.; Dendooven, L. Conversion of a high-altitude temperate forest for agriculture reduced alpha and beta diversity of the soil fungal communities as revealed by a metabarcoding analysis. *Front. Microbiol.*, **2021**, *12*, 667566. https://doi.org/10.3389/fmicb.2021.667566
138. Rossmann, S.; Lysøe, E.; Skogen, M.; Talgø, V.; Brurberg, M. B. DNA metabarcoding reveals broad presence of plant pathogenic oomycetes in soil from internationally traded plants. *Front. Microbiol.*, **2021**, *12*, 645. https://doi.org/10.3389/fmicb.2021.637068
139. Hashizume, H.; Taga, S.; Sakata, M. K.; Taha, M. H. M.; Siddig, E. E.; Minamoto, T.; Fahal, A. H.; Kaneko, S. Detection of multiple mycetoma pathogens using fungal metabarcoding analysis of soil DNA in an endemic area of Sudan. *PLOS Negl. Trop. Dis.*, **2022**, *16(3)*, e0010274. https://doi.org/10.1371/journal.pntd.0010274
140. Alem, D.; Dejene, T.; Geml, J.; Oria-de-Rueda, J. A.; Martín-Pinto, P. Metabarcoding analysis of the soil fungal community to aid the conservation of underexplored church forests in Ethiopia. *Sci. Rep.*, **2022**, *12(1)*, 1-14. https://doi.org/10.1038/s41598-022-08828-3
141. Câmara, P. E.; Bones, F. L. V.; Lopes, F. A. C.; Oliveira, F. S.; Barreto, C. C.; Knop Henriques, D.; Campos, L. P., Carvalho-Silva, M.; Convey, P.; Rosa, L. H. DNA metabarcoding reveals cryptic diversity in forest soils on the isolated Brazilian Trindade Island, South Atlantic. *Microbial Ecol.*, **2022**, 1-16. https://doi.org/10.1007/s00248-022-02018-4
142. Monteiro, J. S.; Almeida, M. S.; Medeiros-Sarmento, P. S.; Caldeira Junior, C. F.; Ramos, S. J.; de Oliveira, G. C.; Valadares, R. B. S.; Gastauer, M. DNA metabarcoding reveals compositional and functional differences in fungal communities among amazonian canga formations. **2022**. http://dx.doi.org/10.2139/ssrn.4059087
143. Banchi, E.; Ametrano, C. G.; Stanković, D.; Verardo, P.; Moretti, O.; Gabrielli, F.; Lazzarin, S.; Borney, Tassan, M. F.; Tretiach, M.; Pallavicini, A.; Muggia, L. DNA metabarcoding uncovers fungal diversity of mixed airborne samples in Italy. *PloS One*, **2018**, *13(3)*, e0194489. https://doi.org/10.1371/journal.pone.0194489
144. Aalismail, N. Metabarcoding and metagenomic characterizations of the Red Sea sector of the global dust belt's microbiome, Doctoral Thesis, King Abdullah University of Science and Technology, **2020**
145. Banchi, E.; Ametrano, C. G.; Tordoni, E.; Stanković, D.; Ongaro, S.; Tretiach, M.; Pallavicini, A.; Lazzarin, S. Environmental DNA assessment of airborne plant and fungal seasonal diversity. *Sci. Total Environ.*, **2020**, *738*, 140249. https://doi.org/10.1016/j.scitotenv.2020.140249
146. de Groot, G. A.; Geisen, S.; Wubs, E. J.; Meulenbroek, L.; Laros, I.; Snoek, L. B.; Lammertsma; D. R.; Hansenef, L. H.; Slim, P. A. The aerobiome uncovered: multi-marker metabarcoding reveals potential drivers of turn-over in the full microbial community in the air. *Environ. Int.*, **2021**, *154*, 106551. https://doi.org/10.1016/j.envint.2021.106551
147. Tordoni, E.; Ametrano, C. G.; Banchi, E.; Ongaro, S.; Pallavicini, A.; Bacaro, G.; Muggia, L. Integrated eDNA metabarcoding and morphological analyses assess spatio-temporal patterns of airborne fungal spores. *Ecol. Indic.*, **2021**, *121*, 107032. https://doi.org/10.1016/j.ecolind.2020.107032
148. Hanson, M. C.; Petch, G. M.; Ottosen, T. B.; Skjøth, C. A. Climate change impact on fungi in the atmospheric microbiome. *Sci. Total Environ.*, **2022**, *830*, 154491. https://doi.org/10.1016/j.scitotenv.2022.154491
149. Korpelainen, H.; Pietiläinen, M.; Huotari, T. Effective detection of indoor fungi by metabarcoding. *Ann. Microbiol.*, **2016**, *66(1)*, 495-498. https://doi.org/10.1007/s13213-015-1118-x
150. Korpelainen, H.; Pietiläinen, M. Diversity of indoor fungi as revealed by DNA metabarcoding. Genome, **2017**, *60(1)*, 55-64. https://doi.org/10.1139/gen-2015-0191
151. Rocchi, S.; Valot, B.; Reboux, G.; Millon, L. DNA metabarcoding to assess indoor fungal communities: Electrostatic dust collectors and Illumina sequencing. *J. Microbiol. Methods*, **2017**, *139*, 107-112. https://doi.org/10.1016/j.mimet.2017.05.014
152. Martin-Sanchez, P. M.; Estensmo, E. L. F.; Morgado, L. N.; Maurice, S.; Engh, I. B.; Skrede, I.; Kauserud, H. Analysing indoor mycobiomes through a large-scale citizen science study in Norway. *Mol. Ecol.*, **2021**, *30(11)*, 2689-2705. https://doi.org/10.1111/mec.15916
153. Straumfors, A.; Foss, O. A.; Fuss, J.; Mollerup, S. K.; Kauserud, H.; Mundra, S. The inhalable mycobiome of sawmill workers: exposure characterization and diversity. *App. Environ. Microbiol.*, **2019**, *85(21)*, e01448-19. https://doi.org/10.1128/AEM.01448-19
154. Chng, K. R.; Li, C.; Bertrand, D.; Ng, A. H. Q.; Kwah, J. S.; Low, H. M.; Tong, C.; Natrajan, M.; Zhang, M. H.; Xu, L.; Ko, K. K. K.; Ho, E. X. P.; Av-Shalom, T. V.; Teo, J. W. P.; Khor, C. C.; The International MetaSUB Consortium, Chen, S. L.; Mason, C. E.; Ng, O. T.; Marimuthu, K.; Ang, B.; Nagarajan, N. Cartography of opportunistic pathogens and antibiotic resistance genes in a tertiary hospital environment. *Nat. Med.*, **2020**, *26(6)*, 941-951. https://doi.org/10.1038/s41591-020-0894-4
155. Estensmo, E. L. F. The diversity and seasonality of the indoor mycobiome. Doctoral Thesis, University of Oslo, **2021**
156. Estensmo, E. L. F.; Botnen, S. S.; Maurice, S.; Martin-Sanchez, P. M.; Morgado, L.; Engh, I. B.; Høiland, K.; Skrede, I.; Kauserud, H. The indoor mycobiome of daycare centers is affected by occupancy and climate. *bioRxiv*, **2021**. https://doi.org/10.1128/aem.02113-21
157. Minahan, N. T.; Chen, C. H.; Shen, W. C.; Lu, T. P.; Kallawicha, K.; Tsai, K. H.; Guo, Y. L. Fungal spore richness in school classrooms is related to surrounding forest in a season-dependent manner. *Microb. Ecol.*, **2021**, 1-12. https://doi.org/10.1007/s00248-021-01844-2
158. Young, J. M.; Weyrich, L. S.; Breen, J.; Macdonald, L. M.; Cooper, A. Predicting the origin of soil evidence: High throughput eukaryote sequencing and MIR spectroscopy applied to a crime scene scenario. *Forensic Sci. Int.*, **2015**, *251*, 22-31. https://doi.org/10.1016/j.forsciint.2015.03.008
159. Brunbjerg, A. K.,; Bruun, H. H.; Hansen, A. J.; Ejrnæs, R. Predicting provenance of forensic soil samples: Linking soil to ecological habitats by metabarcoding and supervised classification. *PLoS One*, **2019**, *14(7)*, e0202844. https://doi.org/10.1371/journal.pone.0202844

160. Fu, X.; Guo, J.; Finkelbergs, D.; He, J.; Zha, L.; Guo, Y.; Cai, J. Fungal succession during mammalian cadaver decomposition and potential forensic implications. *Sci. Rep.*, **2019**, *9(1)*, 1-9. https://doi.org/10.1038/s41598-019-49361-0
161. Procopio, N.; Ghignone, S.; Voyron, S.; Chiapello, M.; Williams, A.; Chamberlain, A.; Mello, A.; Buckley, M. Soil fungal communities investigated by metabarcoding within simulated forensic burial contexts. *Front. Microbiol.*, **2020**, *11*, 1686. https://doi.org/10.3389/fmicb.2020.01686
162. Giampaoli, S.; De Vittori, E.; Frajese, G. V.; Paytuví, A.; Sanseverino, W.; Anselmo, A.; Barni, F.; Berti, A. A semi-automated protocol for NGS metabarcoding and fungal analysis in forensic. *Forensic Sci. Int.*, **2020**, *306*, 110052. https://doi.org/10.1016/j.forsciint.2019.110052
163. Giampaoli, S.; De Vittori, E.; Barni, F.; Anselmo, A.; Rinaldi, T.; Baldi, M.; Miranda, K. C.; Liao, A.; Brami, D.; Frajese, G. V.; Berti, A. DNA metabarcoding of forensic mycological samples. *Egypt. J. Forensic Sci.*, **2021**, *11(1)*. https://doi.org/10.1186/s41935-021-00221-x
164. Saverio, G.; Filippo, B.; Anselmo, A.; Rinaldi, T.; Baldi, M.; Miranda, K. C.; Liao, Arnold; Brami, D.; Frajese, G. V.; Berti, A. DNA metabarcoding of forensic mycological samples. *Egypt. J. Forensic Sci.*, **2021**, *11(1)*. https://doi.org/10.1186/s41935-021-00221-x
165. Karadayı, S. Assessment of the link between evidence and crime scene through soil bacterial and fungal microbiome: A mock case in forensic study. *Forensic Sci. Int.*, **2021**, *329*, 111060. https://doi.org/10.1016/j.forsciint.2021.111060
166. Langarica-Fuentes, A.; Fox, G.; Robson, G. D. Metabarcoding analysis of home composts reveals distinctive fungal communities with a high number of unassigned sequences. *Microbiology*, **2015**, *161(10)*, 1921-1932. https://doi.org/10.1099/mic.0.000153
167. López-González, J. A.; del Carmen Vargas-García, M.; López, M. J.; Suárez-Estrella, F.; del Mar Jurado, M.; Moreno, J. Biodiversity and succession of mycobiota associated to agricultural lignocellulosic waste-based composting. *Bioresour. Technol.*, **2015**, *187*, 305-313. https://doi.org/10.1016/j.biortech.2015.03.124
168. de Oliveira, T. B.; Lopes, V. C. P.; Barbosa, F. N.; Ferro, M.; Meirelles, L. A.; Sette, L. D.; Gomes, E.; Rodrigues, A. Fungal communities in pressmud composting harbour beneficial and detrimental fungi for human welfare. *Microbiology*, **2016**, *162(7)*, 1147-1156. https://doi.org/10.1099/mic.0.000306
169. Tian, X.; Yang, T.; He, J.; Chu, Q.; Jia, X.; Huang, J. Fungal community and cellulose-degrading genes in the composting process of Chinese medicinal herbal residues. *Bioresour. Technol.*, **2017**, *241*, 374-383. https://doi.org/10.1016/j.biortech.2017.05.116
170. Gaylarde, C.; Baptista-Neto, J. A.; Ogawa, A.; Kowalski, M.; Celikkol-Aydin, S.; Beech, I. Epilithic and endolithic microorganisms and deterioration on stone church facades subject to urban pollution in a sub-tropical climate. *Biofouling*, **2017**, *33(2)*, 113-127. https://doi.org/10.1080/08927014.2016.1269893
171. Gaylarde, C.; Ogawa, A.; Beech, I.; Kowalski, M.; Baptista-Neto, J. A. Analysis of dark crusts on the church of Nossa Senhora do Carmo in Rio de Janeiro, Brazil, using chemical, microscope and metabarcoding microbial identification techniques. *Int. Biodeterior. Biodegradation*, **2017**, *117*, 60-67. https://doi.org/10.1016/j.ibiod.2016.11.028
172. Trovão, J.,; Gil, F.; Catarino, L.; Soares, F.; Tiago, I.; Portugal, A. Analysis of fungal deterioration phenomena in the first Portuguese King tomb using a multi-analytical approach. *Int. Biodeterior. Biodegradation*, **2020**, *149*, 104933. https://doi.org/10.1016/j.ibiod.2020.104933
173. Cennamo, P.; De Luca, D. (2022, April). A metabarcoding approach for the study of biodeterioration of ancient wall paintings in an Italian cave. In Journal of Physics: Conference Series, **2022**, *2204(1)*, 012011.
174. Checcucci, A.; Borruso, L.; Petrocchi, D.; Perito, B. Diversity and metabolic profile of the microbial communities inhabiting the darkened white marble of Florence Cathedral. *Int. Biodeterior. Biodegradation*, **2022**, *171*, 105420. https://doi.org/10.1016/j.ibiod.2022.105420

References: Supplementary Table S10

1. Neblett Fanfair, R.; Benedict, K.; Bos, J.; Bennett, S. D.; Lo, Y. C.; Adebanjo, T.; Etienne, K.; Deak, E.; Derado, G.; Shieh, W.-J.; Drew, C.; Zaki, S.; Sugerman, D.; Gade, L.; Thompson, E. H.; Sutton, D. A.; Engelthaler, D. M.; Schupp, J. M.; Brandt, M. E.; Harris, J. R.; Lockhart, S. R.; Turabelidze, G.; Park, B. J. Necrotizing cutaneous mucormycosis after a tornado in Joplin, Missouri, in 2011. *N. Engl. J. Med.*, **2012**, *367(23)*, 2214-2225. https://doi.org/10.1056/NEJMoa1204781
2. Gade, L.; Scheel, C. M.; Pham, C. D.; Lindsley, M. D.; Iqbal, N.; Cleveland, A. A.; Whitney, A. M.; Lockhart, S. R.; Brandt, M. E.; Litvintseva, A. P. Detection of fungal DNA in human body fluids and tissues during a multistate outbreak of fungal meningitis and other infections. *Eukaryot. Cell*, **2013**, *12(5)*, 677-683. https://doi.org/10.1128/EC.00046-13
3. Litvintseva, A. P.; Hurst, S.; Gade, L.; Frace, M. A.; Hilsabeck, R.; Schupp, J. M.; Gillece, J. D.; Roe, C.; Smith, D.; Keim, P.; Lockhart, S. R.; Changayil, S.; Weil, M. R.; MacCannell, D. R.; Brandt, M. E.; Engelthaler, D. M. Whole-genome analysis of *Exserohilum rostratum* from an outbreak of fungal meningitis and other infections. *J. Clin. Microbiol.*, **2014**, *52(9)*, 3216-3222. https://doi.org/10.1128/JCM.00936-14
4. Cuomo, C. A.; Rodriguez-Del Valle, N.; Perez-Sanchez, L.; Abouelleil, A.; Goldberg, J.; Young, S.; Zeng, Q.; Birren, B. W. Genome sequence of the pathogenic fungus *Sporothrix schenckii* (ATCC 58251). *Genome Announc.*, **2014**, *2(3)*, e00446-14. https://doi.org/10.1128/genomeA.00446-14
5. Vandeputte, P.; Ghamrawi, S.; Rechenmann, M.; Iltis, A.; Giraud, S.; Fleury, M.; Thornton, C.; Delhaès, L.; Meyer, W.; Papon, N.; Bouchara, J. P. Draft genome sequence of the pathogenic fungus *Scedosporium apiospermum*. *Genome Announc.*, **2014**, *2(5)*, e00988-14. https://doi.org/10.1128/genomeA.00988-14
6. Sharma, C.; Kumar, N.; Meis, J. F.; Pandey, R.; Chowdhary, A. Draft genome sequence of a fluconazole-resistant *Candida auris* strain from a candidemia patient in India. *Genome Announc.*, **2015**, *3(4)*, e00722-15. https://doi.org/10.1128/genomeA.00722-15

7. Sharma, C.; Kumar, N.; Pandey, R.; Meis, J. F.; Chowdhary, A. Whole genome sequencing of emerging multidrug resistant *Candida auris* isolates in India demonstrates low genetic variation. *New Microbes New Infect.*, **2016**, *13*, 77-82. https://doi.org/10.1016/j.nmni.2016.07.003
8. Chowdhary, A.; Sharma, C.; Meis, J. F. *Candida auris*: a rapidly emerging cause of hospital-acquired multidrug-resistant fungal infections globally. *PLoS Pathog.*, **2017**, *13(5)*, e1006290. https://doi.org/10.1371/journal.ppat.1006290
9. Lockhart, S. R.,; Etienne, K. A.; Vallabhaneni, S.; Farooqi, J.; Chowdhary, A.; Govender, N. P.; Colombo, A. L.; Calvo, B.; Cuomo, C. A.; Desjardins, C. A.; Berkow, E. L.; Castanheira, M.; Magobo, R. E.; Jabeen, K.; Asghar, R. J.; Meis, J. F.; Jackson, B.; Chiller, T.; Litvintseva, A. P. Simultaneous emergence of multidrug-resistant *Candida auris* on 3 continents confirmed by whole-genome sequencing and epidemiological analyses. *Clin. Infect. Dis.*, **2017**, *64(2)*, 134-140. https://doi.org/10.1093/cid/ciw691
10. Rhodes, J.; Abdolrasouli, A.; Farrer, R. A.; Cuomo, C. A.; Aanensen, D. M.; Armstrong-James, D.; Fisher, M. C.; Schelenz, S. Genomic epidemiology of the UK outbreak of the emerging human fungal pathogen *Candida auris*. *Emerg. Microbes Infect.*, **2018**, *7(1)*, 1-12. https://doi.org/10.1038/s41426-018-0045-x
11. Pchelin, I. M.; Azarov, D. V.; Churina, M. A.; Ryabinin, I. A.; Vibornova, I. V.; Apalko, S. V.; Kruglov, A. N.; Sarana, A. M.; Taraskina, A. E.; Vasilyeva, N. V. Whole genome sequence of first *Candida auris* strain, isolated in Russia. *Med. Mycol.*, **2020**, *58(3)*, 414-416. https://doi.org/10.1093/mmy/myz078
12. Welsh, R. M.; Misas, E.; Forsberg, K.; Lyman, M.; Chow, N. A. *Candida auris* whole-genome sequence benchmark dataset for phylogenomic pipelines. *J. Fungi*, **2021**, *7(3)*, 214. https://doi.org/10.3390/jof7030214
13. Takahashi-Nakaguchi, A.; Muraosa, Y.; Hagiwara, D.; Sakai, K.; Toyotome, T.; Watanabe, A.; Kawamoto, S.; Kamei, K.; Gonoi, T.; Takahashi, H. Genome sequence comparison of *Aspergillus fumigatus* strains isolated from patients with pulmonary aspergilloma and chronic necrotizing pulmonary aspergillosis. *Med. Mycol.*, **2015**, *53(4)*, 353-360. https://doi.org/10.1093/mmy/myv003
14. Kusuya, Y.; Sakai, K.; Kamei, K.; Takahashi, H.; Yaguchi, T. Draft genome sequence of the pathogenic filamentous fungus *Aspergillus lentulus* IFM 54703T. *Genome Announc.*, **2016**, *4(1)*, e01568-15. https://doi.org/10.1128/genomeA.01568-15
15. Yang, Y.; Chen, M.; Li, Z.; Al-Hatmi, A.; De Hoog, S.; Pan, W.; Ye, Q.; Bo, X.; Li, Z.; Wang, S.; Wang, J.; Chen, H.; Liao, W. Genome sequencing and comparative genomics analysis revealed pathogenic potential in *Penicillium capsulatum* as a novel fungal pathogen belonging to Eurotiales. *Front. Microbiol.*, **2016**, *7*, 1541. https://doi.org/10.3389/fmicb.2016.01541
16. Sepúlveda, V. E.; Márquez, R.; Turissini, D. A.; Goldman, W. E.; Matute, D. R. Genome sequences reveal cryptic speciation in the human pathogen *Histoplasma capsulatum*. *MBio*, **2017**, *8(6)*, e01339-17. https://doi.org/10.1128/mBio.01339-17
17. Garcia-Hermoso, D.; Criscuolo, A.; Lee, S. C.; Legrand, M.; Chaouat, M.; Denis, B.; Lafaurie, M.; Rouveau, M.; Soler, C.; Schaal, J.-V.; Mimoun, M.; Mebazaa, A.; Heitman, J.; Dromer, F.; Brisse, S.; Bretagne, S.; Alanio, A. Outbreak of invasive wound mucormycosis in a burn unit due to multiple strains of *Mucor circinelloides f. circinelloides* resolved by whole-genome sequencing. *MBio*, **2018**, *9(2)*,e00573-18. https://doi.org/10.1128/mBio.00573-18
18. Persinoti, G. F.; Martinez, D. A.; Li, W.; Döğen, A.; Billmyre, R. B.; Averette, A.; Goldberg, J. M.; Shea, T.; Young, S.; Zeng, Q.; Oliver, B. G.; Barton, R.; Metin, B.; Hilmioğlu-Polat, S.; Ilkit, M.; Gräser, Y.; Martinez-Rossi, N. M.; White, T. C.; Heitman, J.; Cuomo, C. A. Whole-genome analysis illustrates global clonal population structure of the ubiquitous dermatophyte pathogen *Trichophyton rubrum*. *Genetics*, **2018**, *208(4)*, 1657-1669. https://doi.org/10.1534/genetics.117.300573
19. Mixão, V.; Hansen, A. P.; Saus, E.; Boekhout, T.; Lass-Florl, C.; Gabaldón, T. Whole-genome sequencing of the opportunistic yeast pathogen *Candida inconspicua* uncovers its hybrid origin. *Frontiers Genet.*, **2019**, *10*, 383. https://doi.org/10.3389/fgene.2019.00383
20. Palanivel, M.; Mac Aogáin, M.; Purbojati, R. W.; Uchida, A.; Aung, N. W.; Lim, S. B.; Putra, A.; Drautz-Moses, D. I.; Seaton, S.; Rogers, T. R.; Schuster S. C.; Chotirmall, S. H. Whole-genome sequencing of *Aspergillus terreus* species complex. *Mycopathologia*, **2020**, *185(2)*, 405-408. https://doi.org/10.1007/s11046-020-00433-0
21. Ojeda, D. I.; Dhillon, B.; Tsui, C. K.; Hamelin, R. C. Single-nucleotide polymorphism discovery in *Leptographium longiclavatum*, a mountain pine beetle-associated symbiotic fungus, using whole-genome resequencing. *Mol. Ecol. Res.*, **2014**, *14(2)*, 401-410. https://doi.org/10.1111/1755-0998.12191
22. Pattemore, J. A.; Hane, J. K.; Williams, A. H.; Wilson, B. A.; Stodart, B. J.; Ash, G. J. The genome sequence of the biocontrol fungus *Metarhizium anisopliae* and comparative genomics of *Metarhizium* species. *BMC Genom.*, **2014**, *15(1)*, 1-15. https://doi.org/10.1186/1471-2164-15-660
23. Prasad, P.; Varshney, D.; Adholeya, A. Whole genome annotation and comparative genomic analyses of bio-control fungus *Purpureocillium lilacinum*. *BMC Genom.*, **2015**, *16(1)*, 1-14. https://doi.org/10.1186/s12864-015-2229-2
24. Agrawal, Y.; Narwani, T.; Subramanian, S. Genome sequence and comparative analysis of clavicipitaceous insect-pathogenic fungus *Aschersonia badia* with *Metarhizium* spp. *BMC Genom.*, **2016**, *17(1)*, 1-15. https://doi.org/10.1186/s12864-016-2710-6
25. Valero-Jiménez, C. A.; Wiegers, H.; Zwaan, B. J.; Koenraadt, C. J.; van Kan, J. A. Genes involved in virulence of the entomopathogenic fungus *Beauveria bassiana*. *J. Invertebr. Pathol.*, **2016**, *133*, 41-49. https://doi.org/10.1016/j.jip.2015.11.011
26. Haelewaters, D.; Okrasińska, A.; Gorczak, M.; Pfister, D. H. Draft genome sequence of the globally distributed cockroach-infecting fungus *Herpomyces periplanetae* strain D. Haelew. 1187d. *Microbiol. Res. Announc.*, **2020**, *9(6)*, e01458-19. https://doi.org/10.1128/MRA.01458-19
27. Tian, X.; Sun, T.; Lin, R.; Liu, R.; Yang, Y.; Xie, B.; Mao, Z. Genome sequence resource of *Sarocladium terricola* TR, an endophytic fungus as a potential biocontrol agent against *Meloidogyne incognita*. *Mol. Plant-Microbe Interact.*, **2022**, *35(6)*. https://doi.org/10.1094/MPMI-11-21-0284-A

28. Chibucos, M. C.; Crabtree, J.; Nagaraj, S.; Chaturvedi, S.; Chaturvedi, V. Draft genome sequences of human pathogenic fungus *Geomyces pannorum sensu lato* and bat white nose syndrome pathogen *Geomyces* (*Pseudogymnoascus*) *destructans*. *Genome Announc.*, **2013**, *1(6)*, e01045-13. https://doi.org/10.1128/genomeA.01045-13
29. Kusuya, Y.; Takahashi-Nakaguchi, A.; Takahashi, H.; Yaguchi, T. Draft genome sequence of the pathogenic filamentous fungus *Aspergillus udagawae* strain IFM 46973T. *Genome Announc.*, **2015**, *3(4)*, e00834-15. https://doi.org/10.1128/genomeA.00834-15
30. Triana, S.; González, A.; Ohm, R. A.; Wösten, H. A.; de Cock, H.; Restrepo, S.; Celis, A. Draft genome sequence of the animal and human pathogen *Malassezia pachydermatis* strain CBS 1879. *Genome Announc.*, **2015**, *3(5)*, e01197-15. https://doi.org/10.1128/genomeA.01197-15
31. Luo, R.; Zimin, A.; Workman, R.; Fan, Y.; Pertea, G.; Grossman, N.; Wear, M. P.; Jia, B.; Miller, H.; Casadevall, A.; Timp, W.; Zhang, S. X.; Salzberg, S. L. First draft genome sequence of the pathogenic fungus *Lomentospora prolificans* (formerly *Scedosporium prolificans*). *G3*, **2017**, *7(11)*, 3831-3836
32. Blanco-Ulate, B.; Rolshausen, P. E.; Cantu, D. Draft genome sequence of the grapevine dieback fungus *Eutypa lata* UCR-EL1. *Genome Announc.*, **2013**, *1(3)*, e00228-13. https://doi.org/10.1128/genomeA.00228-13
33. Blanco-Ulate, B.; Rolshausen, P.; Cantu, D. Draft genome sequence of *Neofusicoccum parvum* isolate UCR-NP2, a fungal vascular pathogen associated with grapevine cankers. *Genome Announc.*, **2013**, *1(3)*, e00339-13. https://doi.org/10.1128/genomeA.00339-13
34. Wibberg, D.; Jelonek, L.; Rupp, O.; Hennig, M.; Eikmeyer, F.; Goesmann, A.; Hartmann, A.; Borriss, R.; Grosch, R.; Pühler, A.; Schlüter, A. Establishment and interpretation of the genome sequence of the phytopathogenic fungus *Rhizoctonia solani* AG1-IB isolate 7/3/14. *J. Biotechnol.*, **2013**, *167(2)*, 142-155. https://doi.org/10.1016/j.jbiotec.2012.12.010
35. Cubeta, M. A.; Thomas, E.; Dean, R. A.; Jabaji, S.; Neate, S. M.; Tavantzis, S.; Toda, T.; Vilgalys, R.; Bharathan, N.; Fedorova-Abrams, N.; Pakala, S. B.; Pakala, S. M.; Zafar, N.; Joardar, V.; Losada, L.; Nierman, W. C. Draft genome sequence of the plant-pathogenic soil fungus *Rhizoctonia solani* anastomosis group 3 strain Rhs1AP. *Genome Announc.*, **2014**, *2(5)*, e01072-14. https://doi.org/10.1128/genomeA.01072-14
36. Que, Y.; Xu, L.; Wu, Q.; Liu, Y.; Ling, H.; Liu, Y.; Zhang, Y.; Guo, J.; Su, Y.; Chen, J.; Wang, S. ;Zhang, C. Genome sequencing of *Sporisorium scitamineum* provides insights into the pathogenic mechanisms of sugarcane smut. *BMC Genom.*, 15(1), 2014, 1-20. https://doi.org/10.1186/1471-2164-15-996
37. Srivastava, S. K.; Huang, X.; Brar, H. K.; Fakhoury, A. M.; Bluhm, B. H.; Bhattacharyya, M. K. The genome sequence of the fungal pathogen *Fusarium virguliforme* that causes sudden death syndrome in soybean. *PloS One*, **2014**, *9(1)*, e81832. https://doi.org/10.1371/journal.pone.0081832
38. van der Nest, M. A.; Bihon, W.; De Vos, L.; Naidoo, K.; Roodt, D.; Rubagotti, E.; Slippers, B.; Steenkamp, E. T.; Wilken, P. M.; Wilson, A.; Wingfield, M. J.; Wingfield, B. D. Draft genome sequences of *Diplodia sapinea*, *Ceratocystis manginecans*, and *Ceratocystis moniliformis*. *IMA fungus*, **2014**, *5(1)*, 135-140. https://doi.org/10.5598/imafungus.2014.05.01.13
39. Baroncelli, R.; Piaggeschi, G.; Fiorini, L.; Bertolini, E.; Zapparata, A.; Pè, M. E.; Sarrocco, S.; Vannacci, G. Draft whole-genome sequence of the biocontrol agent *Trichoderma harzianum* T6776. *Genome Announc.*, **2015**,*3(3)*, e00647-15. Draft whole-genome sequence of the biocontrol agent Trichoderma harzianum T6776
40. Deng, C. H.; Scheper, R. W.; Thrimawithana, A. H.; Bowen, J. K. Draft genome sequences of two isolates of the plant-pathogenic fungus *Neonectria ditissima* that differ in virulence. *Genome Announc.*, **2015**, 3(6), e01348-15. https://doi.org/10.1128/genomeA.01348-15
41. Gao, R.; Cheng, Y.; Wang, Y.; Wang, Y.; Guo, L.; Zhang, G. Genome sequence of *Phytophthora fragariae var. fragariae*, a quarantine plant-pathogenic fungus. *Genome Announc.*, **2015**, *3(2)*, e00034-15. https://doi.org/10.1128/genomeA.00034-15
42. King, R.; Urban, M.; Hammond-Kosack, M. C.; Hassani-Pak, K.; Hammond-Kosack, K. E. The completed genome sequence of the pathogenic ascomycete fungus *Fusarium graminearum*. *BMC Genom.*, **2015**, *16(1)*, 1-21. https://doi.org/10.1186/s12864-015-1756-1
43. Malapi-Wight, M.; Salgado-Salazar, C.; Demers, J.; Veltri, D.; Crouch, J. A. Draft genome sequence of *Dactylonectria macrodidyma*, a plant-pathogenic fungus in the Nectriaceae. *Genome Announc.*, **2015**, *3(2)*, e00278-15. https://doi.org/10.1128/genomeA.00278-15
44. Okagaki, L. H.; Nunes, C. C.; Sailsbery, J.; Clay, B.; Brown, D.; John, T.; Oh, Y.; Young, N.; Fitzgerald, M.; Haas, B. J; Zeng, Q.; Young, S.; Adiconis, X.; Fan, L.; Levin, J. Z.; Mitchell, T. K.; Okubara, P. A.; Farman, M. L.; Kohn, L. M.; Birren, B.; Ma, L.-J.;Dean, R. A. Genome sequences of three phytopathogenic species of the Magnaporthaceae family of fungi. *G3*, **2015**, *5(12)*, 2539-2545. https://doi.org/10.1534/g3.115.020057
45. Taniguti, L. M.; Schaker, P. D.; Benevenuto, J.; Peters, L. P.; Carvalho, G.; Palhares, A.; Quecine, M. C.; Nunes, F. R. S.; Kmit, M. C. P.; Wai, A.; Hausner, G.; Aitken, K. S.; Berkman, P. J.; Fraser, J. A.; Moolhuijzen, P. M.; Coutinho, L. L.; Creste, S.; Vieira, M. L. C.; Kitajima, J. P.; Monteiro-Vitorello, C. B. Complete genome sequence of *Sporisorium scitamineum* and biotrophic interaction transcriptome with sugarcane. *PloS One*, **2015**, *10(6)*, e0129318. https://doi.org/10.1371/journal.pone.0129318
46. Wingfield, B. D.; Barnes, I.; de Beer, Z. W.; De Vos, L.; Duong, T. A.; Kanzi, A. M.; Naidoo, K.; Nguyen, H. D. T.; Santana, Q. C.; Sayari, M.; Seifert, K. A.; Steenkamp, E. T.; Trollip, C.; van der Merwe, N. A.; van der Nest, M. A.; Wilken, P. M. ; Wingfield, M. J. Draft genome sequences of *Ceratocystis eucalypticola*, *Chrysoporthe cubensis*, *C. deuterocubensis*, *Davidsoniella virescens*, *Fusarium temperatum*, *Graphilbum fragrans*, *Penicillium nordicum*, and *Thielaviopsis musarum*. *IMA fungus*, **2015**, *6(2)*, 493-506. https://doi.org/10.5598/imafungus.2015.06.02.13

47. Pavlov, A. R.; Tyazhelova, T. V.; Moiseenko, K. V.; Vasina, D. V.; Mosunova, O. V.; Fedorova, T. V.; Maloshenok, L. G. ; Landesman, E. O.; Bruskin, S. A.; Psurtseva, N. V.; Slesarev, A. I.; Kozyavkin, S. A.; Koroleva, O. V. Draft genome sequence of the fungus *Trametes hirsuta* 072. *Genome Announc.*, **2015**, *3(6)*, e01287-15. https://doi.org/10.5598/imafungus.2015.06.02.13
48. Taniguti, L. M., Schaker, P. D., Benevenuto, J., Peters, L. P., Carvalho, G., Palhares, A.; Quecine, M. C.; Nunes, F. R. S.; Kmit, M. C. P.; Wai, A.; Hausner, G.; Aitken, K. S.; Berkman, P. J.; Fraser, J. A.; Moolhuijzen, P. M.; Coutinho, L. L.; Silvana Creste, L. C. Vieira, João P. Kitajima, Monteiro-Vitorello, C. B. Complete genome sequence of *Sporisorium scitamineum* and biotrophic interaction transcriptome with sugarcane. *PloS One*, **2015**, *10(6)*, e0129318. https://doi.org/10.1371/journal.pone.0129318
49. Wingfield, B. D.; Duong, T. A.; Hammerbacher, A.; van der Nest, M. A.; Wilson, A.; Chang, R.; de Beer, Z. W.; Steenkamp, E. T.; Wilken, P. M.; Naidoo, K.; Wingfield, M. J. Draft genome sequences for *Ceratocystis fagacearum*, *C. harringtonii*, *Grosmannia penicillata*, and *Huntiella bhutanensis*. *IMA fungus*, **2016**, *7(2)*, 317-323. https://doi.org/10.5598/imafungus.2016.07.02.11
50. Li, S.; Song, Q.; Martins, A. M.; Cregan, P. Draft genome sequence of *Diaporthe aspalathi* isolate MS-SSC91, a fungus causing stem canker in soybean. *Genom. Data*, **2016**, *7*, 262-263. https://doi.org/10.1016/j.gdata.2016.02.002
51. Malapi-Wight, M.; Salgado-Salazar, C.; Demers, J. E.; Clement, D. L.; Rane, K. K.; Crouch, J. A. Sarcococca blight: use of whole-genome sequencing for fungal plant disease diagnosis. *Plant Dis.*, **2016**, *100(6)*, 1093-1100. https://doi.org/10.1094/PDIS-10-15-1159-RE
52. Walkowiak, S.; Rowland, O.; Rodrigue, N.; Subramaniam, R. Whole genome sequencing and comparative genomics of closely related *Fusarium* head blight fungi: *Fusarium graminearum*, *F. meridionale* and *F. asiaticum*. *BMC Genom.*, **2016**, *17(1)*, 1-15. https://doi.org/10.1186/s12864-016-3371-1
53. Gaskell, J.; Kersten, P.; Larrondo, L. F.; Canessa, P.; Martinez, D.; Hibbett, D.; Schmoll, M.; Kubicekg, C. P.; Martinez, A. T.; Yadavi, J.; Master, E.; Magnuson, J. K.; Yaver, D.; Berka, R.; Lail, K.; Chen, C.; La Butti, K.; Nolan, M.; Lipzen, A.; Aerts, A.; Riley, R.; Barry, K.; Henrissat, B.; Blanchette, R.; Grigoriev, I. V.; Cullen, D. Draft genome sequence of a monokaryotic model brown-rot fungus *Postia* (*Rhodonia*) placenta SB12. *Genom. Data*, **2017**, *14*, 21-23. https://doi.org/10.1016/j.gdata.2017.08.003
54. Hong, C. Y.; Lee, S. Y.; Ryu, S. H.; Kim, M. Whole-genome *de novo* sequencing of wood rot fungus *Fomitopsis palustris* (ATCC62978) with both a cellulolytic and ligninolytic enzyme system. *J. Biotechnol.*, **2017**, *251*, 156-159. https://doi.org/10.1016/j.jbiotec.2017.04.009
55. Iquebal, M. A.; Tomar, R. S.; Parakhia, M. V.; Singla, D.; Jaiswal, S.; Rathod, V. M.; Padhiyar, S. M.; Kumar, N.; Rai, A.; Kumar, D. Draft whole genome sequence of groundnut stem rot fungus *Athelia rolfsii* revealing genetic architect of its pathogenicity and virulence. *Sci. Rep.*, **2017**, *7(1)*, 1-10. https://doi.org/10.1038/s41598-017-05478-8
56. Derbyshire, M.; Denton-Giles, M.; Hegedus, D.; Seifbarghy, S.; Rollins, J.; van Kan, J.; Seidl, M. F.; Faino, L.; Mbengue, M.; Navaud, O.; Raffaele, S.; Hammond-Kosack, K.; Heard, S.; Oliver, R. The complete genome sequence of the phytopathogenic fungus *Sclerotinia sclerotiorum* reveals insights into the genome architecture of broad host range pathogens. *Genome Biol. Evol.*, **2017**, *9(3)*, 593-618. https://doi.org/10.1093/gbe/evx030
57. Kumar, A.; Pandey, V.; Singh, M.; Pandey, D.; Saharan, M. S.; Marla, S. S. Draft genome sequence of karnal bunt pathogen (*Tilletia indica*) of wheat provides insights into the pathogenic mechanisms of quarantined fungus. *PloS One*, **2017**, *12(2)*, e0171323. https://doi.org/10.1371/journal.pone.0171323
58. Lee, S. Y.; An, J. E.; Ryu, S. H.; Kim, M. *De novo* whole-genome sequencing of the wood rot fungus *Polyporus brumalis*, which exhibits potential terpenoid metabolism. *Genome Announc.*, **2017**, *5(28)*, e00586-17. https://doi.org/10.1128/genomeA.00586-17
59. Neu, E.; Featherston, J.; Rees, J.; Debener, T. A draft genome sequence of the rose black spot fungus *Diplocarpon rosae* reveals a high degree of genome duplication. *PloS One*, **2017**, *12(10)*, e0185310. https://doi.org/10.1371/journal.pone.0185310
60. Rao, S.; Nandineni, M. R. Genome sequencing and comparative genomics reveal a repertoire of putative pathogenicity genes in chilli anthracnose fungus *Colletotrichum truncatum*. *PLoS One*, **2017**, *12(8)*, e0183567. https://doi.org/10.1371/journal.pone.0183567
61. Rivera, Y.; Zeller, K.; Srivastava, S.; Sutherland, J.; Galvez, M.; Nakhla, M.; Poniatowska, A.; Schnabel, G.; Sundin, G.; Abad, Z. G. Draft genome resources for the phytopathogenic fungi *Monilinia fructicola*, *M. fructigena*, *M. polystroma*, and *M. laxa*, the causal agents of brown rot. *Phytopathology*, **2018**, *108(10)*, 1141-1142. https://doi.org/10.1094/PHYTO-12-17-0418-A
62. Utomo, C.; Tanjung, Z. A.; Aditama, R.; Buana, R. F. N.; Pratomo, A. D. M.; Tryono, R.; Liwang, T. Draft genome sequence of the phytopathogenic fungus *Ganoderma boninense*, the causal agent of basal stem rot disease on oil palm. *Genome Announc.*, **2018**, *6(17)*, e00122-18. https://doi.org/10.1128/genomeA.00122-18
63. Wolters, P. J.; Faino, L.; Van Den Bosch, T. B.; Evenhuis, B.; Visser, R. G.; Seidl, M. F.; Vleeshouwers, V. G. Gapless genome assembly of the potato and tomato early blight pathogen *Alternaria solani*. *Mol. Plant-Microbe Interact.*, **2018**, *31(7)*, 692-694. https://doi.org/10.1094/MPMI-12-17-0309-A
64. Le Cam, B.; Sargent, D.; Gouzy, J.; Amselem, J.; Bellanger, M. N.; Bouchez, O.; Brown, S.; Caffier, V.; De Gracia, M.; Debuchy, R.; Duvaux, L.; Payen, T.; Sannier, M.; Shiller, J.; Collemare, J.; Lemaire, C. Population genome sequencing of the scab fungal species *Venturia inaequalis*, *Venturia pirina*, *Venturia aucupariae* and *Venturia asperata*. *G3*, **2019**, *9(8)*, 2405-2414. https://doi.org/10.1534/g3.119.400047
65. Ren, Y.; Li, D.; Zhao, X.; Wang, Y.; Bao, X.; Wang, X.; Wu, X.; Wang, D.; Song, B.; Chen, Z. Whole genome sequences of the tea leaf spot pathogen *Didymella segeticola*. *Phytopathology*, **2019**, *109(10)*, 1676-1678. https://doi.org/10.1094/PHYTO-02-19-0050-A
66. Baroncelli, R.; Da Lio D.; Vannacci, G.; Sarrocco, S. Genome resources for the endophytic fungus *Paraphaeosphaeria sporulosa*. *Mol. Plant-Microbe Interac.*, **2020**, *33(9)*, 1098-1099. https://doi.org/10.1094/MPMI-04-20-0097-A

67. Crouch, J. A., Dawe, A., Aerts, A., Barry, K., Churchill, A. C., Grimwood, J., Hillman, B. I.; Milgroom, M. G.; Pangilinan, J.; Smith, M.; Salamov, A.; Schmutz, J.; Yadav, J. S.; Grigoriev, I. V.; Nuss, D. L. Genome sequence of the chestnut blight fungus *Cryphonectria parasitica* EP155: a fundamental resource for an archetypical invasive plant pathogen. *Phytopathology*, **2020**, *110(6)*, 1180-1188. https://doi.org/10.1094/PHYTO-12-19-0478-A
68. Fang, X.; Qin, K.; Li, S.; Han, S.; Zhu, T. Whole genome sequence of *Diaporthe capsici*, a new pathogen of walnut blight. *Genomics*, **2020**, *112(5)*, 3751-3761. https://doi.org/10.1016/j.ygeno.2020.04.018
69. 69.Xia, C.; Lei, Y.; Wang, M.; Chen, W.; Chen, X. An avirulence gene cluster in the wheat stripe rust pathogen (*Puccinia striiformis f. sp. tritici*) identified through genetic mapping and whole-genome sequencing of a sexual population. *Msphere*, **2020**, *5(3)*, e00128-20. https://doi.org/10.1128/mSphere.00128-20
70. Zhao, Q.; Wu, J.; Zhang, L.; Yan, C.; Jiang, S.; Li, Z.; Sun, D.; Lai, Y.; Gong, Z. Genome-scale analyses and characteristics of putative pathogenicity genes of *Stagonosporopsis cucurbitacearum*, a pumpkin gummy stem blight fungus. *Sci. Rep.*, **2020**, *10(1)*, 1-15. https://doi.org/10.1038/s41598-020-75235-x
71. Baroncelli, R.; Pensec, F.; Da Lio, D.; Boufleur, T.; Vicente, I.; Sarrocco, S.; Picot, A.; Baraldi, E.; Sukno, S.; Thon, M.; Le Floch, G. Complete genome sequence of the plant-pathogenic fungus *Colletotrichum lupini*. *Mol. Plant Microbe Interact.*, **2021**, *34(12)*, 1461-1464. https://doi.org/10.1094/MPMI-07-21-0173-A
72. Kim, D. W.; Nam, J.; Nguyen, H. T. K.; Lee, J.; Choi, Y.; Choi, J. Draft genome sequence of the white-rot fungus *Schizophyllum commune* IUM1114-SS01. *Mycobiology*, **2021**, *49(1)*, 86-88. https://doi.org/10.1080/12298093.2020.1843222
73. Li, W. C.; Lin, T. C.; Chen, C. L.; Liu, H. C.; Lin, H. N.; Chao, J. L.; Hsieh, C.-H.; Ni, H.-F.; Chen, R.-S.; Wang, T. F. Complete genome sequences and genome-wide characterization of *Trichoderma* biocontrol agents provide new insights into their evolution and variation in genome organization, sexual development, and fungal-plant interactions. *Microbiol. Spectr.*, **2021**, *9(3)*, e00663-21. https://doi.org/10.1128/Spectrum.00663-21
74. Liu, H. H.; Wang, J.; Wu, P. H.; Lu, M. Y. J.; Li, J. Y.; Shen, Y. M.; Tzeng, M.-N.; Kuo, C.-H.; Lin, Y.-H.; Chang, H. X. Whole-genome sequence resource of *Calonectria ilicicola*, the casual pathogen of soybean red crown rot. *Mol. Plant-Microbe Interact.*, **2021**, *34(7)*, 848-851. https://doi.org/10.1094/MPMI-11-20-0315-A
75. Rao, Y.,; Mei, L.; Zhang, L.; Jiang, H.; Ma, L.; Wang, Y. Genome sequence resource of *Botryosphaeria dothidea* CK16, a fungal pathogen causing Chinese hickory trunk canker disease. *Plant Dis.*, **2021**, *105(10)*, 3282-3284. https://doi.org/10.1094/PDIS-02-21-0254-A
76. Tian, Y. Z.; Wang, Z. F.; Liu, Y. D.; Zhang, G. Z.; Li, G. The whole-genome sequencing and analysis of a *Ganoderma lucidum* strain provide insights into the genetic basis of its high triterpene content. *Genomics*, **2021**, *113(1)*, 840-849. https://doi.org/10.1016/j.ygeno.2020.10.015
77. Wang, Y.; Yao, J.; Li, Z.; Huo, J.; Zhou, S.; Liu, W.; Wu, H. Genome sequence resource for *Nigrospora oryzae*, an important pathogenic fungus threatening crop production. *Mol. Plant Microbe Interact*, **2021**, *34(7)*, 835-838. https://doi.org/10.1094/MPMI-11-20-0311-A
78. Wyka, S. A.; Mondo, S. J.; Liu, M.; Dettman, J.; Nalam, V.; Broders, K. D. Whole-genome comparisons of ergot fungi reveals the divergence and evolution of species within the genus *Claviceps* are the result of varying mechanisms driving genome evolution and host range expansion. *Genome Biol. Evol.*, **2021**, *13(2)*, evaa267. https://doi.org/10.1093/gbe/evaa267
79. Liu, X.; Fang, X.; Yu, F.; Wang, S.; Zhang, Z.; Li, K.; Ye, W.; Lee, Y.-W.; Mohamed, S. R.; Dong, F.; Xu, J.; Shi, J. Improved wWhole-genome sequence of *Fusarium meridionale*, the fungal pathogen causing *Fusarium* head blight in rice. *Mol. Plant-Microbe Interact.*, **2022**, *35(1)*, 85-89. https://doi.org/10.1094/MPMI-07-21-0182-A
80. Huang, X.; Zeng, Z.; Chen, Z.; Yang, Y.; Pang, J.; Qian, Y.; Xiang, T. Whole-genome sequence resource of *Fusarium oxysporum* strain TH15, a plant growth promoting endophytic fungus isolated from *Tetrastigma hemsleyanum*. *PhytoFrontiers*, **2022**, *12*. https://doi.org/10.1094/PHYTOFR-12-21-0086-A
81. Majeedano, A. Q.; Chen, J.; Zhu, T.; Li, S.; Gishkori, Z. G. N.; Mastoi, S. M.; Wang, G. The first whole genome sequence discovery of the devastating fungus *Arthrinium rasikravindrae*. *J. Fungi*, **2022**, *8(3)*, 255. https://doi.org/10.3390/jof8030255
82. Weaver, M. A.; Mirza, N.; Mandel, J. R.; Boyette, C. D.; Brown, S. P. Whole-genome sequence and draft assembly of the biocontrol fungal pathogen *Albifimbria verrucaria* CABI-IMI 368023. *Microbiol. Res. Announc.*, **2022**, *11(1)*, e00909-21. https://doi.org/10.1128/MRA.00909-21
83. Marcet-Houben, M:, Ballester, A. R.; de la Fuente, B.; Harries, E.; Marcos, J. F.; González-Candelas, L.; Gabaldón, T. Genome sequence of the necrotrophic fungus *Penicillium digitatum*, the main postharvest pathogen of citrus. *BMC Genom.*, **2012**, *13(1)*, 1-18. https://doi.org/10.1186/1471-2164-13-646
84. Morin, E.; Kohler, A.; Baker, A. R.; Foulongne-Oriol, M.; Lombard, V.; Nagye, L. G.; Ohm, R. A.; Patyshakuliyeva, A.; Brun, A.; Aerts, A. L.; Bailey, A. M.; Billette, C.; Coutinho, P. M.; Deakin, G.; Doddapaneni, H.; Floudas, D.; Grimwood, J.; Hildén, K.; Kües, U.; LaButti, K. M.; Lapidus, A.; Lindquist, E. A.; Lucas, S. M.; Murat, C.; Riley, R. W.; Salamov, A. A.; Schmutz, J.; Subramanian, V.; Wösten, H. A. B.; Xu, J.; Eastwood, D. C.; Foster, G. D.; Sonnenberg, A. S. M.; Cullen, D.; de Vries, R. P.; Lundell, T.; Hibbett, D. S.; Henrissat, B.; Burton, K. S.; Kerrigan, R. W.; Challen, M. P.; Grigoriev, I. V.; Martin, F. Genome sequence of the button mushroom *Agaricus bisporus* reveals mechanisms governing adaptation to a humic-rich ecological niche. *PNAS*, **2012**, *109(43)*, 17501-17506. https://doi.org/10.1073/pnas.1206847109
85. Bao, D.; Gong, M.; Zheng, H.; Chen, M.; Zhang, L.; Wang, H.; Jiang, J.; Wu, L.; Zhu, Y.; Zhu, G.; Zhou, Y.; Li, C.; Wang, S.; Zhao, Y.; Zhao, G.; Tan, Q. Sequencing and comparative analysis of the straw mushroom (*Volvariella volvacea*) genome. *PloS One*, **2013**, *8(3)*, e58294. https://doi.org/10.1371/journal.pone.0058294

86. Crauwels, S.; Zhu, B.; Steensels, J.; Busschaert, P.; De Samblanx, G.; Marchal, K.; Willems, K. A.; Verstrepen, K. J.; Lievens, B. Assessing genetic diversity among *Brettanomyces* yeasts by DNA fingerprinting and whole-genome sequencing. *App. Environm. Microbiol.*, **2014**, *80(14)*, 4398-4413. https://doi.org/10.1128/AEM.00601-14
87. Moore, G. G.; Mack, B. M.; Beltz, S. B. Genomic sequence of the aflatoxigenic filamentous fungus *Aspergillus nomius*. *BMC Genom.*, **2015**, *16(1)*, 1-10. https://doi.org/10.1186/s12864-015-1719-6
88. Nierman, W. C.; Yu, J.; Fedorova-Abrams, N. D.; Losada, L.; Cleveland, T. E.; Bhatnagar, D.; Bennett, J. W.; Dean, R.; Payne, G. A. Genome sequence of *Aspergillus flavus* NRRL 3357, a strain that causes aflatoxin contamination of food and feed. *Genome Announc.*, **2015**, *3(2)*, e00168-15. https://doi.org/10.1128/AEM.00601-14
89. Chen, L.; Gong, Y.; Cai, Y.; Liu, W.; Zhou, Y.; Xiao, Y.; Xu, Z.; Liu, Y.; Lei, X.; Wang, G.; Guo, M.; Ma, X.; Bian, Y. Genome sequence of the edible cultivated mushroom *Lentinula edodes* (Shiitake) reveals insights into lignocellulose degradation. *PloS One*, **2016**, *11(8)*, e0160336. https://doi.org/10.1371/journal.pone.0160336
90. Faustinelli, P. C.; Wang, X. M.; Palencia, E. R.; Arias, R. S. Genome sequences of eight *Aspergillus flavus spp*. and one *A. parasiticus* sp., isolated from peanut seeds in Georgia. *Genome Announc.*, **2016**, *4(2)*, e00278-16. https://doi.org/10.1128/genomeA.00278-16
91. Han, X.; Chakrabortti, A.; Zhu, J.; Liang, Z. X.; Li, J. Sequencing and functional annotation of the whole genome of the filamentous fungus *Aspergillus westerdijkiae*. *BMC Genom.*, **2016**, *17(1)*, 1-14. https://doi.org/10.1186/s12864-016-2974-x
92. Kurata, A.; Fukuta, Y.; Mori, M.; Kishimoto, N.; Shirasaka, N. Draft genome sequence of the basidiomycetous fungus *Flammulina velutipes* TR19. *Genome Announc.*, **2016**, *4(3)*, e00505-16. https://doi.org/10.1128/genomeA.00505-16
93. Schjørring, S.; Lassen, S. G.; Jensen, T.; Moura, A.; Kjeldgaard, J. S.; Müller, L.; Thielke, S; Leclercq, A.; Maury, M. M.; Tourdjman, M.; Donguy, M.-P.; Lecuit, M.; Ethelberg, S.; Nielsen, E. M. Cross-border outbreak of listeriosis caused by cold-smoked salmon, revealed by integrated surveillance and whole genome sequencing (WGS), Denmark and France, 2015 to 2017. *Eurosurveillance*, **2017**, *22(50)*, 17-00762
94. Zhang, C.; Deng, W.; Yan, W.; Li, T. Whole genome sequence of an edible and potential medicinal fungus, *Cordyceps guangdongensis*. *G3*, **2018**, *8(6)*, 1863-1870. https://doi.org/10.1534/g3.118.200287
95. Schmidt-Heydt, M.; Stoll, D.; Geisen, R. Whole-genome sequencing of the fungus *Penicillium citrinum* reveals the biosynthesis gene cluster for the mycotoxin citrinin. *Microbiol. Resour. Announc.*, **2019**, *8(4)*, e01419-18. https://doi.org/10.1128/MRA.01419-18
96. Wu, G.; Jurick II, W. M.; Lichtner, F. J.; Peng, H.; Yin, G.; Gaskins, V. L.; Yin, Y.; Hua, S.-S.; Peter, K. A.; Bennett, J. W. Whole-genome comparisons of *Penicillium* spp. reveals secondary metabolic gene clusters and candidate genes associated with fungal aggressiveness during apple fruit decay. *PeerJ*, **2019**, *7*, e6170. https://doi.org/10.7717/peerj.6170
97. Yuan, Y.; Wu, F.; Si, J.; Zhao, Y. F.; Dai, Y. C. Whole genome sequence of *Auricularia heimuer* (Basidiomycota, Fungi), the third most important cultivated mushroom worldwide. *Genomics*, **2019**, *111(1)*, 50-58. https://doi.org/10.1016/j.ygeno.2017.12.013
98. Gong, W.; Wang, Y.; Xie, C.; Zhou, Y.; Zhu, Z.; Peng, Y. Whole genome sequence of an edible and medicinal mushroom, *Hericium erinaceus* (Basidiomycota, Fungi). *Genomics*, **2020**, *112(3)*, 2393-2399. https://doi.org/10.1016/j.ygeno.2020.01.011
99. Yu, F.; Song, J.; Liang, J.; Wang, S.; Lu, J. Whole genome sequencing and genome annotation of the wild edible mushroom, *Russula griseocarnosa*. *Genomics*, **2020**, *112(1)*, 603-614. https://doi.org/10.1016/j.ygeno.2019.04.012
100. Sun, T.; Zhang, Y.; Jiang, H.; Yang, K.; Wang, S.; Wang, R.; Li, S.; Lei, P.; Xu, H.; Qiu, Y.; Sun, D. Whole genome sequencing and annotation of *Naematelia aurantialba* (Basidiomycota, edible-medicinal fungi). *J. Fungi*, **2021**, *8(1)*, 6. https://doi.org/10.3390/jof8010006
101. Li, S.: Zhao, S.; Hu, C.; Mao, C.; Guo, L.; Yu, H.; Yu, H. Whole genome sequence of an edible mushroom *Stropharia rugosoannulata* (Daqiugaigu). *J. Fungi*, **2022**, *8(2)*, 99. https://doi.org/10.3390/jof8020099
102. Semeiks, J.; Borek, D.; Otwinowski, Z.; Grishin, N. V. Comparative genome sequencing reveals chemotype-specific gene clusters in the toxigenic black mold *Stachybotrys*. *BMC Genom.*, **2014**, *15(1)*, 1-16. https://doi.org/10.1186/1471-2164-15-590
103. Abdolrasouli, A.; Rhodes, J.; Beale, M. A.; Hagen, F.; Rogers, T. R.; Chowdhary, A.; Meis, J. F.; Armstrong-James, D.; Fisher, M. C. Genomic context of azole resistance mutations in *Aspergillus fumigatus* determined using whole-genome sequencing. *MBio*, **2015**, *6(3)*, e00536-15. https://doi.org/10.1128/mBio.00536-15
104. Pizarro, D.; Dal Grande, F.; Leavitt, S. D.; Dyer, P. S.; Schmitt, I.; Crespo, A.; Lumbsch, H. T.; Divakar, P. K. Whole-genome sequence data uncover widespread heterothallism in the largest group of lichen-forming fungi. *Genome Biol. Evol.*, **2019**, *11(3)*, 721–730. https://doi.org/10.1093/gbe/evz027
105. Gilbert, M. K.; Mack, B. M.; Moore, G. G.; Downey, D. L.; Lebar, M. D.; Joardar, V.; Losada, L.; Yu, J.-J.; Nierman, W. C.; Bhatnagar, D. Whole genome comparison of *Aspergillus flavus* L-morphotype strain NRRL 3357 (type) and S-morphotype strain AF70. *PLoS One*, **2018**, *13(7)*, e0199169. https://doi.org/10.1371/journal.pone.0199169
106. Wang, Y.; Wang, L.; Wu, F.; Liu, F.; Wang, Q.; Zhang, X.; Selvaraj, J. N.; Zhao, Y.; Xing, F.; Yin, W.-B.; Liu, Y. A consensus ochratoxin A biosynthetic pathway: Insights from the genome sequence of *Aspergillus ochraceus* and a comparative genomic analysis. *App. Environ. Microbiol.*, **2018**, *84(19)*, e01009-18. https://doi.org/10.1128/AEM.01009-18
107. Pizarro, D.; Dal Grande, F.; Leavitt, S. D.; Dyer, P. S.; Schmitt, I.; Crespo, A.; Lumbsch, H. T.; Divakar, P. K. Whole-genome sequence data uncover widespread heterothallism in the largest group of lichen-forming fungi. *Genome Biol. Evol.*, **2019**, *11(3)*, 721-730. https://doi.org/10.1093/gbe/evz027
108. Puértolas-Balint, F.; Rossen, J. W.; Oliveira dos Santos, C.; Chlebowicz, M. M.; Raangs, E. C.; Van Putten, M. L.; Sola-Campoy, P. J.; Han, L.; Schmidt, M.; García-Cobos, S. Revealing the virulence potential of clinical and environmental *Aspergillus fumigatus* isolates using whole-genome sequencing. *Front. Microbiol.*, **2019**, *10*, 1970. https://doi.org/10.3389/fmicb.2019.01970

109. Park, S. Y.; Choi, J.; Kim, J. A.; Yu, N. H.; Kim, S.; Kondratyuk, S. Y.; Lee, Y.-H.; Hur, J. S. Draft genome sequence of lichen-forming fungus *Caloplaca flavorubescens* strain KoLRI002931. *Genome Announc.*, **2013**, *1(4)*, e00678-13. https://doi.org/10.1128/genomeA.00678-13
110. Park, S. Y.; Choi, J.; Kim, J. A.; Jeong, M. H.; Kim, S.; Lee, Y. H.; Hur, J. S. Draft genome sequence of *Cladonia macilenta* KoLRI003786, a lichen-forming fungus producing biruloquinone. *Genome Announc.*, **2013**, *1(5)*, e00695-13. https://doi.org/10.1128/genomeA.00695-13
111. Park, S. Y., Choi; J., Lee, G. W.; Jeong, M. H.; Kim, J. A.; Oh, S. O.; Lee, Y.-H.; Hur, J. S. Draft genome sequence of *Umbilicaria muehlenbergii* KoLRILF000956, a lichen-forming fungus amenable to genetic manipulation. *Genome Announc.*, **2014**, *2(2)*, e00357-14. https://doi.org/10.1128/genomeA.00357-14
112. Park, S. Y.; Choi, J.; Lee, G. W.; Kim, J. A.; Oh, S. O.; Jeong, M. H.; Yu, N. H.; Kim, S.; Lee, Y.-H.; Hur, J. S. Draft genome sequence of lichen-forming fungus *Cladonia metacorallifera* strain KoLRI002260. *Genome Announc.*, **2014**, *2(1)*, e01065-13. https://doi.org/10.1128/genomeA.01065-13
113. Park, S. Y.; Choi, J.; Lee, G. W.; Park, C. H.; Kim, J. A.; Oh, S. O.; Lee, Y.-H.; Hur, J. S. Draft genome sequence of *Endocarpon pusillum* strain KoLRILF000583. *Genome Announc.*, **2014**, *2(3)*,e00452-14. https://doi.org/10.1128/genomeA.00452-14
114. Armstrong, E. E.; Prost, S.; Ertz, D.; Westberg, M.; Frisch, A.; Bendiksby, M. Draft genome sequence and annotation of the lichen-forming fungus *Arthonia radiata*. *Genome Announc.*, **2018**, *6(14)*, e00281-18. https://doi.org/10.1128/genomeA.00281-18
115. Dal Grande, F.; Meiser, A.; Tzovaras, B. G.; Jürgen, O. T. T. E.; Ebersberger, I.; Schmitt, I. The draft genome of the lichen-forming fungus *Lasallia hispanica* (Frey) Sancho & A. Crespo. *Lichenol.* , **2018**, *50(3)*, 329-340. https://doi.org/10.1017/S002428291800021X
116. Wang, Y.; Yuan, X.; Chen, L.; Wang, X.; Li, C. Draft genome sequence of the lichen-forming fungus *Ramalina intermedia* strain YAF0013. *Genome Announc.*, **2018**, *6(23)*, e00478-18. https://doi.org/10.1128/genomeA.00478-18
117. Allen, J. L.; Jones, S. J.; McMullin, R. T. Draft genome sequence of the lichenized fungus *Bacidia gigantensis*. *Microbiol. Res. Announc.*, **2021**, *10(44)*, e00686-21. https://doi.org/10.1128/MRA.00686-21
118. Chen, S.; Xu, J.; Liu, C.; Zhu, Y.; Nelson, D. R.; Zhou, S.; Li, C.; Wang, L.; Guo, X.; Sun, Y.; Luo, H.; Li, Y.; Song, J.; Henrissat, B.; Levasseur, A.; Qian, J.; Li, J.; Luo, X.; Shi, L.; He, L.; Xiang, L.; Xu, X.; Niu, Y.; Li, Q.; Han, M. V.; Yan, H.; Zhang, J.; Chen, H.; Lv, A.; Wang, Z.; Liu, M.; Schwartz, D. C.; Sun, C. Genome sequence of the model medicinal mushroom *Ganoderma lucidum*. *Nat. Comm.*, **2012**, *3(1)*, 1-9. https://doi.org/10.1038/ncomms1923
119. Zheng, P.; Xia, Y.; Xiao, G.; Xiong, C.; Hu, X.; Zhang, S.; Zheng, H.; Huang, Y.; Zhou, Y.; Wang, S.; Zhao, G.-P.; Liu, X.; St Leger, R. J.; Wang, C. Genome sequence of the insect pathogenic fungus *Cordyceps militaris*, a valued traditional Chinese medicine. *Genome Biol.*, **2012**, *12(11)*, 1-22. https://doi.org/10.1186/gb-2011-12-11-r116
120. Huo, J.; Zhong, S.; Du, X.; Cao, Y.; Wang, W.; Sun, Y.; Tian, Y.; Zhu, J.; Chen, J.; Xuan, L.; Wu, C.; Li, Y. Whole-genome sequence of *Phellinus gilvus* (mulberry Sanghuang) reveals its unique medicinal values. *J. Adv. Res.*, **2020**, *24*, 325-335. https://doi.org/10.1016/j.jare.2020.04.011
121. Jiang, J. H.; Wu, S. H.; Zhou, L. W. The first whole genome sequencing of *Sanghuangporus sanghuang* provides insights into its medicinal application and evolution. *J. Fungi*, **2021**, *7(10)*, 787. https://doi.org/10.3390/jof7100787
122. Lin, W.; Shi, Y.; Jia, G.; Sun, H.; Sun, T.; Hou, D. Genome sequencing and annotation and phylogenomic analysis of the medicinal mushroom *Amauroderma rugosum*, a traditional medicinal species in the family Ganodermataceae. *Mycologia*, **2021**, *113(2)*, 268-277. https://doi.org/10.1080/00275514.2020.1851135
123. Liu, Y.; Huang, L.; Hu, H.; Cai, M.; Liang, X.; Li, X.; Zhang, Z.; Xie, Y.; Xiao, C.; Chen, S.; Chen, D.; Yong, T.; Pan, H.; Gao, X.; Wu, Q. Whole-genome assembly of *Ganoderma leucocontextum* (Ganodermataceae, Fungi) discovered from the Tibetan Plateau of China. *G3*, **2021**, *11(12)*, jkab337. https://doi.org/10.1093/g3journal/jkab337
124. Long, L.; Liu, Z.; Deng, C.; Li, C.; Wu, L.; Hou, B.; Lin, Q. Genomic sequence and transcriptome analysis of the medicinal fungus *Keithomyces neogunnii*. *Genome Biol. Evol.*, **2022**, *(3)*, evac033. https://doi.org/10.1093/gbe/evac033
125. Lenassi, M.; Gostinčar, C.; Jackman, S.; Turk, M.; Sadowski, I.; Nislow, C.; Jones, S.; Birol, I.; Cimerman, N. G.; Plemenitaš, A. Whole genome duplication and enrichment of metal cation transporters revealed by *de novo* genome sequencing of extremely halotolerant black yeast *Hortaea werneckii*. *PLoS One*, **2013**, *8(8)*, e71328. https://doi.org/10.1371/journal.pone.0071328
126. Zajc, J.; Liu, Y.; Dai, W.; Yang, Z.; Hu, J.; Gostinčar, C.; Gunde-Cimerman, N. Genome and transcriptome sequencing of the halophilic fungus *Wallemia ichthyophaga*: haloadaptations present and absent. *BMC Genom.*, **2013**, *14(1)*, 1-21. https://doi.org/10.1186/1471-2164-14-617
127. Zhou, P.; Zhang, G.; Chen, S.; Jiang, Z.; Tang, Y.; Henrissat, B.; Yan, Q.; Yang, S.; Chen, C.-F.; Zhang, B.; Du, Z. Genome sequence and transcriptome analyses of the thermophilic zygomycete fungus *Rhizomucor miehei*. *BMC Genom.*, **2014**, *15(1)*, 1-13. https://doi.org/10.1186/1471-2164-15-294
128. Sterflinger, K.; Lopandic, K.; Pandey, R. V.; Blasi, B.; Kriegner, A. Nothing special in the specialist? Draft genome sequence of *Cryomyces antarcticus*, the most extremophilic fungus from Antarctica. *PLoS One*, **2014**, *9(10)*, e109908. https://doi.org/10.1371/journal.pone.0109908
129. Coleine, C.; Masonjones, S.; Selbmann, L.; Zucconi, L.; Onofri, S.; Pacelli, C.; Stajich, J. E. Draft genome sequences of the Antarctic endolithic fungi *Rachicladosporium antarcticum* CCFEE 5527 and *Rachicladosporium* sp. CCFEE 5018. *Genome Announc.*, **2017**, *5(27)*, e00397-17. https://doi.org/10.1128/genomeA.00397-17
130. Singh, N. K.,; Blachowicz, A.; Romsdahl, J.; Wang, C.; Torok, T.; Venkateswaran, K. Draft genome sequences of several fungal strains selected for exposure to microgravity at the International Space Station. *Genome Announc.*, **2017**, *5(15)*, e01602-16. https://doi.org/10.1128/genomeA.01602-16

131. Arora, P.; Singh, P.; Wang, Y.; Yadav, A.; Pawar, K.; Singh, A.; Padmavati, G.; Xu, J.; Chowdhary, A. Environmental isolation of *Candida auris* from the coastal wetlands of Andaman Islands, India. *MBio*, **2021**, *12(2)*, e03181-20. https://doi.org/10.1128/mBio.03181-20
132. Shi-Kunne, X.; Seidl, M. F.; Faino, L.; Thomma, B. P. Draft genome sequence of a strain of cosmopolitan fungus *Trichoderma atroviride*. *Genome Announc.*, **2015**, *3(3)*, e00287-15. https://doi.org/10.1128/genomeA.00287-15
133. Masclaux, F. G.; Wyss, T.; Pagni, M.; Rosikiewicz, P.; Sanders, I. R. Investigating unexplained genetic variation and its expression in the arbuscular mycorrhizal fungus *Rhizophagus irregularis*: a comparison of whole genome and RAD sequencing data. *PloS One*, **2019**, *14(12)*, e0226497. https://doi.org/10.1371/journal.pone.0226497
134. Liu, Y.; Hu, H.; Cai, M.; Liang, X.; Wu, X.; Wang, A; Chen, X.; Li, X.; Xiao, C.; Huang, L.; Xie, Y.; Wu, Q. Whole genome sequencing of an edible and medicinal mushroom, *Russula griseocarnosa*, and its association with mycorrhizal characteristics. *Gene*, **2022**, *808*, 145996. https://doi.org/10.1016/j.gene.2021.145996
135. Mao, J.; Guan, W. Fungal degradation of polycyclic aromatic hydrocarbons (PAHs) by *Scopulariopsis brevicaulis* and its application in bioremediation of PAH-contaminated soil. *Acta Agric. Scand. B Soil Plant Sci.*, **2016**, *66(5)*, 399-405. https://doi.org/10.1080/09064710.2015.1137629
136. See-Too, W. S.; Ee, R.; Lim, Y. L.; Convey, P.; Pearce, D. A.; Mohidin, T. B. M.; Yin, W.-F.; Chan, K. G. Complete genome of *Arthrobacter alpinus* strain R3. 8, bioremediation potential unraveled with genomic analysis. *Stand. Genom. Sci.*, **2017**, *12(1)*, 1-7. https://doi.org/10.1186/s40793-017-0264-0
137. Pandey, R. K.; Tewari, S.; Tewari, L. Lignolytic mushroom *Lenzites elegans* WDP2: Laccase production, characterization, and bioremediation of synthetic dyes. *Ecotoxicol. Environ. Saf.*, **2018**, *158*, 50-58. https://doi.org/10.1186/s40793-017-0264-0
138. Tkavc, R.; Matrosova, V. Y.; Grichenko, O. E.; Gostinčar, C.; Volpe, R. P.; Klimenkova, P.; Gaidamakova, E. K.; Zhou, C. E.; Stewart, B. J.; Lyman, M. G.; Malfatti, S. A.; Rubinfeld, B.; Courtot, M.; Singh, J.; Dalgard, C. L.; Hamilton, T.; Frey, K. G.; Gunde-Cimerman, N.; Dugan, L.; Daly, M. J. Prospects for fungal bioremediation of acidic radioactive waste sites: characterization and genome sequence of *Rhodotorula taiwanensis* MD1149. *Front. Microbiol.*, **2018**, *8*, 2528. https://doi.org/10.3389/fmicb.2017.02528
139. Su, X.; Zhou, M.; Hu, P.; Xiao, Y.; Wang, Z.; Mei, R.; Hashmi, M. Z.; Lin, H.; Chen, J.; Sun, F. Whole-genome sequencing of an acidophilic *Rhodotorula* sp. ZM1 and its phenol-degrading capability under acidic conditions. *Chemosphere*, **2019**, 232, 76-86. https://doi.org/10.1016/j.chemosphere.2019.05.195
140. Tesei, D.; Tafer, H.; Poyntner, C.; Piñar, G.; Lopandic, K.; Sterflinger, K. Draft genome sequences of the black rock fungus *Knufia petricola* and its spontaneous nonmelanized mutant. *Genome Announc.*, **2017**, *5(44)*, e01242-17. https://doi.org/10.1128/genomeA.01242-17
141. Mchunu, N. P.; Permaul, K.; Abdul Rahman, A. Y.; Saito, J. A.; Singh, S.; Alam, M. Xylanase superproducer: genome sequence of a compost-loving thermophilic fungus, *Thermomyces lanuginosus* strain SSBP. *Genome Announc.*, **2013**, *1(3)*, e00388-13. https://doi.org/10.1128/genomeA.00388-13
142. Park, Y. J.; Baek, J. H.; Lee, S.; Kim, C.; Rhee, H.; Kim, H.; Seo, J.-S.; Park, H.-R.; Yoon, D.-E.; Nam, J.-Y.; Kim, H.-I.; Kim, J.-G.; Yoon, H., Kang, H.-W.; Cho, J.-Y.; Song, E.-S.; Sung, G.-H.; Yoo, Y.-B.; Lee, C.-S.; Lee, B.-M.; Kong, W. S. Whole genome and global gene expression analyses of the model mushroom *Flammulina velutipes* reveal a high capacity for lignocellulose degradation. *PloS One*, **2014**, *9(4)*, e93560. https://doi.org/10.1371/journal.pone.0093560
143. Cuomo, C. A.; Untereiner, W. A.; Ma, L. J.; Grabherr, M.; Birren, B. W. Draft genome sequence of the cellulolytic fungus *Chaetomium globosum*. *Genome Announc.*, **2015**, *3(1)*, e00021-15. https://doi.org/10.1128/genomeA.00021-15
144. Youssar, L.; Grüning, B. A.; Erxleben, A.; Günther, S.; Hüttel, W. Genome sequence of the fungus *Glarea lozoyensis*: the first genome sequence of a species from the Helotiaceae family. *Eukaryotic Cell*, **2012**, *11(2)*. https://doi.org/10.1128/EC.05302-11
145. Chen, L.; Yue, Q.; Zhang, X.; Xiang, M.; Wang, C.; Li, S.; Che, Y.; Ortiz-López, F. J.; Bills, G. F.; Liu, X.; An, Z. Genomics-driven discovery of the pneumocandin biosynthetic gene cluster in the fungus *Glarea lozoyensis*. *BMC Genom.*, **2013**, *14(1)*, 1-18. https://doi.org/10.1186/1471-2164-14-339
146. Lorenz, S.; Guenther, M.; Grumaz, C.; Rupp, S.; Zibek, S.; Sohn, K. Genome sequence of the basidiomycetous fungus *Pseudozyma aphidis* DSM70725, an efficient producer of biosurfactant mannosylerythritol lipids. *Genome Announc.*, **2014**, *2(1)*, e00053-14. https://doi.org/10.1128/genomeA.00053-14
147. Takeda, I.; Tamano, K.; Yamane, N.; Ishii, T.; Miura, A.; Umemura, M.; Terai, G.; Baker, S. E.; Koike, H.; Machida, M. Genome sequence of the Mucoromycotina fungus *Umbelopsis isabellina*, an effective producer of lipids. *Genome Announc.*, **2014**, *2(1)*, e00071-14. https://doi.org/10.1128/genomeA.00071-14
148. Yang, H.; Wang, Y.; Zhang, Z.; Yan, R.; Zhu, D. Whole-genome shotgun assembly and analysis of the genome of *Shiraia* sp. strain Slf14, a novel endophytic fungus producing huperzine A and hypocrellin A. *Genome Announc.*, **2014**, *2(1)*, e00011-14. https://doi.org/10.1128/genomeA.00011-14
149. . Yang, Y.; Liu, B.; Du, X.; Li, P.; Liang, B.; Cheng, X.; Du, L.; Huang, D.; Wang, L. ;Wang, S. Complete genome sequence and transcriptomics analyses reveal pigment biosynthesis and regulatory mechanisms in an industrial strain, *Monascus purpureus* YY-1. *Sci. Rep.*, **2015**, *5(1)*, 1-9. https://doi.org/10.1038/srep08331
150. Savitha, J.; Bhargavi, S. D.; Praveen, V. Complete genome sequence of soil fungus *Aspergillus terreus* (KM017963), a potent lovastatin producer. *Genome Announc.*, **2016**, *4(3)*, e00491-16. https://doi.org/10.1128/genomeA.00491-16
151. Bhargavi, S. D.; Praveen, V. K.; Anil Kumar, M.; Savitha, J. Comparative study on whole genome sequences of *Aspergillus terreus* (soil fungus) and *Diaporthe ampelina* (endophytic fungus) with reference to lovastatin production. *Curr. Microbiol.*, **2018**, *75(1)*, 84-91

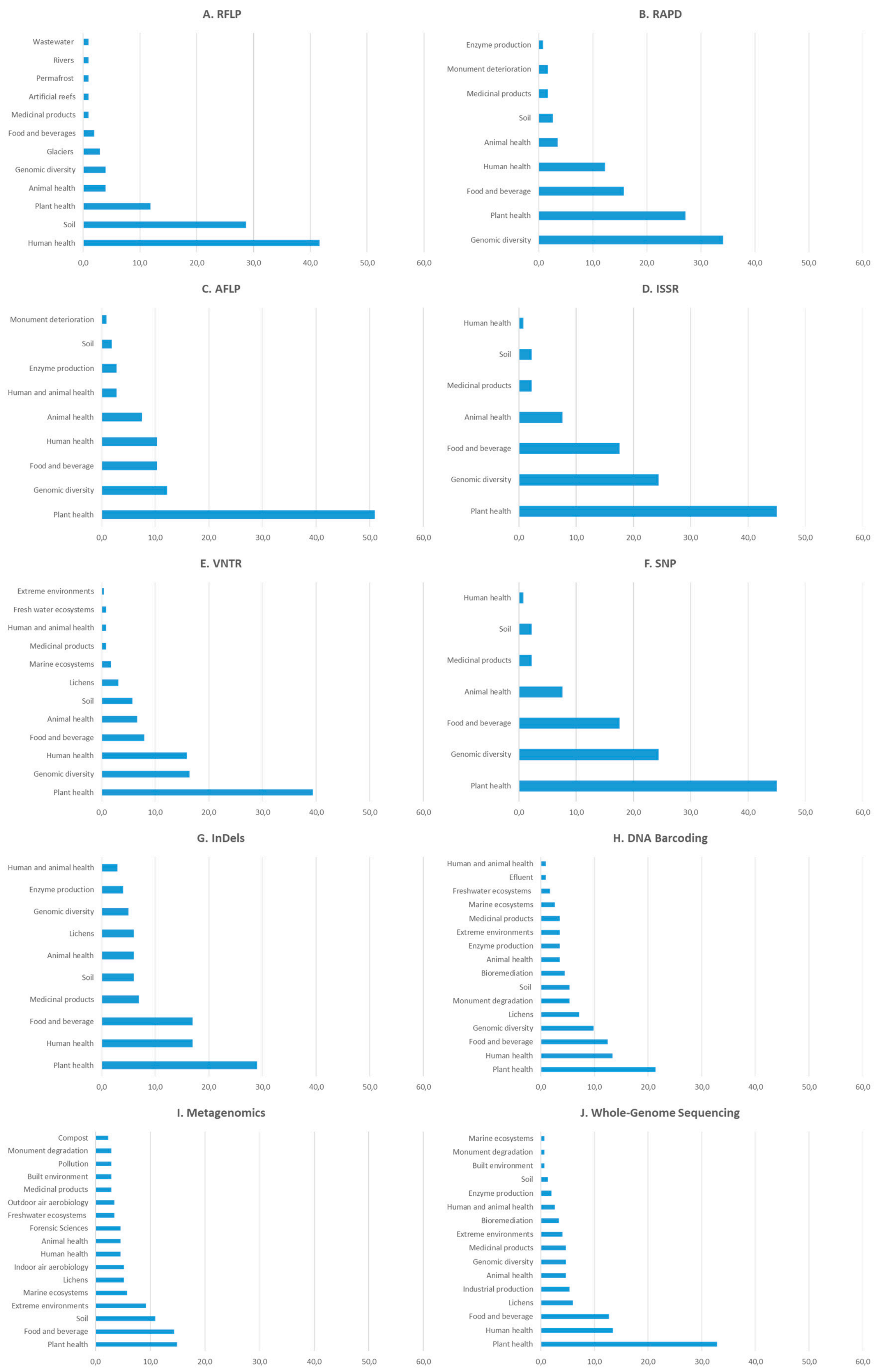

Supplementary Figure S1. Distribution of the published papers per molecular marker.

Supplementary Table S1. Molecular markers in fungi: an overview of manuscripts published in the last ten years using RFLP and similar techniques (HRM: high resolution melting; PCR-RFLP: PCR restriction fragment length polymorphism; qPCR: quantitative PCR; tHDA-RFLP: thermophilic helicase DNA amplification and restriction fragment length polymorphism; T-RFLP: PCR-terminal restriction fragment length polymorphism; UMPCR-RFLP: Universal MT-RFLP).

**Importance**	**Molecular marker**	**Fungal identification**	**Purpose**	**Reference**
Human health	PCR-RFLP	*Alternaria chlamydospora*	Identification of the etiological agent of malignant otitis externa	[1]
	PCR-RFLP	*Aspergillus* spp.	Identification of the clinically important *Aspergillus* species	[2–4]
	PCR-RFLP	*Aspergillus flavus*<br>*Penicillium chrysogenum*<br>*Candida glabrata* species complex	Identification of the etiological agent of non-invasive fungal sinusitis	[5]
	PCR-RFLP	*Candida* spp.	Identification of the etiological agent of oral thrush in HIV-positive patients	[6]
	PCR-RFLP	*Candida spp.*	Identification of the etiological agent of candemia	[7,8]
	PCR-RFLP	*Candida* spp.	Identification of the etiological agent of cutaneous candidiasis	[8]
	PCR-RFLP	*Candida* spp.	Identification of the etiological agent of onychomycosis	[9,10]
	PCR-RFLP	*Candida* spp.	Identification of the etiological agent of vulvovaginal candidiasis	[11]
	PCR-RFLP	*Candida* spp.	Identification of medically important *Candida* species	[12]
	PCR-RFLP	*Candida* spp.	Identification of the etiological agent of neonatal candidemia	[13]
	PCR-RFLP	*Candida* spp.	Study of the frequency of *Candida* species in the oral cavity of narcotics and stimulants smokers	[14]
	T-RFLP	*Candida albicans*<br>*Candida dubliniensis*	Differentiation between *species* associated with superficial and systemic infections	[15]
	PCR-RFLP	*Candida parapsilosis* species complex	Identification and differentiation of the *C. parapsilosis* complex species	[16]
	PCR-RFLP	*Candida* spp.<br>*Cryptococcus* spp.<br>*Histoplasma capsulatum*	Diagnosis of invasive mycoses	[17]
	PCR-RFLP	*Cryptococcus neoformans*<br>*Cryptococcus gattii* species complex	Identification and differentiation of the species included in the same species complex	[18]

	RFLP	Dermatophytes	Detection of source of dermatophytosis	[19]
	PCR-RFLP	Dermatophytes	Study of the molecular variation analysis of species *Arthroderma benhamiae* associated with dermatophytosis	[20]
	PCR—RFLP	Dermatophytes	Identification of the etiological agent of dermatophytosis	[21–23]
	PCR-RFLP	Dermatophytes	Differentiation between environmental and clinically isolates	[24]
	PCR-RFLP	Dermatophytes	Study of the molecular variation analysis of *Fusarium* species associated with dermatophytosis	[25]
	RFLP	Dermatophytes	Identification of the etiological agent of dermatophytosis	[26]
	RFLP-PCR	Dermatophytes<br>*Candida* spp.	Identification of clinical isolates from hair and skin samples	[27]
	PCR-RFLP	Dermatophytes<br>Non-dermatophytes	Confirmatory test for onychomycosis	[28]
	PCR-RFLP	Dermatophytes<br>Non-dermatophytes	Identification and antifungal susceptibility of species associated with onychomycosis	[29]
	RFLP	*Enterocytozoon bieneusi*	Identification of *E. bieneusi* isolated from HIV-infected patients	[30]
	PCR-RFLP	*Mucorales* species	Molecular diagnosis of rhino-orbito-cerebral mucormycosis	[31]
	RFLP	*Pneumocystis jirovecii*	Identification of the etiological agent of pneumocystis pneumonia in immunocompromised patients	[32]
	PCR-RFLP	*Paracoccidioides* spp.	Identification of the etiological agent of paracoccidioidomycosis	[33]
	RFLP	*Proteus mirabilis*	Identification and phyletic evolution analysis of *Proteus mirabilis* strains	[34]
	tHDA-RFLP	*Pythium insidiosum*	Discrimination between species associated with pythiosis	[35]
	PCR-RFLP	*Scopulariopsis*	Identification of genera associated with opportunistic infections	[36]
	UMPCR-RFLP	*Talaromyces marneffei*	Identification of the etiological agent of opportunistic infections in immunocompromised patients	[37]
	T-RFLP	*Trichophyton* spp.	Identification of the etiological agent of onychomycoses	[38]
	RFLP	*Trichophyton mentagrophytes* species complex	Discrimination between species among the species complex	[39,40]
	PCR-RFLP	*Sporothrix schenckii* complex species	Identification of the etiological agent of sporotrichosis	[41,42]
	T-RFLP	Dermatophytes	Identification of dermatophytes in veterinary mycology	[43]
	RFLP	Gut microbiome	Intestinal fungal diversity of sub-adult giant panda	[44]

Animal health	PCR-RFLP	*Malassezia* spp.	Identification of *Malassezia* species isolated from animals	[45]
	PCR-RFLP	Yeast	Identification of yeast species isolated from bovine intramammary infection	[46]
	PCR-RFLP	*Aspergillus* spp.<br>*Alternaria* spp.<br>*Penicillium* spp.	Identification of fungal contamination of date palm tissue cultures	[47]
Plant health	RFLP	*Beauveria* spp.<br>*Metarhizium* spp.	Plant tissue localization of the endophytic pathogenic fungi	[48]
	PCR-RFLP	*Colletotrichum gloeosporioides*<br>*C. truncatum*	Discrimination between fungal species associated with anthracnose in papaya and bell pepper	[49]
	PCR-RFLP	*Colletotrichum gloeosporioides sensu lato*	Discrimination between strains	[50]
	PCR-RFLP	Endophytic fungi	Identification of endophytic fungi of sugarcane	[51,52]
	T-RFLP	Endophytic fungi	Identification of endophytic fungi of *Alpinia officinarum*	[53]
	PCR-RFLP	*Fomes fomentarius*	Discrimination of *Fomes fomentarius* genotypes	[54]
	PCR-RFLP	Fungal pathogens	Identification of fungal pathogens in potato crops	[55]
	T-RFLP	Fungal phyllosphere fungi	Effects of Summer season and long-term drought in the foliar phyllosphere	[56]
	T-RFLP	*Fusarium oxysporum* f. sp. *fragariae*	Study of genetic diversity of the etiological agent of Fusarium wilt in strawberries	[57]
	T-RFLP	*Puccinia psidii*	Study of genetic diversity of the etiological agent of rust in Eucalyptus spp. and other trees	[58]
	PCR- RFLP	Fungal species	Identification of pineapple spoilage fungi	[59]
Food and beverages	T-RFLP	Yeast	Identification of yeast used in wine fermentation in Chinese enology	[60]
	PCR-RFLP	*Ophiocordyceps sinensis*	Identification of Chinese caterpillar medicinal mushroom from counterfeit species	[61]
Medicinal products	PCR-RFLP	*Cercospora beticola*	Analysis of fungicide resistance	[62]
Genomic diversity	RFLP	*pfle* somatic hybrids	Characterization of somatic hybrids from *Pleurotus florida* and *Lentinula edodes*	[63]
	PCR-RFLP	*Aspergillus flavus*	Study of the molecular variation analysis of *A. flavus* from clinical and environmental isolates	[64]
	PCR-RFLP	*Volvariella volvacea*	Identification of A mating-type loci in *Volvariella volvacea*	[65]

	T-RFLP	Mycorrhizal fungi	Effect of different degrees of salinization	[66]
Soil	T-RFLP	Mycorrhizal fungi	Effect of time and plant composition	[67]
	T-RFLP	Mycorrhizal fungi	Effects of plant composition	[68]
	T-RFLP	Mycorrhizal fungi	Effects of maize expressing the *Bacillus thuringiensis* Cry1Ab endotoxin (Bt maize)	[69]
	T-RFLP	Mycorrhizal fungi	Effects of local environment and geographical distance	[70,71]
	T-RFLP	Mycorrhizal fungi	Effects of fertilization	[72–74]
	T-RFLP	Mycorrhizal fungi	Effects of geographical distance	[75,76]
	T-RFLP	Mycorrhizal fungi	Effect of poly (lactic) acid (PLA)	[77]
	T-RFLP	Mycorrhizal fungi	Effects of root age from *Panax ginseng*	[78]
	T-RFLP	Mycorrhizal fungi	Effects of plant composition	[79,80]
	T-RFLP	Mycorrhizal fungi	Effects of till	[81]
	T-RFLP	Mycorrhizal fungi	Effects of host during ecosystem development	[82]
	T-RFLP	Mycorrhizal fungi	Effects of time	[83]
	T-RFLP	Mycorrhizal fungi	Effects of transgenic crops	[84]
	T-RFLP	Mycorrhizal fungi	Effects of long-term effects of multiwalled carbon nanotubes and graphene	[85]
	T-RFLP	Mycorrhizal fungi	Effects of plant organ and development of stage winter wheat	[86]
	RFLP	Mycorrhizal fungi	Effects of season	[87]
	RFLP	Mycorrhizal fungi	Effects of tree harvesting	[88]
	RFLP	Mycorrhizal fungi	Effects of the invasive plant *Impatiens glandulifera*	[89]
	T-RFLP	Mycorrhizal fungi	Effects of biochar	[90,91]
	T-RFLP	Mycorrhizal fungi	Effects of pentachlorophenol	[92]
	T-RFLP	Mycorrhizal fungi	Effects of tea plantation ages	[93]
	T-RFLP	Mycorrhizal fungi	Effects of herbivory and soil water availability	[94]
	T-RFLP	Fungal communities	Study of fungal diversity of marine biofilms on artificial reefs	[95]
Artificial reefs	T-RFLP	Fungal communities	Identification of fungal succession in the forefield of a receding glacier	[96]
Glaciers	T-RFLP	Fungal communities	Identification of fungal isolates from cryoconite holes on glaciers	[97]
	T-RFLP	Fungal communities	Study of fungal responses to reciprocal soil transfer along a temperature and soil moisture	[98]
	T-RFLP	Fungal communities	Study of the diversity and community structure of fungi	[99]

Permafrost	PCR-RFLP	Fungal communities	Influence of water quality on diversity and composition of fungal communities	[100,101]
Rivers	PCR-RFLP	Fungal communities	Identification of fungal isolates from rivers with various contaminations	[102]
Wastewater	ITS-RFLP	Fungal communities	Identification of fungal isolates from textile wastewater	[103]

Supplementary Table S2. Molecular markers in fungi: an overview of manuscripts published in the last 10 years using RAPD and similar techniques.

Importance	Molecular marker	Fungal identification	Purpose	Reference
Human health	RAPD	*Candida albicans*	Analysis of isolates from clinical sources of hospital in south China	[1]
	RAPD	*Aspergillus niger*<br>*Aspergillus flavus*	Molecular characterization of infections in an Iranian educational hospital	[2]
	RAPD	*Candida* spp.<br>*Aspergillus* spp.	Survey of opportunistic fungi in the clinical and environmental specimens from Urmia educational hospitals	[3]
	RAPD	*Candida albicans*<br>*Candida glabrata*	Molecular epidemiology of strains isolated from intensive care unit	[4]
	RAPD	*Scopulariopsis* spp.<br>*Microascus* spp.	Study of the genetic diversity of opportunistic human pathogenic fungi	[5]
	RAPD	*Rhizopus microsporus*	Identification of the etiological agent of a mucormycosis	[6]
	RAPD	*Candida* spp.	Study of genetic diversity and relationships among the clinical isolates	[7]
	RAPD-PCR	*Candida albicans*	Evaluation of genomic polymorphism isolates from patients with vaginitis	[8]
	RAPD-PCR	*Aspergillus flavus*<br>*Aspergillus tamarii*	Identification of DNA polymorphisms of isolates from corneal ulcer/keratitis cases	[9]
	RAPD-PCR	*Trichophyton rubrum*	Genomic polymorphism of *T. rubrum* isolated from keratinized clinical sources	[10]
Animal health	RAPD	*Metarhizium anisopliae* var. *anisopliae*	Study of the genetic diversity among isolates of the entomopathogenic fungus	[11]
	RAPD–PCR	*Saprolegnia* spp.	Molecular identification of a pathogenic aquatic fungi from rainbow and Caspian trout eggs	[12]
	RAPD	*Monacrosporium eudermatum*	Study of the genetic diversity of the nematode trapping fungus	[13]
	RAPD	*Metarhizium anisopliae*<br>*Isaria amoenerosea*	Study of genetic diversity and pathogenicity of the entomopathogenic fungi	[14]
Plant health	RAPD	Pyrenophora graminea	Study of the molecular phylogeny of the etiological agent of barley leaf stripe	[15]

	RAPD-PCR	*Phaeoacremonium parasiticum*<br>*Phaeacremonium aleophilum*<br>*Phaeomoniella chlamydospore*<br>*Fomitiporia mediterranea*	Study on genetic diversity of fungi associated with esca disease	[16]
	RAPD	*Drechslera teres*	Diversity in the Polish isolates from spring barley	[17]
	PCR-RAPD	*Fusarium oxysporum f. sp. psidii*<br>*Fusarium solani*	Profiling of etiological agent of guava wilt disease	[18]
	RAPD	*Alternaria alternata*	Study of the genetic diversity and pathogenicity of the etiological agent of brown spot, leaf spot, and black rot in citrus hybrids trees	[19]
	RAPD	*Ustilago maydis*	Study of genetic diversity of the etiological agent of smuts in maize	[20]
	RAPD	*Sporisorium scitamineum*	Study on genetic diversity of the etiological agent of smuts in maizesugarcane	[21]
	RAPD	*Lasiodiplodia theobromae*	Characterisation of the etiological agent of crown rot disease in banana fruits	[22]
	RAPD	*Fusarium oxysporum* f. sp. *cumini*	Study of the genetic variability of the etiological agent of wilt in cumin	[23]
	RAPD	*Mycosphaerella pinodes*<br>*Phoma pinodella*<br>*Phoma koolunga*<br>*Ascochyta pisi*	Characterization of the etiological agents of ascochyta blight of field pea	[24]
	RAPD	*Bipolaris sorokiniana*	Study of genetic diversity and phylogeny of the etiological agent of spot blotch disease in wheat	[25]
	RAPD	*Myrothecium* spp.	Study of genetic diversity of endophytic fungi	[26]
	RAPD	*Aspergillus flavus*	Characterization of the fungi associated with *Vitis vinifera* decline	[27]
	RAPD	*Phytophthora colocasiae*	Study of the genetic diversity of the etiological agent of leaf blight of taro	[28]
	RAPD	*Tulasnella* spp.	Study of morphological and molecular characterization of fungi isolated from the roots from *Epidendrum secundum*	[29]
	RAPD	*Trichoderma atroviride*	Study of genetic variability of strains isolated from a lentil field	[30]
	RAPD	*Alternaria lini*	Study of genetic diversity of isolates from linseed	[31]

	RAPD	*Fusarium solani*	Study of genetic diversity and pathogenicity of strains isolated from chickpea	[32]
	RAPD	*Fusarium udum*	Study of the genetic diversity of the etiological agent of wilt in pigeonpea	[33]
	RAPD	*Stemphylium solani*	Differentiation among isolates from two varieties of eggplant	[34]
	RAPD	*Alternaria* spp<br>*Cladosporium* spp<br>*Epicoccum* spp<br>*Ulocladium* spp	Identification of different fungal fruit rot pathogens of date palm	[35]
	RAPD	*Diaporthe/Phomopsis*	Identification of strains from fruit trees	[36]
	RAPD	*Pestalotia* sp.	Study of the genetic diversity of isolates of endophytes from different hosts	[37]
	RAPD	*Fusarium oxysporum*<br>*Fusarium solani*<br>*Fusarium equiseti*	Study of the genetic diversity of the etiological agent of root rot in apple	[38]
	RAPD	*Fusarium oxysporum*<br>*Fusarium equiseti*<br>*Fusarium subglutinans*<br>*Fusarium proliferatum*	Identification and characterization etiological agent of wilt in tomato	[39]
	RAPD	*Fusarium moniliforme*<br>*Fusarium xylarioides*<br>*Fusarium fusarioides*	Study of the genetic diversity and phylogeny of the etiological agent of pokkah boeng disease in sugarcane	[40]
	RAPD	*Sclerotium rolfsii*	Study of the genetic diversity of the etiological agent of collar rot in chickpea	[41]
	RAPD	*Puccinia graminis*<br>*Puccinia tricina*<br>*Puccinia striiformis*	Molecular characterization of predominant Indian wheat rust pathotypes	[42]
	RAPD	*Alternaria alternata*<br>*Aspergillus neoflavipes*<br>*Curvularia geniculate*<br>*Penicillium singorense*	Study of phylogeny and genetic diversity of potent endophytic fungi isolated from medicinal plants	[43]
	RAPD	*Aureobasidium pullulans*	Study of the genetic diversity and population structure in withered grape carposphere	[44]

	RAPD	*Sporisorium scitamineum*	Genetic characterization of isolates in sugarcane	[45]
Food and beverage	RAPD	*Penicillium roqueforti*	Molecular and mycotoxigenic identification of fungi in moldy civil cheese	[46]
	RAPD	*Aspergillus* spp.	Characterization of species associated with commercially stored triphala powder	[47]
	RAPD	*Agaricus bisporus*	Assisted development of improved strains	[48]
	RAPD	*Aspergillus* spp.<br>*Penicillium* spp.<br>*Rhizopus* spp.<br>*Fusarium* spp.<br>*Alternaria alternata*<br>*Mucor* spp.	Identification of fungi from stored citrus	[49]
	PCR-RAPD	*Pleurotus ostreatus*<br>*Pleurotus sapidus*	Differentiation between edible mushrooms	[50,51]
	RAPD	*Agaricus bisporus*<br>*Pleurotus eryngii*<br>*Lentinus edodes*<br>*Hypsizygus tesselatus*<br>*Flammulina velutipes*<br>*Pleurotus ostreatus*<br>*Pleurotus djamor*<br>*Calocybe indica*<br>*Pleurotus florida*	Discrimination between commercial mushrooms	[52]
	RAPD	*Aspergillus flavus*	Study of the genetic diversity of strains isolates from barley grains	[53]
	RAPD	*Aspergillus flavus*	Study of the genetic diversity of strains isolates from chilies	[54]
	RAPD	*Alternaria solani*	Study of the genetic diversity among isolates from potatoes	[55]
	RAPD	*Pleurotus pulmonarius*	Study of the genetic diversity of edible mushrooms	[56]
	RAPD	*Aspergillus flavus*	Detection of seedborne Aspergillus flavus from rice cultivars	[57]
	RAPD	Agaricomycetes	Molecular characterization and phylogeny of mushrooms	[58]
	RAPD	*Aspergillus* spp.<br>*Penicillium* spp.<br>*Saccharomyces cerevisiae*<br>*Wickerhamomyces anomalus*	Identification of contaminative fungi isolated from rye breads	[59]

	RAPD	*Monascus* spp.	Study of the genetic diversity of strains with culinary and medicinal applications	[60]
	RAPD	*Aspergillus niger*<br>*Aspergillus flavus*<br>*Aspergillus fumigatus*<br>*Penicilium citrinum.*	Study of the genetic diversity of mycotoxin-producing fungi from contaminated animal feed	[61]
	RAPD	*Pleurotus cystidiosus*<br>*Pleurotus ostreatus*<br>*Pleurotus flabellatus*<br>*Pleurotus flabellatus*<br>*Pleurotus pulmonarius*<br>*Pleurotus fossulatus*<br>*Pleurotus eryngii*<br>*Pleurotus sapidus*<br>*Pleurotus florida*	Study of the genetic diversity of edible mushrooms	[62]
	RAPD	*Colletotricum gleosporioides*	Study of the prevalence and genetic diversity of mycotoxigenic-producing strains from wheat grains	[63]
	RAPD	Seed-borne pathogenic fungi	Study of genetic diversity of maize seed-borne pathogenic fungi	[64]
Fungal diversity	RAPD	*Fusarium verticillioides*	Study of the genetic diversity and vegetative compatibility of a major fungal pathogen from cereals in Iran	[65]
	RAPD	*Rhizoctonia solani*	Study of the genetic diversity in isolates from different agro-ecological zones	[66]
	RAPD	*Phytophthora colocasiae*	Study of the genetic diversity	[67]
	RAPD	*Alternaria alternata*	Study of the genetic diversity	[68]
	RAPD	*Gaeumannomyces graminis* var. *tritici*	Study of the genetic diversity of populations	[69]
	RAPD	*Monilinia fructicola*	Study of genetic diversity in strains from the Ebro Valley in Spain	[70]
	RAPD	*Fusarium* spp.	Study of genetic diversity among 14 different species	[71]
	RAPD	*Fusarium semitectum*	Study of morphological and genetic diversity of the isolates from cereal grains	[72]
	RAPD	*Trichoderma* spp.	Molecular characterization of 12 isolates	[73]
	RAPD	*Pyricularia* spp	Study of the genetic diversity in phytopathogenic	[74]

	RAPD	*Rhizoctonia* spp.	Study of the genetic diversity in phytopathogenic	[75]
	RAPD	*Fusarium oxysporum* f. sp. *raphani*,	Differentiation of isolates of the pathogen among cultivated and wild rocket	[76]
	RAPD	*Conidiobolus coronatus*	Study of the genetic diversity	[77]
	RAPD	*Verticillium chlamydosporium*	Genetic characterization of *V. chlamydosporium* isolated from Pakistan	[78]
	RAPD	*Lentinula edodes*	Study on the genetic diversity of *L. edodes* strains	[79]
	RAPD	*Fusarium solani*	Study of the genetic diversity of the etiological agent of citrus dry root rot	[80]
	RAPD	*Aspergillus flavus*	Fingerprinting of toxigenic and non-toxigenic strains isolated from different habitats	[81]
	RAPD	*Fusarium* sp.	Characterization of antibacterial activity	[82]
	RAPD	*Botrytis alli*	Study of genetic diversity of the agent of onion gray mold	[83]
	RAPD	*Aspergillus niger*	Comparative sequence analysis of citrate synthase and 18S ribosomal DNA from a wild and mutant strains of *A. niger* with various fungi	[84]
	RAPD	*Trichophyton mentagrophytes*	Terbinafine susceptibility and genotypic heterogeneity in clinical isolates	[85]
	RAPD	*Aspergillus niger*	Study of the genetic diversity among isolates	[86]
	RAPD	*Penicillium* spp.	Molecular Characterization of Isolates	[87]
	RAPD	*Rhizoctonia solani*	Study of the genetic variability of isolates of different geographical regions and hosts	[88,89]
	RAPD-PCR	*Beauveria bassiana*	Study of the genetic diversity	[90,91]
	RAPD	*Ganoderma lucidum*	Study of genetic diversity	[92]
	RAPD	*Fusarium asiaticum*	Identification of a novel phenamacril-resistance-related gene	[93]
	RAPD	*Sclerotinia sclerotiorum*	Study of genetic diversity of strains isolated from eggplant	[94]
	RAPD	*Morchella* sp.	Study of the genetic diversity	[95]
	RAPD	*Aspergillus* section Nigri	Study of the genetic diversity of ochratoxin-producing fungi	[96]
	RAPD	*Fusarium pallidoroseum*	Study of the genetic diversity	[97]
	RAPD	*Ganoderma lucidum*<br>*Leucoagricus* spp.<br>*Lentinus* spp.	Study of genetic diversity among three most frequent species of macrofungi from a tropical forest	[98]
	RAPD	*Trichoderma* spp.	Study of the genetic diversity	[99]

	RAPD	*Colletotrichum falcatum*	Study of genetic diversity	[100]
	RAPD	Flammulina velutipes	Study of the genetic diversity	[101]
	RAPD	*Bjerkandra adusta*<br>*Fomes fomentarius*<br>*Rigidoporus ulmarius*<br>*Tremetes versicolor*	Study of the genetic diversity of macrofungi	[102]
Medicinal products	RAPD	*Lentinus edodes*	Molecular characterization of shiitake medicinal mushroom strains	[103]
	RAPD-SCAR	*Ganoderma lucidum*<br>*Ganoderma sinense*<br>*Ganoderma tropicum*<br>*Ganoderma gibbosum*	Differential authentication of the presence of *Ganoderma* species in medicinal products	[104]
Enzyme production	RAPD	*Aspergillus niger*<br>*Penicillium chrysogenum*	Pectinase hyperproduction in fungi Isolates	[105]
Soil	RAPD	*Aspergillus* spp.	Identification of antibiotic producing fungi from soil	[106]
	RAPD	*Glomus* spp.<br>*Gigaspora* spp.<br>*Scutellospora* spp.	Study of the genetic diversity of arbuscular mycorrhizal fungi	[107]
	RAPD	*Trichoderma asperellum*<br>*Trichoderma longibrachiatum*<br>*Trichoderma harzianum*	Identification of rhizosphere fungi and their antagonistic impact against plant pathogenic fungi	[108]
Monument deterioration	RAPD	*Rhizopus* spp.	Study of the genetic diversity of fungi associated with the deterioration of historical sandstone monuments	[109]
	RAPD	*Aspergillus niger*<br>*Aspergillus fumigatus*	Study of ecologic and genetic diversity of black fungus from associated with the deterioration of stone monuments	[110]

Supplementary Table S3. Molecular markers in fungi: an overview of manuscripts published in the last 10 years using AFLP and similar techniques.

Importance	Fungal identification	Purpose	Reference
Human health	*Cladophialophora carrionii*<br>*Fonsecaea* spp.	Study of genetic diversity of the etiological agent of chromoblastomycosis	[1]
	Candida tropicalis	Study of azole resistance genes in the etiological agent of opportunistic infections	[2]
	Apophysomyces variabilis	Determination of environmental sources of the etiological agent of mucormycosis	[3]
	Sporothrix globosa	Study of genetic diversity of the etiological agent of sporotrichosis	[4]
	Hortaea werneckii	Study of genetic diversity of the halotolerant fungus causing tinea nigra	[5]
	Aspergillus *fumigatus*<br>*Penicillium* spp.<br>*Aspergillus* spp.<br>*Scedosporium* spp.<br>*Exophiala dermatitidis*<br>*Cladosporium* spp.	Study of the genetic diversity and prevalence of filamentous fungi in the airways of cystic fibrosis patients	[6]
	Trichophyton schoenleinii	Molecular epidemiology of the etiological agent of *tinea capitis favosa*	[7]
	Sporothrix spp.	Study of genetic diversity of the etiological agent of sporotrichosis	[8,9]
	Candida auris	Evaluation of *C. auris* associated with nosocomial infections	[10]
	Paracoccidioides spp.	Study of genetic diversity, population structure, and phylogeography of the etiological agent of paracoccidioidomycosis	[11]
	Coccidioides posadasii	Study of genetic diversity of the etiological agent of coccidioidomycosis	[12]
Human and animal health	*Cryptococcus gattii*	Study of the origin and genetic diversity of the etiological agent of cryptococcosis	[13]
	Cryptococcus spp.	Study of the environmental distribution	[14]
	Black yeast-like fungi	Identification of fungi associated with lethargic crab disease in the mangrove-land crab	[15]
	Entomopathogenic fungi	Study of genetic diversity of entomopathogenic fungi	[16]
Animal health	*Beauveria bassiana*	Study of genetic diversity of the arthropod species' parasite	[17]
	Aphanomyces astaci	Transmission from invasive to wild populations of crayfish	[18]
	Metarhizium flavoviride	Study of the genetic diversity of the Hemiptera and Coleoptera species' parasite	[19]
	Acromyrmex echinatior<br>*Acromyrmex octospinosus*	Study of somatic incompatibility and genetic structure of fungal crops in sympatric ants	[20]
	Beauveria bassiana	Study of differential genes expression of the entomopathogenic fungus during infection of lepidopteran larvae	[21]

Plant health	*Pseudocercospora griseola*	Study of the genetic diversity of the etiological agent of angular bean leaf spot	[22]
	Colletotrichum gloeosporioides	Study of the genetic diversity of the etiological agent of anthracnose	[23]
	Bipolaris sorokiniana (*Cochliobolus sativus*)	Study of genetic diversity, virulence and population structure of the etiological barley and wheat spot blotch	[24–26]
	Veronaea botryosa	Study of genetic diversity of the etiological agent of asian soybean rust	[27]
	Neotyphodium spp.	Study of the genetic diversity of the endophytic fungi symbiotic with grasses	[28]
	Puccinia striiformis	Study of genetic diversity of the etiological agent of yellow rust	[29]
	Pyrenophora graminea	Study of virulence on barley	[30]
	Fusarium mangiferae	Diagnosis and phylogeny of the etiological agent of mango malformation disease	[31]
	Colletotrichum sublineolum	Study of genetic diversity of the etiological agent of anthracnose in sorghum	[32]
	Mycosphaerella pinodes	Study of genetic diversity of the etiological agent of ascochyta blight on pea	[33]
	Fusarium oxysporum f. sp. *acaciae*	Study of pathogenicity of fungal species responsible for *Acacia koa* dieback	[34]
	Fusarium oxysporum f. sp. *lycopersici*	Study of the genetic diversity of the etiological agent of wilt in tomato	[35]
	Fusarium udum	Study of the genetic variability of the etiological agent of the wilt in pigeon pea	[36]
	Coniophora complex species	Identification of species associated with rotten wood	[37]
	Tilletia foetida	Detection of pathogen on wheat	[38]
	Fusarium solani	Detection of etiological agent of peanut brown root rot	[39]
	Seiridium cardinale	Study of the population structure of the etiological agent of canker in Cupressaceae	[40]
	Hemileia vastatrix	Study of population structure of the etiological agent of coffee leaf rust	[41]
	Diplodia seriata	Discrimination of fungal species associated with grapevine decline	[42]
	Phaeomoniella chlamydospore		
	Phaeoacremonium aleophilum		
	Alternaria alternata	Study of the genetic diversity in toxigenic *Alternaria* species in wheat	[43]
	Alternaria infectoria		
	Macrophomina phaseolina	Identification of three fruit-rot fungi of banana	[44]
	Fusarium oxysporum		
	Nigrospora oryzae		
	Fusarium oxysporum f. sp. *passiflorae*	Identification of the etiological agent of fusariosis in passionfruit	[45]
	Venturia inaequalis	Study of genetic diversity of the etiological agent of apple scab within mixed orchards	[46]
	Phaeoacremonium aleophilum	Characterization of the fungi associated with *Vitis vinifera* decline	[47]

	Phytophthora colocasiae	Study of the genetic diversity of the etiological agent of leaf blight of taro	[48]
	Magnaporthe spp.	Characterization of the fungi associated with rice blast	[49]
	Pyrenophora teres f. *teres*	Study of virulence factors of the etiological agent of foliar disease net form net blotch	[50]
	Fusarium circinatum	Evidence for a new introduction of the pitch canker fungus	[51]
	Pyrenophora teres<br>*Sphaerulina musiva*	Studies of positional cloning in biparental fungal populations, association mapping of natural fungal populations and population genetics studies	[52]
	Phakopsora pachyrhizi	Study of genetic diversity of the etiological agent of soybean rust	[53]
	Podosphaera xanthii	Study of genetic diversity and population structure of the etiological agent of powdery mildew in cucurbit	[54]
	Puccinia striiformis f. sp. *tritici*	Study of genetic diversity and virulence factors of the etiological agent of yellow stripe rust on wheat varieties and their mixtures	[55]
	Rhizoctonia solani	Study of differentially expressed genes associated with sclerotial metamorphosis	[56]
	Aspergillus flavus	Characterisation of isolates from peanut fields	[57]
	Fusarium oxysporum f. sp. *strigae*	AFLP-based marker for diagnosis of the fungi in tropical soils	[58]
	Colletotrichum coccodes	Study of genetic diversity of the etiological agent of anthracnoses and black-dot in crops	[59]
	Sclerotinia sclerotiorum	Study of genetic diversity of the rot producing fungal plant pathogen	[60]
	Fusarium fujikuroi species complex	Distinction of species within the species complex	[61]
	Sporisorium scitamineum	Study of genetic diversity and relationship of the etiological agent of smuts in sugarcane smut	[62]
	Neonectria ditissima	Study of genetic diversity and phylogeny of the etiological agent of fruit tree canker	[63]
	Pyricularia grisea	Study of genetic diversity of the etiological agent of rice blast	[64]
	Fusarium verticillioides	Study of genetic diversity of the etiological agent of rot in maize kernels	[65]
	Blumeria graminis f. sp. *hordei*	Study of genetic diversity of the etiological agent of barley powdery mildew	[66]
	Fusarium proliferatum	Study of genetic diversity of the fungal pathogen of maize, rice, sugarcane and onion	[67]
	Puccinia striiformis f. sp. *tritici*	Characterization of populations of the etiological agent of yellow stripe rust	[68]
	Alternaria solani	Study of genetic diversity and aggressiveness of the etiological agent of tomato blight	[69]
	Colletotrichum gloeosporioides	Study of the genetic diversity of the etiological agent of anthracnose in water yam (*Dioscorea alata*)	[70]

	Petriella setifera	Study of genetic diversity	[71]
	Fusarium oxysporum f. sp. *elaeidis*	Study of genetic diversity of the etiological agent of fusarium wilt disease of oil palm (*Elaeis guineensis*)	[72]
	Magnaporthe oryzae	Study of differentially expressed genes associated with appressorium formation in the rice blast fungus	[73]
	Fusarium tricinctum	Study of genetic diversity of the etiological agent of root rots of alfalfa	[74]
	Quambalaria pitereka	Study of genetic diversity of the fungal pathogens of *Corymbia*	[75]
Food and beverage	*Fusarium verticillioides*	Study of genetic diversity, fumonisin production and pathogenicity in isolates from maize	[76]
	Aspergillus flavus	Study of genetic diversity of aflatoxin producing fungi in maize	[77]
	Pleurotus spp.	Study of genetic diversity of edible mushrooms	[78]
	Fusarium verticillioides	Identification of the fungus on finger millet in Uganda	[79]
	Agaricus bisporus var. burnettii	Study of the ability of to produce mushrooms at high temperature	[80]
	Aspergillus spp.	Study of genetic diversity of aflatoxin producing fungi in marketed spices	[81]
	Aspergillus flavus	Study of genetic diversity of aflatoxin producing fungi in food and feed samples	[82]
	Coprinus comatus	Study of genetic diversity	[83]
	Aspergillus pseudonomius	Study of genetic diversity of aflatoxin producing fungi in nuts	[84]
	Aspergillus flavus	Study of genetic diversity of aflatoxin producing fungi in the cattle feed	[85]
	Aspergillus parasiticus		
	Aspergillus novoparasiticus	Identification of food contaminants in corn kernels	[86]
	Aspergillus arachidicola		
	Aspergillus pseudocaelatus		
Genomic diversity	*Cochliobolus miyabeanus*	Study of population structure, gene flow and recombination of on cultivated wildrice	[87]
	Cryptococcus spp.	Environmental isolation and characterization of fungal species from living trees	[88]
	Aspergillus flavus	Study of biogeography and phylogeny among isolates	[89]
	Candida glabrata	Differential expression of aldo-keto-reductase in azole-resistant clinical isolates	[90]
	Lentinula ssp.	Study of genetic relationships among shiitake mushrooms.	[91]
	Phoma macrostoma	Biological and genetic characterization of isolates with bioherbicidal activity	[92]
	Fusarium spp.	Study of genetic diversity among isolates	[93]
	Botrytis cinerea	Isolation of genes related to abscisic acid production	[94]
	Leptosphaeria spp.	Study of the genetic diversity and occurrence of on *Brassica oleracea* and *B. napus*	[95]
	Dichomitus squalens	Study of genetic diversity of the etiological agent of white rot	[96]

	Ganoderma lucidum	Study of genetic and metabolic diversity	[97]
	Arbuscular mycorrhizal fungi	Study of genetic diversity	[98]
	Root-endophytic fungi	Study of community structure and dynamics of plant-associated fungi	[99]
Enzyme production	*Myceliophthora* spp.	Study of the genetic diversity and phylogeny of thermostable enzymes producing fungi	[100]
	Corynascus spp.		
	Tricoderma reesei	Study of the genetic diversity of cellulolytic enzyme producing fungi	[101]
	Aspergillus spp.	Study of the genetic diversity of the glucose dehydrogenase producing fungi	[102]
Soil	*Rhizopogon roseolus*	Study of genetic diversity of the an ectomycorrhizal fungus	[103]
	Arbuscular mycorrhizal fungi	Distinction between root system and rhizosphere soil arbuscular mycorrhizal fungi of *Prunus mume*	[104]
Monument deterioration	*Sarcinomyces spp.*	Identification of the fungi contributing to the blackened areas on outdoor marble statues	[105]
	Phaeococcomyces spp.		
	Sporobolomyces yunnanensis		

Supplementary Table S4. Molecular markers in fungi: an overview of manuscripts published in the last 10 years using ISSR and similar techniques.

Importance	Fungal identification	Purpose	Reference
Human health	Dermatophytes<br>*Candida* spp.	Study of genetic relationships and isozyme profile from strains from Egypt and Libya	[1]
Animal health	*Beauveria* spp.<br>*Metarhizium* spp.	Diversity of indigenous fungus from a commercial banana field and their virulence toward *Cosmopolites sordidus*	[2]
	Metarhizium spp.	Study of genetic diversity of the fungal pathogen, causing epizootics in burrower bugs	[3]
	Isaria spp.	Species clarification of *Isaria* isolates used as biocontrol agents against *Diaphorina citri* (Hemiptera: Liviidae)	[4]
	F. incarnatum-equiseti<br>*F. fujikuroi*	Study of genetic diversity of entomopathogenic fungus	[5]
	Metarhizium rileyi	Study of genetic diversity and population structure of the etiological agent of green muscardine in silkworm	[6]
	Lecanicillium	Identification of entomopathogenic fungi	[7]
	Beauveria bassiana	Study of genetic diversity of isolates	[8]
	Helicoverpa gelotopoeon<br>*Diabrotica speciosa*<br>*Beauveria bassiana*<br>*Purpureocillium lilacinum*	Study of genetic diversity of entomopathogenic fungi and its implications for biological control of tobacco pests	[9]
	Beauveria bassiana	Study of acaricidal and pathogenic effects of the entomopathogenic fungus on engorged females of the fowl tick (Argas persicus, Argasidae)	[10]
Plant health	*Pseudocercospora griseola*	Study of genetic diversity of isolates of the fungal bean pathogen	[11]
	Pyrenophora graminea	Study of the molecular phylogeny of the etiological agent of barley leaf stripe	[12]
	Macrophomina phaseolina	Study of genetic diversity of isolates from sesame	[13]
	Ustilago stamina	Study of genetic diversity of the etiological agent of smuts	[14]
	Inonotus obliquus	Study of genetic diversity among 21 strains of plant parasites	[15]
	Colletotrichum spp.	Study of genetic diversity of the etiological agent of bitter rot in apple	[16]
	Bremia lactucae	Genetic diversity in lettuce plantations	[17]
	Colletotrichum gloeosporioides sl	Genetic structure of isolates from the etiological agent of anthracnose in papaya	[18]
	Colletotrichum sublineolum	Study of genetic diversity of the etiological agent of sorghum anthracnose	[19,20]

	Verticillium dahliae	Characterization of the etiological agent of wilt from different geographic origins	[21]
	Rhizoctonia solani AG 2-2 IIIB	Genetic structure of a population isolated from *Agrostis stolonifera*	[22]
	Bipolaris oryzae	Study of genetic diversity and population structure of brown spot fungus isolates	[23]
	Knoxdaviesia proteae	Study of genetic characterization of the ophiostomatoid fungi from South Africa	[24]
	Cadophora luteo-olivacea	Identification of the genetic groups of grapevine fungal pathogens	[25]
	Myrothecium spp.	Study of genetic diversity of endophytic fungi	[26]
	Colletotrichum spp.	Study of genetic diversity and differentiation of isolates associated with Leguminosae	[27]
	Ganoderma spp.	Genotyping	[28]
	Fusarium solani	Study of genetic diversity of etiological agent of yellowing disease from black pepper	[29]
	Cytospora chrysosperma	Study of genetic diversity of the etiological agent of cytodpora canker from walnut trees	[30]
	Alternaria alternata	Study of genetic diversity of the etiological agent of potato brown leaf spot	[31]
	Neofabraea spp.	Identification and characterization of the etiological agent of bull's eye rot on apple	[32,33]
	Botryosphaeria species-complex	Molecular identification of the etiological agent of cankers and drying of buds in *Eucalyptus* sp.	[34]
	Rhizoctonia solani (AG 1-IA)	Study of pathogenic and genetic variation among the isolates of the etiological agent of rice sheath blight	[35]
	Bipolaris sorghicola	Study of genetic diversity of the fungal pathogen infecting sorghum, India	[36]
	Myrothecium roridum	Genetic relationships of isolates from water hyacinth in Thailand	[37]
	Monilinia polystroma	Study of genetic diversity and pathogenicity of a pathogen from cherries	[38]
	Fusarium spp	Study of genetic diversity of the etiological agent of sugarcane wilt	[39]
	Pestalotiopsis longiseta	Study of population genetic structure of the etiological agent of tea gray blight	[40]
	Fusarium oxysporum f. sp. *ciceris*	Study of genetic diversity and population structure of Iranian isolates of the etiological agent of chickpea wilt	[41,42]
	Ustilago esculenta	Identification and genetic diversity of isolates	[43]
	Calonectria pseudonaviculata	Study of genetic diversity of the etiological agent of boxwood blight	[44,45]
	Diaporthe vaccinii	Identification and characterization of the etiological agent of upright dieback and viscid rot of cranberry	[46]

	Fusarium oxysporum f. sp. *cumini*	Study of genetic diversity among isolates	[47]
	Erysiphe pisi	Study of natural incidence and genetic variability of the etiological agent of powdery mildew on peas	[48]
	Monascus spp.	Comparative analysis of genetic polymorphisms among *Monascus* strains	[49]
	Alternaria carthami	Study of population structure and virulence analysis of isolates	[50]
	Zymoseptoria tritici	Study of genetic diversity of the etiological agent of *Septoria tritici* blotch	[51]
	Fusarium solani f. sp. cucurbitae	Genetic diversity of the etiological agent of crown and root rot of watermelon	[52]
	FUsarium proliferatum	Study of genetic diversity of endophytic fungi strains isolated from *Belamcanda chinensis*	[53]
	Macrophomina phaseolina	Study of genetic diversity among isolates of sesame, Iran	[54]
	Fusarium spp.	Study of genetic diversity and phylogenetic profiling of the etiological agent of storage rot of ginger (*Zingiber officinale*)	[55]
	Alternaria solani	Study of genetic diversity of the etiological agent of early tomato blight	[56]
	Neopestalotiosis sp	Study of genetic diversity of the etiological agent canker disease from guava (Psidium guajava L.)	[57]
	Lasiodiplodia theobromae	Study of genetic diversity of the etiological agent of mulberry root rot	[58,59]
	Tilletia laevis	Detection of teliospores from closely related species implicated in wheat diseases	[60]
	C. gloeosporioides (<br>*Fusarium fujikuroi*<br>*Phaeosphaeria nodorum*<br>*Botryosphaeria dothidea*<br>*Pseudofusicoccum ardesiacum*	Identification of six fungal pathogens from the different varieties of the mango (*Mangifera indica* L.)	[61]
	Tulasnella spp.	Identification of fungal populations associated to the Australian evergreen terrestrial orchid *Cryptostylis ovata*	[62]
	Colletotrichum spp.	Study of genetic diversity of the etiological agent of anthracnose in fruit crops	[63]
	Sarocladium oryzae	Study of genetic diversity of the etiological agent of rice sheath rot disease	[64]
	F. oxysporum f. sp. *cubense*	Study of genetic variation of the etiological agent of *Fusarium* wilt in banana	[65]
	Colletotrichum truncatum	Study of pathological, biochemical and genetic diversity of the etiological agent of anthracnose in chilli (*Capsicum annuum* L.)	[66]
	Magnaporthe oryzae	Study of pathogenicity of rice blast fungus	[67]
	Diaporthe sp	Study of genetic diversity of indigenous and exotic species associated with various crops	[68]

	Colletotrichum spp.	Study of genetic diversity of the etiological agent of anthracnose from Mango cv. Nam Dork Mai See Tong	[69]
Genomic diversity	*Armillaria gallica*	Study of genetic diversity	[70]
	A. alternata	Study of genetic diversity	[71]
	F. oxysporum	Study of genetic diversity	[72]
	Monilinia fructicola	Study of genetic diversity	[73]
	Sclerotium rolfsii<br>*Sclerotium delphinii*	Study of genetic diversity	[74]
	Mycosphaerella fijiensis	Study of mating-type alleles and M13 PCR markers in the etiological agent of black leaf spot of bananas	[75]
	Hypsizygus marmoreus	Evaluation of growth characteristics and genetic diversity of commercial and stored lines	[76]
	Pleurotus eryngii var. *tuoliensis*	Study of genetic diversity	[77]
	A. flavus	Study of genetic diversity	[78]
	F. oxysporum	Study of pathogenicity and genetic diversity of isolates from corms of *Crocus sativus*	[79]
	Tricholoma lobayense	Study of genetic diversity	[80]
	Sporisorium scitamineum	Study of biogeographical variation and population structure of isolates	[81]
	Thricoderma spp.	Study of genetic diversity and biocontrol potential of rhizospheric microbes isolated from tomato and maize	[82]
	Verticillium dahliae	Study of genetic diversity, virulence, and vegetative compatibility in isolates from cotton	[83]
	Blumeria graminis f. sp. tritici	Study of genetic diversity and virulence structure	[84]
	Shiraia bambusicola	Study of genetic diversity	[85]
	A. fumigatus	Molecular Identification	[86]
	Ganoderma lucidum	Study of genetic diversity	[87]
	Magnaporthe oryzae	Characterisation of isolates from rice in peninsular Malaysia	[88]
	Fusarium graminearum<br>*F. culmorum*	Study of genetic diversity among isolates	[89]
	Stenocarpella maydis	Study of genetic variability of stalk and ear rot disease in corn plants	[90]
	A. flavus	Study of genetic diversity analysis of isolates from plants and air	[91]
	Colletotrichum graminicola	Study of genetic diversity	[92]
	Corollospora maritima sensu lato	Study of genetic diversity in the marine arenicolous fungus	[93]

	Sclerotium rolfsii	Study of virulence and genetic diversity of isolates infecting groundnut	[94]
	F. oxysporum f. sp. melongenae	Characterization of isolates	[95]
	Curvularia lunata	Study of genetic diversity of indigenous and exotic isolates	[96]
	Aspergillus spp.	Molecular identification and differentiation of *Aspergillus* species and its implications in mycotoxins production	[97]
	Pyricularia oryzae	Study of genetic diversity of rice blast fungus	[98]
	Trichoderma harzianum	Study of antagonistic activity and molecular characterization of the biological control agent	[99]
	Athelia rolfsii	Study of genetic diversity	[100]
	Botrytis cinerea	Study of genetic diversity of isolates from different plant hosts and localities	[101]
Food and beverage	*Auricularia polytricha*	Study of genetic diversity among wild populations of edible mushrooms	[102]
	Pleurotus eryngii	Study of genetic diversity among Chinese edible mushrooms cultivars	[103,104]
	Auricularia auricula-judae	Study of genetic diversity of wild medicinal and edible mushrooms	[105]
	A. bisporus (Button)<br>*A. bisporus* (Portobello)<br>*P. eryngii* (King Oyster)<br>*L. edodes* (shiitake)<br>*H. tessellatus* (Brown Shimeji)<br>*H. tessellatus* (White Shimeji)<br>*F. velutipes* (Enoki)<br>*P. ostreatus* (Oyster)<br>*P. djamor* (Pink Oyster)<br>*C. indica* (Milky)<br>*P. florida* (Florida Oyster)	DNA fingerprinting of commercial mushrooms for genetic discrimination	[106]
	Fusarium spp.	Identification and characterization of isolated associated with sorghum seeds	[107]
	A. flavus	Study of genetic diversity of strains isolated from barley grains	[108]
	A. flavus	Molecular characterization of aflatoxigenic and non-aflatoxigenic isolates of fungal strains isolated from corn grains	[109]
	Agaricus bisporus	Identification and strain-typing of button mushroom	[110]
	Aspergillus section *Flavi*	Identification of members belonging to food-contaminating aflatoxigenic and non-aflatoxigenic species	[111]
	Volvariella volvacea	Phylogenetic analysis of high yielding strain of paddy straw mushroom	[112]
	Pleurotus pulmonarius	Study of the genetic diversity of edible mushrooms	[113]
	Polyporus umbellatus	Study of genetic diversity of edible mushrooms	[114]

	Macrophomina phaseolina	Study of genetic diversity of isolates associated with cowpea	[115]
	Fusarium spp.	Molecular identification and characterization of isolates from wheat grains	[116]
	Pleurotus ostreatus	Study of genetic diversity of different strains	[117]
	Monascus spp.	Comparative analysis of genetic polymorphisms among strains	[118]
	Lepista nuda	Study of genetic diversity	[119]
	Colletotrichum falcatum	Study of genetic diversity	[120]
	Alternaria spp.	Molecular identification and genetic variation of isolates from tomatoes	[121]
	Grifola frondosa	Study of genetic diversity of edible mushrooms	[122]
	Phomopsis asparagi	Study of genetic diversity of the etiological agent of asparagus stem blight	[123]
	Tuber spp	Genetic diversity in some of black and brown Iranian truffles	[124]
Medicinal products	*Wolfiporia cocos*	Study of genetic diversity of Chinese cultivars of medicinal mushrooms	[125]
	Sanghuangporus sanghuang	Study of genetic diversity of wild strains	[126]
	Ganoderma lingzhi	Identification of Lingzhi medicinal mushroom	[127]
Soil	*Aspergillus flavus*	Study of genetic diversity of isolates from peanut-cropped soils in China	[128]
	Trichoderma viride	Molecular characterization of isolates from rhizospheric soils of uttar pradesh	[129]
	Hypocreales	Study of genetic diversity of entomopathogenic fungi in soil and phylloplanes of five Mediterranean cropping systems	[130]

Supplementary Table S5. Molecular markers in fungi: an overview of manuscripts published in the last 10 years using VNTR and similar techniques.

Importance	Molecular marker	Fungal identification	Purpose	Reference
Human health	Microsatellites	*Candida albicans*<br>*Candida glabrata*	Analysis of isolates from recurrent vulvovaginal candidiasis	[1]
	Minisatellite	*Cryptococcus neoformans*<br>*Cryptococcus gattii*	Molecular characterisation of the etiological agent of cryptococcosis in patients of a tertiary healthcare facility	[2]
	Microsatellites	*Cryptococcus neoformans*	Study of genetic diversity of the etiological agent of cryptococcosis	[3]
	Microsatellites	*Candida parapsilosis*	Study of the transmission in neonatal intensive care units	[4]
	VNTR	*Aspergillus flavus*	Tracing sources of the etiological agent of fungal sinusitis, keratitis, and endophthalmitis	[5]
	Microsatellites	*Candida parapsilosis s. s.*	Study of dominance and persistence of a particular epidemiological clone among neonatal intensive care unit patients	[6]
	Microsatellites	*Microsporum* spp.<br>*Trichophyton* spp.	Identification and epidemiology of dermatophytes causing cutaneous mycosis	[7]
	Minisatellites	*Candida glabrata*	Study of genome structure and potential virulence factors of the yeast pathogen	[8]
	VNTR	*Aspergillus flavus*	Study of genetic diversity, relatedness and virulence of the etiological agent of invasive aspergillosis and cutaneous, sinus, nasal and nail infections	[9,10]
	VNTR	*Trichophyton interdigitale*	Study of population structure and genotype differentiation of the etiological agent of superficial fungal infections	[11]
	Minisatellites	*Cryptococcus gattii*	Identification of the etiological agent of cutaneous cryptococcosis in an immunocompetent patient	[12]
	Microsatellites	*Candida tropicalis*	Molecular typing of the etiological agent of nosocomial fungemia and hepatosplenic fungal infections in patients with cancer	[13]
	VNTR	*Rasamsonia argillacea*	Identification in cystic fibrosis patients	[14]
	Microsatellites	*Aspergillus terreus* species-complex	Study of genetic diversity of isolates of the etiologic agent of invasive aspergillosis in immunocompromised individuals	[15,16]

	Microsatellites	*Trichophyton rubrum*	Study of genetic diversity of the etiological agent of tinea unguium, tinea pedis, and tinea corporis	[17]
	Minisatellites	*Cryptococcus neoformans*	Study of genetic diversity and variation in antifungal susceptibility profiles	[18]
	Microsatellites	*Candida parapsilosis*	Genotyping of clinical isolates of the etiological agent of nosocomial infections	[19]
	VNTR	*Aspergillus fumigatus*	Study of clinical strains and environmental fungal aerocontamination to prevent invasive aspergillosis infections in hospital	[20]
	Minisatellites	*Candida parapsilosis* species-complex	Molecular identification of isolates for epidemiological studies and the establishment of appropriate therapies and prophylactic measures	[21]
	Minisatellites	*Cyberlindnera fabianii*	Study of a fungaemia outbreak in preterm neonates	[22]
	Minisatellites	*Enterocytozoon bieneusi*	Genotyping approach for potential common source of the etiological agent of *E. bieneusi* microsporidiosis in hematology unit	[23]
	Microsatellites	*Sporothrix globosa*	Study of genetic diversity and population structure	[24]
	VNTR	*Madurella mycetomatis*	Study the genetic diversity, susceptibility to antifungal agents monitor any potential outbreaks of the etiological agent of mycetoma	[25–27]
	VNTR	*Aspergillus fumigatus*	Investigation of the relationships between clinical and environmental isolates during major demolition work in a French hospital	[28]
	Minisatellites	*Candida kefyr*	Study of prevalence, antifungal drug susceptibility and genotypic heterogeneity	[29]
	Microsatellite	*Candida glabrata*	Study of pathogenicity, virulence, or drug resistance traits	[30]
	Microsatellite	*Pneumocystis jirovecii*	Genotyping of the etiological agent of severe respiratory infections in immunocompromised patients.	[31]
	Microsatellite	*Candida auris*	Microsatellite typing during a emergence of in Brazil in a COVID-19 intensive care unit	[32]
	VNTR	*Candida auris*	Study of the prevalence and emerging status of multidrug-resistant fungi and their mortality	[33]
	VNTR	*Aspergillus fumigatus*	Molecular epidemiology of in chronic pulmonary aspergillosis patients	[34]

	Microsatellite	*Candida auris*	Colonization and transmission dynamics among chronic respiratory diseases patients hospitalized in a chest hospital	[35]
	Microsatellite	*Candida parapsilosis*	Study of genetic diversity of the etiological agent of candidemia among hospitalized pediatric patients	[36]
Animal health	VNTR	*Trichoderma pseudokoningii*	Fungi detection on pulmonary infection in tuberculous dairy cattle	[37]
	VNTR	*Aspergillus* spp.	Study of genetic diversity and antifungal susceptibility of isolates from avian farms	[38]
	VNTR	*Aspergillus fumigatus*	Study of pathogenicity in aerosol-challenged chickens (*Gallus gallus*)	[39]
	Microsatellites	*Trichophyton mentagrophyton*<br>*Microsporum gypseum*<br>*Microsporum canis*	Detection for rabbit-derived dermatophytes	[40]
	Microsatellites	*Beauveria* spp.<br>*Metarhizium* spp.	Specific diversity of the entomopathogenic fungi in Mexican agricultural soils	[41]
	Microsatellites	*Beauveria bassiana*	Microsatellite markers to monitor a commercialized isolate of the entomopathogenic fungus from different environments	[42]
	VNTR	*Aspergillus fumigatus*	Procedures and techniques for aspergillosis diagnosis of in birds	[43]
	Microsatellites	*Metarhizium* spp.	Community composition and population genetics of insect pathogenic fungi from soils of a long-term agricultural research system	[44,45]
	Minisatellites	*Malassezia pachydermatis*	Study of the genetic diversity of the etiological agent of otitis and dermatitis on pets	[46]
	Minisatellites	*Enterocytozoon bieneusi*	Molecular detection of the etiological agent of microsporidiosis	[47,48]
	Minisatellites	Marine fungi	Study of the diversity of culturable mycobiota associated with the Mediterranean	[49]
	Microsatellites	*Pseudogymnoascus destructans*	Mating type determination of the etiological agent of white-nose disease in bats	[50]
	Minisatellites	*Beauveria bassiana*<br>*Metarhizium anisopliae*	Efficacy of different entomopathogenic fungal isolates against four key stored-grain beetle species	[51]
Human and animal health	VNTR	*Aspergillus flavus*	Study of genetic structure of populations in human and avian isolates	[52]
	Microsatellite	*Microsporum canis*	Development and validation of method for tracing infections	[53]

Plant health	Microsatellite	*Macrophomina phaseolina*	Molecular identification of the phytopathogen	[54]
	Minisatellites	*Hymenoscyphus pseudoalbidus*	Study of population structure of and its genetic relationship to *H. albidus*	[55]
	VNTR	*Hypoxylon pulicicidum*	Characterization of a pantropical insecticide-producing endophyte	[56]
	Minisatelitte	*Leptosphaeria maculans*	Migration patterns and changes in population biology associated with the worldwide spread of the oilseed rape pathogen	[57]
	Microsatellite	*Monilinia fructicola*	Study of genetic population structure of the etiological agent of brown rot in peach trees	[58]
	VNTR	*Mycosphaerella fijiensis*	Study of genetic diversity of the etiological agent of black sigatoka in banana	[59]
	Microsatellite	*Erysiphe necator*	Study of population structure in North America and Europe	[60]
	Microsatellite	*Geosmithia morbida*	Study of the genetic diversity of the etiological agent of thousand canker disease in black walnut (*Juglans nigra*)	[61]
	Minisatellites	*Ustilago scitaminea*	Study of the genetic diversity of the etiological agent of sugarcane smut	[62]
	Minisatellites	*Puccinia triticina*	Study of population structure, pathological and molecular characterization of the etiological agent of slow leaf rusting in wheat	[63,64]
	Microsatellite	*Plasmopara viticola*	Characterization of native and introduced populations of the etiological agent of grapevine downy mildew	[65]
	Microsatellite	*Ceratocystis fimbriata*	Analysis of microsatellite markers in the genome of the plant pathogen	[66,67]
	Microsatellite	*Peronospora tabacina*	Ten polymorphic microsatellite loci identified from a small insert genomic library for the tobacco pathogen	[68]
	Minisatellite	*Botrytis cinerea*	Comparison of populations isolated from two open-field cultivated host plants	[69,70]
	Microsatellite	*Puccinia* spp.	Rust fungi forming aecia on barberry (Berberis spp.)	[71]
	VNTR	*Bipolaris oryzae*	Study of genetic diversity, population structure, sexual state. And aggressiveness of the etiological agent of rice brown spot	[72]-[74]
	Microsatellite	*Anisogramma anomala*	Identification of the etiological agent of filbert blight disease on commercial European hazelnut	[75]

	VNTR	*Colletotrichum lindemuthianum*	Molecular genetic characterization of fungal isolates of the etiological agent of anthracnose on common bean (*Phaseolus vulgaris*)	[76]
	Minisatellites	*Puccinia melanocephala*	Development and 88haracterization of the etiological agent of sugarcane brown rust	[77]
	Microsatellite	*Phytophthora* spp.	Development of new polymorphic microsatellite markers for three closely related plant-pathogenic species	[78]
	Microsatellite	*Knoxdaviesia proteae*	Study of genetic diversity and dispersal of the ophiostomatoid fungus within *Protea repens* infructescences	[79]
	Minisatellites	*Erwinia amylovora*	Study of phylogeography and population structure of the etiological agent of fire blight	[80]
	Minisatellites	*Monilinia vaccinii-corymbosi*	Study of population structure, genetic diversity, and the reproductive biology of the Blueberry Pathogen. North Carolina State University.	[81]
	Minisatellites	*Melampsora larici-populina*	Microsatellite analysis of Icelandic populations of the poplar fungal pathogen	[82]
	Microsatellite	*Exserohilum turcicum*	Study of microsatellite and mating type primers for the maize and sorghum pathogen	[83]
	Microsatellite	*Lecanosticta acicola*	Development of microsatellite and mating type markers for the pine needle pathogen	[84]
	Microsatellite	*Fusarium verticillioides*	Genome distribution and validation of novel microsatellite markers and their transferability to other *Fusarium* species	[85]
	Microsatellite	*Gaeumannomyces graminis* var. *tritici*	Analysis of SSR in the genome and development of microsatellite markers	[86]
	Microsatellite	*Colletotrichum* spp	Characterization for the etiological agent of anthracnose in blackberry	[87]
	Minisatellites	*Beauveria bassiana*	Study of the potential of the entomopathogenic fungus as an endophyte in grapevine	[88]
	Microsatellite	*Colletotrichum truncatum*	Study of genetic diversity of the etiological agent of chilli	[89]
	Microsatellite	*Alternaria brassicicola*	Assessment of cross-species transferability and utility of microsatellites as a diagnostic marker	[90]
	Microsatellite	*Magnaporthe grisea*	Study of genetic diversity of the etiological agent of rice blast	[91]
	Microsatellite	*Epichloë festucae*	Development and characterization of nuclear microsatellite markers in the endophytic fungus	[92]

	Microsatellites	*Blumeria graminis* f. sp. *Tritici*	Study of genetic diversity	[93]
	Microsatellite	*Sclerotinia sclerotiorum*	Study of genetic diversity and compatibility groups within and among populations of sunflower	[94]
	Microsatellite	*Stagonosporopsis* spp.	Study of genetic diversity and population structure of the etiological agent of cucurbit gummy stem blight	[95]
	Minisatellites	*Ralstonia solanacearum*	Study of genetic diversity of isolates from three different regions	[96]
	VNTR	*Sarocladium oryzae*	Molecular characterization and distribution of the etiological agent of sheath rot on rice	[97]
	Microsatellite	*Colletotrichum* spp.	Study of the genetic diversity of the endophytic fungi from *Phaseolus vulgaris*	[98]
	Microsatellite	*Villosiclava virens*	Study of population structure of the rice false smut fungus	[99]
	Microsatellite	*Ganoderma boninense*	Identification and of the etiological causal agent of oil palm basal stem rot disease	[100]
	Microsatellite	*Neofabraea* spp.	Identification and characterization of etiological agent of bull's eye rot on apple	[101]
	VNTR	*Pseudocercospora fijiensis*	Study of genetic diversity of the etiological agent of banana black Sigatoka	[102]
	Minisatellites	*Togninia minima*	Study of genetic diversity in Iranian populations of the etiological agent of leaf stripe disease on grapevines	[103]
	Minisatellite	*Trichaptum abietinum*	Study of population structure of the wood-decay fungus	[104]
	Microsatellite	*Fusarium oxysporum*	Evolution of nine microsatellite loci in the fungus	[105]
	Microsatellite	*Diaporthe sp.*<br>*Diaporthe infecunda*<br>*Diaporthe phaseolorum*	Identification of endophytic fungi isolated from common bean (Phaseolus *vulgaris* L.)	[106]
	Microsatellites	*Microbotryum saponariae*	Polymorphic microsatellite markers for the tetrapolar anther-smut fungus based on genome sequencing	[107]
	VNTR	*Bipolaris sorghicola*	Genetic diversity of fungal pathogen infecting *Sorghum bicolor*	[108]
	Microsatellites	*Stagonosporopsis* spp.	Study of genetic structure and population dynamics of gummy stem blight fungi in watermelon fields	[109]
	Microsatellites	*Colletotrichum gloeosporioides*	Study of genetic diversity and population structure	[110,111]
	VNTR	*Rhizoctonia solani*	Study of genetical diversity among isolates causing sheath blight on rice	[112]

	Microsatellites	*Fusarium virguliforme*	Development and characterization of microsatellite markers and their utility within clade 2 of the *Fusarium solani* species complex	[113]
	Microsatellites	*Ustilaginoidea virens*	Analysis of SSR and the development of microsatellite markers	[114]
	VNTR	*Fusarium culmorum*	Study of population structure and mycotoxin potential of the wheat crown rot and head blight pathogen	[115]
	Microsatellites	*Fusarium oxysporum* f. sp. *lentis*	Study of population genetic structure of the etiological agent of lentils wilt	[116]
	Minisatellites	*Zymoseptoria tritici*	Study of the population structure of the etiological agent of Septoria tritici blotch on wheat	[117]
	Microsatellites	*Microbotryum* spp.	Co-occurrence among three divergent plant-castrating fungi in the same *Silene* host species	[118]
	Microsatellites	*Thekopsora areolata*	Development of microsatellite markers for the etiological agent of cherry spruce rust	[119]
	Microsatellites	*Puccinia triticina*	Genetic variation between different populations of the etiological agent of wheat leaf rust from different geographic locations and hosts	[120]
	VNTR	*Rhizoctonia oryzaesativae*	Molecular characterization of etiological agent of aggregate sheath spot on rice	[121]
	Microsatellites	*Sclerotinia sclerotiorum*	Microsatellite loci for isolates collected from ten locations in Bangladesh and one in Ohio, USA	[122]
	Minisatellites	*Amylostereum areolatum*	Study of the genetic diversity of the fungal symbiont of the invasive woodwasp	[123]
	Microsatellites	*Verticillium dahliae*	Study of population structure in the clonal fungus	[124]
	Microsatellites	*Tilletia indica*<br>*Ustilago segetum tritici*	Development and validation of microsatellite markers for Karnal bunt and loose smut of wheat from related fungal species	[125]
	Microsatellites	*Aspergillus flavus*	Use of microsatellite markers to assess the competitive ability of nontoxigenic strains in studies on biocontrol of aflatoxins in maize	[126]
	VNTR	*Podosphaera xanthii*<br>*Golovinomyces cichoracearum*	Identification of fungal species causing powdery mildew on cucurbits, determination of the genetic and pathogenic variations of the fungi and their sensitivity to major powdery mildew fungicides	[127]
	Microsatellites	*Marssonina brunnea*	Identification and development of microsatellite markers and their applications in two populations	[128]

	Minisatellites	*Pyrenophora semeniperda*	Study of mating system complexity and cryptic speciation in the seed bank pathogen	[129]
	Microsatellites	*Cadophora luteo-olivacaea*<br>*Colletotrichum fioriniae*<br>*Seimatosporium vitis-vinifera*<br>*Truncatella angustata*	Identification and pathogenicity of lignicolous fungi associated with grapevine trunk diseases	[130]
	Microsatellites	*Alternaria alternata*	Study of the genetic diversity of the etiological agent of saffron corm rot	[131]
	Microsatellites	*Glutinomyces brunneus*	Recombination and local population structure of the root endophytic fungus	[132]
	Microsatellites	*Pyrenophora graminea*	Study of pathogenicity of the etiological agent of barley leaf stripe	[133]
	VNTR	*Phomopsis vexans*	Detection of the etiological agent of leaf blight and fruit rot on eggplant (*Solanum melongena* L.)	[134,135]
	Microsatellites	*Ustilago hordei*	Study of genetic diversity and population structure of the etiological agent of covered smut of barley	[136]
	Microsatellites	*Fusarium* spp.<br>*Rhizoctonia solani*<br>*Alternaria alternata*<br>*Curvularia coatesiae*	Population structure and genetic diversity of the etiological agent of rice seedling blight	[137]
	Microsatellites	*Puccinia striiformis* f. sp. *tritici*	Development and characterization of novel microsatellite markers and their transferability in *Puccinia* species	[138]
	VNTR	*Erwinia amylovora*	Molecular characterization of strains and biocontrol efficacy of *Bacillus* spp. and *Pseudomonas brassicacearum* antagonists	[139]
	Minisatellites	*Beauveria bassiana*<br>*Metarhizium anisopliae*	Identification of entomopathogenic fungi from agricultural and forestry crops	[140]
	Microsatellites	*Puccinia triticina*<br>*Puccinia recondita* f. sp. *secalis*	Study if utility and informativeness in genetic studies of brown rust fungi on wheat, triticale, and rye	[141]
	Microsatellites	*Racodium therryanum*	New microsatellite markers for the population studies of the etiological agent of snow blight	[142]
Fungal diversity	Minisatellites	*Leptosphaeria maculans*	Study of population structure, reproductive behavior and virulence factors of a fungal phytopathogen	[143–147]

	Minisatellites	*Diversispora* sp	Study of inter-and intrasporal nuclear ribosomal gene sequence variation within one isolate of arbuscular mycorrhizal fungus	[148]
	Microsatellite	*Protoparmeliopsis muralis*	Development of microsatellite markers in a lichenized Ascomycete	[149]
	Microsatellite	*Fusarium oxysporum*<br>*Fusarium udum*	Cross-species transferability and assessment of genetic diversity	[150]
	Minisatellite	*Saccharomyces cerevisiae*	Study of genetic diversity of isolates	[151]
	Minisatellites	*Verticillium albo-atrum*	Study of frequency and distribution of microsatellites in the whole genome	[152]
	Minisatellite	*Mycosphaerella fijiensis*	Study of genetic diversity and mating-type of the etiological agent of black leaf spot of bananas	[153]
	Minisatellites	*Orpinomyces* sp.	Study of the evolutionary history of a anaerobic fungus strain acting as a plant biomass degrader	[154]
	Minisatellites	*Magnaporthe oryzae*	Study of the genetic diversity of the fungal pathogen	[155,156]
	Microsatellite	*Valsa malicola*	Study of genetic diversity of isolates	[157]
	VNTR	*Aspergillus fumigatus*	Study of genetic diversity and susceptibility to itraconazole in isolated from avian farms	[158]
	Minisatellite	*Microbotryum lychnidis-dioicae*	Study of genetic diversity and mating-type of the etiological agent of anther-smut fungus	[159]
	Minisatellites	*Puccinia striiformis*	Molecular identification of somatic recombination of etiological agent of stripe rust	[160]
	Microsatellite	*Puccinia psidii*	Study of genetic population structure	[161]
	Minisatellite	*Colletotrichum lentis*	Study of genetic diversity and pathogenicity of the etiological agent of lentil anthracnose	[162]
	VNTR	*Pyrenophora semeniperda*	Characterization and variation of Mating-type Locus	[163]
	Microsatellite	*Aspergillus flavus*	Characterization of nonaflatoxigenic strains	[164]
	Microsatellite	*Epichloë typhina*<br>*Epichloë clarkii*	Genetic evidence for reproductive isolation among sympatric endophytes	[165]
	Microsatellite	*Pyrenophora tritici-repentis*	Study of genetic structure of populations	[166]
	Minisatellite	*Ophiocordyceps sinensis*	Study of genetic diversity of the Chinese caterpillar fungus	[167]
	Minisatellites	*Chondrostereum purpureum*	Study of interfertility and genetic variability among European and North American isolates	[168]

	Minisatellite	*Epichloë* spp.	Identification, ecological evaluation and phylogenetic analysis of non-symbiotic endophytic fungi colonizing timothy grass and perennial ryegrass grown in adjacent plots	[169]
	VNTR	*Acremonium masseei*	Identification of Insect-Deterrent Metabolites from a Saprophytic Fungus	[170]
	Microsatellite	*Fusarium oxysporum*<br>*Fusarium avenaceum*<br>*Fusarium poae*	Comparative analysis of polymorphism of microsatellite markers in several species of *Fusarium*	[171]
	Minisatellites	*Leptosphaeria maculans*	Study of the distribution of mating-type alleles and genetic variability in field populations	[172]
	Microsatellite	*Aspergillus flavus*	Study of genetic diversity and resistance mechanism analysis of voriconazole-resistant isolates	[173]
	Microsatellite	*Aspergillus fumigatus*	Clonal expansion of environmental triazole resistant	[174]
	Microsatellites	*Usnea florida*<br>*Usnea subfloridana*	Differentiation between apotheciate between species	[175]
	Microsatellites	*Aspergillus fumigatus*	Molecular epidemiology of azole-resistant fungi in sawmills of eastern France by genotyping	[176]
	Microsatellites	*Ceratocystis*	Quantification of outcrossing events in haploid fungi	[177]
	Microsatellites	*Rhizopus arrhizus*	Study of population structure	[178]
	MInisatellites	*Colletotrichum lentils*	Study of mating incompatibility genes of the etiological agent of anthracnose in lentil	[179]
Food and beverage	Minisatellite	*Agaricus bisporus*	Study of repetitive DNA elements in the button mushroom	[180]
	Microsatellite	*Tuber aestivum*	Identification of polymorphic microsatellite markers in the Burgundy truffle	[181]
	Minisatellites	*Amanita ponderosa*	Molecular evaluation of fungal strains living in association with these mushrooms in the southwestern Iberian Peninsula	[182]
	Microsatellite	*Volvariella volvacea*	Microsatellites in the genome of the edible mushroom	[183]
	Minisatellites	*Torulaspora delbrueckii*	Study of molecular typing of yeast strains being applied in the winemaking process	[184]
	Microsatellite	*Lachancea thermotolerans*	Study of genetic diversity and population structure of wine-associated isolates	[185]
	Microsatellite	*Agaricus bisporus*	Study of genetic diversity among commercial and wild	[186]
	Minisatellites	*Saccharification fungi*<br>*Makgeolli* yeasts	Distribution of Autochthonous Yeast Strains Isolated from Different Regional Makgeolli, a Korean Traditional Fermented	[187]

			Rice Wine and Their Biochemical and Genetical Characterization	
	VNTR	*Fusarium graminearum*	Study of population structure and trichothecene mycotoxin profiling from corn and wheat	[188]
	Microsatellite	*Tricholoma matsutake*	Study of genetic diversity and population structure of matsutake mushroom	[189]
	Minisatellites	*Tuber bellonae*	Morphological, physico-chemical and molecular investigations on truffle from Basilicata-Italy	[190]
	VNTR	*Lentinula edodes*	Study of mtDNA variation and their application as genetic marker	[191]
	Microsatellites	*Didymella pisi*	Study of genetic diversity and population structure of associated with ascochyta blight of dry pea	[192]
	Microsatellites	*Tuber magnatum*	Study of genetic diversity in white truffles	[193]
	Microsatellites	*Penicillium digitatum*	Microsatellite characterization and marker development for the etiological agent of green mold of citrus	[194]
	Microsatellites	*Agaricus bisporus*	Identification of commercial cultivars from China	[195]
	VNTR	*Saccharomyces cerevisiae*	Yeast inoculation practices influence the microbial communities of barrel-fermented Chardonnay wines	[196]
	Minisatellites	*Leccinum* sp.	Morphological and Molecular characterization of the ectomycorrhizal symbionts and edible mushrooms	[197]
Lichens	Microsatellites	*Lobaria pindarensis*	Study of genetic diversity of the lichen fungus in the Himalayas	[198]
	Microsatellites	Lobarina scrobiculata	Study of genetic diversity of the lichen fungus in the Iberian Peninsula and Europe	[199]
	Microsatellites	*Rhizoplaca melanophthalma*	Characterization of the cosmopolitan lichen-forming fungus	[200]
	Microsatellites	*Bactrospora dryina*	Study of population structure	[201]
	Microsatellites	*Usnea antarctica*<br>*U. aurantiacoatra*	Species delimitation in Antarctic *Usnea* subgenus *Neuropogon*	[202]
	Microsatellites	*Usnea subfloridana*	Study of genetic diversity of the widespread epiphytic lichen and comparison of among different populations	[203]
	Microsatellites	*L. immixta*<br>*L. macaronesica*	Deep divergence between island populations in lichenized fungi	[204]
Medicinal products	Microsatellite	*Agaricus subrufescens*	Development of polymorphic microsatellite markers for the medicinal mushroom	[205]

	Minisatellites	*Ganoderma applanatum*<br>*Fomitopsis pinicola*	Study of intraspecies genetic variability, mating compatibility and clonality in two medicinal mushrooms	[206]
Extreme environments	Minisatellites	*Leucosporidium* spp.<br>*Pseudogymnoascus* spp.<br>non-identified Ascomycota NIA6<br>*Metschnikowia* sp.<br>*Penicillium* spp.	Identification of fungi from admiralty bay (King George Island, Antarctica) soils and marine sediments	[207]
Soil	Microsatellites	*Rhizopogon*	Vertical partitioning between sister species on mesic and xeric sites in an interior Douglas-fir forest	[208]
	Minisatellites	Hypholoma lateritium	Identification of fungi isolated from coarse woody debris, the forest floor, and mineral soil in a deciduous forest	[209]
	Ministellites	*Rhizoscyphus*	Novel root-fungus symbiosis in Ericaceae: sheathed ericoid mycorrhiza formed by a hitherto undescribed basidiomycete with affinities to Trechisporales	[210]
	Microsatellites	*Rhizopogon vinicolor*<br>*R. vesiculosus*	Study of population genetic structure between two sympatric sister-species of ectomycorrhizal fungi	[211]
	Microsatellites	*Tulasnella*	Phylogenetic and microsatellite markers for mycorrhizal fungi associated with Australian orchids	[212]
	Microsatellites	*Trichophyton ajelloi*	Study of intra-species genetic relatedness in strains isolated from various soil samples	[213]
	Microsatellites	*Rhizopogon kretzerae*<br>*R. salebrosus*	Microsatellite primers for the fungi for 454 shotgun pyrosequencing	[214]
	Microsatellites	*Laccaria* sp	Mitochondrial microsatellite markers for the Australian ectomycorrhizal fungus	[215]
	Microsatellites	Laccaria amethystina<br>Laccaria laccata	Genetic dynamics and ecological functions of the pioneer ectomycorrhizal fungi in a volcanic desert on Mount Fuji	[216]
	Microsatellites	*Pisolithus tinctorius sensu stricto*	Study of the genetic structure in different populations	[217]
	Minisatellites	*Cenococcum geophilum*	Study of genetic diversity and pathogenicity genes of the symbiont of the mycorrhizal species	[218]
	Minisatellites	*Aspergillus flavus*	Study of diversity of isolates from corn and soil in three different Tuscany areas treated with the biological product AF-X1	[219]

	Microsatellites	*Phialocephala fortinii s.l.*<br>*Acephala applanata* species-complex	Study of host preference of root endophytes of three european tree species	[220]
Fresh water ecosystems	Minisatellites	*Candida* spp.<br>*Cryptococcus* spp.<br>*Williopsis saturnus*<br>*Hanseniaspora* spp.<br>*Rhodotorula mucilaginosa*<br>*Saccharomyces cerevisiae*<br>*Torulaspora* spp.<br>*Tricosporon* spp.<br>*Yarrowia lypolitica*	Study of yeast diversity associated to sediments and water from two Colombian artificial lakes	[221]
	Microsatellites	*Blastomyces dermatitidis*<br>*Blastomyces gilchristii*	Study of genetic diversity of fungi from freshwater drainage basins	[222]
Salt water ecosystems	Microsatellites	*Penicillium lusitanum*	Study of the diversity of fungi isolated from marine environments	[223]
	Microsatellites	*Neoascochyta fuci*<br>*Paraconiothyrium salinum*	Study of the diversity of marine fungi	[224]
	Microsatellites	*Paralulworthia halima*<br>*Remispora submersa*<br>*Zalerion pseudomaritima,*	Study of the diversity of marine fungi associated with wood baits in the estuary Ria de Aveiro	[225]
	Microsatellites	*Cladosporium rubrum*<br>*Hypoxylon aveirense*	Study of the diversity of estuarine environment	[226]

Supplementary Table S6. Molecular markers in fungi: an overview of manuscripts published in the last 10 years using SNP.

Importance	Fungal identification	Purpose	Reference
Human health	*Aspergillus fumigatus*	Detection, identification and genotyping of the fungi in patients with invasive aspergillosis	[1–3]
	Blastomyces gilchristii	Identification of the etiological agent of blastomycosis	[4]
	Cladophialophora bantiana<br>*C. immunda*<br>*C. devriesii*	Identification of the etiological agent of human infection	[5]
	Exophiala dermatitidis<br>*E. oligosperma*<br>*E. spinifera*<br>*E. xenobiotica*<br>*E. jeanselmei*	Detection and identification of opportunistic *Exophiala* species causing mycosis	[6]
	Exserohilum rostratum	Detection and identification of the etiological agent of an outbreak of fungal meningitis and other infections	[7]
	Paracoccidioides spp.	Study of the genetic diversity of the etiological agente of paracoccidioidomycosis	[8,9]
	Rhizopus<br>*Mucor*	Identification of the etiological agent of mucormycosis	[10]
	Sarocladium kiliense	Determination of the origin of multinational outbreak of bloodstream infections	[11]
	Aspergillus fumigatus	Detection, identification and study of azole resistance in samples from patients with invasive aspergilosis	[12,13]
	Aspergillus fumigatus	Study of fungal natural product gene cluster	[14]
	Candida auris	Study of the genetic diversity and genomic epidemiology of the emerging human fungal pathogen	[15–17]
	Candida glabrata	Study of the genetic diversity of the etiological agent of candidiasis	[18]
	Candida albicans<br>*Cryptococcus neoformans*	Study of population genetics in human pathogenic fungi	[19]
	Trichophyton rubrum<br>*T. violaceum*<br>*T. soudanense*	Study of the genetic diversity and genotyping of the etiological agent of skin, nail and hair infections	[20]

	T. kuryangei<br>*T. megninii*		
Animal health	*Arthrobotrys oligospora*	Study of the genetic diversity and population structure of the nematode-trapping fungus	[21]
	Grosmannia clavigera<br>*Leptographium longiclavatum*<br>*Ophiostoma montium*	Study of the genetic diversity and relatedness in fungi associated with the mountain pine beetle	[22–24]
	Pseudogymnoascus destructans<br>*Pseudogymnoascus spp.*	Distintiction between Pseudogymnoascus species	[25]
	Aspergillus fumigatus<br>*Aspergillus tubingensis*<br>*Aspergillus uvarum*	Identification of the etiological agents of canine sino-nasal aspergillosis	[26]
	Ophidiomyces ophiodiicola	Detection of the fungus associated with snake fungal disease	[27]
	Batrachochytrium dendrobatidis	Study of the genetic diversity of the chytrid fungi causing global amphibian declines	[28]
	Ophiocordyceps unilateralis	Study of the genetic diversity of the entomopathogenic fungi responsible for zombie-ants	[29]
Human and animal health	*Pythium insidiosum*	Identification and genotyping etiological agent of pythiosis in humans and animals	[30]
Plant health	*Ophiognomonia clavigignenti-juglandacearum*	Study of the population structure of the etiological agent of butternut canker	[31]
	Leptosphaeria maculans	Study of genetic diversity, virulence and reproductive behaviour of the an important phytopathogen	[32–34]
	Podosphaera plantaginis	Study of the genetic diversity of an obligate fungal pathogen of Plantago lanceolata	[35]
	Heterobasidion annosum ss	Study of virulence factors	[36]
	Magnaporthe oryzae	Study of the genetic diversity and virulence factors of the etiological agent of rice blast	[37–40]
	Pyrenophora teres	Study of the genetic diversity of the etiological agent of barley net blotch	[41,42]
	Ganoderma<br>*Inonotus*<br>*Phellinus*<br>*Ceriporia*<br>*Schizophyllum*<br>*Phanerochaete*<br>*Pleurotus*<br>*Leucoagaricus*	Study of genetic diversity of wood-decay macro-fungi associated with declining arid zone trees	[43]

	Ustilaginoidea virens	Study of the genetic diversity and population structure of the etiological agent of false smut on rice	[44]
	Verticillium longisporum	Distinction between pathogen and apathogen species	[45]
	Rhizoctonia solani	Study of the genetic diversity of a broad host-range pathogen	[46]
	Zymoseptoria tritici	Study of the genetic diversity, virulence and antimicrobial resistance in the plant pathogenic fungus	[47–49]
	Alternaria solani	Characterization and detection of mutations associated with resistance to succinate dehydrogenase-inhibiting fungicides	[50]
	Verticillium dahliae	Study of genetic diversity, population structure and mating-type locus of a plant-pathogenic fungus	[51,52]
	Melampsora larici-populina	Study of the genetic diversity and virulence factors of the poplar rust fungus	[53]
	Alternaria alternata	Study of the genetic diversity of genes involved in toxin production in the etiological agent of black and brown spot diseases	[54]
	Pyrenophora teres<br>*Sphaerulina musiva*	Genotyping of plant-pathogenic fungi	[55]
	Pseudoperonospora cubensis<br>*Pseudoperonospora humuli*	Study of the genetic variation between obligate plant pathogens	[56]
	Melampsora lini	Study of genetic diversity and virulence of the etiological agent of flax rust	[57]
	Parastagonospora nodorum	Study of virulence factors in the etiological agent of septoria nodorum blotch on wheat	[58]
	Colletotrichum fructicola	Study of genetic diversity of the fungal pathogen on tea-oil trees	[59]
	Calonectria pseudonaviculata	Distinction of the etiological agent of sarcococca blight from two different plant species	[60]
	Rhynchosporium commune	Study of multilocus resistance evolution to azole fungicides	[61]
	Fusarium graminearum species complex	Distinction of closely related Fusarium species causing head blight fungi	[62]
	Puccinia striiformis f. sp. *tritici*	Study of genetic diversity, population structure, and virulence in the etiological agent of wheat stripe rust	[63,64]
	Monilinia yunnanensis<br>*Monilinia polystroma*<br>*Monilinia fructicola*	Identification of species associated with brown rot of cultivated apple and pear fruit	[65]
	Fusarium oxysporum f. sp. lycopersici	Distinction of races in plant pathogenic fungi	[66]

	Diaporthaceae<br>Botryosphaeriaceae	Study of the genetic diversity of fungal families associated with grapevine	[67]
	Phellinus noxius	Study of the genetic diversity of the etiological agent of root rot in trees	[68]
	Rhynchosporium commune	Study of the genetic diversity	[69]
	Fusarium graminearum	Study of genetic diversity and host adaptation	[70]
	Blumeria graminis f. sp. *tritici*	Study of genetic diversity and virulence factors in the etiological agent of wheat and rye powdery mildew fungus	[71]
	Fusarium proliferatum<br>*Fusarium solani*<br>*Fusarium brachygibbosum*<br>*Fusarium oxysporum*<br>*Fusarium verticillioides*	Identification of species associated with date palm diseases (chlorosis, necrosis and whitening)	[72]
	Microbotryum lychnidis-dioicae<br>*Microbotryum silenes-dioicae*	Study of the genetic diversity of castrating anther-smut fungi	[73,74]
	Bipolaris sorokiniana	Study of the genetic diversity and virulence factors of the etiological agent of multiple diseases on wheat and barley	[75]
	Hymenoscyphus fraxineus	Study of the structure of the etiological agent of ash dieback	[76]
	Hemileia vastatrix	Study of genetic diversity and recombination in the etiological agent of coffee leaf rust	[77]
	Colletotrichum kahawae	Study of genetic diversity and evolution of the etiological agent of coffee berry disease in Arabica coffee	[78]
	Puccinia triticina	Study of genetic diversity the etiological agent of leaf rust on wheat	[79]
	Plasmopara viticola	Study of mating-type locus in the etiological agent of grapevine downy mildew	[80]
	Fusarium odoratissimum	Study of the genetic diversity of the etiological agent of fusarium wilt in bananas	[81]
	Tilletia indica	Study of the genetic diversity of the etiological agent of karnal bunt on wheat	[82]
	Setosphaeria turcica	Study of the genetic diversity and population structure of the etiological agent of northern leaf blight in sorghum	[83]
Genetic diversity	*Neurospora crassa*	Study of the genetic diversity	[84]
	Glomeromycota	Study of vegetative compatibility, genetic diversity, and population structure in arbuscular mycorrhizal fungi	[85–90]
	Fusarium fujikuroi	Study of the ability to produce fumonisin	[91]
	Parasite fungi	Study of the genetic diversity	[92]

	Armillaria cepistipes	Study of the population genetic structure	[93]
	Fusarium culmorum	Study of virulence and comparison between species	[94]
	Aspergillus section *versicolores*	Detection of sterigmatocystin producing fungal strains	[95]
Food and beverage	*Lentinula edodes*	Genotyping and genetic linkage maps of the Shiitake mushroom	[96]
	Aspergillus flavus	Study of the genetic diversity and aflatoxin production	[97]
	Tuber melanosporum	Study of the genetic diversity of the Périgord black truffle	[98]
	Penicillium digitatum<br>*Penicillium expansum*	Study of the genetic diversity in two postharvest pathogens from fruits	[99]
	Agaricus bisporus	Study of the of genetic diversity and population structure in the germplasm of button mushroom	[100]
Lichens	*Xanthoparmelia* genera	Study of the evolution of the lichenized fungi	[101]
	Letharia spp.	Distinction between species included in the same species complex	[102]
	Cetradonia linearis	Study of population structure of a endangered, endemic lichen	[103]
	Tremalla spp.	Identification of fungi present in the cortex of wolf lichens	[104]
	Rhizoplaca melanophthalma	Study of the role of hybridization in lichen-forming fungi	[105]
Medicinal products	*Ophiocordyceps sinensis*	Study of the genetic diversity of an important medicinal fungus	[106,107]
Extreme environments	*Psychrophila spp.*<br>*Tetracladium*	Identification of psychrophilic fungi from the world's roof (alpine glaciers)	[108]
	Aspergillus fumigatus	Study of the genetic diversity of isolates from air and surfaces of the international space station	[109]
	Agaricus bisporus	Study of genetic diversity, evolution and adaptation to extreme environment	[110]
Soil	*Rhizophagus irregularis*	Study of the genetic diversity of the mycorrhizal fungus	[111,113]
	Tricholoma populinum	Study of the genetic diversity of a ectomycorrhizal fungus	[114]
	Suillus brevipes	Distinction between populations of a symbiotic fungus	[115]
	Rhizophagus irregularis<br>*Gigaspora margarita*	Study of the genetic diversity of mycorrhizal fungi	[116]
	Arbuscular mycorrhizal fungi	Study of the genetic diversity and mating type	[117]
	Fungal community	Characterization of fungi from Arabidopsis root microbiome	[118]
	Suillus luteus	Study of adaptation in the mycorrhizal fungus driven by soil heavy metal contamination	[119]
Enzyme production	*Trichoderma reesei*	Identification of a hyper-cellulolytic mutant fungi	[120]

Supplementary Table S7. Molecular markers in fungi: an overview of manuscripts published in the last 10 years using InDels.

Importance	Molecular marker	Fungal identification	Purpose	Reference
Human health	InDels	*Cryptococcus neoformans*	Study of the genetic diversity and virulence factors	[1–3]
	InDels	*Aspergillus fumigatus*	Study of the genetic diversity and recombination rates among of the etiological agent of invasive aspergillosis	[4–6]
	InDels	*Enterocytozoon bieneusi*	Study of the genetic diversity and population structure of the agent causing diarrhea and enteric disease	[7,8]
	InDels	*Candida albicans*	Study of the genetic diversity	[9]
	InDels	*Paracoccidioides brasiliensis*<br>*Paracoccidioides lutzii*	Distinction of the etiological agents of paracoccidioidomycosis	[10]
	INdels	*Trichophyton spp.*<br>*Microsporum spp.*<br>*Epidermophyton spp.*	Identification of the etiological agents of superficial mycosis	[11]
	InDels	*Cryptococcus gattii*	Study of microevolutionary traits and comparative population genomics of the emerging pathogenic fungus	[12]
	InDels	*Malassezia arunalokei*	Study of the genetic diversity from isolates from seborrheic dermatitis patients and healthy individuals	[13]
	InDels	*Cryptococcus deuterogattii*	Study of genetic diversity and and drug resistance	[14]
	InDels	*Curvularia tamilnaduensis*<br>*Curvularia coimbatorensis*	Identification of new etiological agents of fungal keratitis	[15]
	InDels	*Candida auris*	Diagnostic and identification of the pathogen	[16]
	InDels	*Trichophyton persicum*<br>*Trichophyton spiraliforme*	Identification of new etiological agents of highly inflammatory tinea	[17]
	InDels	*Mucor irregularis*<br>*Mucor hiemalis*<br>*Lichtheimia corymbifera*<br>*Rhizopus arrhizu*	Characterization of mitogenomes from Mucorales species and insights into pathogenicity	[18]
Animal health	InDels	*Batrachochytrium dendrobatidis*	Study of the genetic diversity and phylogeny of the etiological agent of the white-nose syndrome	[19,20]

	InDels	*Tritrichomonas foetus*	Study of the genetic diversity of the etiological agent of tritrichomonosis in different animal hosts	[21]
	InDels	*Sporothrix insectorum*	Study of the mitogenome of the entomopathogenic fungus and comparative mitogenomics in Ophiostomatales	[22]
	InDels	*Beauveria bassiana*	Study of the genetic diversity and virulence	[23]
	InDels	*Trichophyton benhamiae* species complex	Discrimination between the etiological agents of zoonotic tinea corporis and capitis	[24]
Human and animal health	InDels	*Trichophyton tonsurans* *Trichophyton equinum*	Discrimination between species	[25]
	InDels	*Enterocytozoon bieneusi*	Genotyping and study host specificity and its impacts on public health	[26,27]
Plant health	InDels	*Botrytis cinerea*	Study of the genome sequence, genetic diversity and resistance to fungicides	[28–30]
	InDels	*Phlebiopsis gigantea*	Study of the genetic diversity of the etiological agent of white rot of conifer logs and stumps	[31]
	InDels	*Colletotrichum gloeosporioides* species complex	Study of phylogeny and distinction between the species of the complex	[32,33]
	InDels	*Ceratocystis ss*	Distinction between the species of the complex	[34]
	InDels	*Phakopsora pachyrhizi*	Study of the genetic diversity of the etiological agent of soybean rust	[35]
	InDels	*Puccinia striiformis*	Study of the genetic diversity, evolution and virulence factors of the etiological agent of stripe rust	[36–38]
	InDels	*Blumeria graminis f. sp. hordei*	Study of the genetic diversity and comparison between populations	[39]
	InDels	*Pyrenophora tritici-repentis*	Detection of the wheat tan spot pathogen	[40]
	InDels	*Verticillium longisporum*	Study of the genetic diversity of the etiological agent of stem striping in oilseed rape	[41]
	InDels	*Epichloë spp.*	Study of the genetic diversity of a fungal endophyte	[42]
	InDels	*Fusarium graminearum*	Study of the genetic diversity and evolution	[43]
	InDels	*Coleosporium solidaginis* *Coleosporium asterum*	Study of the genetic diversity of the etiological agent of rust in *Solidago*	[44]
	InDels	*Magnaporthe oryzae*	Study of the phylogeny and population structure of the etiological agent of rice blast	[45]

	InDels	*Endoconidiophora resinifera*	Study of the genetic diversity of the etiological agent of blue-stain disease on sapwood	[46]
	InDels	*Pyrenophora semeniperda*	Study of the genetic diversity and mating system types of the seed bank pathogen	[47]
	InDels	*Ascochyta fabae*	Identification of a polyketide synthase gene responsible for Ascochitine biosynthesis	[48]
	InDels	*Fusarium oxysporum f. sp. conglutinans*	Study of the genetic diversity, virulence, race profiling, and comparative genomic analysis of strains infecting cabbages	[49]
	InDels	*Parastagonospora nodorum*	Study of the genetic diversity of the etiological agent of septoria nodorum blotch on wheat	[50]
	InDels	*Fusarium culmorum*<br>*Fusarium graminearum ss*	Distinction between closely related strains and its implication for diagnostic purposes	[51]
	InDels	*Fusarium asiaticum*	Study of the genetic diversity	[52]
	InDels	*Leptosphaeria maculans*	Study of the genetic diversity and population structure of the etiological agent of phoma stem canker in oilseed rape	[53]
	InDels	*Phomopsis longicolla*	Study of the genetic diversity of phomopsis seed decay in soybean	[54]
	InDels	*Podosphaera macularis*	Study of the population of the etiological agent of hop powdery mildew	[55]
	InDels	*Cryphonectria parasitica*	Study of the genetic diversity of the etiological agent of chestnut blight	[56]
Genomic diversity	InDels	*Neurospora crassa*	Study of the genetic diversity	[57]
	InDels	*Pyronemataceae*	Study of the genetic diversity, phylogeny and evolution	[58]
	InDels	*Bolbitiaceae*	Study of phylogenetic relationships in the mushroom family	[59]
	InDels	*Auricularia auricula-judae*	Identification of protoplast-regenerated monokaryotic isolates	[60]
	InDels	*Sclerotinia sclerotiorum*	Study of spontaneous and fungicide-induced genetic diversity	[61]
Food and beverage	InDels	*Zygosaccharomyces sapae*	Identification of new species isolated from Italian traditional balsamic vinegar	[62]
	InDels	*Lentinula edodes*	Construction of the genetic linkage map and mapping quantitative trait loci for vegetative mycelium growth rate, and architecture of fruiting body development	[63–66]
	InDels	*Aspergillus flavus*	Study of the genetic diversity of aflatoxin biosynthesis genes	[67]
	InDels	*Pleurotus eryngii*	Construction of the genetic linkage map and analysis of quantitative trait loci associated with the agronomically traits	[68]

	InDels	*Lentinula edodes*	Study of the genetic diversity and population structure of shiitake mushroom	[69–71]
	InDels	*Aspergillus section Flavi*	Study of the genetic diversity and aflatoxin biosynthesis cluster from isolates from nuts	[72–76]
	InDels	*Aspergillus oryzae*	Study of the genetic diversity of strains used in soy sauce koji fermentation	[77]
	InDels	*Auricularia species*	Study of the genetic diversity and evolution	[78]
	InDels	*Morchella importuna*	Construction of genetic linkage maps	[79]
Lichens	InDels	*Solenopsora spp.*	Study of the genetic diversity and phylogenetics	[80]
	InDels	*Peltigera spp*	Phylogenetic placement, species delimitation, and cyanobiont identity of endangered aquatic lichenized fungus	[81,82]
	InDels	*Umbilicaria decussata*	Study of the genetic diversity of the lichenized fungus collected in polar and non-polar regions	[83]
	InDels	*Strigula spp.*	Reallocation of foliicolous species into six genera	[84]
	InDels	*Sticta spp.*	Study of the evolutionary history of the lichenized fungus	[85]
Medicinal products	InDels	*Tolypocladium inflatum*	Study of genetic diversity of the cyclosporin-producing fungus	[86]
	InDels	*Cordyceps militaris*	Study of the genetic diversity of a medicinal fungus	[87]
	InDels	*Ganoderma lucidum*	Study of genetic diversity and phylogenetic relationships	[88]
	InDels	*Hypsizygus marmoreus*	Study of genetic diversity and phylogeny of a edible mushroom	[89]
	InDels	*Hericium erinaceus*	Study of the genetic diversity of an edible and medicinal mushroom	[90]
	InDels	*Gloeostereum incarnatum*	Construction of a genetic linkage map of an edible and medicinal mushroom	[91,92]
Riparian ecosystems	InDels	*Phytophthora spp.*	Characterization of hybrids	[93–95]
Soil	InDels	*Tricholoma populinum*	Study of the genetic diversity of the Populus-associated ectomycorrhizal fungus	[96]
	InDels	*Diversispora sp.*	Study of the genetic diversity of a arbuscular mycorrhizal fungus	[97]
	InDels	*Gigasporaceae*<br>*Glomeraceae*	Sequencing and comparison of the mitochondrial COI gene from isolates of arbuscular mycorrhizal fungi belonging to different families	[98]
	InDels	*Sieverdingia tortuosa*<br>*Diversispora peloponnesiaca*	Identification of new Diversisporaceae species of arbuscular mycorrhizal fungi	[99]
	InDels	*Dominikia bonfanteae*	Identification of new Glomeraceae species of arbuscular mycorrhizal fungi	[100]

		Glomus atlanticum		
	InDels	*Glomus chinense*<br>*Dominikia gansuensis*	Identification of new Glomeraceae species of arbuscular mycorrhizal fungi from high altitude in the Tibetan Plateau	[101]
Enzyme production	InDels	*Lentinula edodes*	Study of the genetic diversity in genes involved in lignocellulose degradation in Shiitake mushroom	[102]
	InDels	*Arthrographis curvata*<br>*Rhodosporidium babjevae*	Study of new potential fungal lipase producers	[103]
	InDels	*Aspergillus sydowii*	Genome analysis and characterization of two heterologous expressed, non-redundant xylanases	[104]
	InDels	*Trichoderma reesei*	Study of the genetic diversity of the workhorse strains used for industrial production of lignocellulosic enzymes and recombinant proteins	[105]

Supplementary Table S8. Molecular markers in fungi: an overview of manuscripts published in the last 10 years using DNA Barcoding (ACT: actin, Btub: β-tubulin, CAL: calmodulin, COX1; cytochrome c oxidase subunit I, COX2; cytochrome c oxidase subunit II, GPDH: glyceraldehyde-3-phosphate dehydrogenase, ITS: internal transcribed spacer regions of the nrDNA operon, LSU: 28S nrDNA, PGK: phosphoglycerate kinase, RP60S: small ribosomal protein 60S L10, RPB2: RNA polymerase II second largest subunit, SSU: 18S nrDNA, TEF-1α: translation elongation factor 1-alpha, TOP1: topoisomerase I).

Importance	Barcode	Fungal identification	Purpose	Reference
Human health	β-TUB	*Wickerhamomyces anomalus*	Identification of an human opportunistic fungi	[1]
	ITS<br>LSU	*Backusella spp.*<br>*Cunninghamella spp.*<br>*Mucor spp.*<br>*Rhizomucor spp.*<br>*Zygorhynchus moelleri*	Identification of clinical isolates	[2]
	LSU	*Fusarium spp.*<br>*Neocosmospora spp.*	Study of the genetic diversity of fungi associated with chronic rhinosinusitis	[3]
	ITS<br>TEF-1α	*Cunninghamella spp.*	Identification the etiological agent of mucormycosis	[4]
	ACT<br>β-TUB<br>ITS<br>TEF-1α	*Exophiala jeanselmei*	Identification of black yeasts involved in opportunistic disease in humans	[5]
	PGK<br>TOP1	*Fusarium spp.*	Identification of opportunistic pathogens	[6]
	ACT<br>β-TUB<br>RP60S<br>TEF-1α	*Scedosporium apiospermum species-complex*	Identification of species of the etiological agent of human infections	[7]
	β-TUB	*Aspergillus spp.*	Distinction between *Aspergillus* species.	[8]
	ITS	*Epidermophyton spp.*<br>*Microsporum audouinii*<br>*Nannizzia gypsea*<br>*Trichophyton spp.*	Identification of clinical dermatophyte strains	[9]
	ITS<br>TEF-1α	*Candida spp.*<br>*Diutina spp.*	Identification of the agents of invasive fungal infections	[10]

		Pichia spp.<br>*Scedosporium spp.*		
	ITS	*Colletotrichum spp.*<br>*Purpureocillium linacinum*<br>*Aspergillus spp.*<br>*Paracremonium spp.*<br>*Phellinum spp.*	Identification of the etiological agents of fungal keratitis	[11]
	ITS	*Paracoccidioides spp.*	Identification of the etiological agents of paracoccidioidomycosis	[12]
	β-TUB<br>ITS	*Scedosporium spp.*<br>*Lomentospora spp.*	Identification of the etiological agents of invasive disease	[13]
	ITS	*Periconia spp.*	Identification of the etiological agent of corneal mycotic keratitis	[14]
	COX<br>LSU	*Pythium Insidiosum*	Identification of the etiological agent of pythiosis	[15]
Animal health	β-TUB<br>ITS<br>LSU	*Phialophora spp.*	Identification of new species causing diseases in leaf-cutting ants	[16]
	ITS<br>LSU	*Candida cetoniae*<br>*Nematodospora valgi*	Identificaton of new species from the Lodderomyces clade	[17]
	ITS<br>LSU	*Alternaria alternata*<br>*Cladophialophora bantiana*<br>*Curvularia lunata*<br>*Exserohilum rostrata*<br>*Flavodon flavus*<br>*Fusarium solani*<br>*Toxicocladosporium irritans*	Identification of the etiological agents of mycotic rhinitis and sinusitis in horses	[18]
	ITS	*Alternaria spp.*<br>*Aspergillus spp.*<br>*Cladosporium spp.*<br>*Mucor indicus*<br>*Penicillium spp.*<br>*Pericornia sp.*<br>*Sarocladium implicatum*	Identification of guanophilic fungi of bats	[19]

Human and animal health	ITS	*Acremonium spp.*<br>*Alternaria spp.*<br>*Arthrographis kalrae*<br>*Arthropsis hispanica*<br>*Aspergillus spp.*<br>*Aureobasidium pullulans*<br>*Bipolaris spp.*<br>*Blastobotrys spp.*<br>*Candida spp.*<br>*Cryptococcus spp.*<br>*Curvularia spp.*<br>*Cyberlindnera spp.*<br>*Debaryomyces hansenii*<br>*Epidermophyton floccosum*<br>*Exophiala spp.*<br>*Filobasidium uniguttulatum*<br>*Fonsecaea spp.*<br>*Fusarium spp.*<br>*Galactomyces candidus*<br>*Hanseniaspora uvarum*<br>*Histoplasma capsulatum*<br>*Hormographiella aspergillata*<br>*Hyphopichia burtonii*<br>*Hypocrea orientalis*<br>*Kazachstania pintolopesii*<br>*Kluyveromyces spp.*<br>*Kodamaea ohmeri*<br>*Leptosphaeria sen*<br>*Lichtheimia spp.*<br>*Lomentospora prolificans*<br>*Magnusiomyces capitatus*<br>*Medicopsis romeroi*	Detection of human and animal pathogenic fungi	[20]

		Meyerozyma guilliermondii<br>*Meyerozyma spp.*<br>*Microascus cirrosus*<br>*Microsporum spp.*<br>*Millerozyma farinosa*<br>*Mucor circinelloides*<br>*Neoscytalidium dimidiatum*<br>*Paracoccidioides brasiliensis*<br>*Penicillium brevicompactum*<br>*Phialemonium atrogriseum*<br>*Pichia spp.*<br>*Pithomyces spp.*<br>*Purpureocillium lilacinum*<br>*Rasamsonia aegroticola*<br>*Rhinocladiella similis*<br>*Rhizomucor pusillus*<br>*Rhizopus spp.*<br>*Rhodotorula mucilaginosa*<br>*Saccharomyces cerevisiae*<br>*Sarocladium spp.*<br>*Scedosporium spp.*<br>*Scopulariopsis spp.*<br>*Scytalidium cuboideum*<br>*Sporothrix schenckii*<br>*Torulaspora delbrueckii*<br>*Trichoderma spp.*<br>*Trichophyton spp.*<br>*Trichosporon spp.*<br>*Wickerhamomyces anomalus*<br>*Yamadazyma spp.*<br>*Yarrowia lipolytica*		

Plant health	ACT<br>β-TUB<br>CAL<br>ITS<br>LSU<br>RPB2<br>TEF-1α	*Lecanosticta acicola species-complex*<br>*Mycosphaerella spp.*	Identification of species of quarantine importance	[21]
	AAC<br>CDC48<br>Hsp90<br>TEF3	*Nectriaceae*	Identification of Nectriaceae species	[22]
	ITS	*Cronartium spp.*<br>*Diaporthe spp.*<br>*Fusarium spp.*<br>*Gymnosporangium spp.*<br>*Mycosphaerella spp.*<br>*Peronosclerospora spp.*<br>*Phoma spp.*<br>*Phytophthora spp.*<br>*Puccinia spp.*<br>*Tilletia spp.*	Identification of species of quarantine importance	[23]
	ITS	*Ganoderma spp.*<br>*Phellinus noxius*<br>*Rigidoporus microporus*	Identification of basidiomycete fungi in hardwood plantations	[24]
	ITS<br>LSU	*Fusarium fujikuroi species-complex*	Distinction of species within the complex	[25]
	ITS<br>GPDH<br>RPB1<br>TEF-1α	*Bipolaris oryzae*<br>*Curvularia spp.*<br>*Pyricularia oryzae*	Identification of fungal pathogens associated with foliar diseases of wild and cultivated rice	[26]
	ITS<br>SSU	*Fusarium spp.*	Detection and identification of phytopathogens	[27]

	TEF-1α			
	ITS	*Entyloma cosmi*	Identification and phylogenetic analysis of the etiological agent of garden cosmos	[28]
	ITS	*Alternaria longissima*<br>*Curvularia spp.*<br>*Epicoccum sorghinum*<br>*Exserohilum rostratum*<br>*Fusarium thapsinum*	Identification of ascomycetes in sorghum	[29]
	ITS<br>LSU	*Coleosporium spp.*	Identification and phylogenetic analyses of the etiological agent of North American goldenrod rust	[30]
	β-TUB<br>ITS	*Colletotrichum spp.*	Identification of fungal etiological agent of fruit rot	[31]
	β-TUB<br>ITS<br>LSU<br>RPB2	*Allophoma spp.*<br>*Alternaria spp.*<br>*Arezzomyces spp.*<br>*Brunneosphaerella spp.*<br>*Elsinoe spp.*<br>*Exserohilum spp.*<br>*Globoramichloridium spp.*<br>*Neosetophoma spp.*<br>*Neostagonospora spp.*<br>*Nothophoma spp.*<br>*Parastagonospora spp.*<br>*Phaeosphaeriopsis spp.*<br>*Pleiocarpon spp.*<br>*Pyrenophora spp.*<br>*Ramichloridium spp.*<br>*Seifertia spp.*<br>*Seiridium spp.*<br>*Septoriella spp.*<br>*Setophoma spp.*<br>*Stagonosporopsis spp.*<br>*Stemphylium spp.*	Identification of species of phytopathogens	[32]

		Tubakia spp.<br>*Wingfieldomyces spp.*<br>*Zasmidium spp.*		
	ITS	*Uromyces erythronii*	Distribution, infection rates and identification	[33]
	ITS<br>LSU	*Vishniacozyma alagoana*	Identification of a new yeast associated with plants from dry and rainfall tropical forests	[34]
	ITS	*Aspergillus spp.*<br>*Fusarium spp.*	Identification of phytopathogens for disease diagnostics and bio-surveillance	[35]
	ITS	*Dothistroma spp.*	Identification of pathogens on *Pinus* species	[36]
	ITS	*Fusarium equiseti*	Identification of the etiological agent of chilli wilt	[37]
Genetic diversity	ITS	*Tomophagus cattienensis*	Identification of a new species	[38]
	B-TUB<br>CAM<br>ITS<br>TEF 1-α	*Diaporthe spp.*	Study of the genetic diversity and phylogeny	[39]
	ITS	*Annulohypoxylon*<br>*Hypoxylon*	Identification of species within the genera	[40]
	ITS<br>LSU	*Mortierellales spp.*	Study of the genetic diversity and phylogeny of Mortierellomycotina	[41]
	ACT<br>ITS<br>TEF-1α	*Rhizopus microsporus*	Study of fungal diversity and delimitation of the species	[42]
	ITS	*Sporothrix schenckii* species-complex	Study of fungal diversity	[43]
	ITS<br>HSP<br>RPB2<br>TEF-1α	*Serpula species-complex*	Distinction of species within the complex	[44]
	ITS	*Cyathus stercoreus*	Study of the genetic diversity and distribution of the fungus	[45]
	β-TUB<br>ITS<br>TEF-1α	*Neofusicoccum batangarum*	Identification of the etiological agent of scabby canker on *Opuntia ficus-indica*	[46]

	ITS	*Plenodomus biglobosus*	Identification of the etiological agent of blackleg on oilseed rape	[47]
	IGS<br>RPB2<br>TEF-1α	*Fusarium oxysporum f. sp. opuntiarum*	Identification of the etiological agent of stem rot on *Mammillaria painteri*	[48]
	LSU<br>SSU<br>ITS<br>TEF-1α	*Heterosporicola beijingense*	Identification of the etiological agent of leaf spots on *Chenopodium quinoa*	[49]
	β-TUB<br>ITS<br>TEF-1α	*Cadophora luteo-olivacea*<br>*Diaporthe rudis*<br>*Neofusicoccum parvum*<br>*Peroneutypa scoparia*	Identification of the etiological agent of stem blight and dieback on blueberry	[50]
	β-TUB<br>CAM<br>IGS<br>TEF-1α	*Fusarium incarnatum-equiseti* complex-species	Identification of the etiological agent of leaf spot in leafy vegetable crops	[51]
	ITS<br>LSU	*Palmeiromyces chamaeropicola*	Identification of the etiological agent of leaf spot on *Chamaerops humilis*	[52]
	ITS<br>LSU<br>TEF1-α	*Codinaea spp.*<br>*Codinaeella spp.*<br>*Nimesporella spp.*<br>*Stilbochaeta spp.*<br>*Tainosphaeriella spp.*<br>*Xyladelphia spp.*	Phylogenetic Reassessment, Taxonomy, and Biogeography of Codinaea	[53]
	β-TUB<br>ITS<br>LSU<br>SSU<br>RPB2<br>TEF1-α	*Achrochaeta spp.*<br>*Tubulicolla spp.*	Study of the genetic diversity and phylogeny of Dictyochaeta	[54]

	CAM	*Aspergillus spp.*	Study of the genetic diversity and description of one new species from the Gcwihaba Cave (Botswana)	[55]
Food and beverage	LSU	*Candida spp.*<br>*Isatchenkia spp.*<br>*Kazachstania spp.*<br>*Kluyveromyces spp.*<br>*Pichia spp.*<br>*Rhodotorula spp.*<br>*Saccharomyces spp.*	Identification of yeasts from selected bulgarian food products	[56]
	ITS<br>LSU<br>BTUB<br>TEF-1α	*Aureobasidium pullulans*<br>*Cladosporium oxysporum*<br>*Eutypella scoparia*<br>*Penicillium commune*	Identification of fungal species from Taleggio cheese	[57]
	ITS<br>LSU<br>BTUB	*Alternaria solani*<br>*Aspergillus spp.*<br>*Atrotorquata lineata*<br>*Candida spp.*<br>*Clavispora lusitaniae*<br>*Colletothricum gloesporioides*<br>*Cryptococcus spp.*<br>*Debaryomyces hansenii*<br>*Euroticum amstelodami*<br>*Exophiala spp.*<br>*Funneliformis mosseae*<br>*Galactomyces spp.*<br>*Kluyveromyces marxianus*<br>*Macrophomina phaseolina*<br>*Ophiocordyceps oxycephala*<br>*Pachyphloeus virescens*<br>*Peniophora cinerea*<br>*Peronospora pulveracea*<br>*Phoma spp.*	Identification of the fungal flora in raw milk from the Italian Alps in relation to pasture altitude	[58]

		Pichia spp.<br>*Priceomyces carsonii*<br>*Psathyrella lutensis*<br>*Rhodotorula spp.*<br>*Torulaspora delbrueckii*<br>*Trichosporon spp.*<br>*Wallemia sebi*<br>*Yarrowia lipolytica*		
	ITS<br>LSU	*Amanita spp.*	Distinction between edible and poisonous amanitas mushrooms	[59]
	ITS	*Gliophorus spp.*	Identification of new species of waxcap mushrooms	[60]
	ITS<br>β-TUB<br>CAL	*Alternaria spp.*<br>*Aspergillus spp.*<br>*Botrytis spp.*<br>*Cladosporium spp.*<br>*Mucor spp.*<br>*Penicillium spp*<br>*Rhizopus spp.*	Identification of fungi in organic and conventional fruits and derived products	[61]
	SSU<br>ITS<br>RPB1<br>RPB2	*Albatrellus ellisii*<br>*Cantharellus cibarius*<br>*Clavulina cristata.*<br>*Gomphus floccosus*<br>*Inocybe aff. Sphaerospora*<br>*Laccaria vinaceoavellanea*<br>*Lactarius deliciosus*<br>*Lactarius volemus*<br>*Ramaria maculatipes*<br>*Tricholoma viridiolivaceum*	Identification of wild edible mushrooms	[62]
	ITS	*Agaricus bisporus*<br>*Armillaria spp.*<br>*Boletus spp.*<br>*Chalciporus piperatus*	Identification of wild and native mushrooms sold in the open-air markets of south-eastern Poland	[63]

		Cortinarius caperatus<br>*Imleria badia*<br>*Lactarius spp.*<br>*Leccinum schistophilum*<br>*Leucoagaricus nympharum*<br>*Pleurotus cornucopiae*<br>*Polyporus umbellatus*<br>*Sparassis crispa*<br>*Suillus spp.*<br>*Tricholoma spp.*<br>*Xerocomellus cisalpinus*		
	ITS<br>LSU	*Candida spp.*<br>*Clavispora lusitaniae*<br>*Cyberlindnera fabianii*<br>*Debaryomyces spp.*<br>*Dipodascaceae*<br>*Geotrichum spp.*<br>*Hanseniaspora spp.*<br>*Hypocreaceae*<br>*Kazachstania spp.*<br>*Kluyveromyces spp.*<br>*Lachancea spp.*<br>*Mucor spp.*<br>*Nectriaceae*<br>*Penicillium spp.*<br>*Sarocladium spp.*<br>*Sistotrema spp.*<br>*Torulaspora delbrueckii*<br>*Trichocomaceae*<br>*Wickerhamomyces anomalus*<br>*Yarrowia lipolytica*	Study of the fungal diversity in dairy products	[64]

	ITS<br>LSU	*Aspergillus spp.*<br>*Cladosporium spp.*<br>*Eurotium spp.*<br>*Hyphopichia spp.*<br>*Lichtheimia spp.*<br>*Millerozyma spp.*<br>*Monascus spp.*<br>*Mucor spp.*<br>*Paecilomyces spp.*<br>*Penicillium spp.*<br>*Pichia spp.*<br>*Rhizomucor spp.*<br>*Rhizopus spp.*<br>*Saccharomycopsis spp.*<br>*Syncephalastrum spp.*<br>*Wickerhamomyces spp.*	Identification of fungal species and enzyme activity in nuruk, a Korean fermenting starter	[65]
	ITS<br>TEF-1α<br>RPB1<br>RPB2	*Pleurotus ostreatus* complex-species	Distinction between species of oyster mushrooms	[66]
	ITS	*Agaricus bisporus*<br>*Boletus edulis*<br>*Cantharellus cibarius*<br>*Craterellus comucopioides*<br>*Grifola frondosa*<br>*Hericium erinaceous*<br>*Inonotus obliquus*<br>*Lentinula edodes*<br>*Tremetes versicolor*<br>*Wolfiporia cocos*	Identification of consumer-relevant mushrooms	[67]
	ITS	*Clitopilus prunulus*<br>*Coprinus comatus*	Identification of wild mushrooms samples	[68]

		Craterellus cornucopioides<br>*Cyclocybe cylindracea*<br>*Hymenopellis radicata*<br>*Infundibulicybe gibba*<br>*Lactarius deterrimus*<br>*Laetiporus sulphureus*<br>*Lepista nuda*<br>*Leucoagaricus leucothites*<br>*Lycoperdon perlatum*<br>*Macrolepiota procera*<br>*Paralepista flácida*<br>*Pleurotus dryinus*<br>*Psathyrella spp.*		
	ITS	*Fomitopsis spp.*<br>*Ganoderma spp.*<br>*Hexagonia spp.*<br>*Lentinus spp.*<br>*Lenzites spp.*<br>*Leucoagaricus spp.*<br>*Leucocoprinus spp.*<br>*Lycoperdon spp.*<br>*Microporus spp.*<br>*Panus spp.*<br>*Physisporinus spp.*<br>*Pleurotus spp.*<br>*Polyporus spp.*<br>*Trametes spp.*	Identification of wild mushrooms	[69]
Lichens	ITS	*Rhizoplaca melanophthalma species-complex*	Distinction of species within the complex	[70,71]
	ITS	*Cladia aggregata* species-complex	Distinction of species within the complex	[72]
	COX	*Cladonia spp.*	Identification of species	[73,74]

	ITS<br>IGS<br>RPB2<br>TEF-1α			
	ITS	*Parmelia sensu stricto*	Study of the diversity in Parmeliaceae (Ascomycota)	[75]
	ITS	*Usnea spp.*	Identification of lichen-forming fungi	[76]
	ITS	*Cora spp.*<br>*Corella spp.*	Identification of lichen-forming Basidiomycetes belonging to different genera	[77]
Medicinal products	ITS	*Ophiocordyceps sinensis*	Identification of the medicinal mushroom	[78,79]
	ITS	*Lignosus cameronensis*<br>*Lignosus tigris*	Identification of two new species of tiger milk mushroom	[80]
	ITS	*Fomes fomentarius*	Study of the genetic variability of the medicinal tinder bracket polypore	[61]
Extreme environments	ITS<br>SSU	*Ascotricha sp.*<br>*Aspergillus sp.*<br>*Cerrena sp.*<br>*Chaetomium sp.*<br>*Cladosporium sp.*<br>*Eurotium sp.*<br>*Hortaea sp.*<br>*Penicillium sp.*<br>*Pleospora sp.*<br>*Sagenomella sp.*<br>*Trametes sp.*	Identification of fungal species in deep-sea sediments	[82]
	ITS	*Melanelia spp.*	Identification and discrimination of the lichen-forming genus from Icelandic	[83]
	ACT<br>B-TUB<br>CAM<br>GAPDH<br>ITS<br>TEF-1α	*Alternaria spp.*<br>*Aspergillus spp.*<br>*Aureobasidium spp.*<br>*Candida spp.*<br>*Cladosporium spp.*<br>*Debaryomyces spp.*<br>*Fusarium spp.*<br>*Hortaea spp.*	Identification and discrimination of fungal species of the hypersaline Inland Sea	[84]

		Hyphozyma spp.<br>*Knufia spp.*<br>*Kondoa spp.*<br>*Naganishia spp.*<br>*Papiliotrema spp.*<br>*Penicillium spp.*<br>*Rhodotorula spp.*<br>*Sarocladium spp.*<br>*Symmetrospora spp.*<br>*Tremellales spp.*<br>*Trichoderma spp.*<br>*Ulocladioides spp.*<br>*Zalaria obscura*		
	ITS	*Dothideomycetes*<br>*Eurotiomycetes*<br>*Lecanoromycetes*<br>*Leotiomycetes*<br>*Pezizomycetes*<br>*Sordariomycetes*	Study of the endophytic and endolichenic fungal diversity in maritime Antarctica	[85]
Saltwater ecosystems	ITS	*Ascomycota*<br>*Basidiomycota*	Study of the diversity of marine-derived fungal cultures	[86]
	ITS<br>RPB2<br>TEF-1α	*Trichoderma arenarium*	Identification of fungal bioeffectors for biosaline agriculture in salt marshes	[87]
	ITS	*Aspergillus*<br>*Penicillium*<br>*Phialemoniopsis*<br>*Purpureocillium*<br>*Trametes*	Identification of culturable marine fungi from coastal region	[88]
Freshwater ecosystems	ITS	*Hyphomycete*	Identification of fungal species	[89]
	ITS	*Capnodiales*<br>*Eurotiales*	Identification of fungal species in Dukan Freshwater Lake	[90]

		Helotiales<br>*Hypocreales*<br>*Mortierellales*<br>*Onygenales*<br>*Pezizales*<br>*Pleosporales*<br>*Xylariales*		
Soil	ITS<br>TEF-1α	*Trichoderma spp.*	Study of the diversity in soil and litter	[91]
	ITS	*Cortinarius spp.*	Study of the diversity and geographic distribution of the ectomycorrhizal fungi	[92,93]
	ITS<br>LSU<br>RPB1<br>RPB2<br>SSU<br>TEF-1α	*Russula spp.*	Identification of species of the ectomycorrhizal mushrooms	[94]
	ITS<br>LSU	*Gongronella eborensis*	Identification of a new fungal species from vineyard soil	[95]
	B-TUB	*Exophiala pseudooligosperma*	Identification of a new black yeast from soil	[96]
Enzyme production	ITS	*Aspergillus spp.*<br>*Colletotrichum gloeosporioides*<br>*Fusarium oxysporum*<br>*Penicilliun echinulatum*	Identification of leaf litter fungi potential for cellulose degradation	[97]
	ITS	*Trichoderma spp*	Identification of fungal species and screening for cellulolytic activity	[98]
	ITS	*Aspergillus fischeri*<br>*Cosmospora viridescens.*<br>*Curvularia spicifera*<br>*Fusarium spp.*<br>*Humicola grisea*<br>*Mucor spp.*<br>*Paecilomyces sp.*<br>*Penicillium spp.*	Identification of ligno-cellulolytic fungi	[99]

		Trichoderma atroviride		
	ITS<br>TEF-1α	*Aspergillus nidulans*<br>*Stemphylium lucomagnoense*<br>*Trichoderma asperellum*	Screening of marine fungal strains for their potential to produce oxidases with laccase activities	[100]
Monument degradation	LSU	*Alternaria spp.*<br>*Aspergillus spp.*<br>*Aureobasidium spp.*<br>*Cladosporium spp.*<br>*Fusarium spp.*<br>*Mucor spp.*<br>*Penicillium spp.*<br>*Pestalotiopsis spp.*<br>*Trichoderma spp.*	Identification of culturable fungi associated with urban stone surfaces	[101]
	β-TUB<br>ITS<br>LSU<br>TEF1-α	*Bionectria* cf. *ochroleuca*<br>*Chaetomium murorum*<br>*Cladosporium spp.*<br>*Epicoccum nigrum*<br>*Fusarium proliferatum*<br>*Mucor racemosus*<br>*Penicillium crustosum*<br>*Rhizopus stolonifer*<br>*Trichoderma harzianum*	Identification and biodeteriorative potential of fungal dwellers on ancient stone stela	[102]
	ITS<br>LSU	*Aspergillus spp.*<br>*Bjerkandera adusta*<br>*Cladosporium spp.*<br>*Penicillium spp.*	Identification of contaminant fungi at different coloured materials in ancient Egypt tombs and mosques	[103]
	ITS<br>LSU	*Acremonium spp.*<br>*Lecanicillium sp.*<br>*Parengyodontium album*<br>*Purpureocillium lilacinum*<br>*Sarocladium kiliense*	Micromycetes as colonizers of mineral building materials in historic monuments and museums	[104]
	ITS	*Alternaria spp.*	Identification of fungi associated with building materials biodeterioration	[105]

		Arthothelium spectabile<br>*Arthrinium spp.*<br>*Aspergillus sp.*<br>*Aureobasidium pullulans*<br>*Cladosporium sp.*<br>*Geomyces sp.*<br>*Lasiodiplodia theobromae*<br>*Moniliella sp.*<br>*Mucor sp.*<br>*Penicillium sp.*		
	ACT<br>β-TUB<br>ITS<br>LSU<br>RPB1<br>RPB2<br>SSU<br>TEF-1α	*Anthracina spp.*<br>*Bradymyces spp.*<br>*Cladophialophora spp.*<br>*Cyphellophora spp.*<br>*Exophiala spp.*<br>*Knufia spp.*<br>*Lithohypha spp.*<br>*Trichomerium spp.*	Identification of rock-inhabiting Chaetothyriales fungi	[106]
Effluents	ITS<br>LSU	*Wickerhamiella shivajii*	Identification of a thermotolerant yeast isolated from distillery effluent	[107]
	β-TUB<br>CAM<br>ITS<br>RPB2	*Aspergillus spp.*<br>*Fusarium spp.*<br>*Penicillium spp.*<br>*Trichoderma spp.*	Identification and phylogeny of fungi isolated from industrial wastewater	[108]
Bioremediation	ITS	*Acremonium sp.*<br>*Alternaria sp.*<br>*Aspergillus spp.*<br>*Beauvaria sp.*<br>*Chaetomium spp.*<br>*Cladosporium clasdosporoides*<br>*Cryptococcus spp.*<br>*Cunninghamella elegans*	Identification and phylogeny of fungi associated with tarballs	[109]

		Epicoccum purpurascens<br>*Fusarium sp.*<br>*Graphium sp.*<br>*Humicola fuscoatra*<br>*Microascus cinereus*<br>*Mucor sp.*<br>*Paecilomyces sp.*<br>*Passalora sp.*<br>*Penicillium spp.*<br>*Pseudallescheria ampullus*<br>*Rhizopus spp.*<br>*Rhodotorula sp.*<br>*Saccharomyces estuary*<br>*Sarocladium spp.*<br>*Scopulariopsis brevicaulis*<br>*Sordaria spp.*<br>*Syncephalastrum racemosum*<br>*Tetracoccosporium sp.*		
	β-TUB<br>ITS	*Acremonium spp.*<br>*Alternaria spp.*<br>*Aspergillus spp.*<br>*Beauveria spp.*<br>*Curvularia spp.*<br>*Emericellopsis spp.*<br>*Exophiala spp.*<br>*Fusarium spp.*<br>*Sarocladium spp.*<br>*Stachybotrys spp.*<br>*Stemphylium spp.*<br>*Trichoderma spp.*<br>*Ulocladium spp.*	Study of the diversity of culturable fungi inhabiting petroleum-contaminated soils	[110]
	ITS	*Trichoderma spp.*	Identification of the fungus	[111]

	RPB2<br>TEF-1α			
	ITS	*Aspergillus spp.*<br>*Cladosporium spp.*<br>*Exophiala spp.*<br>*Flavodon spp.*<br>*Hypocreales*<br>*Nectriaceae*<br>*Penicillium spp.*<br>*Peniophora spp.*<br>*Trichoderma spp.*	Identification of culturable petroleum-degrading fungi from petroleum-contaminated sites	[112]
	β-TUB<br>ITS<br>TEF-1α	*Aspergillus*<br>*Chaetomella*<br>*Cladosporium*<br>*Cochliobolus*<br>*Diaporthe*<br>*Epicoccum*<br>*Eutypella*<br>*Fusarium*<br>*Gongronella*<br>*Microsphaeropsis*<br>*Myrothecium*<br>*Neoascochyta*<br>*Neocosmospora*<br>*Oudemansiella*<br>*Paraconiothyrium*<br>*Paraphaeosphaeria*<br>*Penicillium*<br>*Perenniporia*<br>*Periconia*<br>*Phanerochaete*<br>*Phoma*	Diversity and oil degradation potential of culturable fungi isolated from chronically contaminated soils	[113]

		Phytophthora<br>*Pyrenochaetopsis*<br>*Rhizopus*<br>*Roussoella*<br>*Saccharicola*<br>*Scedosporium*<br>*Sydowia*<br>*Talaromyces*<br>*Trichoderma*<br>*Westerdykella*		

Supplementary Table S9. Molecular markers in fungi: an overview of manuscripts published in the last 10 years using Metagenomics (COX2; cytochrome c oxidase subunit II, ITS1: internal transcribed spacer regions of the nrDNA operon (region 1), ITS2: internal transcribed spacer regions of the nrDNA operon (region 2), LSU: 28S nrDNA, SSU: 18S nrDNA, TEF-1α: translation elongation factor 1-alpha,).

Importance	Metabarcode	Purpose	Reference
Human health	ITS1 / ITS2	Identification of fungal species in the gut of HIV-infected patients	[1]
	SSU	Mosquito vector-associated microbiota across habitat endemic for dengue and other arthropod-borne diseases	[2]
	ITS2	Mycobiome in the middle ear cavity with and without otitis media with effusion	[3]
	ITS	Oral microbiome in down syndrome and its implications on oral health	[4]
	ITS	Comparison of fungal species from the oral microbiome in cystic fibrosis patients and controls	[5]
	ITS2	Identification of the human oral mycobiome in tissue and saliva	[6]
	ITS	Global distribution of the emerging pathogenic fungus *Scedosporium aurantiacum*	[7]
	ITS	Changes in the oral microbiome through age and lifestyle factors	[8]
Animal health	ITS<br>LSU	Identification of saprophytic Polyporales mutualists of *Ambrosiodmus ambrosia* beetles	[9]
	ITS	Identification of fungal communities from bark beetles	[10]
	ITS2	Identification of the fungal microbiota associated to the olive fruit fly	[11]
	ITS1	Study of endomycobiome associated with females of the planthopper *Delphacodes kuscheli*	[12]
	ITS2	Identification of mixed strongyle infections in horses	[13]
	ITS2	Study of fungal diversity from feces of small mammals	[14]
	ITS2	Identification of fungal entomopathogens from *Aedes albopictus* larvae	[15]
	ITS2<br>LSU	Identification of insect-associated fungal communities	[16]
Plant health	ITS2	Identification of fungal diversity in the phyllosphere and carposphere of olive (*Olea europaea*)	[17]
	ITS1	Identification interactions of the etiological agent of oak powdery mildew from pedunculate oak	[18]
	ITS1 / ITS2<br>LSU	Identification of grapevine trunk pathogens in planta	[19,20]
	ITS	Identification of fungal community diversity associated with Norway spruce	[21]
	TEF-1α	Assessment of *Fusarium* diversity on cereals	[22]
	ITS	Identification of the fungal community in leaves of *Acanthus ilicifolius* var. *xiamenensis*	[23]

	ITS<br>TEF-1α	Identification of *Fusarium* communities in maize stalks	[24]
	ITS1	Fungicides have complex effects on the wheat phyllosphere mycobiome	[25]
	ITS1	Identification of soil fungal and oomycete communities in holm oak declined	[26]
	COX2	Identification of oak rhizosphere-associated oomycetes	[27]
	ITS1	Identification of fungal species from wheat ears across a topographically heterogeneous field	[28]
	ITS1<br>ITS2	Identification of Botryosphaeriaceae fungal endophytes in grapevines and adjacent forest trees	[29]
	ITS	Identification of fungal endophyte communities in *Theobroma cacao* leaves	[30]
	ITS1	Identification of endophytic fungal community from *Olea europaea*	[31]
	ITS2	Identification of the fungal endophytic assemblage of *Brachypodium rupestre* growing in a range of anthropized disturbance regimes	[32]
	ITS2	Identification of fungal endophytes within wood of cultivated Proteaceae	[33]
	ITS2	Study of fungal endophyte diversity in the phyllosphere of a barley crop	[34]
	ITS1 / ITS2	Detection and surveillance of emerging and recurrent plant pathogens	[35]
	ITS2	Identification of fungal assemblages in fallen Norway spruce trunks	[36]
	ITS2	Detection and identification of rust fungal pathogens in environmental samples	[37]
	ITS2	Identification of fungal communities in Heshouwu (*Polygonum multiflorum* Thunb.)	[38]
	ITS2	Identification of endophytic fungal communities in *Vaccinium myrtillus* plant organs	[39]
	ITS1 / ITS2	Identification of weed-colonizing fungi	[40]
	ITS1	Study of *Phytophthora* diversity using genus-specific primers	[41]
	ITS1	Study of *Phytophthora* diversity in anthropized and natural ecosystems	[42]
Food and beverage	ITS1 / ITS2	Identification of fungal communities in brined Aloreña de Málaga green olive fermentations	[43]
	ITS	Identification of fungal species associated with spontaneous wine fermentation	[44]
	ITS	Identification from fungal species from dairy products and Mayonnaise	[45]
	ITS2	Identification of fungal species from traditionally prepared starters for alcoholic beverages in India	[46]
	ITS	Identification of fungal species from spontaneous fermentation of Chinese pu-erh tea	[47]
	ITS2	Identification of fungal species in honey samples from local beekeepers and markets	[48]
	ITS2	Identification of fungal species of barley grain	[49]
	ITS2	Identification of fungal contaminants in nutmeg	[50]
	ITS1 / ITS2	Identification of fungal species from Doushen with different flavors	[51]

	ITS2	Identification of fungal species associated with gwell	[52]
	LSU	Identification of fungal species associated with the Algerian traditional date product "Btana"	[53]
	ITS	Identification of fungal species from apple fruit after harvest and implications for fruit quality	[54]
	ITS	Identification of fungal species from French fermented sausages and mycotoxin risk evaluation during storage	[55]
	ITS1	Authentication of "wild mushrooms" in food products	[56]
	ITS1	Identification of fungal species from artisanal colonial salami-type dry-fermented sausages	[57]
	ITS	Identification of fungal species from kombucha tea	[58,59]
	ITS2	Identification of natural fungal contaminants in bee pollen	[60]
	ITS1	Identification of fungal communities from Greek PDO cheeses	[61]
	ITS2	Identification of desirable and spoilage fungi sources during black olive fermentations	[62]
	ITS2	Study of seasonal changes in the fungal species from an Australian Winery	[64]
	ITS1	Identification of truffles	[65]
	ITS2	DNA traces the origin of honey by identifying fungi	Wirta et al., [2021]
	ITS	Identification of fungal species from Mexican tepache fermented beverage	[66]
	ITS1 / ITS2	Identification of fungal species from flaxseed	[67]
Lichens	ITS2	Identification of fungal species in lichens	[68–70]
	ITS1	Identification of fungal species of lichen forming-fungi at a local scale	[71]
	ITS1 / ITS2	Identification of fungal species in photobiont lichens	[72]
	ITS	Identification of lichen-forming fungi from herbarium specimens	[73,74]
	ITS1 / ITS2	Study of the biodiversity of lichen-forming fungi	[75]
	ITS1	Identification of endolichenic fungal community of *Parmotrema tinctorum*	[76]
Medicinal products	LSU	Identification of yeasts and fungi from kombucha probiotic beverage	[77,78]
	ITS<br>TEF-1α	Identification the "mushroom of immortality": assessing the Ganoderma species composition in commercial Reishi products	[79]
	ITS2	Identification of fungal communities in medicinal and edible Cassiae Semen	[80]
	ITS2	Identification of biological ingredient from traditional herbal medicine Fuke Desheng Wan	[81]
Extreme environments	ITS	Fungal paleo-diversity from arctic permafrost	[823]
	ITS1	Identification of cryptoendolithic fungal communities in Antarctica	[83–85]

	ITS	Identification of fungal communities from Castaño Overa glacier–Mount Tronador, Patagonia, Argentina	[86]
	SSU	Study of fungal diversity of a shallow-water hydrothermal vent field at Kueishan Island, Taiwan	[87]
	ITS1	Identification of fungal communities from rock and soil substrates on a maritime antarctic glacier forefield	[88]
	ITS2	Identification of fungal diversity in soils from Antarctica	[89,90]
	ITS1 / ITS2	Identification of fungal communities from the continental solar saltern in Añana Salt Valley	[91]
	ITS2	Identification of fungal diversity present on rocks from a polar desert from Antarctica	[92]
	ITS2	Identification of fungal species present in lakes of Maritime Antarctica	[93,94]
	ITS	Identification of fungal communities present in Antarctic deep-sea sediments	[95]
	ITS2	Identification of fungal communities from permafrost in the South Shetland Islands, maritime Antarctic	[96]
	ITS	Identification of fungal communities from Arctic paleoecosystems	[97]
Marine ecosystems	ITS2	Diversity assessment of fungi from protected coastal *Salix repens* communities	[98]
	SSU	Identification of fungal communities from estuaries.	[99]
	SSU	Identification of fungal species from sediments of marine canyons	[100]
	ITS	Identification of fungal communities in sediments of subtropical Chinese seas	[101]
	SSU	Identification of fungal communities in marine sediments	[102]
	ITS2	Identification of fungal communities in unflooded and tidal flat soil in coastal saline ecosystem	[103]
	ITS<br>SSU	Identification OF fungal species from sediments from Stellwagen Bank National Marine Sanctuary	[104]
	ITS2	Identification of fungal communities on the Swedish west coast	[105]
	SSU	Study of the diversity of endolithic fungi in coral skeletons and other reef substrates	[106]
	ITS2	Study of fungal diversity in deep-sea sediments from the Magellan seamounts	[107]
Freshwater ecosystems	SSU	Identification of fungal diversity on coarse and fine particulate organic matter in a first-order stream	[108]
	ITS	Identification of aquatic fungi on deciduous leaves	[109]
	ITS1	Study of spatial structure of fungal DNA assemblages of a forest river network	[110]
	ITS	Identification of fungal airborne communities from rice paddy	[111]
	ITS1	Study of seasonal dynamics of fungal communities in a flow-regulated stream in a restored forest	[112]
	ITS	Identification of fungal species from microplankton in lower reaches of Qiantang River	[113]
Built environment	ITS	The metagenomics and metadesign of the subways and urban biomes	[114]
	ITS1	Changes in fungal species associated with urban stress	[115]

	ITS	A global metagenomic map of urban microbiomes and antimicrobial resistance	[116]
	ITS	Urban environments harbor greater oomycete and Phytophthora diversity, creating a bridgehead for potential new pathogens to natural ecosystems	[117]
	ITS1 / ITS2	Identification of fungal community and potential pathogen identification across the PM size fractions and seasons in the urban atmosphere	[118]
Pollution	ITS2	Identification of belowground fungal communities in pioneer Scots pine stands growing on heavy metal polluted and non-polluted soils	[119]
	ITS<br>SSU	Identification of plastic-associated species in the Mediterranean Sea	[120]
	ITS1 / ITS2	Identification of fungal communities in microplastics from terrestrial ecosystems	[121]
	SSU	Identification of fungal species and tolerance to contaminants in an urban brownfield site	[122]
	ITS	Identification of fungal communities at differential polluted sites of Rome	[123]
**Soil**	SSU	Identification of fungi in communities from a decommissioned gold mine	[124]
	ITS	Identification of fungal communities from poplar at a Hg phytomanagement site	[125]
	ITS1	Identification of mycorrhizal and pathogenic fungi from the soil of a Eucalyptus plantation	[126]
	ITS	Study of the diversity of mycorrhizal fungi in forest soils	[127]
	ITS1	Identification of soil mycobiota of the upper Andean Colombian agro-environment	[128]
	ITS	Identification of the diversity of woody host-associated *Phytophthora* spp. in soils at public gardens and amenity woodlands	[129]
	ITS	Fungal communities decline from the air and soil associated with urbanization	[130]
	ITS1 / ITS2	Identification of soil fungal communities in metropolitan green spaces across a vegetation biodiversity gradient	[131]
	ITS	Study of soil microbial communities in diverse agroecosystems exposed to the herbicide glyphosate	[132]
	ITS1	Study of soil fungal communities differ between shaded and sun-intensive coffee plantations	[133]
	SSU	Identification of arbuscular mycorrhizal fungal communities colonising the roots of indigenous legumes	[134]
	ITS1 / ITS2<br>LSU	Identification of soil fungi from different urban greenspaces	[135]
	ITS2	Identification of soil fungal communities associated with alpine field-grown saffron (*Crocus sativus* l.) inoculated with AM fungi	[136]
	SSU	Study in changes of fungal communities associated with the conversion of a High-Altitude temperate forest to agriculture fields or husbandry	[137]

	ITS	Identification of plant pathogenic oomycetes in soil from internationally traded plants	[138]
	ITS1 / ITS2	Identification of multiple mycetoma pathogens from soil	[139]
	ITS2	Identification of the soil fungal community to aid the conservation of underexplored church forests	[140]
	ITS2	Identification of cryptic diversity in forest soils on the isolated Brazilian Trindade Island	[141]
	ITS2	Identification of fungal communities among Amazonian Canga formations	[142]
Outdoor air aerobiology	ITS2	Identification of fungal spores from mixed airborne samples	[143]
	SSU	Identification of fungal species from the Red Sea Sector of the Global Dust Belt's Microbiome	[144]
	ITS2	Assessment of airborne fungal seasonal diversity	[145]
	SSU	Identification of the aerobiome and potential drivers of turn-over in the full microbial community in the air	[146]
	ITS2	Spatio-temporal patterns of airborne fungal spores	[147]
	ITS2	Climate change impact on fungi in the atmospheric microbiome	[148]
Indoor air aerobiology	ITS2	Identification of indoor fungi	[149,152]
	ITS2	Identification of fungal species of sawmills	[153]
	ITS	Cartography of opportunistic pathogens and antibiotic resistance genes in a tertiary hospital environment	[154]
	ITS1 / ITS2	The diversity and seasonality of the indoor mycobiome	[155]
	ITS2	Indoor mycobiome of daycare centers	[156]
	ITS2	Identification of fungal species from school classrooms surrounded by a forest	[157]
Forensic Sciences	SSU	Predicting the origin of soil evidence: high throughput eukaryote sequencing applied to a crime scene scenario	[158]
	ITS2	Predicting provenance of forensic soil samples	[159]
	ITS	Identification of fungal succession during mammalian cadaver decomposition and potential forensic implications	[160]
	ITS1 / ITS2	Identification of soil fungal communities investigated in simulated forensic burial contexts	[161]
	ITS1 / ITS2	Identification of fungal species from forensic samples	[162,164]
	ITS1	Link between evidence and crime scene through soil fungal microbiome	[165]
Compost	ITS1 / ITS2	Identification of fungal species from home composts	[166]
	ITS	Biodiversity and succession of mycobiota associated to agricultural lignocellulosic waste-based composting	[167]

	ITS1	Identification of fungal communities in pressmud composting harbour beneficial and detrimental fungi for human welfare	[168]
	SSU	Fungal community in the composting process of Chinese medicinal herbal residues	[169]
Monument degradation	ITS	Identification of epilithic and endolithic fungi associated with deterioration on stone church facades subject to urban pollution in a sub-tropical climate	[170]
	ITS	Identification of fungal species from the dark crusts on the church of Nossa Senhora do Carmo, Rio de Janeiro, Brazil	[171]
	ITS	Identification of fungal species in the first Portuguese King tomb	[172]
	ITS2	Study of biodeterioration of ancient wall paintings in an Italian cave	[173]
	ITS1	Identification of fungal communities from darkened white marble of Florence cathedral	[174]

Supplementary Table S10. Molecular markers in fungi: an overview of manuscripts published in the last 10 years using Whole Genome Sequencing.

Importance	Fungal identification	Purpose	Reference
Human health	*Apophysomyces trapeziformis*	Sequence of the etiological agent of necrotizing cutaneous mucormycosis	[1]
	Exserohilum rostratum	Detection of the etiological agent of fungal meningitis and other infections	[2,3]
	Sporothrix schenckii	Sequence of the etiological agent of sporotrichosis	[4]
	Scedosporium apiospermum	Sequence of the pathogenic fungus	[5]
	Candida auris	Sequence of the etiological agent of candidiasis with multi-drug resistances	[6–12]
	Aspergillus fumigatus	Comparison of strains isolated from patients with pulmonary aspergilloma and chronic necrotizing pulmonary aspergillosis	[13]
	Aspergillus lentulus	Sequence of the etiological agent of aspergillosis	[14]
	Penicillium capsulatum	Sequence of the potential pathogenic fungus	[15]
	Histoplasma capsulatum	Sequence of the etiological agent of lung infections	[16]
	Mucor circinelloides f. circinelloides	Sequence of the etiological agent of invasive wound mucormycosis in a burn unit	[17]
	Trichophyton rubrum	Sequence of an ubiquitous dermatophyte pathogen	[18]
	Candida inconspicua	Sequence of the etiological agent of candidiasis	[19]
	Aspergillus terreus complex-species	Sequence of the etiological agent of invasive infection and chronic respiratory disease	[20]
Animal health	*Leptographium longiclavatum*	Sequence of a mountain pine beetle-associated symbiotic fungus	[21]
	Metarhizium anisopliae	Sequence of the fungus used as biocontrol agent of insect pests	[22]
	Purpureocillium lilacinum	Comparative genomic of bio-control fungus against nematodes	[23]
	Aschersonia badia<br>*Metarhizium spp.*	Sequence and comparative analysis of two insect-pathogenic fungus	[24]
	Beauveria bassiana	Sequence of an entomopathogenic fungus	[25]
	Herpomyces periplanetae	Sequence of a cockroach-infecting fungus	[26]
	Sarocladium terricola	Sequence of an endophytic fungus with potential biocontrol activity against *Meloidogyne incognita*	[27]
Human and animal health	*Geomyces pannorum sensu lato*<br>*Geomyces destructans*	Sequences of human pathogenic fungus and bat white nose syndrome pathogen	[28]
	Aspergillus udagawae	Genome sequence of the pathogenic filamentous fungus	[29]
	Malassezia pachydermatis	Sequence of the animal and human pathogen strain	[30]
	Lomentospora prolificans	Sequence of an opportunistic fungal pathogen causing infections in immunocompromised and immunocompetent people and animals	[31]
Plant health	*Eutypa lata*	Sequence of the etiological agent of grapevine dieback	[32]

	Neofusicoccum parvum	Sequence of the etiological agent of grapevine cankers	[33]
	Rhizoctonia solani	Sequence of the phytopathogenic fungus	[34,35]
	Sporisorium scitamineum	Sequence of the etiological agent of smut from sugarcane	[36]
	Fusarium virguliforme	Sequence of the etiological agent of sudden death syndrome in soybean	[37]
	Diplodia sapinea<br>*Ceratocystis manginecans*<br>*Ceratocystis moniliformis*	Sequences of phytopathogenic fungal species	[38]
	Trichoderma harzianum	Sequence of the fungus used as biostimulants and biopesticides agent	[39]
	Neonectria ditissima	Comparison of two isolates of the plant-pathogenic fungus that differ in virulence	[40]
	Phytophthora fragariae var. fragariae	Sequence of a quarantine plant-pathogenic fungus	[41]
	Fusarium graminearum	Sequence of the pathogenic ascomycete fungus	[42]
	Dactylonectria macrodidyma	Sequence of a plant-pathogenic fungus	[43]
	Ceratocystis eucalypticola<br>*Chrysoporthe cubensis*<br>*Chrysoporthe deuterocubensis*<br>*Davidsoniella virescens*<br>*Fusarium temperatum*<br>*Graphilbum fragrans*<br>*Penicillium nordicum*<br>*Thielaviopsis musarum*	Sequences of phytopathogenic fungal species	[44]
	Trametes hirsuta	Sequence of the etiological agent of white-rot	[45]
	Sporisorium scitamineum	Sequence of and biotrophic interaction transcriptome with sugarcane	[46]
	Ceratocystis fagacearum<br>*Ceratocystis harringtonii*<br>*Grosmannia penicillate*<br>*Huntiella bhutanensis*	Sequences of important phytopathogenic fungi	[47]
	Diaporthe aspalathi	Sequence of the etiological agent of stem canker in soybean	[48]
	Calonectria pseudonaviculata	Diagnosis of Sarcococca blight	[49]
	Fusarium graminearum<br>*Fusarium meridionale*<br>*Fusarium asiaticum*	Comparative genomics of closely related Fusarium head blight fungus	[50]
	Postia placenta	Sequence of the etiological agent of brown-rot	[51]
	Fomitopsis palustris	Sequence of wood rot fungus with cellulolytic and ligninolytic enzyme system	[52]

	Athelia rolfsii	Whole genome sequence of groundnut stem rot fungus	[53]
	Sclerotinia sclerotiorum	Sequence of the phytopathogenic fungus	[54]
	Tilletia indica	Sequence of Karnal bunt pathogen of wheat	[55]
	Polyporus brumalis	Sequence of the wood rot fungus	[56]
	Diplocarpon rosae	Sequence of the etiological agent of rose black spot	[57]
	Colletotrichum truncatum	Sequence of the etiological agent of anthracnose in chilli	[58]
	Monilinia spp.	Sequence of the etiological agent of brown rot	[59]
	Ganoderma boninense	Sequence of the etiological agent of basal stem rot on oil palm	[60]
	Alternaria solani	Sequence of the etiological agent of early blight from potato and tomato	[61]
	Venturia inaequalis<br>*Venturia pirina*<br>*Venturia aucupariae*<br>*Venturia asperata*	Sequence of the scab fungal species	[62]
	Didymella segeticola	Sequences of the etiological of leaf spot from tea	[63]
	Paraphaeosphaeria sporulosa	Sequence of a plant pathogen, biocontrol, bioremediator, and endophytic fungus	[64]
	Cryphonectria parasitica	Sequence of the etiological agent of blight on chestnut	[65]
	Diaporthe capsici	Sequence of the etiological agent of blight from walnut	[66]
	Puccinia striiformis f. sp. tritici	Sequence of the etiological agent of stripe rust from wheat	[67]
	Stagonosporopsis cucurbitacearum	Analyses of putative pathogenicity genes of the etiological agent of pumpkin gummy stem blight fungus	[68]
	Colletotrichum lupini	Sequence of a plant-pathogenic fungus	[69]
	Schizophyllum commune	Sequence of the etiological agent of white-rot	[70]
	Trichoderma virens<br>*Trichoderma asperellum*<br>*Trichoderma atroviride*	Sequences of fungal species used as biocontrol agents or biofertilizers	[71]
	Calonectria ilicicola	Sequence of the etiological agent of red crown rot from soybean	[72]
	Botryosphaeria dothidea	Sequence of the etiological agent of trunk canker from Chinese hickory	[73]
	Ganoderma lucidum	Sequence of the etiological agent of white	[74]
	Nigrospora oryzae	Sequence of an pathogenic fungus	[75]
	Claviceps spp.	Comparisons of ergot fungi	[76]
	Fusarium meridionale	Sequence of the etiological agent of Fusarium head blight in rice	[77]
	Fusarium oxysporum	Sequence Resource of a plant growth promoting endophytic fungus from *Tetrastigma hemsleyanum*	[78]

	Arthrinium rasikravindrae	Sequence of a phytopathogen	[79]
	Albifimbria verrucaria	Sequence of a biocontrol fungal pathogen	[80]
Food and beverage	*Penicillium digitatum*	Sequence of the necrotrophic fungus considered as the main postharvest pathogen of citrus	[81]
	Agaricus bisporus	Sequence of the button mushroom reveals mechanisms governing adaptation to a humic-rich ecological niche	[82]
	Volvariella volvacea	Sequence and comparative genome analysis of the straw mushroom	[82]
	Brettanomyces spp.	Sequence of yeasts associated with spoilage from beer and wine	[83]
	Aspergillus nomius	Sequence of the aflatoxigenic filamentous fungus	[84]
	Aspergillus flavus	Sequence of a strain that causes aflatoxin contamination of food and feed	[85]
	Lentinula edodes	Sequence of the edible cultivated mushroom	[86]
	Aspergillus flavus<br>*Aspergillus parasiticus*	Sequences of strains isolated from peanut seeds	[87]
	Aspergillus westerdijkiae	Sequence of a fungus contaminating several food products	[88]
	Flammulina velutipes	Sequence of the edible mushroom	[89]
	Listeria monocytogenes	Detection of a listeriosis outbreak of caused by cold-smoked salmon	[90]
	Cordyceps guangdongensis	Sequence of an edible and potential medicinal fungus	[91]
	Penicillium citrinum	Sequence of the fungus responsible for citrinin production	[92]
	Penicillium expansum<br>*Penicillium solitum*	Comparisons of species with different secondary metabolic gene clusters and candidate genes associated with fungal aggressiveness during apple fruit decay	[93]
	Auricularia heimuer	Sequence of the third most important cultivated mushroom	[94]
	Hericium erinaceus	Sequence of an edible-medicinal mushroom	[95]
	Russula griseocarnosa	Sequence of a wild edible mushroom	[96]
	Naematelia aurantialba	Sequence of an edible-medicinal fungi	[97]
	Stropharia rugosoannulata	Sequence of the edible mushroom Daqiugaigu	[98]
Genomic diversity	*Stachybotrys spp.*	Identification of chemotype-specific gene clusters	[99]
	Aspergillus fumigatus	Sequence data uncover azole resistance mutations	[100]
	Helicobacter pylori	Study of sequence diversity within single strains	[101]
	Aspergillus flavus	Comparison of L-morphotype strain NRRL 3357 (type) and S-morphotype strain AF70	[102]
	Aspergillus ochraceus	Comparative genomic analysis of ochratoxin A biosynthetic pathway	[103]
	Lecanoromycetes	Sequence data uncover heterothallism in lichen-forming fungi	[104]
	Aspergillus fumigatus	Study of the virulence potential of clinical and environmental isolates	[105]
Lichens	*Caloplaca flavorubescens*	Sequence of the lichen-forming fungus	[106]

	Cladonia macilenta	Sequence of the lichen-forming fungus producing biruloquinone	[107]
	Umbilicaria muehlenbergii	Sequence of the lichen-forming fungus	[108]
	Cladonia metacorallifera	Sequence of the lichen-forming fungus	[109]
	Endocarpon pusillum	Sequence of the lichen-forming fungus	[110]
	Arthonia radiata	Sequence of the lichen-forming fungus	[111]
	Lasallia hispanica	Sequence of the lichen-forming fungus	[112]
	Ramalina intermedia	Sequence of the lichen-forming fungus	[113]
	Bacidia gigantensis	Sequence of the lichen-forming fungus	[114]
Medicinal products	*Ganoderma lucidum*	Sequence of the model medicinal mushroom	[115]
	Cordyceps militaris	Sequence of the insect pathogenic fungus valued in traditional Chinese medicine	[116]
	Phellinus gilvus	Sequence of a medicinal fungus	[117]
	Sanghuangporus sanghuang	Sequence of a medicinal fungus	[118]
	Amauroderma rugosum	Sequence of a traditional medicinal musroom	[119]
	Ganoderma leucocontextum	Sequence of a fungus with potential pharmacological activities	[120]
	Keithomyces neogunnii	Sequence of a medicinal fungus	[121]
Extreme environments	*Hortaea werneckii*	Genome of the extremely halotolerant black yeast	[122]
	Wallemia ichthyophaga	Sequence of the halophilic fungus	[123]
	Rhizomucor miehei	Sequence of the thermophilic zygomycete fungus	[124]
	Cryomyces antarcticus	Sequence of the most extremophilic fungus from Antarctica	[125]
	Rachicladosporium antarcticum<br>*Rachicladosporium sp.*	Sequences of the Antarctic endolithic fungi	[126]
	Aspergillus niger<br>*Aspergillus terreus*<br>*Aureobasidium pullulans*<br>*Beauveria bassiana*<br>*Cladosporium cladosporioides*<br>*Cladosporium sphaerospermum*<br>*Fusarium solani*<br>*Trichoderma virens*	Effects of exposure to microgravity at the International Space Station in radiation-tolerant microorganisms	[127]
Marine ecosystems	*Candida auris*	Sequence from isolates from the coastal wetlands of Andaman Islands	[128]
Built environment	*Trichoderma atroviride*	Sequence of a cosmopolitan fungus	[129]

Soil	*Rhizophagus irregularis*	Sequence of the arbuscular mycorrhizal fungus	[130]
	Russula griseocarnosa	Sequence of an edible and medicinal mushroom and its association with mycorrhizal characteristics	[131]
Bioremediation	*Scopulariopsis brevicaulis*	Fungal degradation of polycyclic aromatic hydrocarbons (PAHs) by and its application in bioremediation of PAH-contaminated soil	[132]
	Arthrobacter alpinus	Sequence of a fungus potentially used for bioremediation	[133]
	Lenzites elegans	Sequence of a Lignolytic mushroom used for the bioremediation of synthetic dyes	[134]
	Rhodotorula taiwanensis	Sequence of a potential fungal species used for the bioremediation of acidic radioactive waste sites	[135]
	Rhodotorula sp.	Sequence of an acidophilic strain and its phenol-degrading capability under acidic conditions	[136]
Monument degradation	*Knufia petricola*	Sequence of the black rock fungus isolated from marble	[137]
Enzyme production	*Thermomyces lanuginosus*	Sequence of a compost-loving thermophilic fungus considered as a xylanase super-producer:	[138]
	Flammulina velutipes	Sequence of a mushroom with high capacity for lignocellulose degradation	[139]
	Chaetomium globosum	Sequence of the cellulolytic fungus	[140]
Industrial production	*Glarea lozoyensis*	Sequence of the pneumocandin producing fungus	[141,142]
	Pseudozyma aphidis	Genome sequence of an efficient producer of biosurfactant mannosylerythritol lipids	[143]
	Umbelopsis isabellina,	Genome sequence of an effective producer of lipids	[144]
	Shiraia sp.	Analysis of the huperzine A and hypocrellin A producing fungus	[145]
	Monascus purpureus	Pigment biosynthesis and regulatory mechanisms in an industrial strain	[146]
	Aspergillus terreus	Sequence of a producing lovastatin fungus	[147]
	Aspergillus terreus<br>*Diaporthe ampelina*	Comparative study of the two lovastatin producing fungus	[148]
